# Supplementary material for: Detection of syrup adulterants in manuka and jarrah honey using HPTLC-multivariate data analysis
Source: PeerJ. 2021 Sep 22;9:e12186. doi: 10.7717/peerj.12186 (PMC8464195; doi:10.7717/peerj.12186)
Supplement: Supplemental Information 1 [file peerj-09-12186-s001.pdf]

Class: MAN

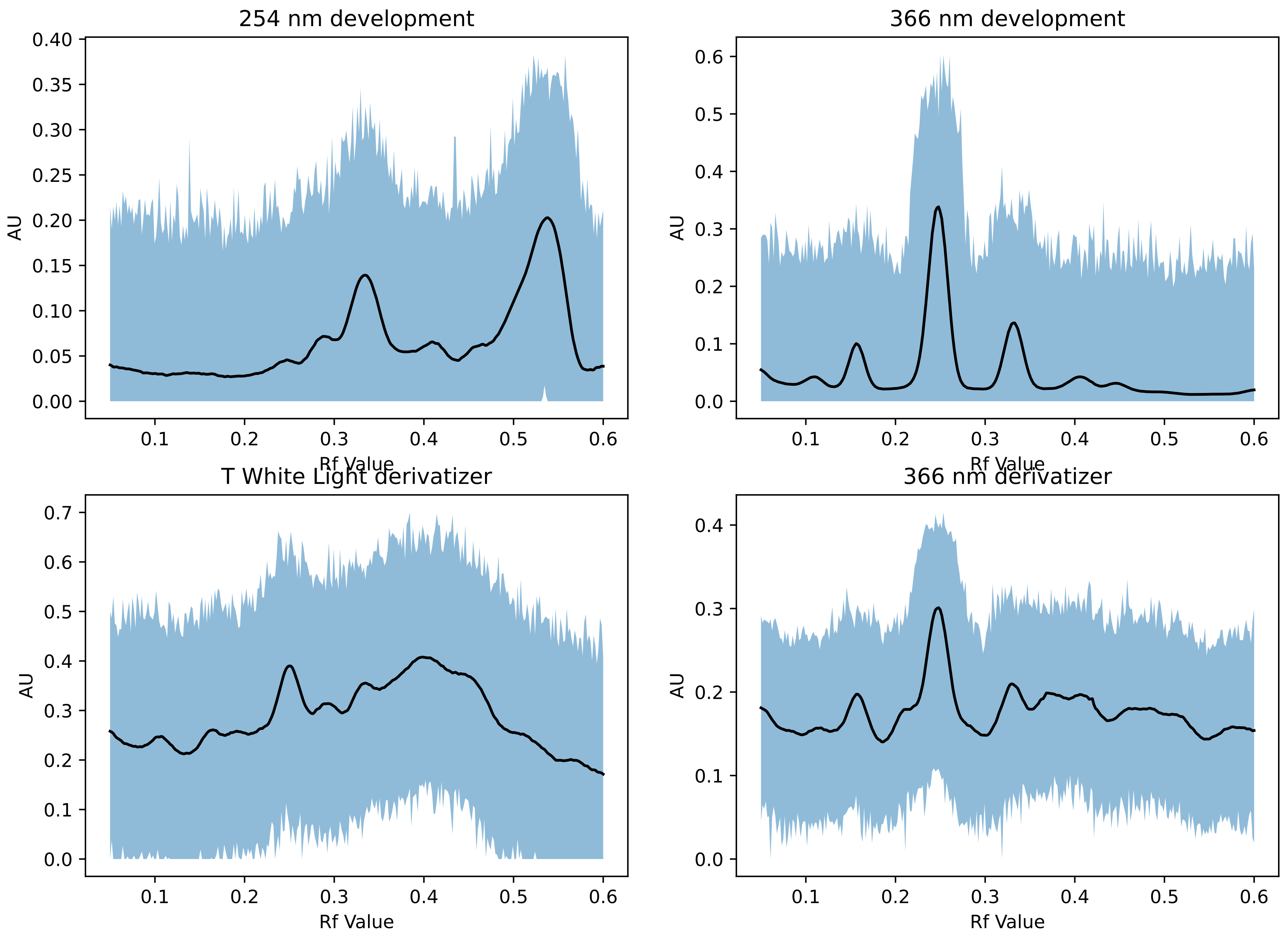

254 nm development
366 nm development
T White Light derivatizer
366 nm derivatizer
AU
Rf Value
0.40
0.35
0.30
0.25
0.20
0.15
0.10
0.05
0.00
0.7
0.6
0.5
0.4
0.3
0.2
0.1
0.0

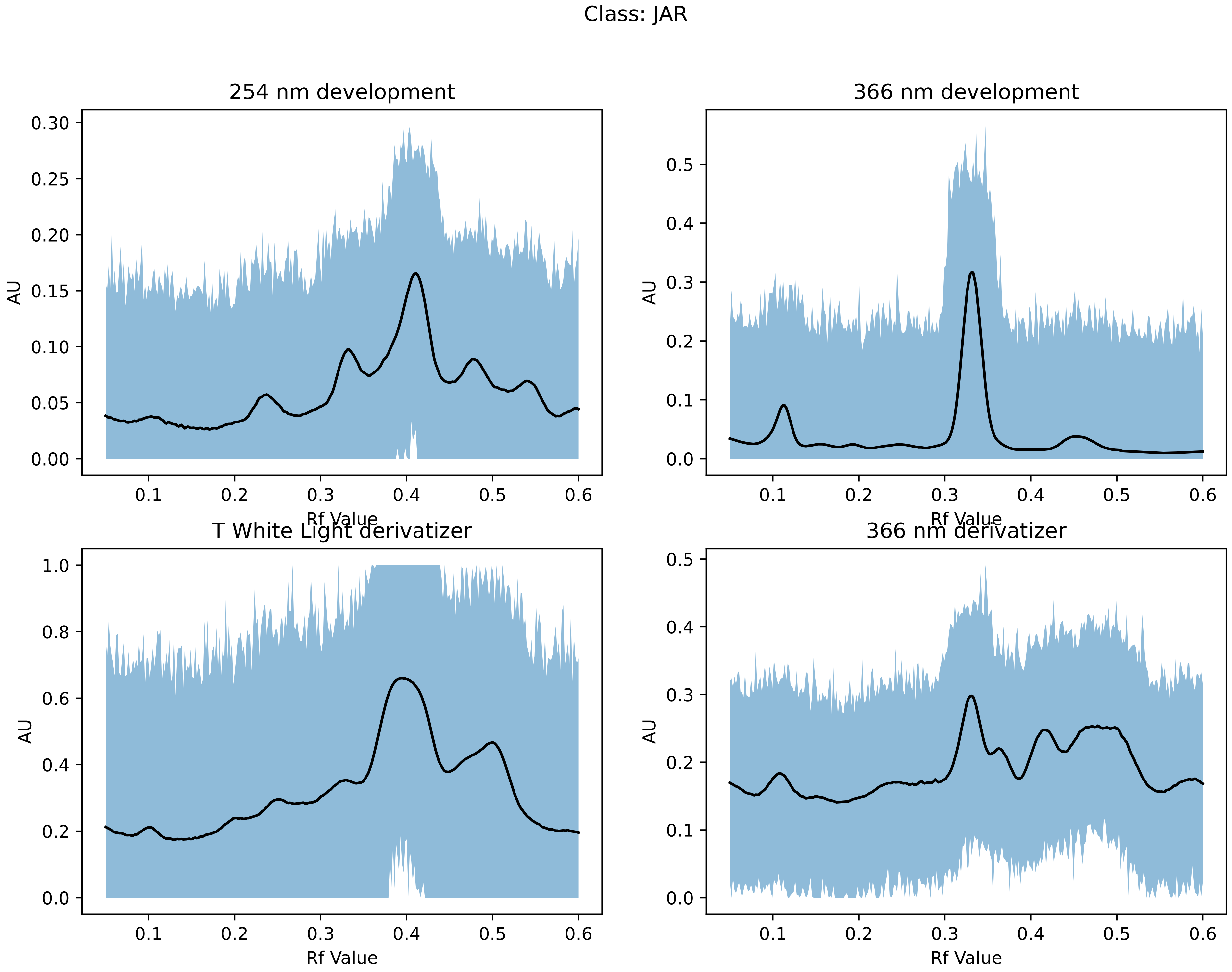
Class: JAR
254 nm development
366 nm development
T White Light derivatizer
366 nm derivatizer
AU
Rf Value
0.00
0.05
0.10
0.15
0.20
0.25
0.30
0.0
0.1
0.2
0.3
0.4
0.5
0.6
0.8
1.0

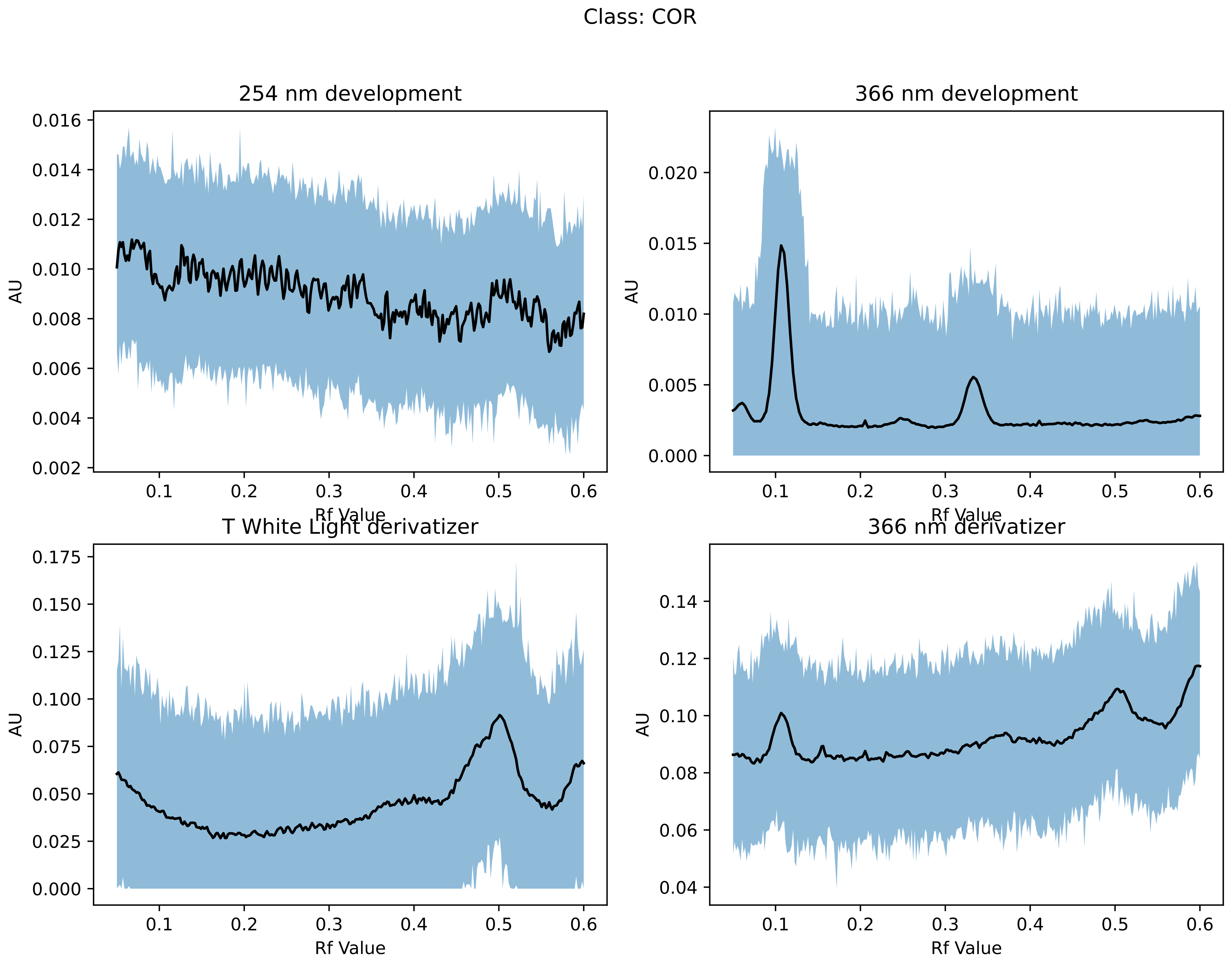
Class: COR
254 nm development
366 nm development
T White Light derivatizer
366 nm derivatizer
AU
Rf Value
0.016
0.014
0.012
0.010
0.008
0.006
0.004
0.002
0.020
0.015
0.005
0.000
0.175
0.150
0.125
0.100
0.075
0.050
0.025
0.14
0.12
0.10
0.08
0.06
0.04
0.1
0.2
0.3
0.4
0.5
0.6

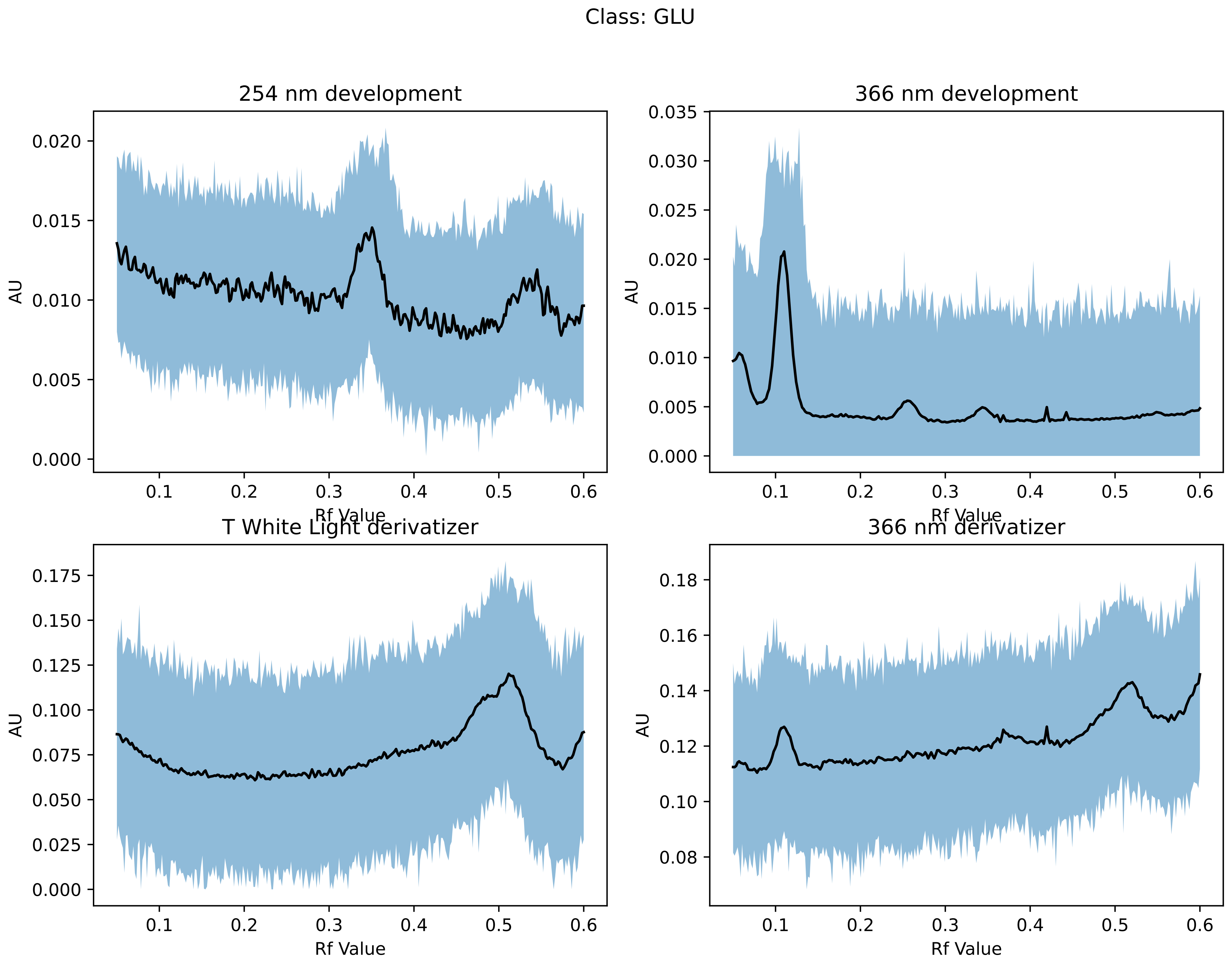
Class: GLU
254 nm development
366 nm development
T White Light derivatizer
366 nm derivatizer
AU
Rf Value
0.000
0.005
0.010
0.015
0.020
0.025
0.030
0.035
0.050
0.075
0.100
0.125
0.150
0.175
0.08
0.10
0.12
0.14
0.16
0.18
0.1
0.2
0.3
0.4
0.5
0.6

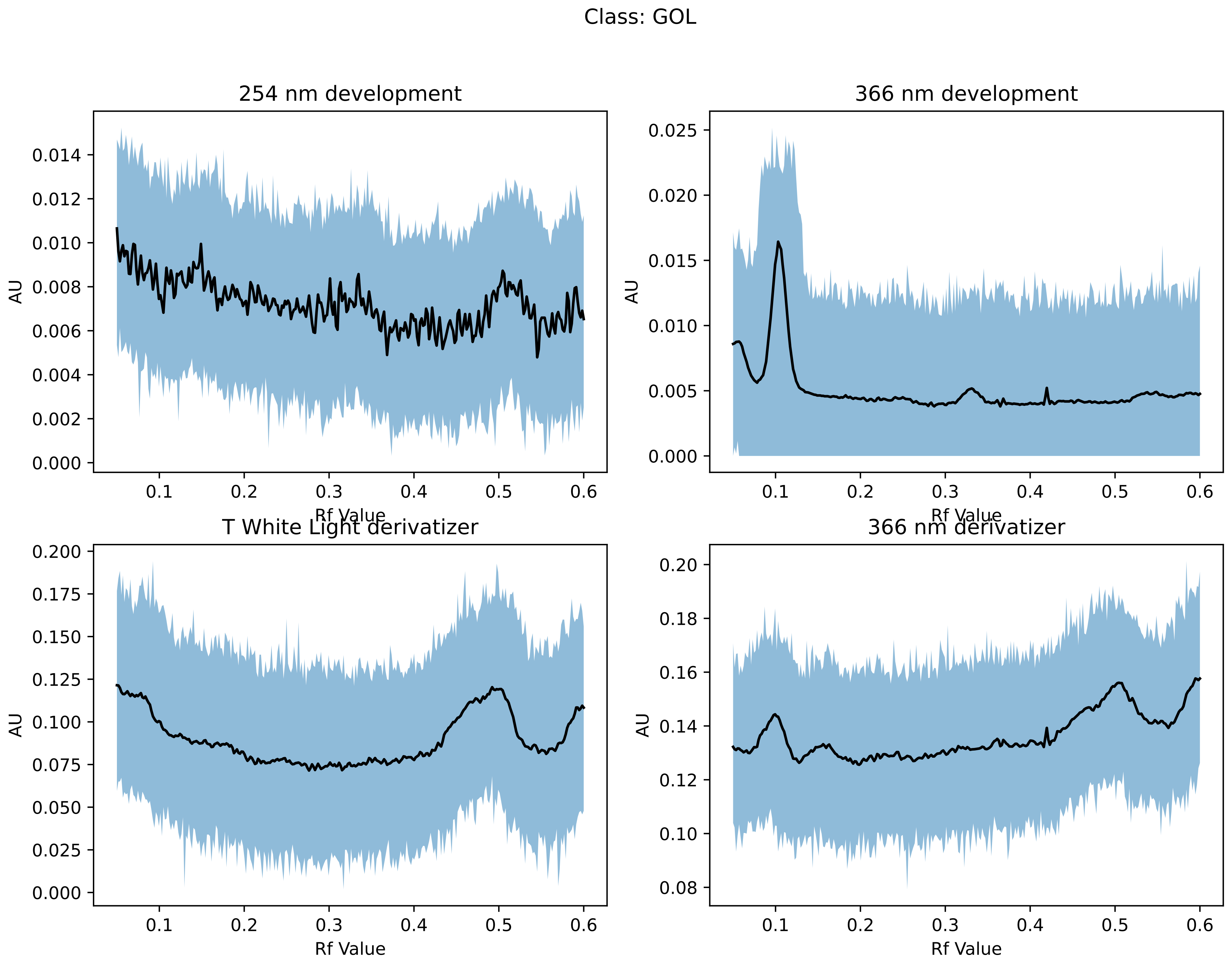

Class: GOL
254 nm development
366 nm development
T White Light derivatizer
366 nm derivatizer
AU
Rf Value
0.000
0.002
0.004
0.006
0.008
0.010
0.012
0.014
0.005
0.015
0.020
0.025
0.025
0.050
0.075
0.100
0.125
0.150
0.175
0.200
0.08
0.10
0.12
0.14
0.16
0.18
0.20
0.1
0.2
0.3
0.4
0.5
0.6

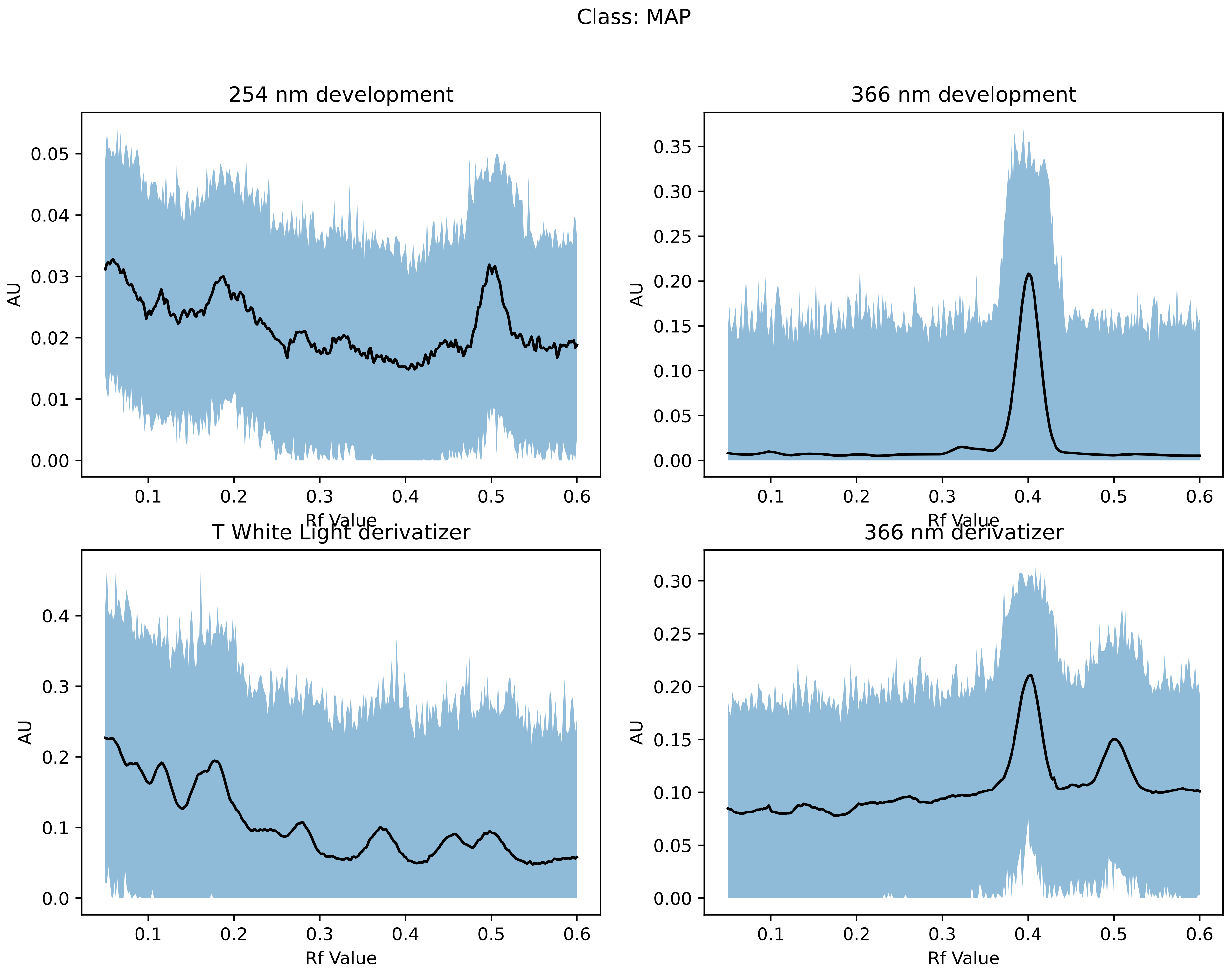
Class: MAP
254 nm development
366 nm development
T White Light derivatizer
366 nm derivatizer
AU
Rf Value
0.05
0.04
0.03
0.02
0.01
0.00
0.35
0.30
0.25
0.20
0.15
0.10
0.4
0.3
0.2
0.1
0.0
0.1
0.2
0.3
0.4
0.5
0.6

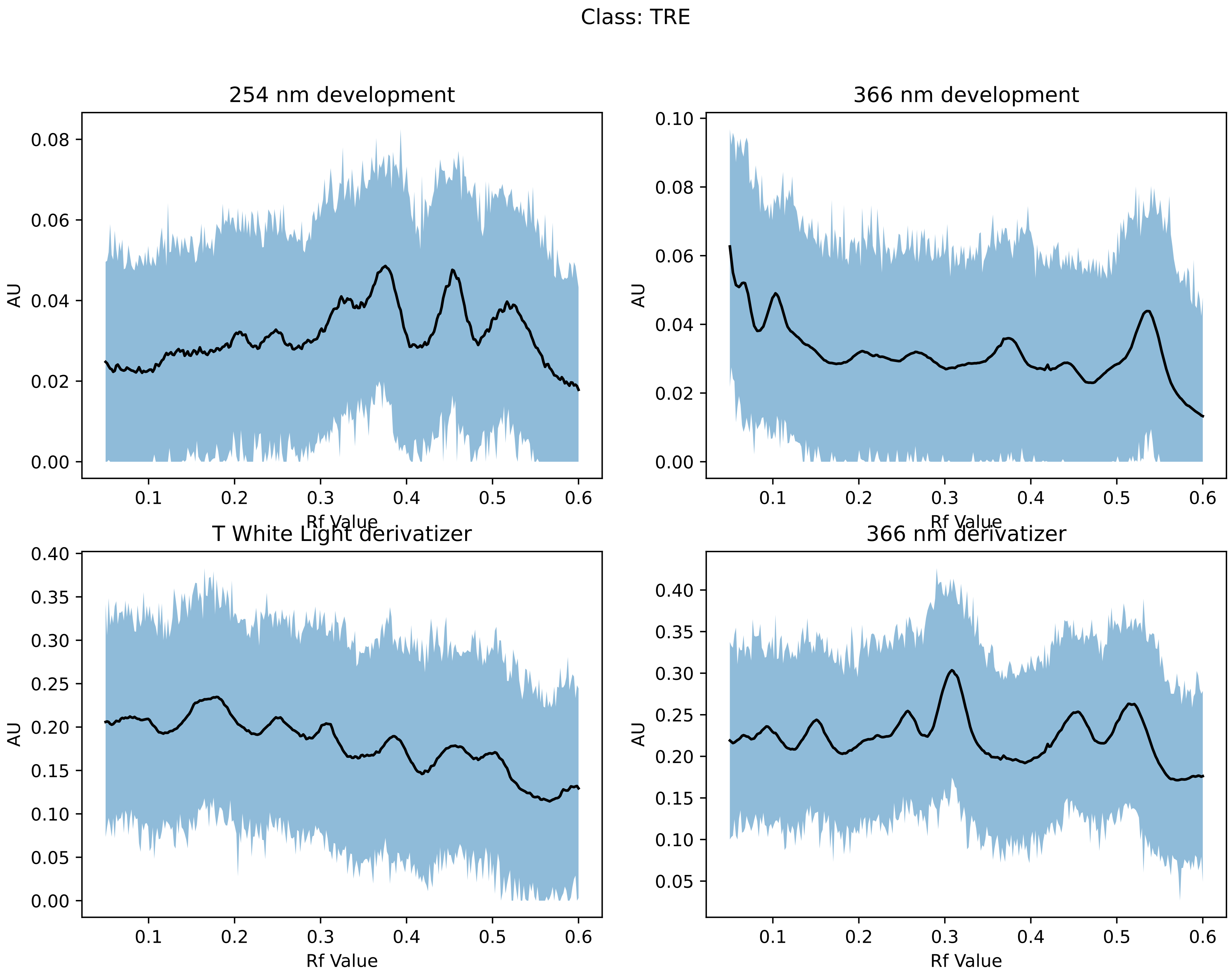
Class: TRE
254 nm development
366 nm development
T White Light derivatizer
366 nm derivatizer
AU
Rf Value
0.00
0.02
0.04
0.06
0.08
0.10
0.05
0.15
0.20
0.25
0.30
0.35
0.40
0.1
0.2
0.3
0.4
0.5
0.6

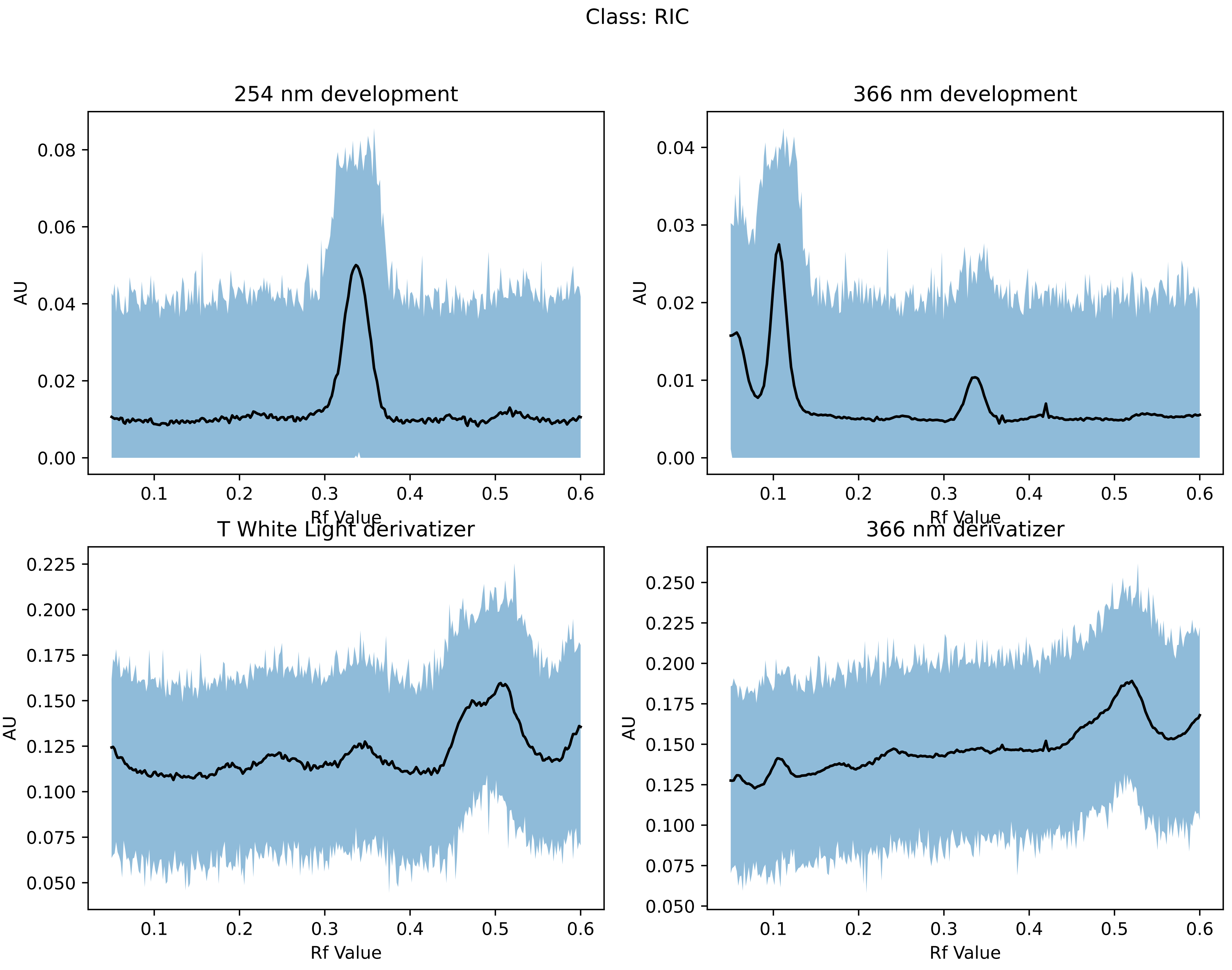

Class: RIC
254 nm development
AU
0.00
0.02
0.04
0.06
0.08
0.1
0.2
0.3
0.4
0.5
0.6
Rf Value
366 nm development
AU
0.00
0.01
0.02
0.03
0.04
Rf Value
T White Light derivatizer
AU
0.050
0.075
0.100
0.125
0.150
0.175
0.200
0.225
Rf Value
366 nm derivatizer
AU
0.050
0.075
0.100
0.125
0.150
0.175
0.200
0.225
0.250
Rf Value

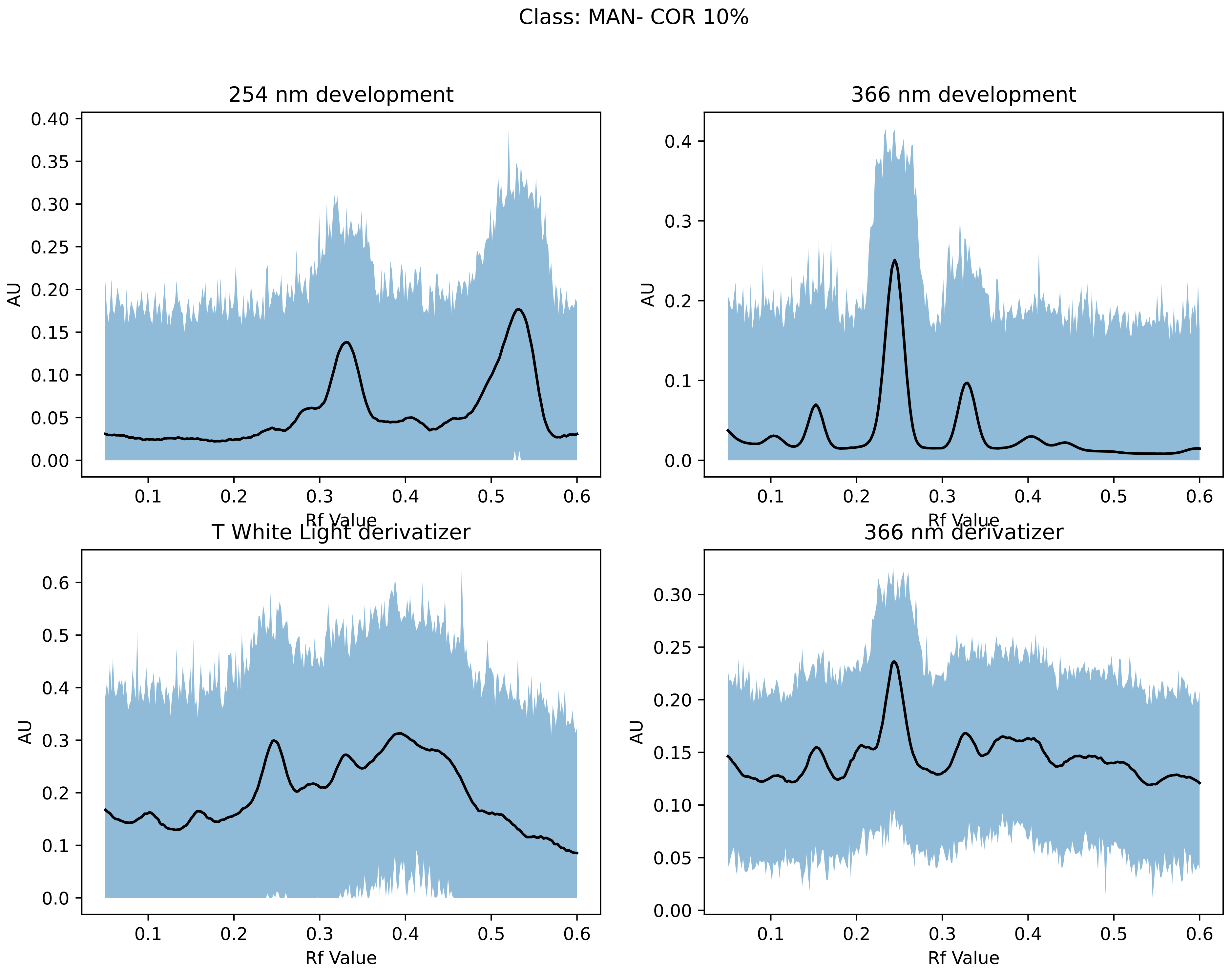

Class: MAN- COR 10%
254 nm development
366 nm development
T White Light derivatizer
366 nm derivatizer
AU
Rf Value
0.00
0.05
0.10
0.15
0.20
0.25
0.30
0.35
0.40
0.0
0.1
0.2
0.3
0.4
0.5
0.6

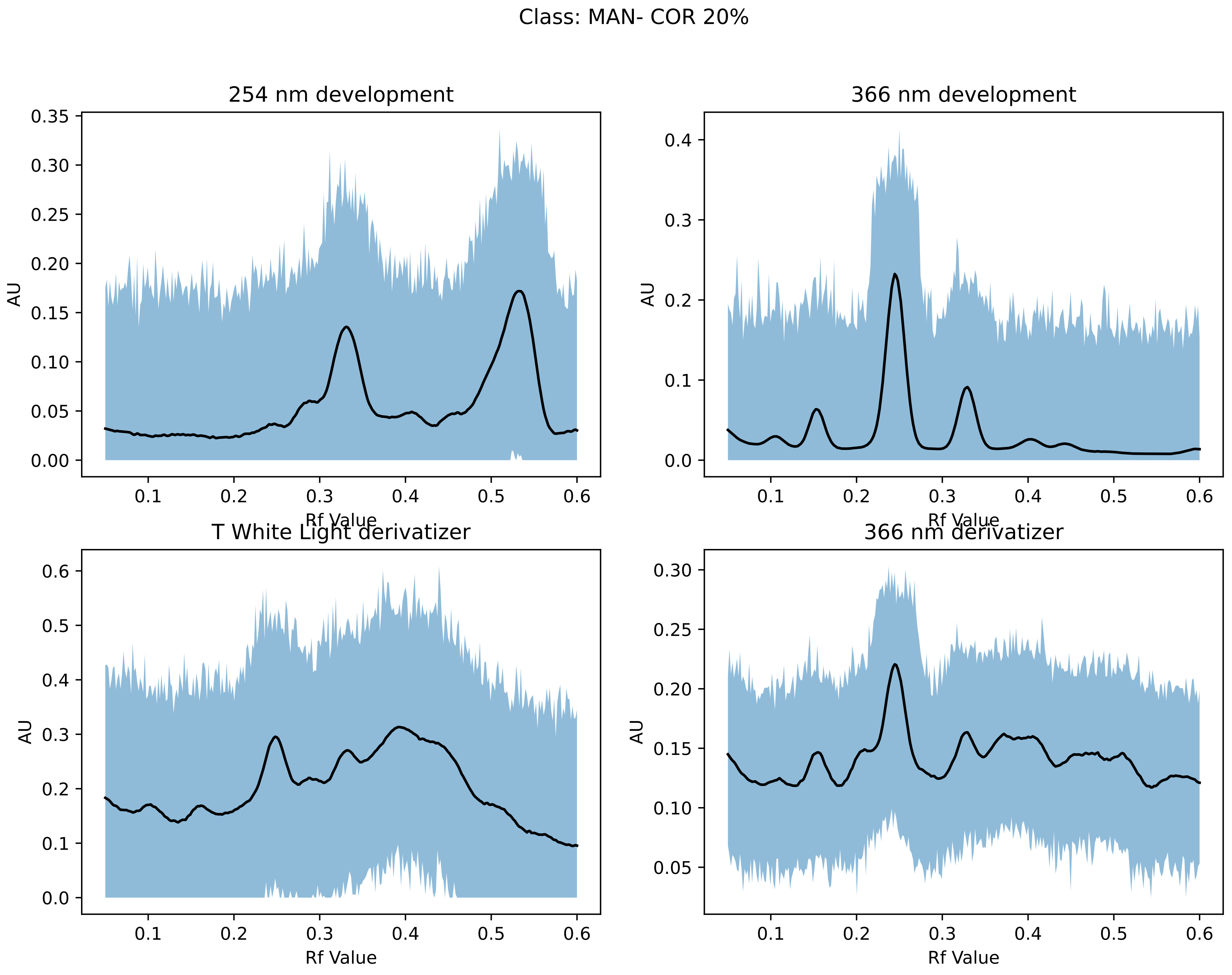

Class: MAN- COR 20%
254 nm development
366 nm development
T White Light derivatizer
366 nm derivatizer
AU
Rf Value
0.35
0.30
0.25
0.20
0.15
0.10
0.05
0.00
0.4
0.3
0.2
0.1
0.0
0.6
0.5
0.1
0.2
0.3
0.4
0.5
0.6

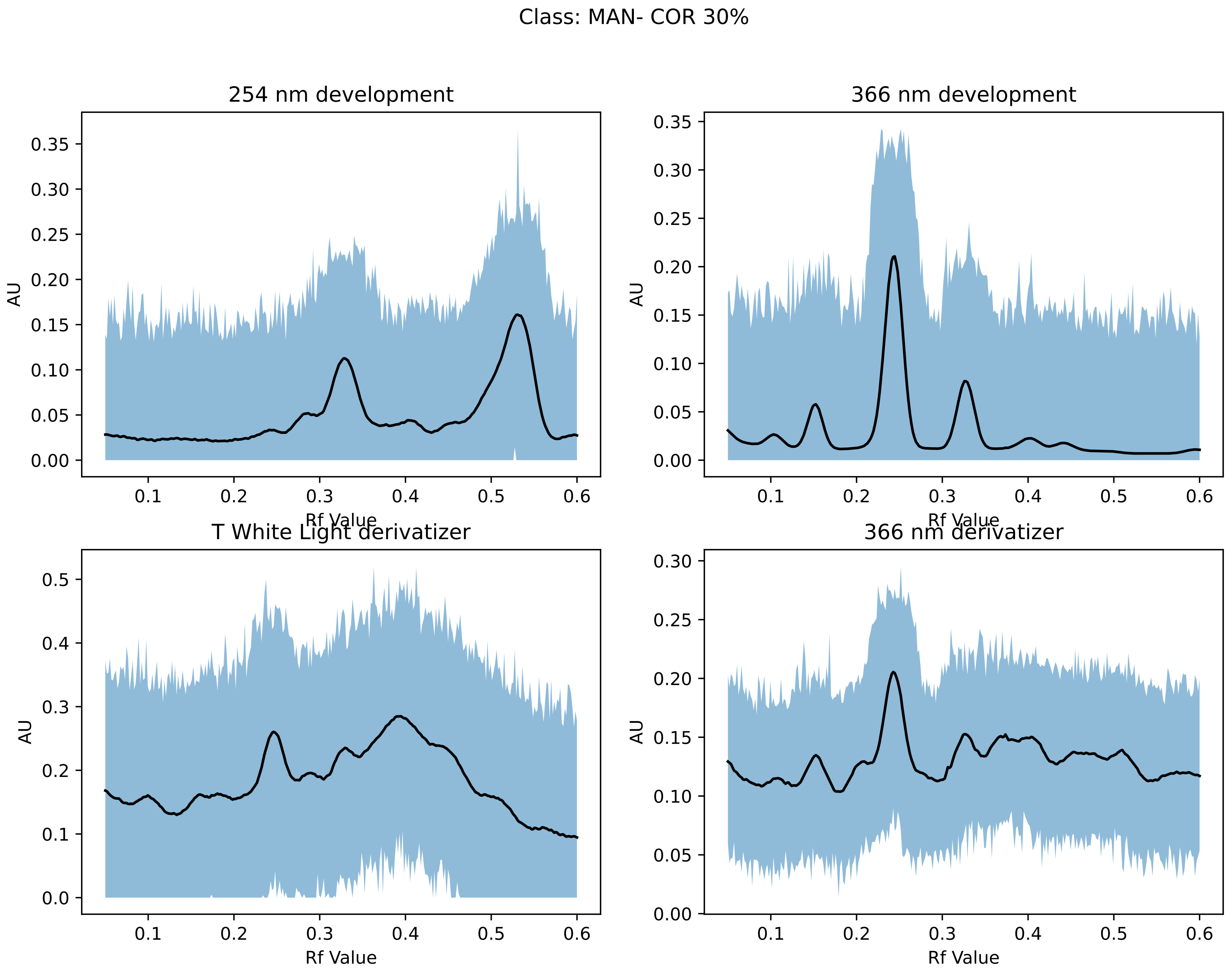
Class: MAN- COR 30%
254 nm development
366 nm development
T White Light derivatizer
366 nm derivatizer
AU
Rf Value
0.00
0.05
0.10
0.15
0.20
0.25
0.30
0.35
0.0
0.1
0.2
0.3
0.4
0.5
0.6

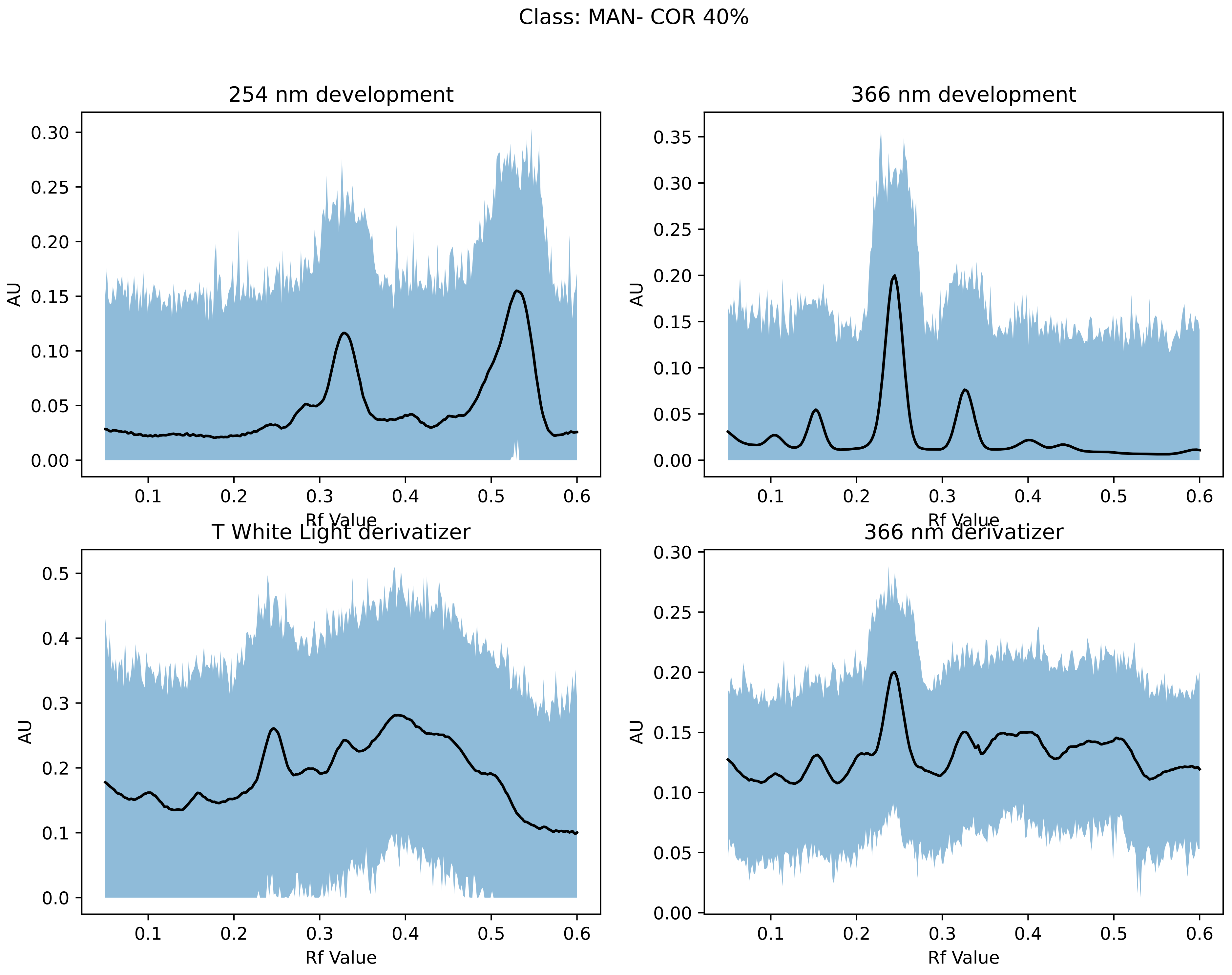

Class: MAN- COR 40%
254 nm development
366 nm development
T White Light derivatizer
366 nm derivatizer
AU
Rf Value

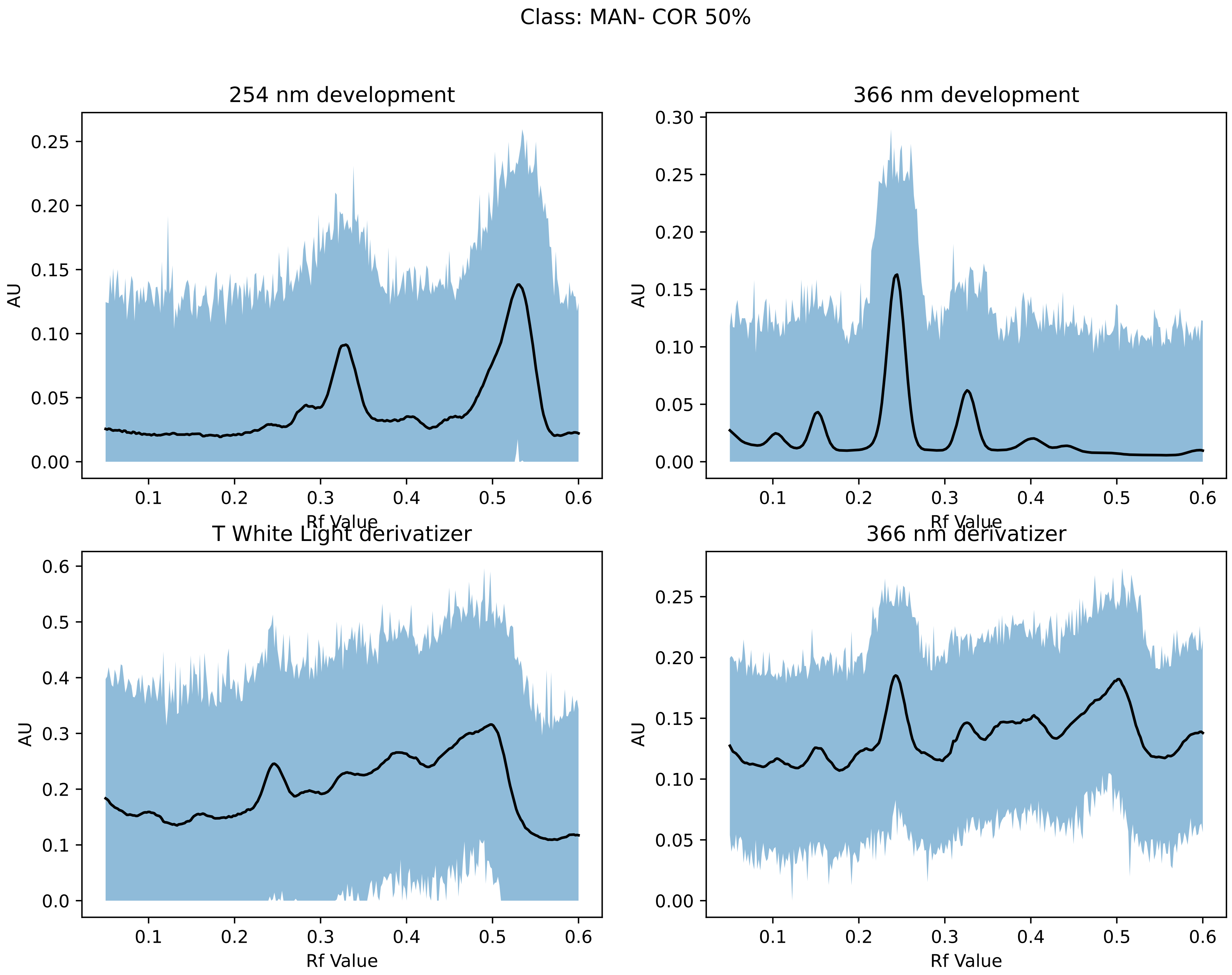
Class: MAN- COR 50%
254 nm development
366 nm development
T White Light derivatizer
366 nm derivatizer
AU
Rf Value
0.00
0.05
0.10
0.15
0.20
0.25
0.30
0.0
0.1
0.2
0.3
0.4
0.5
0.6

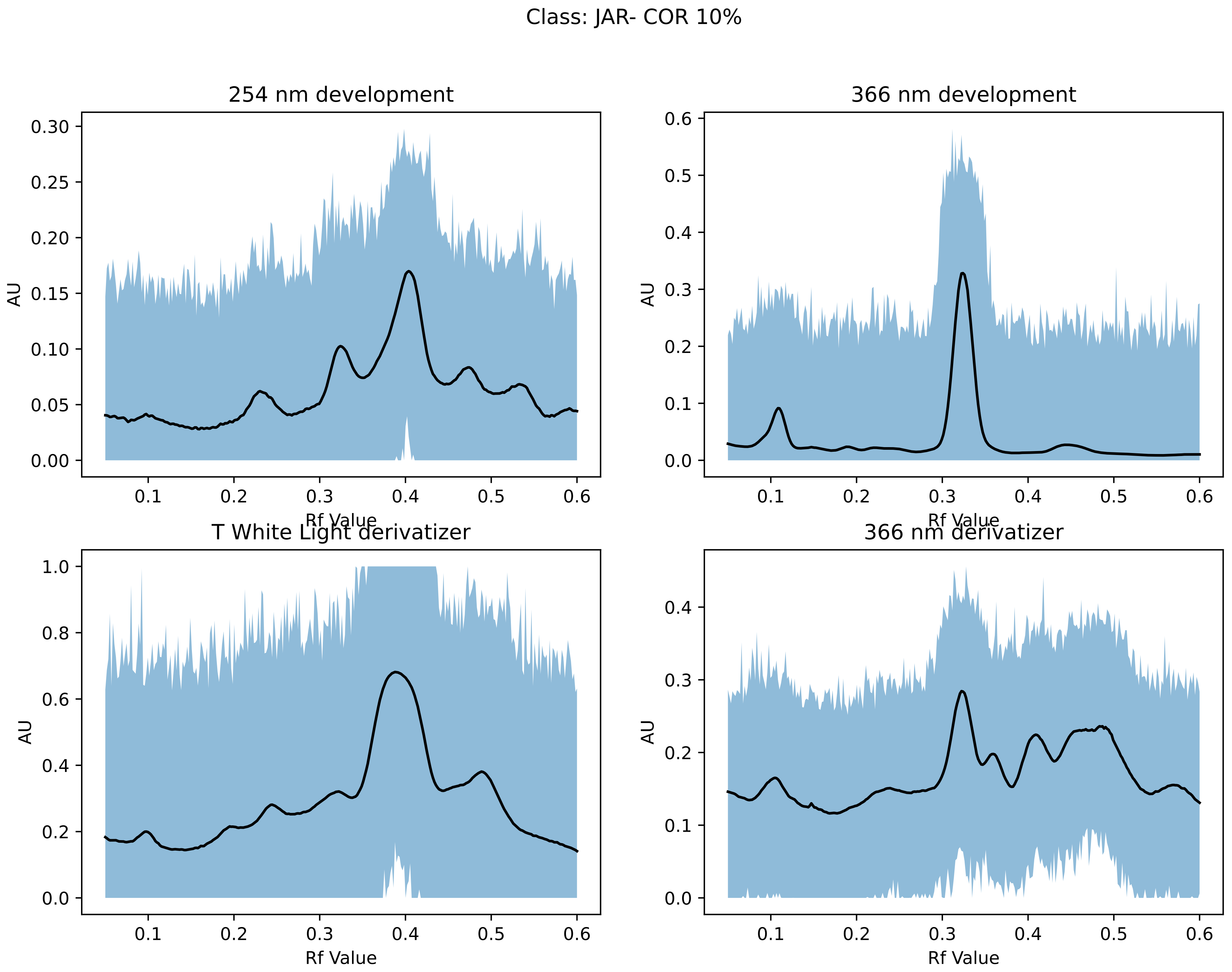
Class: JAR- COR 10%
254 nm development
366 nm development
T White Light derivatizer
366 nm derivatizer
AU
Rf Value
0.00
0.05
0.10
0.15
0.20
0.25
0.30
0.0
0.1
0.2
0.3
0.4
0.5
0.6
0.8
1.0

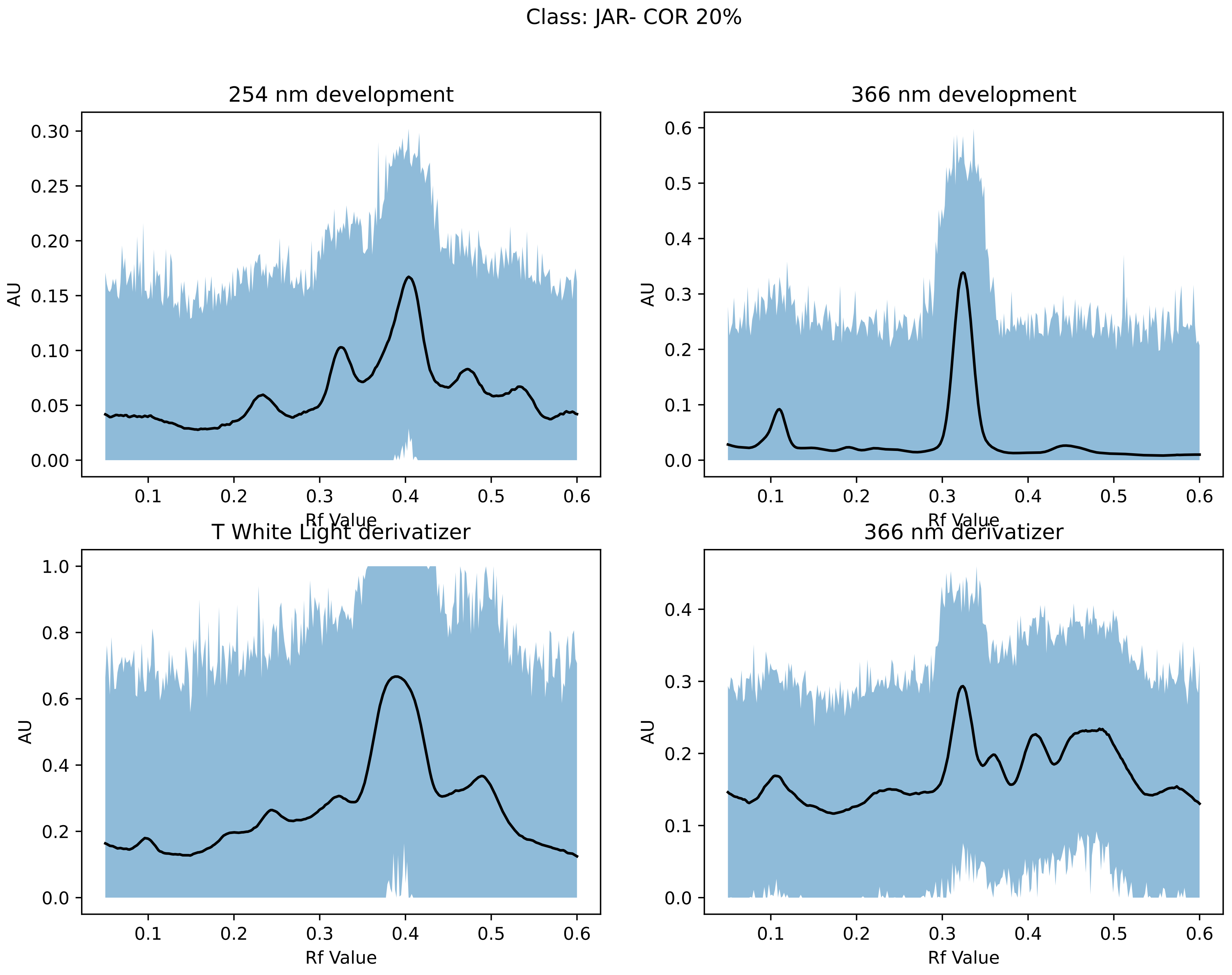

Class: JAR- COR 20%
254 nm development
366 nm development
T White Light derivatizer
366 nm derivatizer
AU
Rf Value
0.00
0.05
0.10
0.15
0.20
0.25
0.30
0.0
0.1
0.2
0.3
0.4
0.5
0.6
0.8
1.0

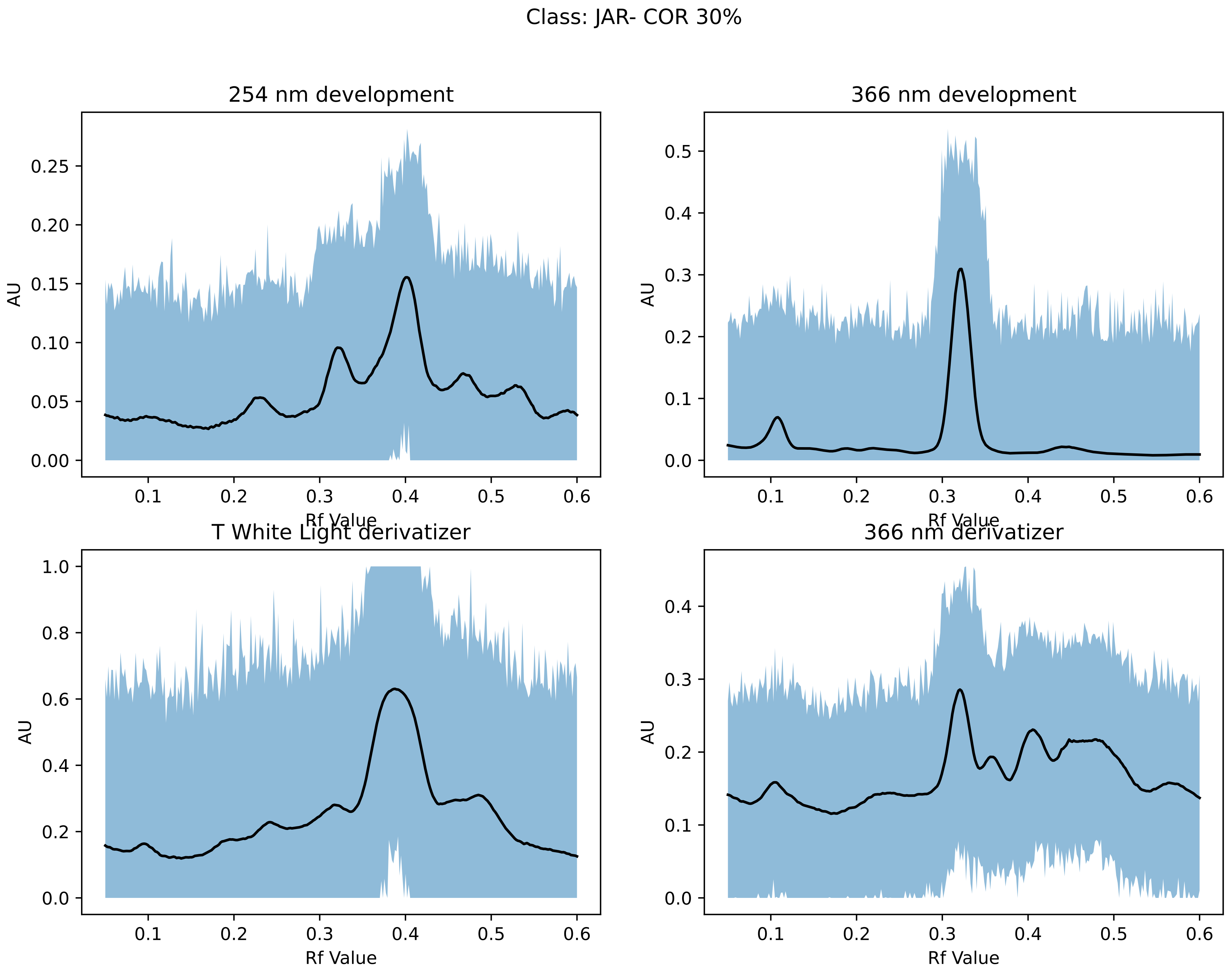
Class: JAR- COR 30%
254 nm development
366 nm development
T White Light derivatizer
366 nm derivatizer
AU
Rf Value

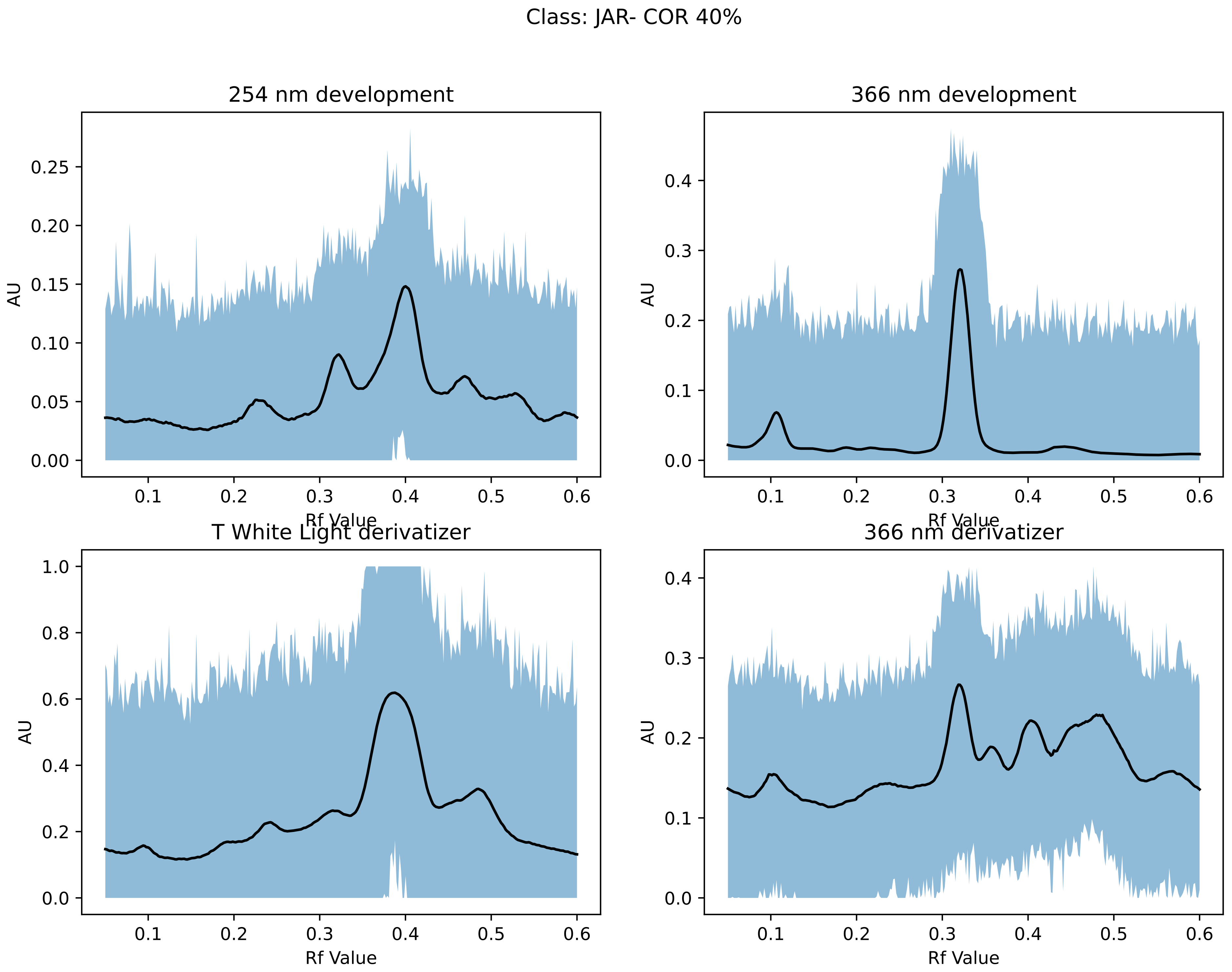
Class: JAR- COR 40%
254 nm development
366 nm development
T White Light derivatizer
366 nm derivatizer
AU
Rf Value
0.00
0.05
0.10
0.15
0.20
0.25
0.0
0.1
0.2
0.3
0.4
0.6
0.8
1.0
0.5

Class: JAR- COR 50%

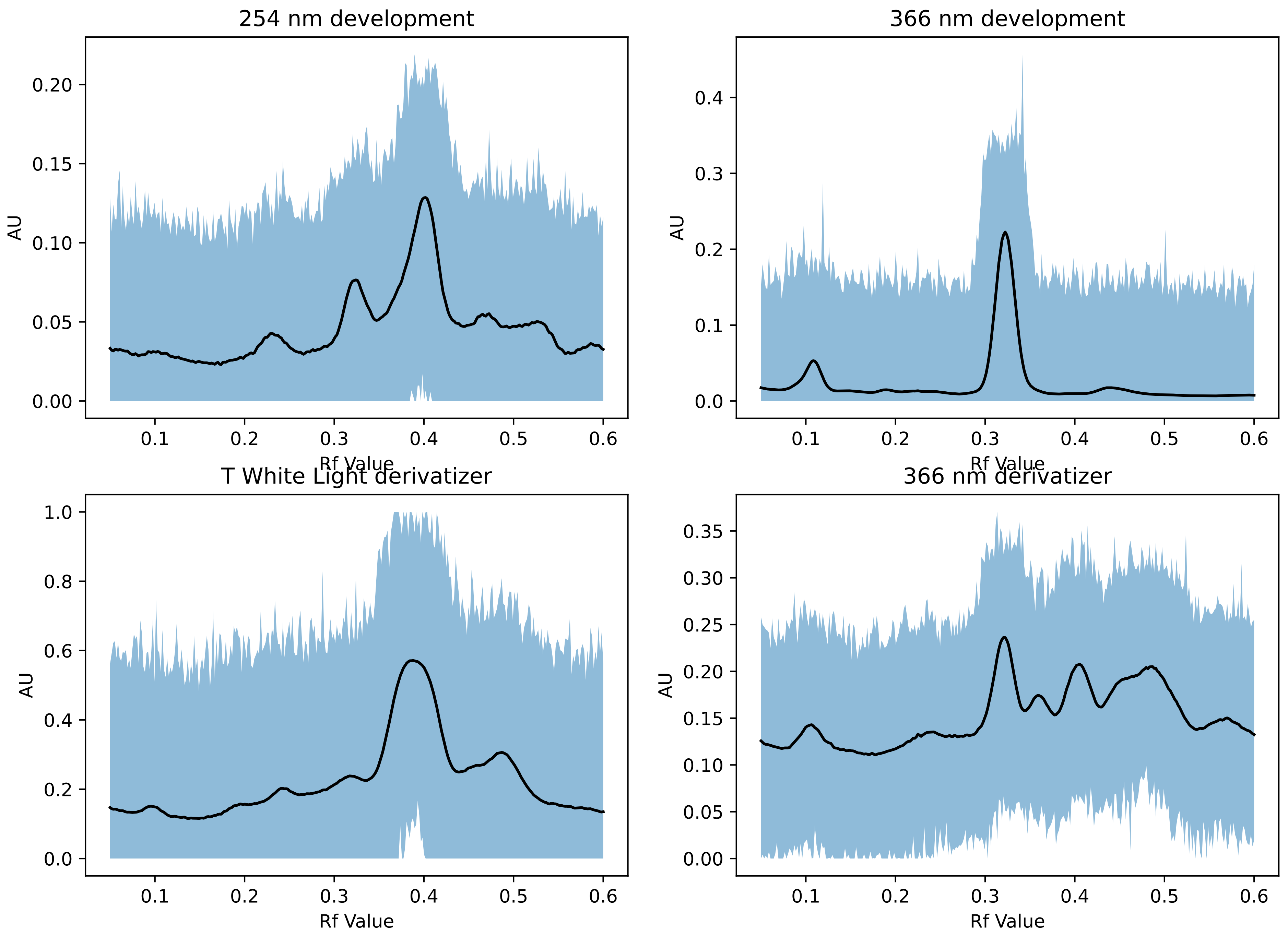

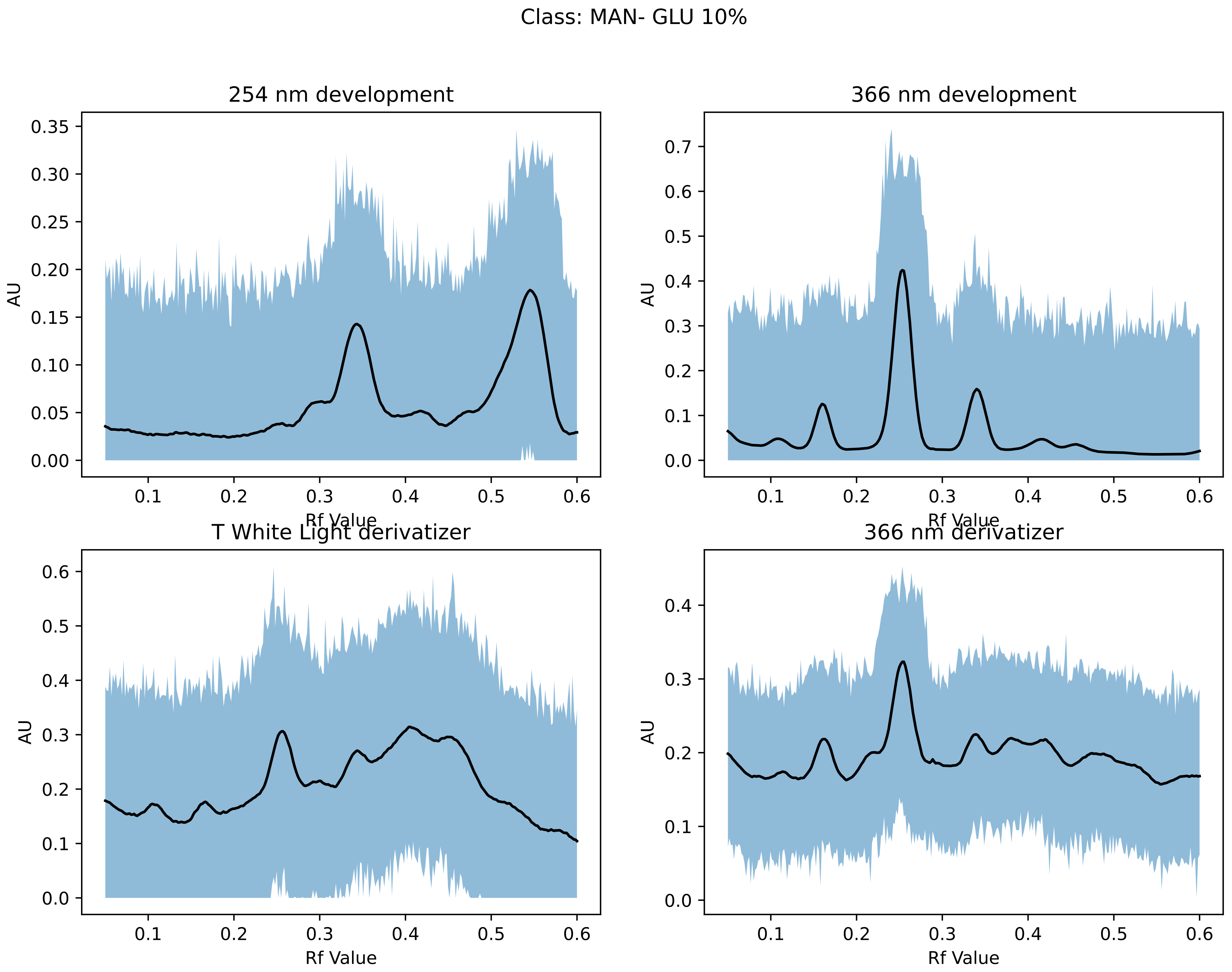

Class: MAN- GLU 10%
254 nm development
366 nm development
T White Light derivatizer
366 nm derivatizer
AU
Rf Value

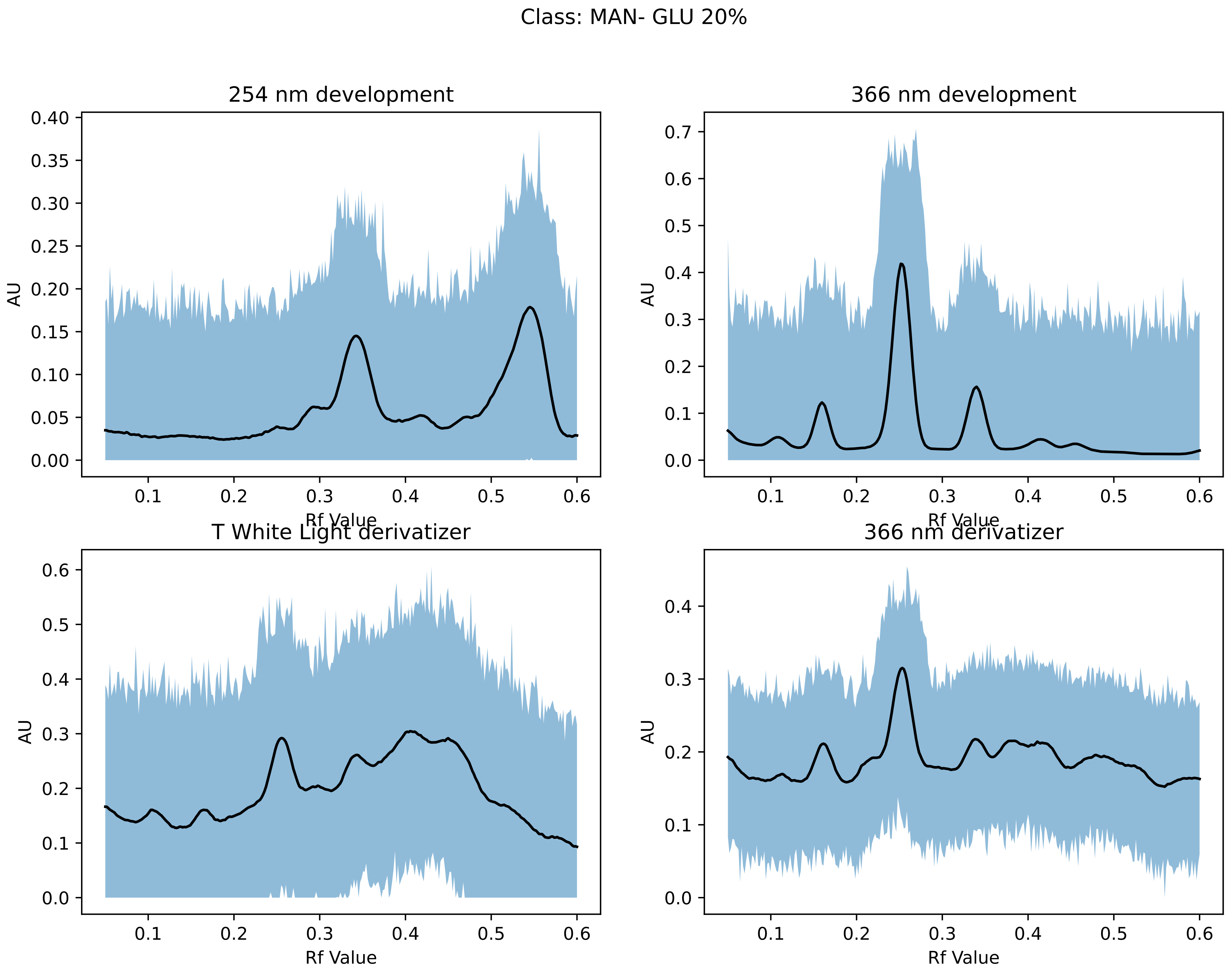
Class: MAN- GLU 20%
254 nm development
366 nm development
T White Light derivatizer
366 nm derivatizer
AU
Rf Value
0.40
0.35
0.30
0.25
0.20
0.15
0.10
0.05
0.00
0.7
0.6
0.5
0.4
0.3
0.2
0.1
0.0
0.1
0.2
0.3
0.4
0.5
0.6

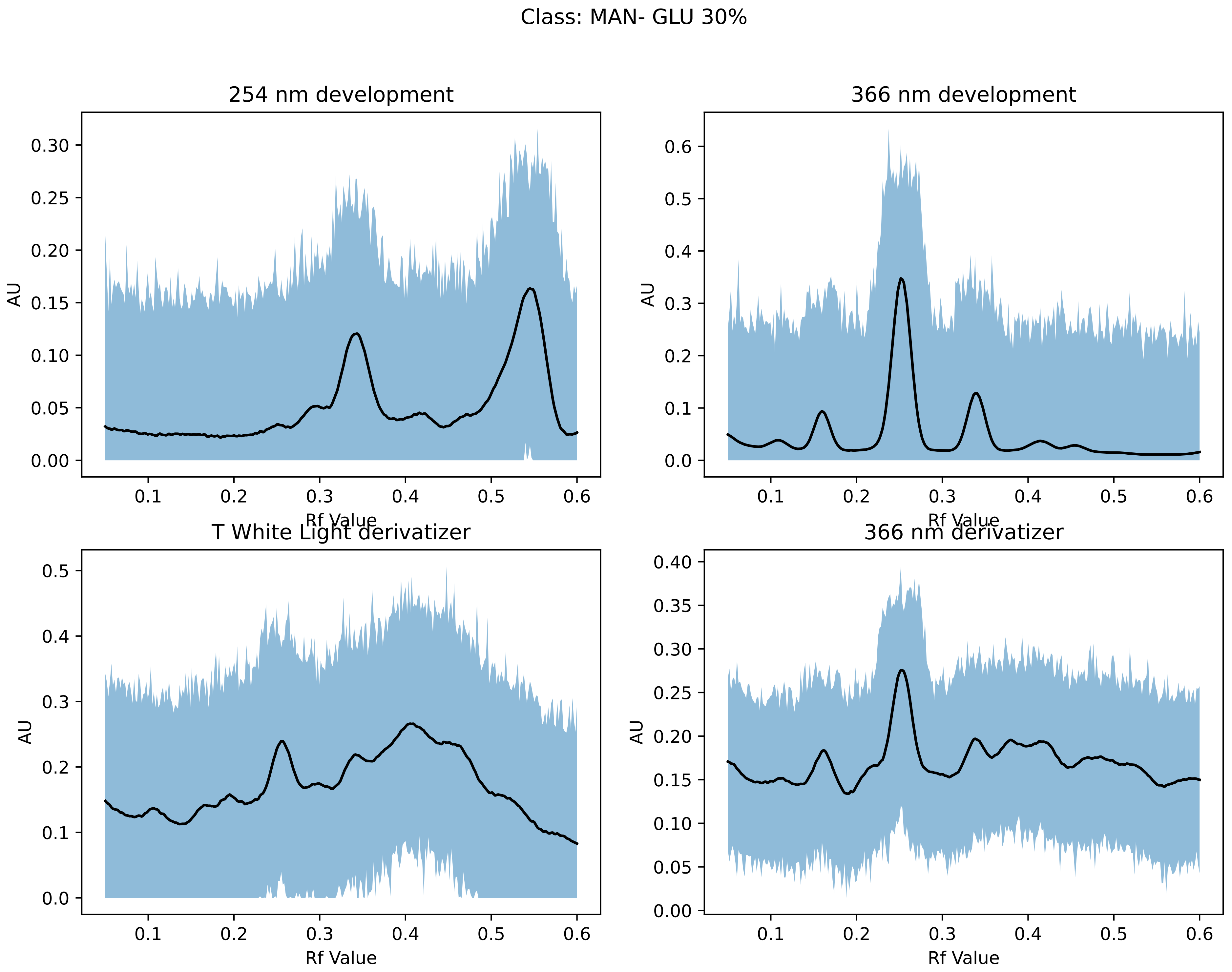

Class: MAN- GLU 30%
254 nm development
366 nm development
T White Light derivatizer
366 nm derivatizer
AU
Rf Value
0.1
0.2
0.3
0.4
0.5
0.6
0.00
0.05
0.10
0.15
0.20
0.25
0.30
0.35
0.40

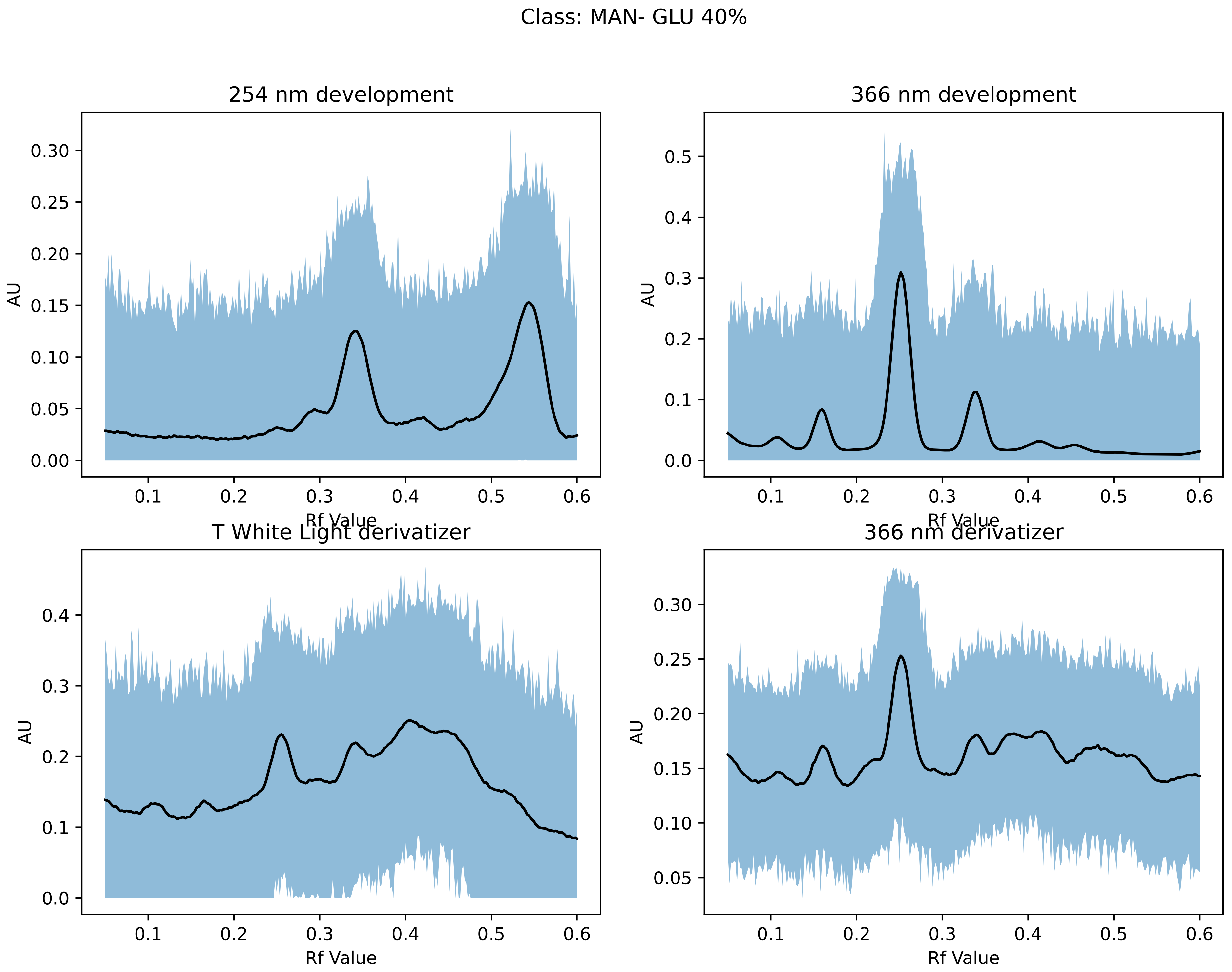

Class: MAN- GLU 40%
254 nm development
366 nm development
T White Light derivatizer
366 nm derivatizer
AU
Rf Value
0.00
0.05
0.10
0.15
0.20
0.25
0.30
0.0
0.1
0.2
0.3
0.4
0.5
0.6

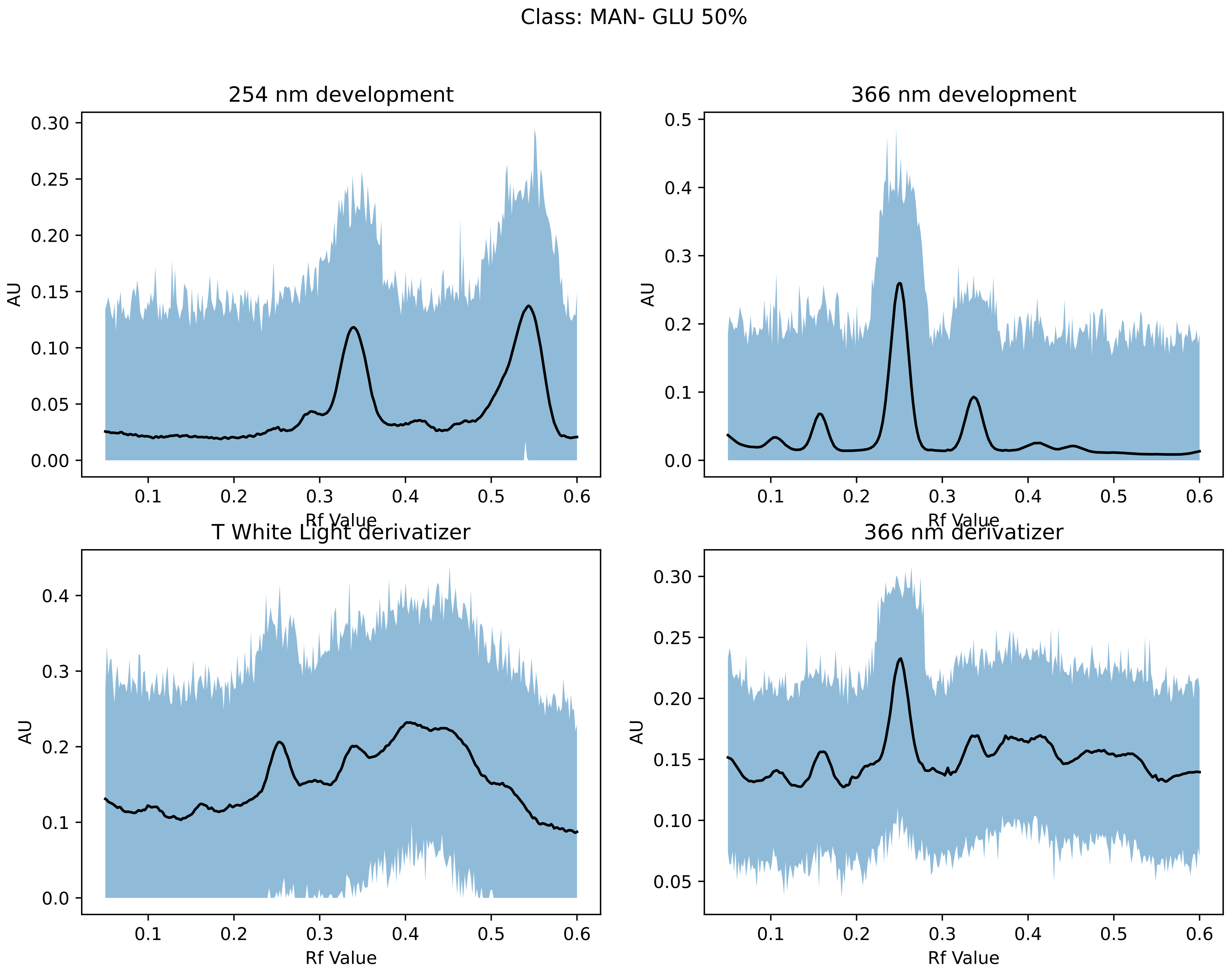

Class: MAN- GLU 50%
254 nm development
366 nm development
T White Light derivatizer
366 nm derivatizer
AU
Rf Value
0.00
0.05
0.10
0.15
0.20
0.25
0.30
0.0
0.1
0.2
0.3
0.4
0.5
0.6

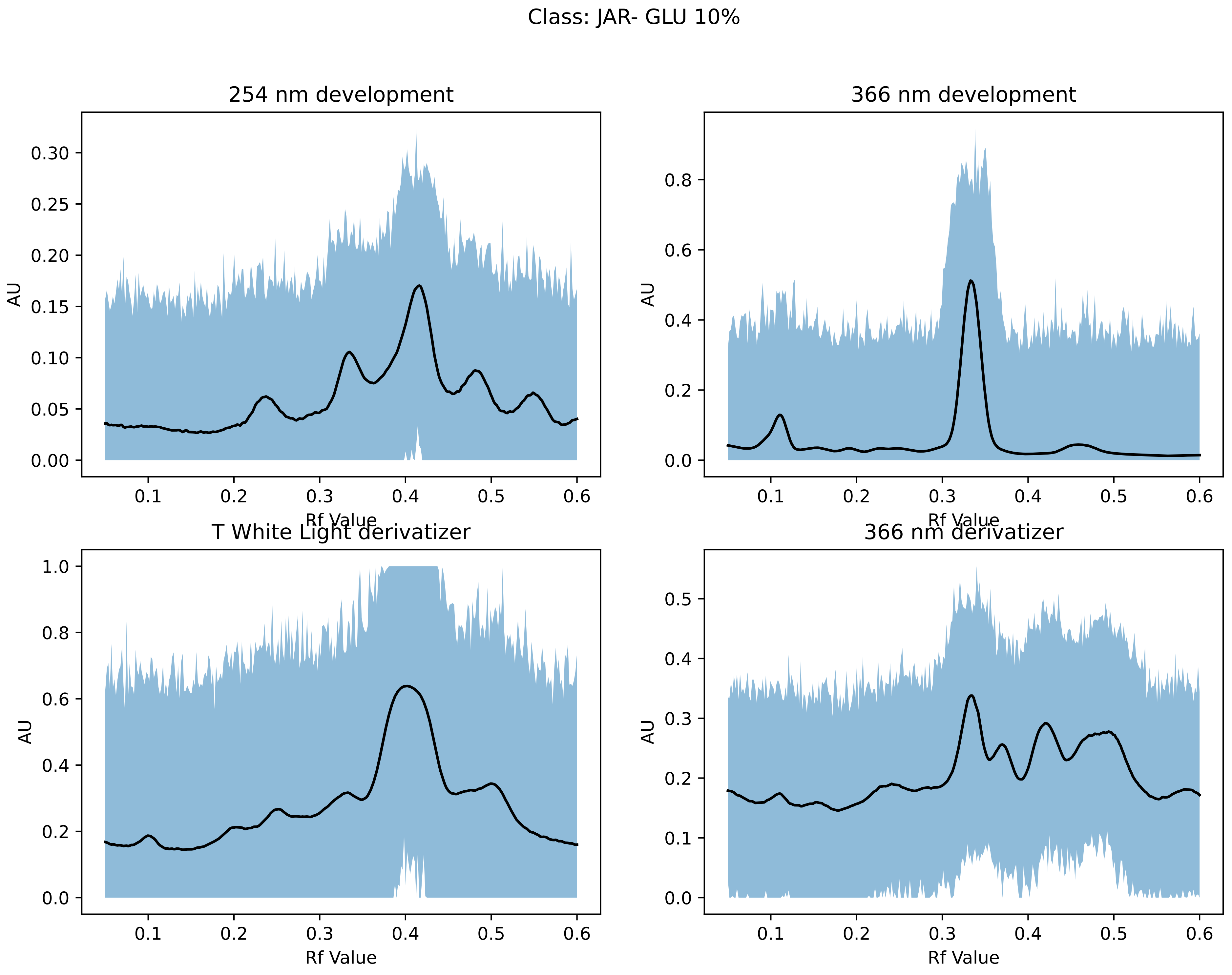
Class: JAR- GLU 10%
254 nm development
366 nm development
T White Light derivatizer
366 nm derivatizer
AU
Rf Value
0.00
0.05
0.10
0.15
0.20
0.25
0.30
0.0
0.2
0.4
0.6
0.8
1.0
0.1
0.3
0.5
0.1
0.2
0.3
0.4
0.5
0.6

Class: JAR- GLU 20%

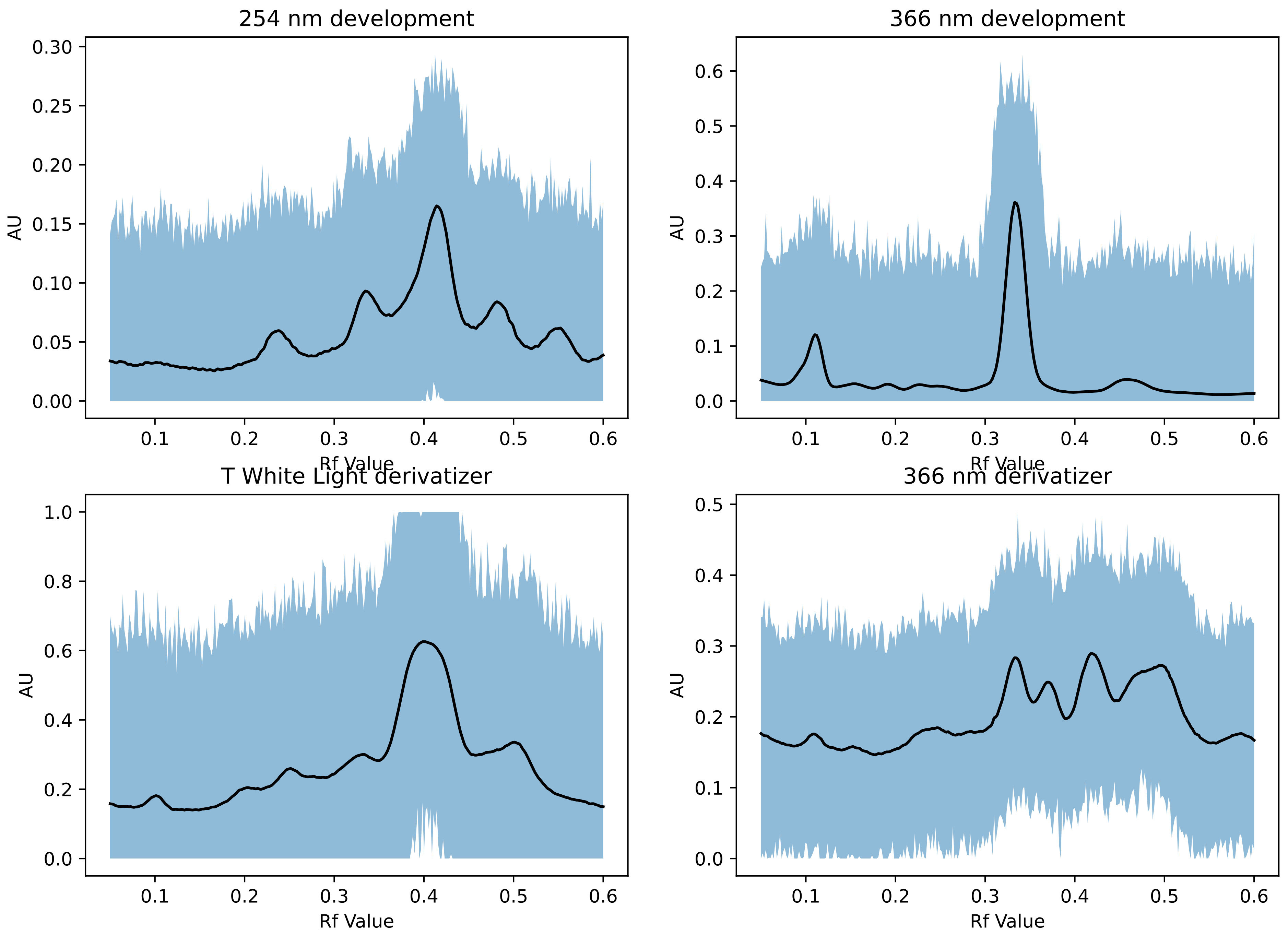

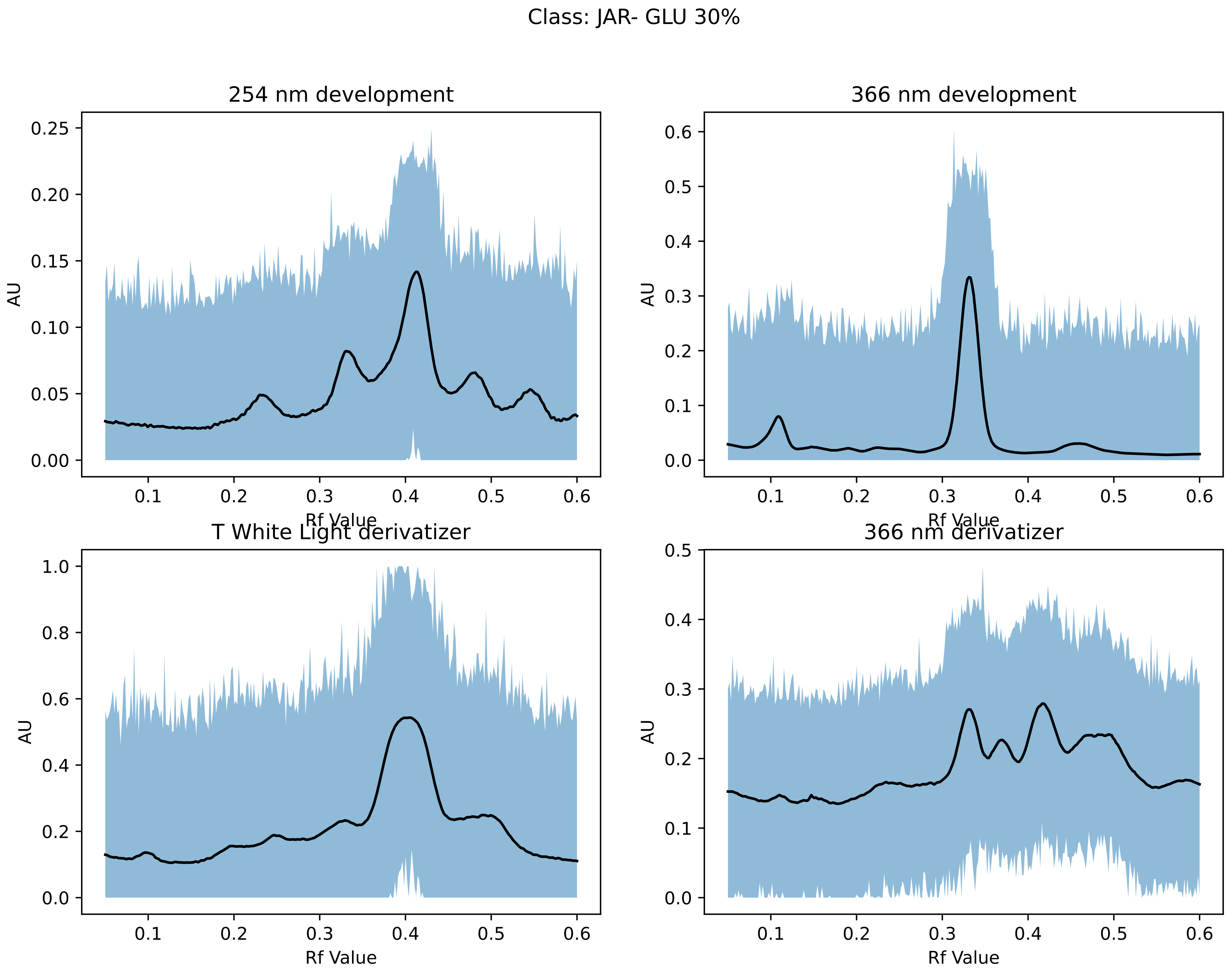
Class: JAR- GLU 30%
254 nm development
366 nm development
T White Light derivatizer
366 nm derivatizer
AU
Rf Value
0.00
0.05
0.10
0.15
0.20
0.25
0.0
0.1
0.2
0.3
0.4
0.5
0.6
0.8
1.0

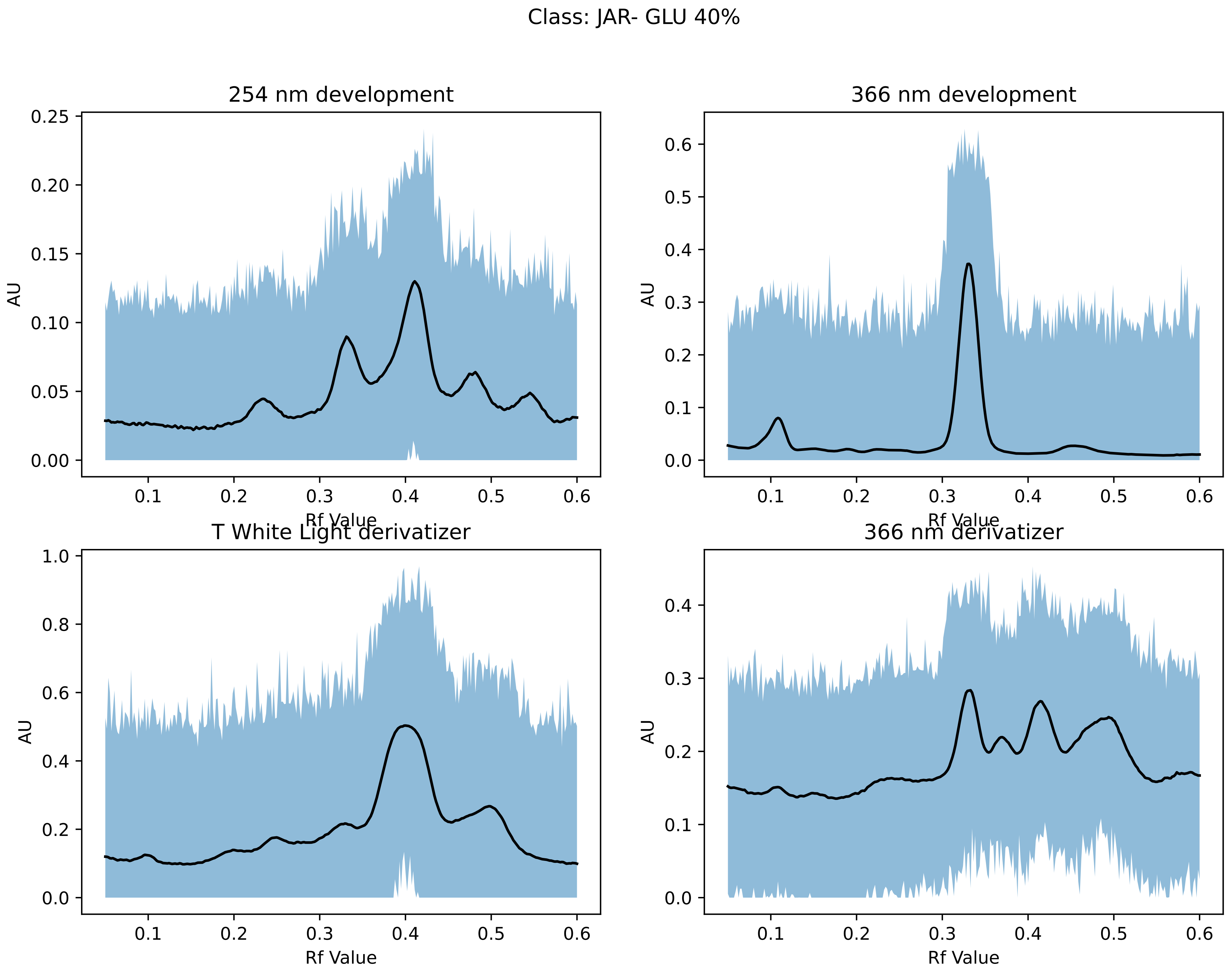
Class: JAR- GLU 40%
254 nm development
366 nm development
T White Light derivatizer
366 nm derivatizer
AU
Rf Value
0.1
0.2
0.3
0.4
0.5
0.6
0.00
0.05
0.10
0.15
0.20
0.25
0.0
0.1
0.2
0.3
0.4
0.5
0.6
0.8
1.0

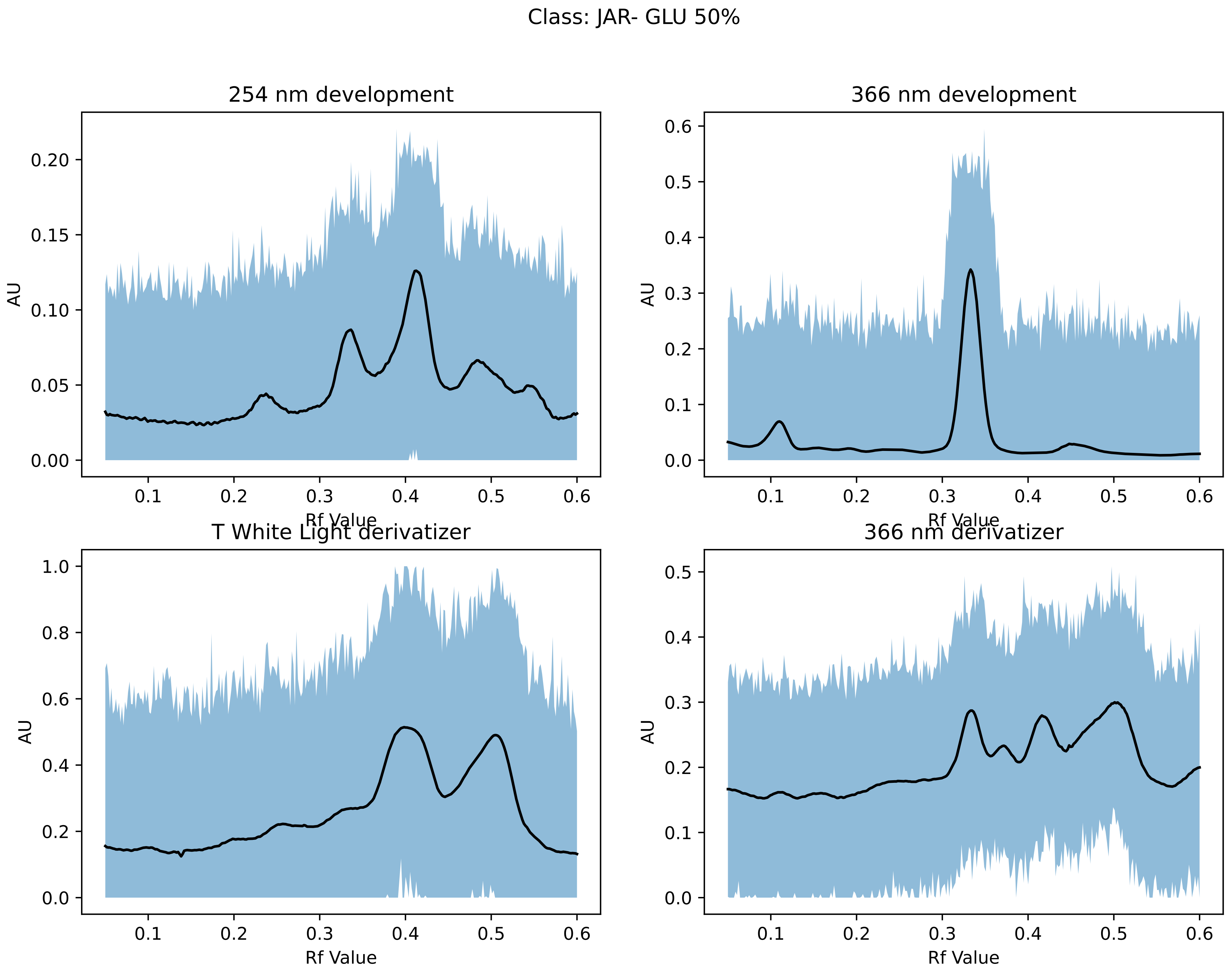

Class: JAR- GLU 50%
254 nm development
366 nm development
T White Light derivatizer
366 nm derivatizer
AU
Rf Value
0.00
0.05
0.10
0.15
0.20
0.0
0.1
0.2
0.3
0.4
0.5
0.6
0.8
1.0

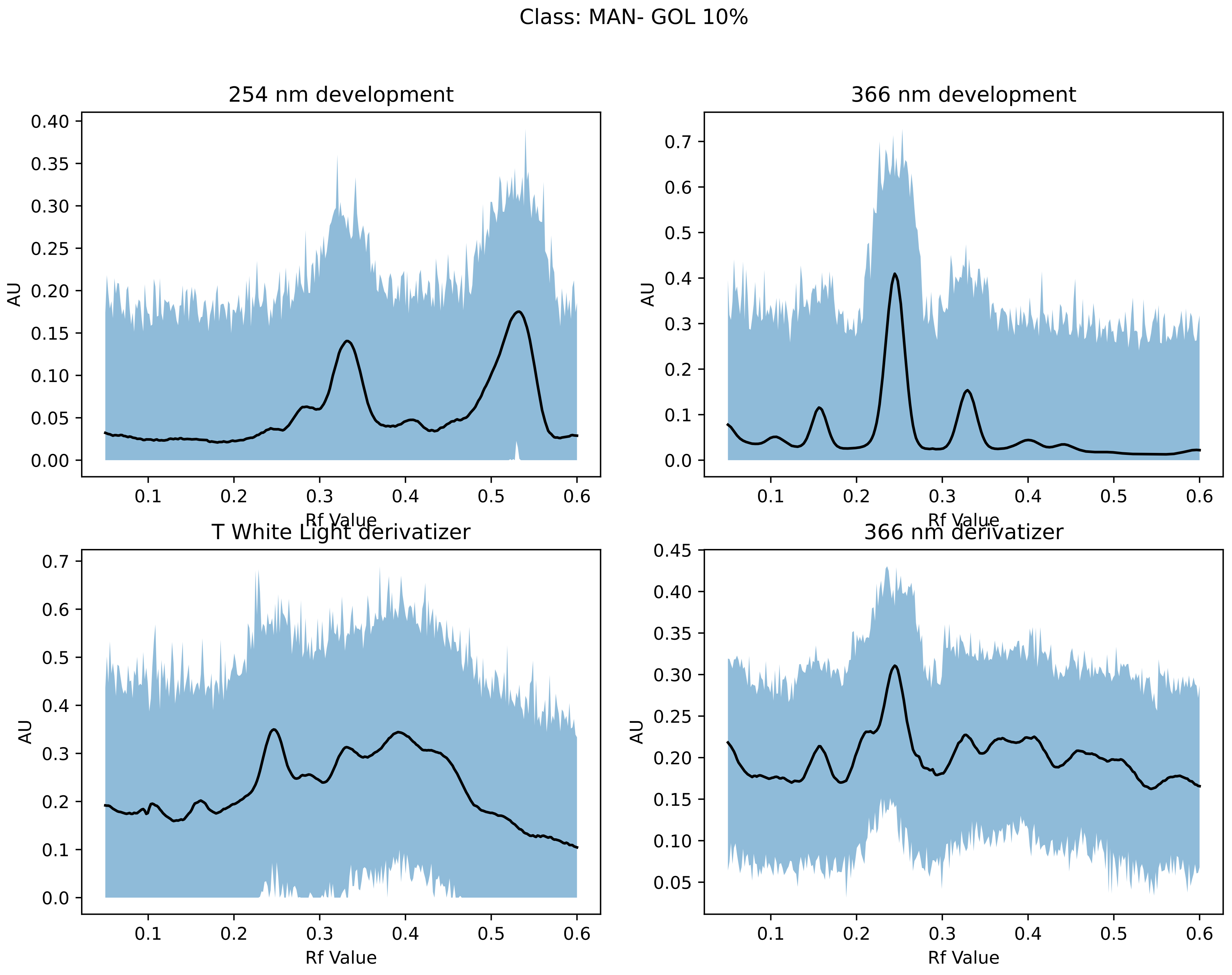
Class: MAN- GOL 10%
254 nm development
366 nm development
T White Light derivatizer
366 nm derivatizer
AU
Rf Value
0.40
0.35
0.30
0.25
0.20
0.15
0.10
0.05
0.00
0.7
0.6
0.5
0.4
0.3
0.2
0.1
0.0
0.45
0.1
0.2
0.3
0.4
0.5
0.6

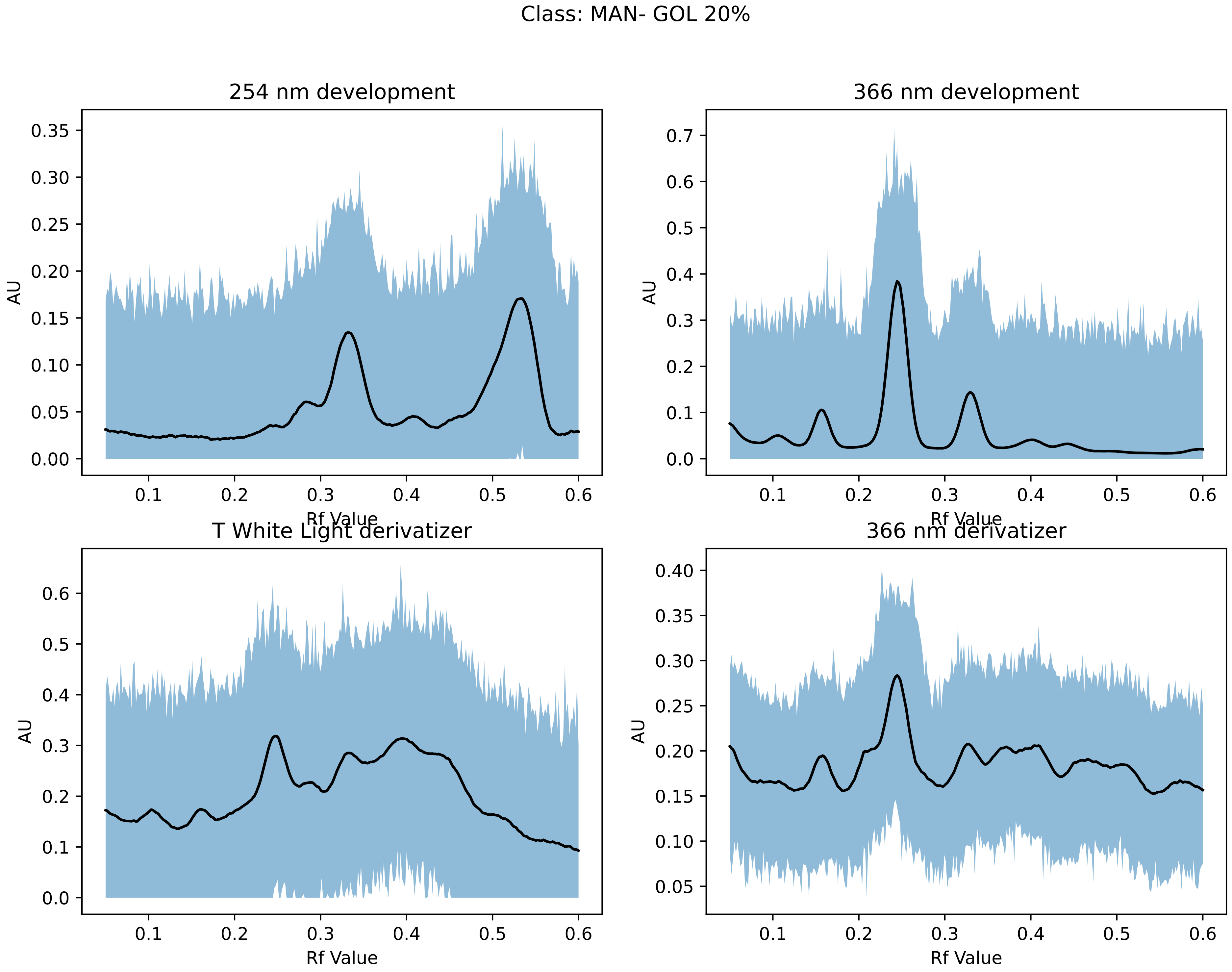

Class: MAN- GOL 20%
254 nm development
366 nm development
T White Light derivatizer
366 nm derivatizer
AU
Rf Value
0.00
0.05
0.10
0.15
0.20
0.25
0.30
0.35
0.0
0.1
0.2
0.3
0.4
0.5
0.6
0.7
0.40
0.1
0.2
0.3
0.4
0.5
0.6

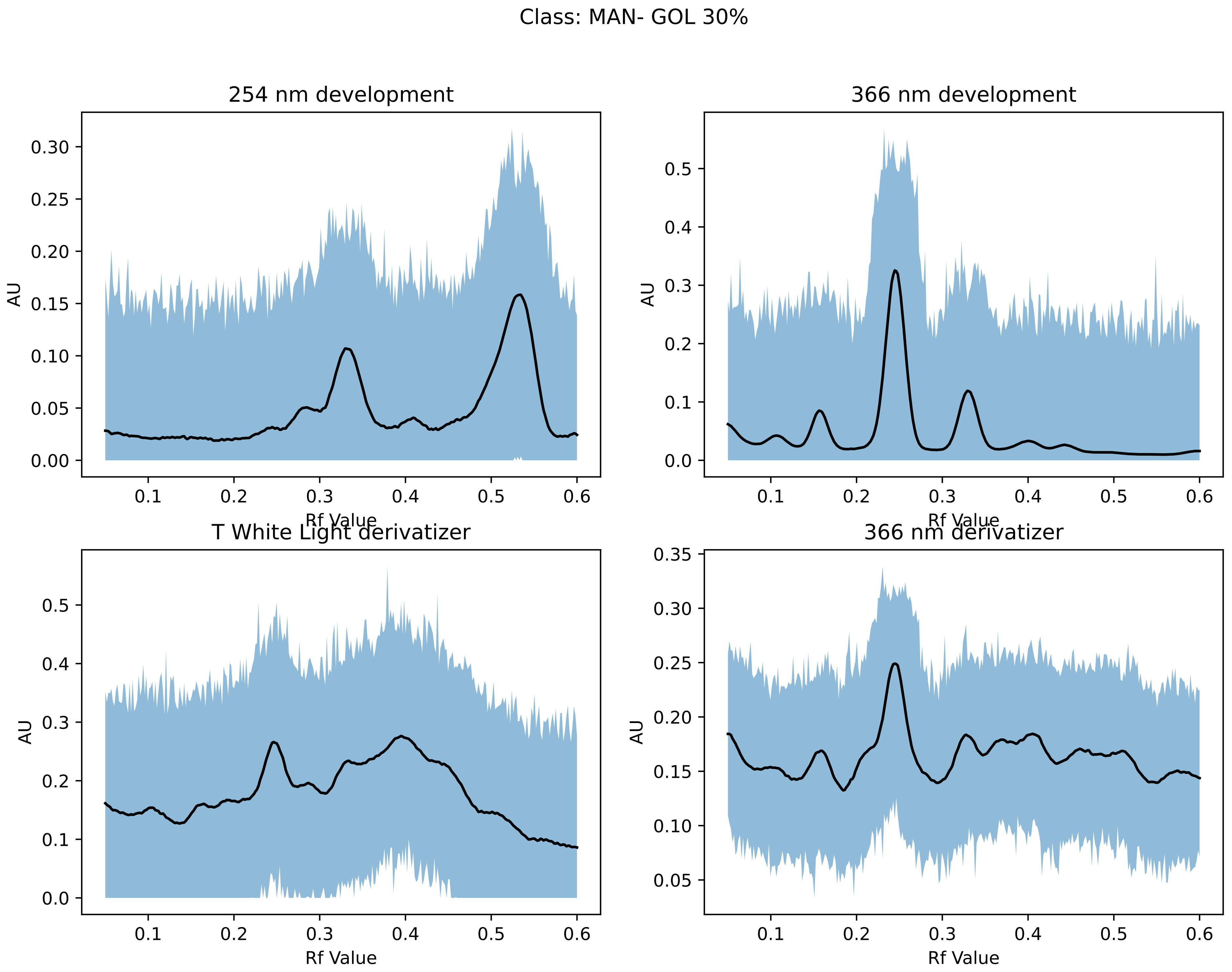
Class: MAN- GOL 30%
254 nm development
366 nm development
T White Light derivatizer
366 nm derivatizer
AU
Rf Value

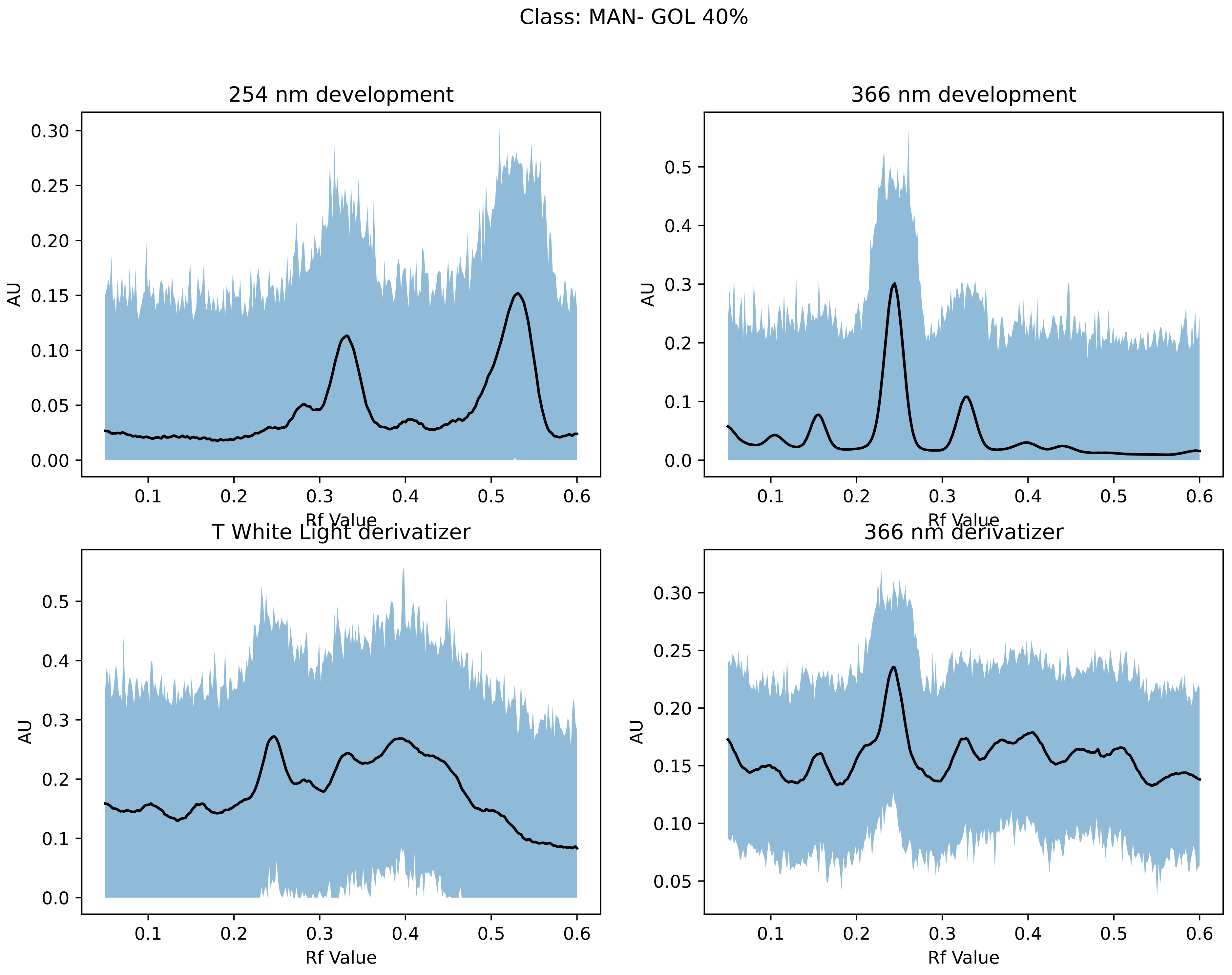
Class: MAN- GOL 40%
254 nm development
366 nm development
T White Light derivatizer
366 nm derivatizer
AU
Rf Value

Class: MAN- GOL 50%

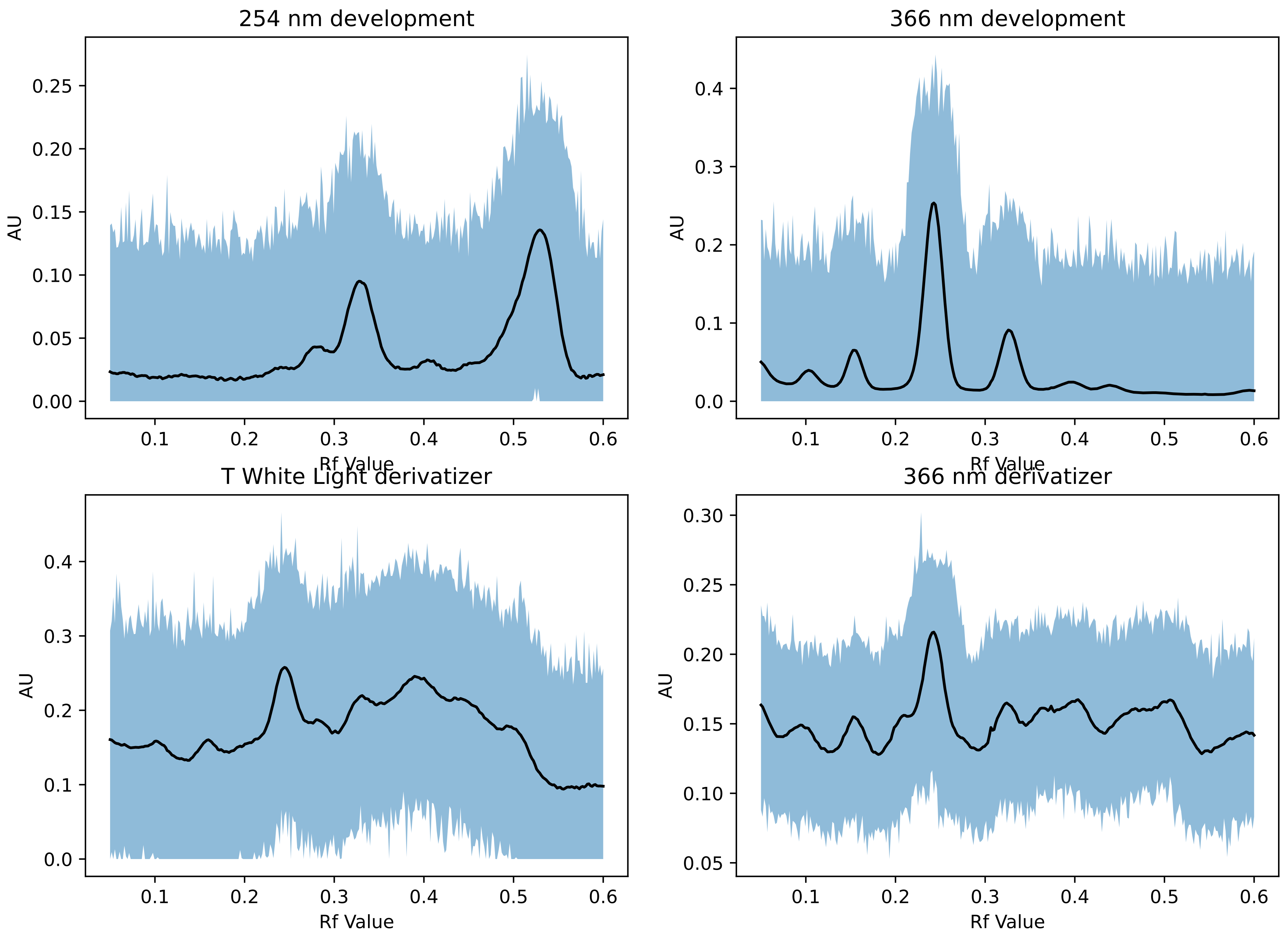

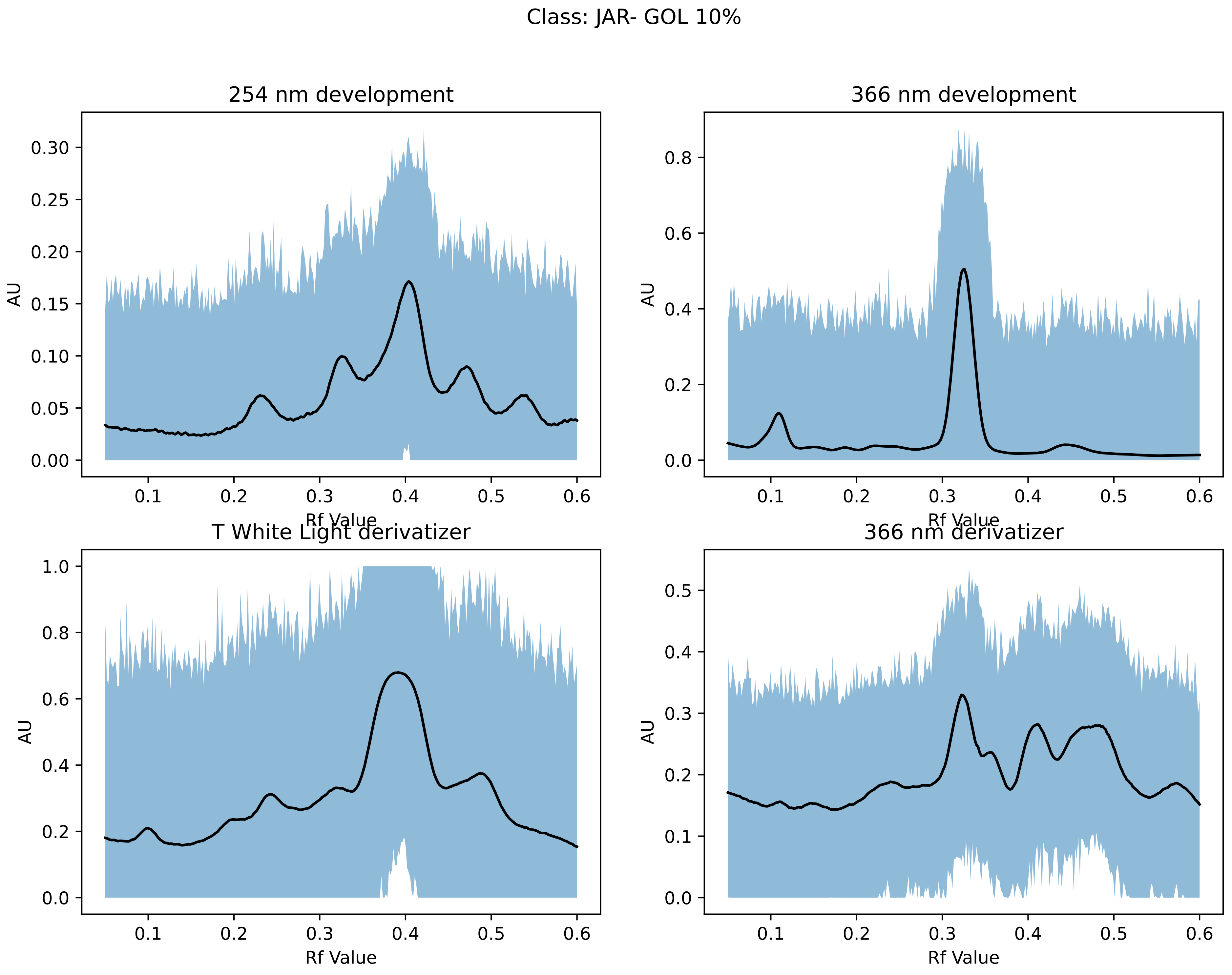
Class: JAR- GOL 10%
254 nm development
366 nm development
T White Light derivatizer
366 nm derivatizer
AU
Rf Value
0.00
0.05
0.10
0.15
0.20
0.25
0.30
0.0
0.2
0.4
0.6
0.8
1.0
0.1
0.3
0.5
0.6

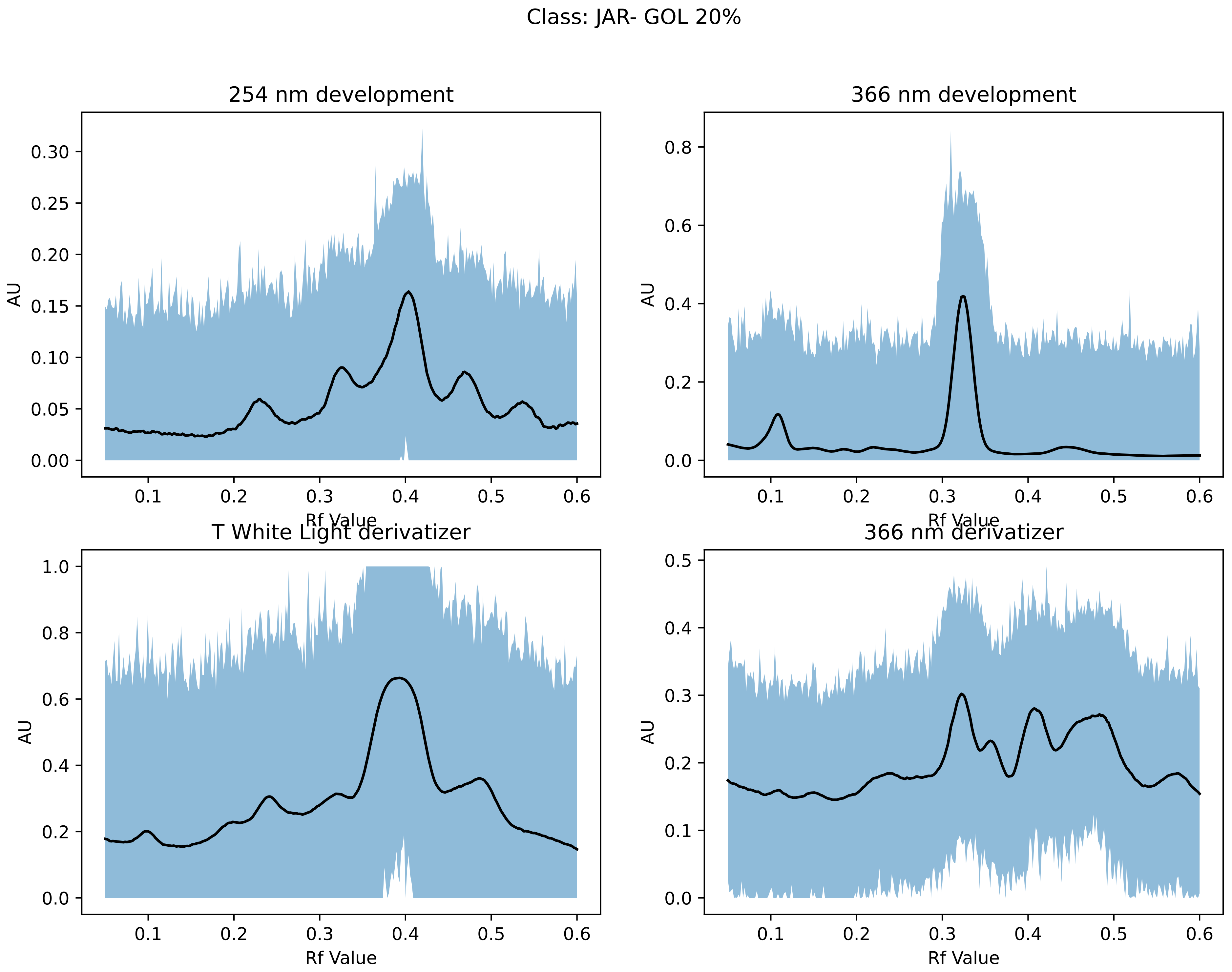

Class: JAR- GOL 20%
254 nm development
366 nm development
T White Light derivatizer
366 nm derivatizer
AU
Rf Value

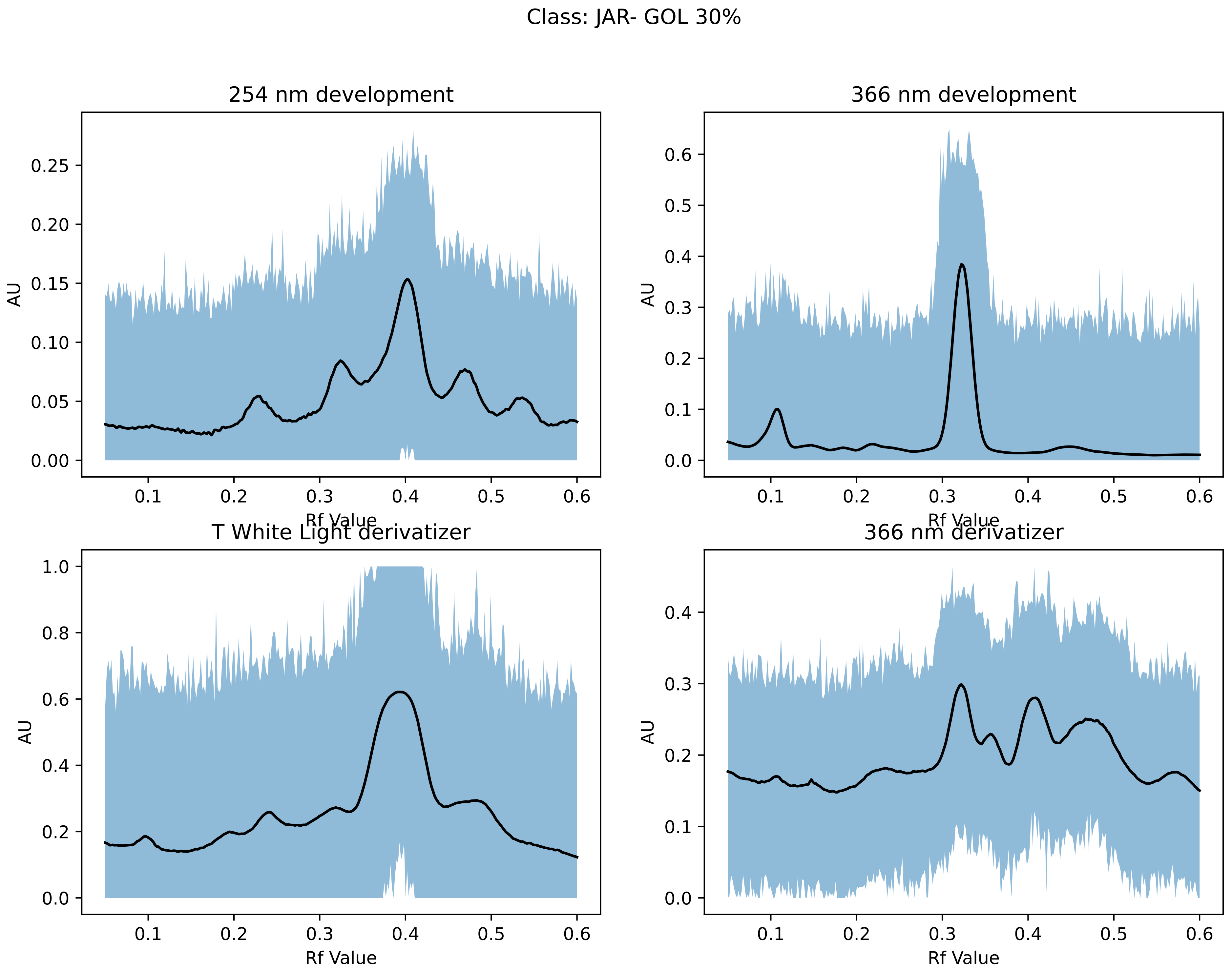

Class: JAR- GOL 30%
254 nm development
366 nm development
T White Light derivatizer
366 nm derivatizer
AU
Rf Value

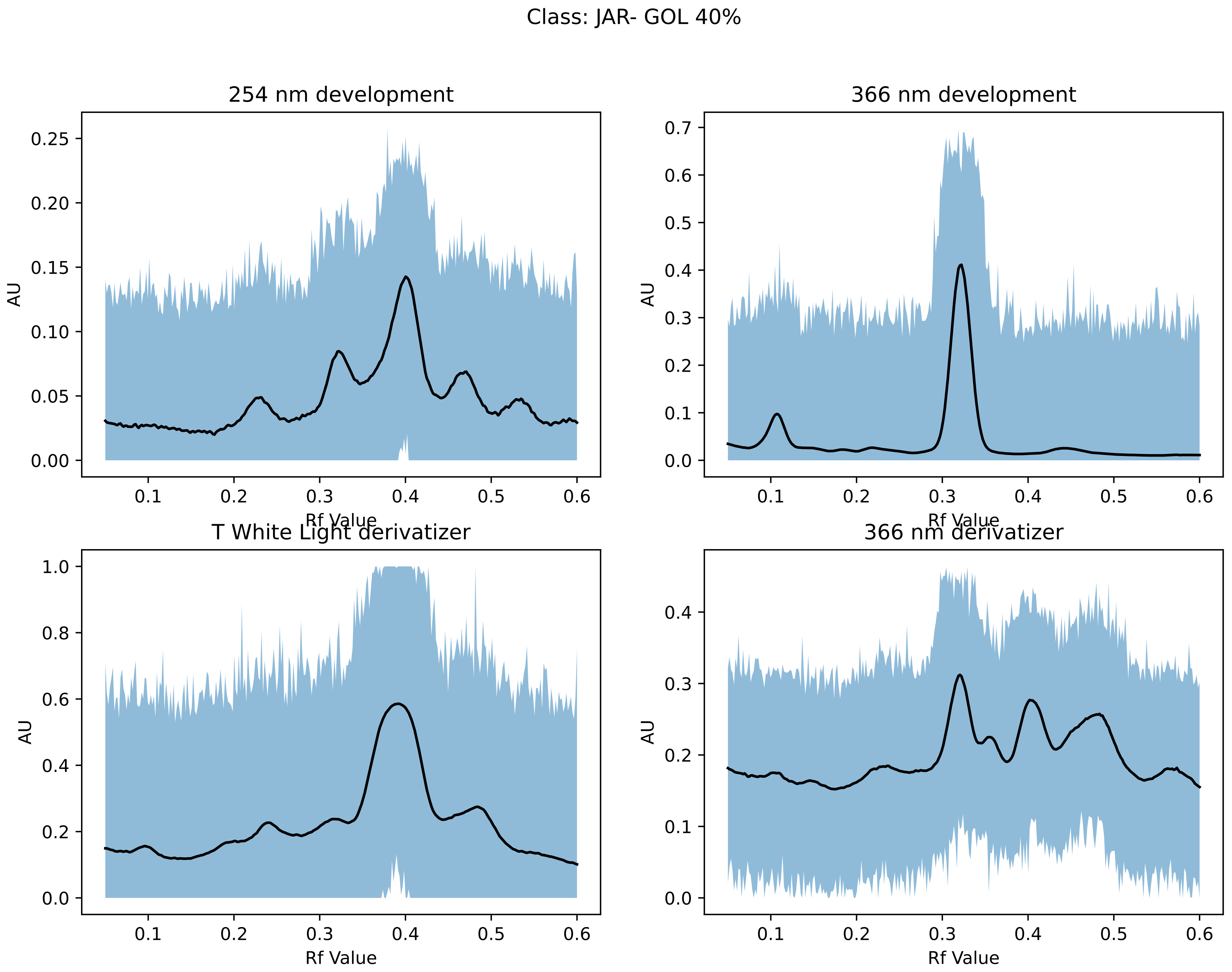
Class: JAR- GOL 40%
254 nm development
366 nm development
T White Light derivatizer
366 nm derivatizer
AU
Rf Value
0.00
0.05
0.10
0.15
0.20
0.25
0.0
0.1
0.2
0.3
0.4
0.5
0.6
0.7
0.8
1.0

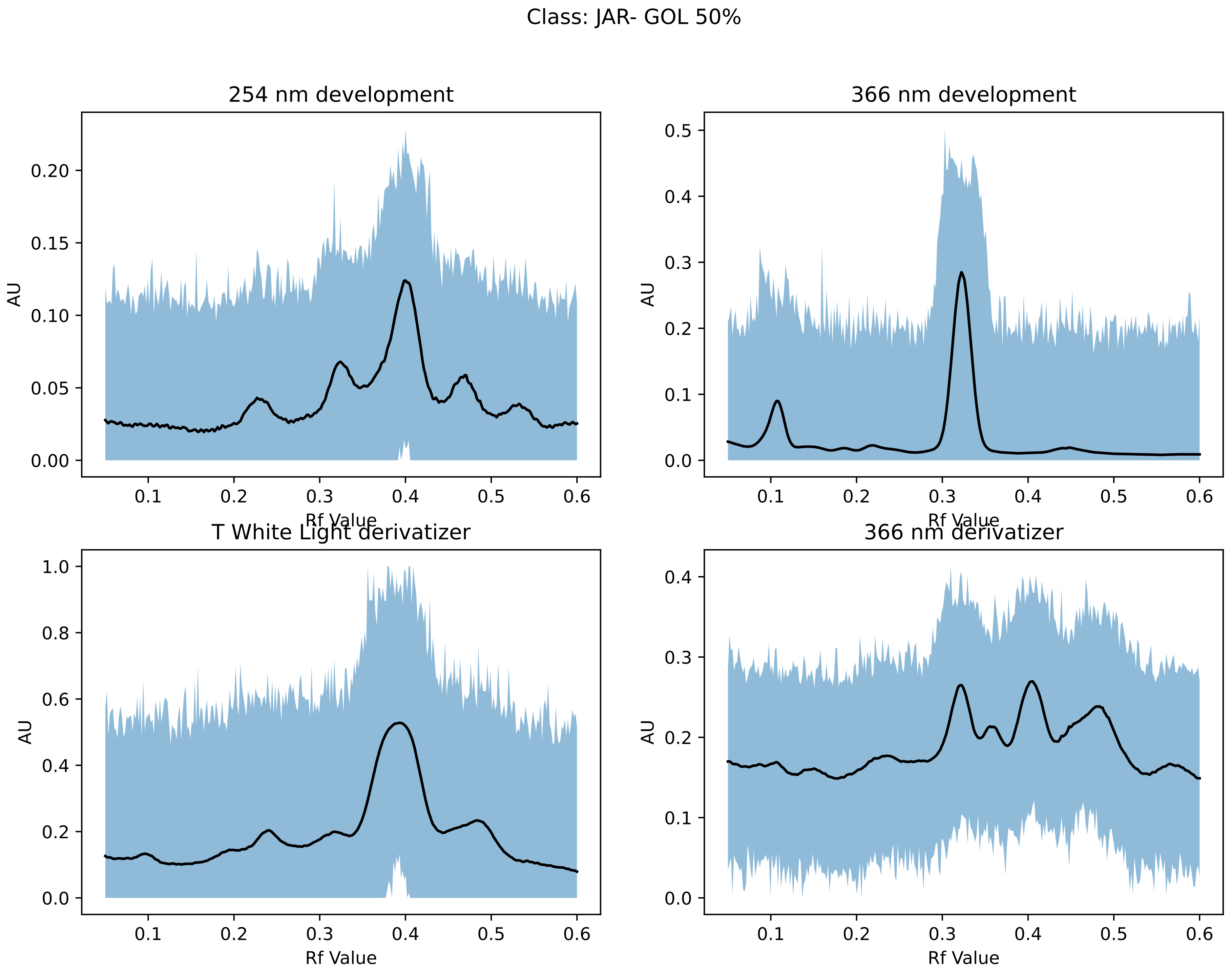
Class: JAR- GOL 50%
254 nm development
366 nm development
T White Light derivatizer
366 nm derivatizer
AU
Rf Value

Class: MAN- MAP 10%

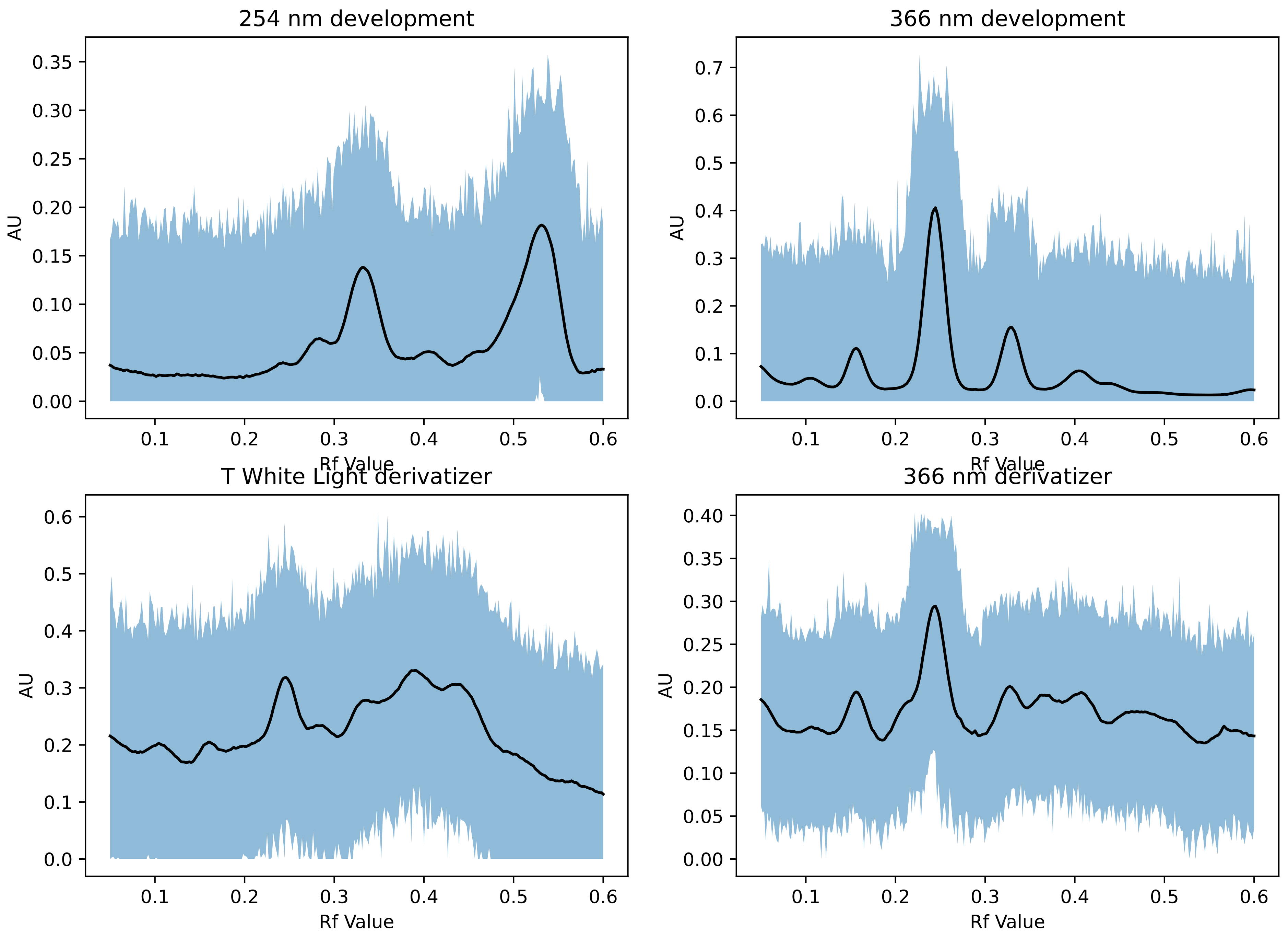

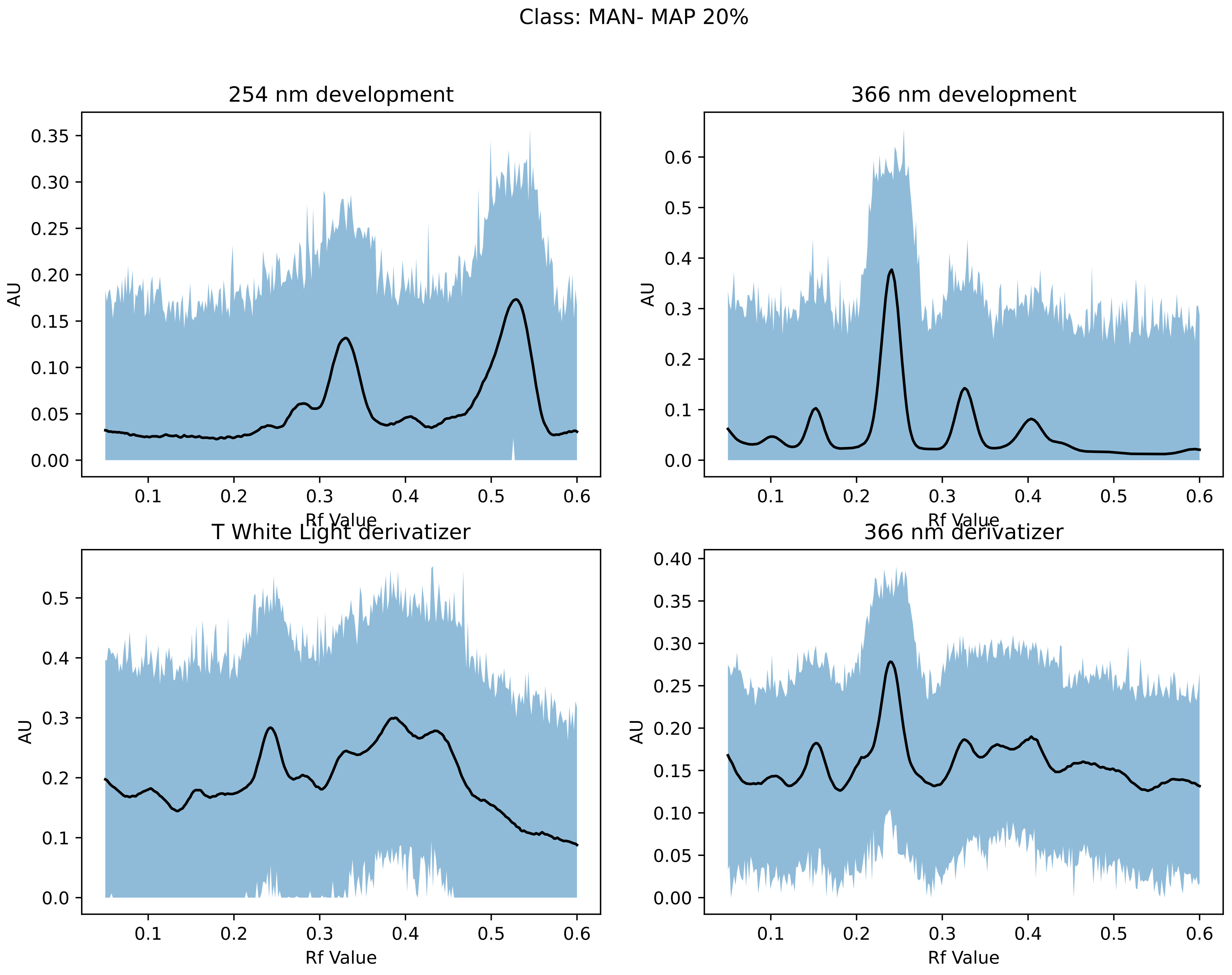
Class: MAN- MAP 20%
254 nm development
366 nm development
T White Light derivatizer
366 nm derivatizer
AU
Rf Value

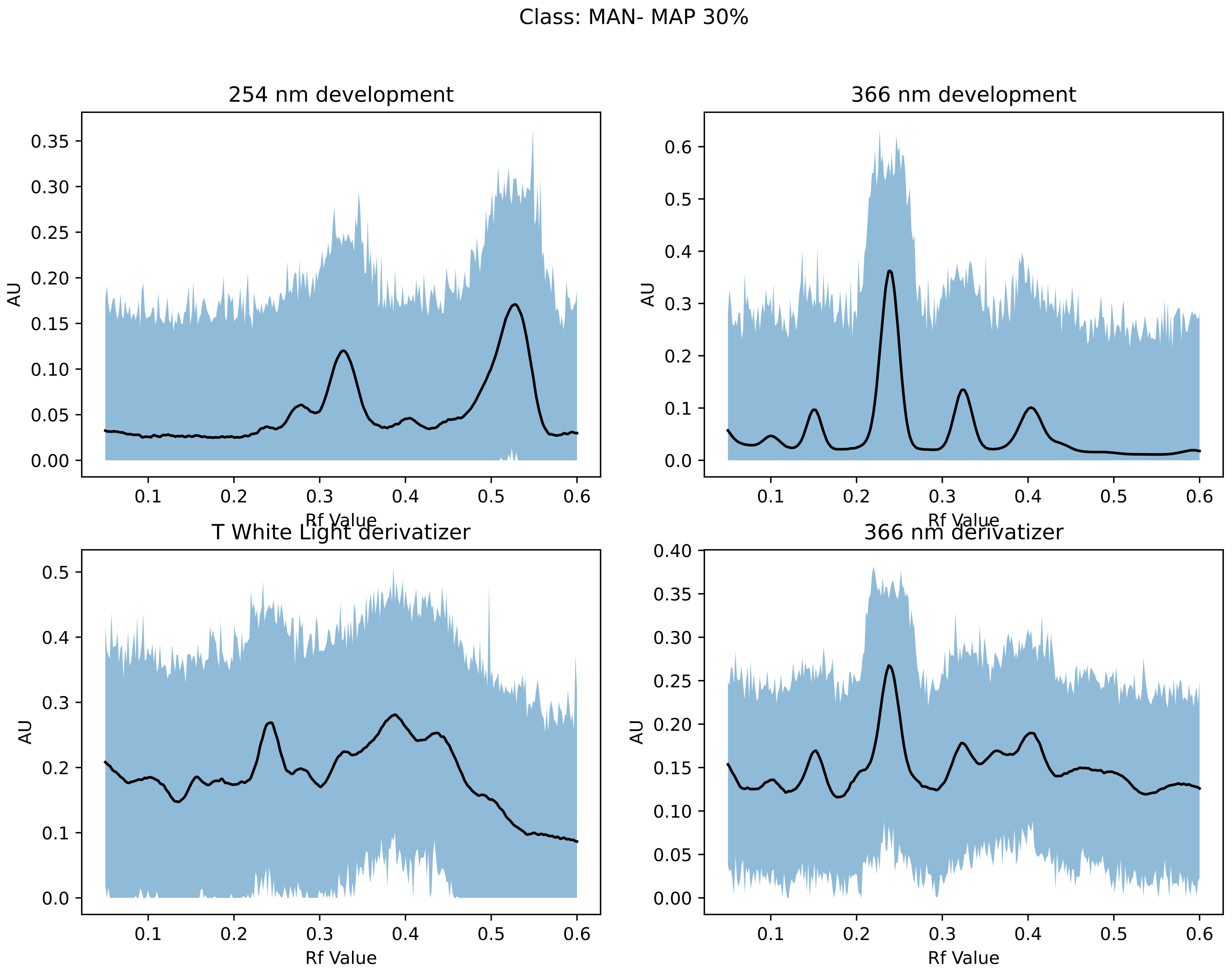

Class: MAN- MAP 30%
254 nm development
366 nm development
T White Light derivatizer
366 nm derivatizer
AU
Rf Value

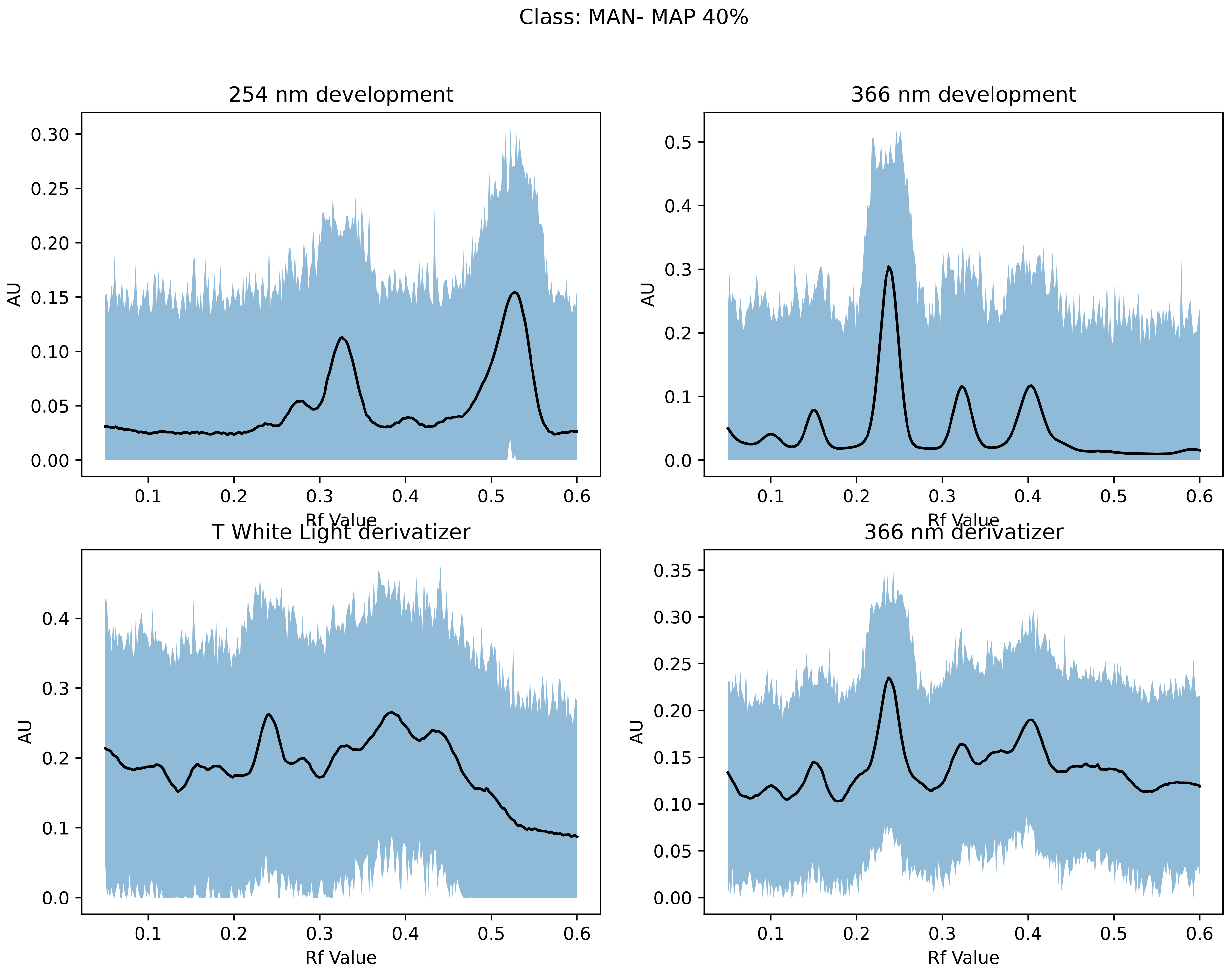
Class: MAN- MAP 40%
254 nm development
366 nm development
T White Light derivatizer
366 nm derivatizer
AU
Rf Value
0.00
0.05
0.10
0.15
0.20
0.25
0.30
0.35
0.0
0.1
0.2
0.3
0.4
0.5
0.6

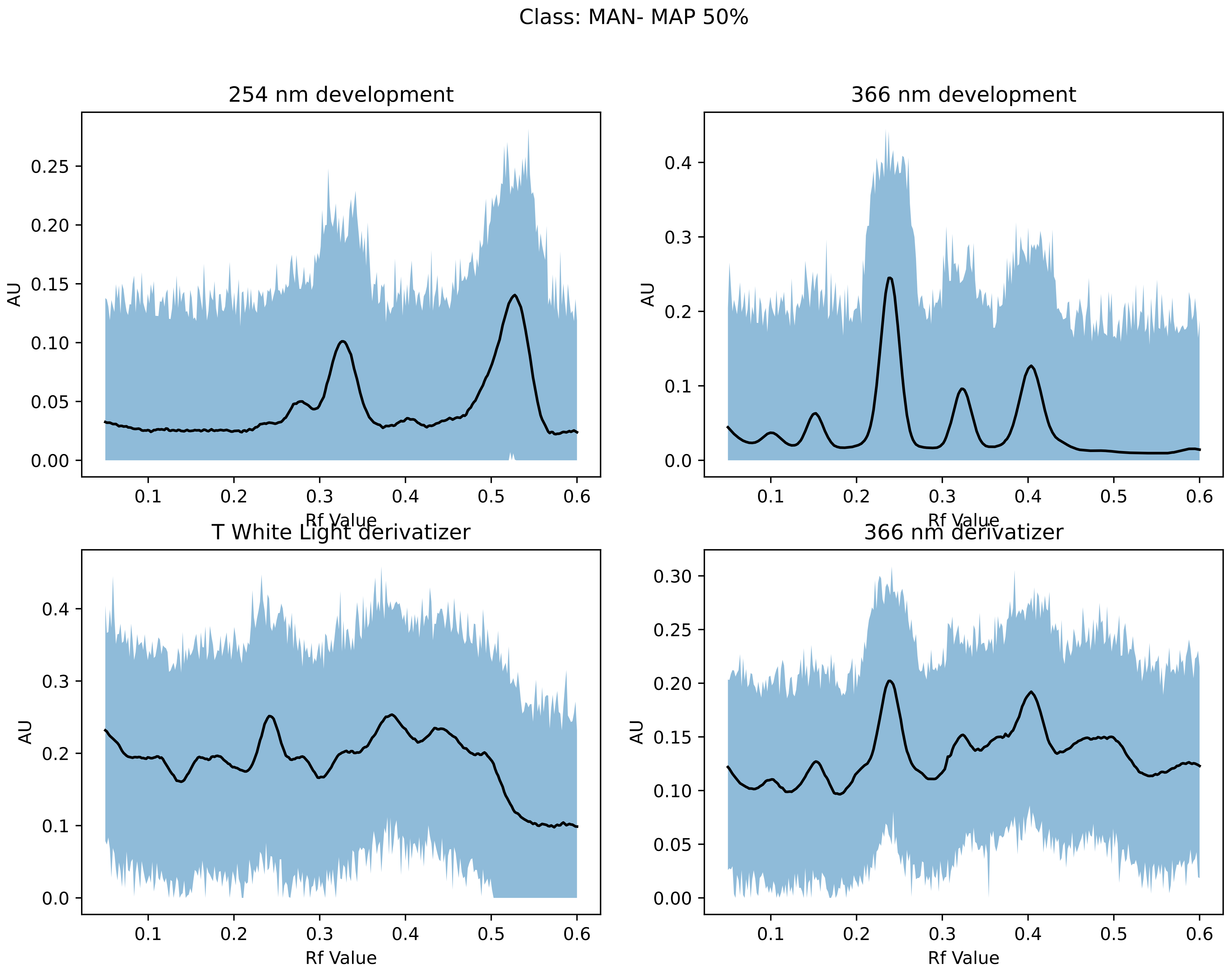

Class: MAN- MAP 50%
254 nm development
366 nm development
T White Light derivatizer
366 nm derivatizer
AU
Rf Value

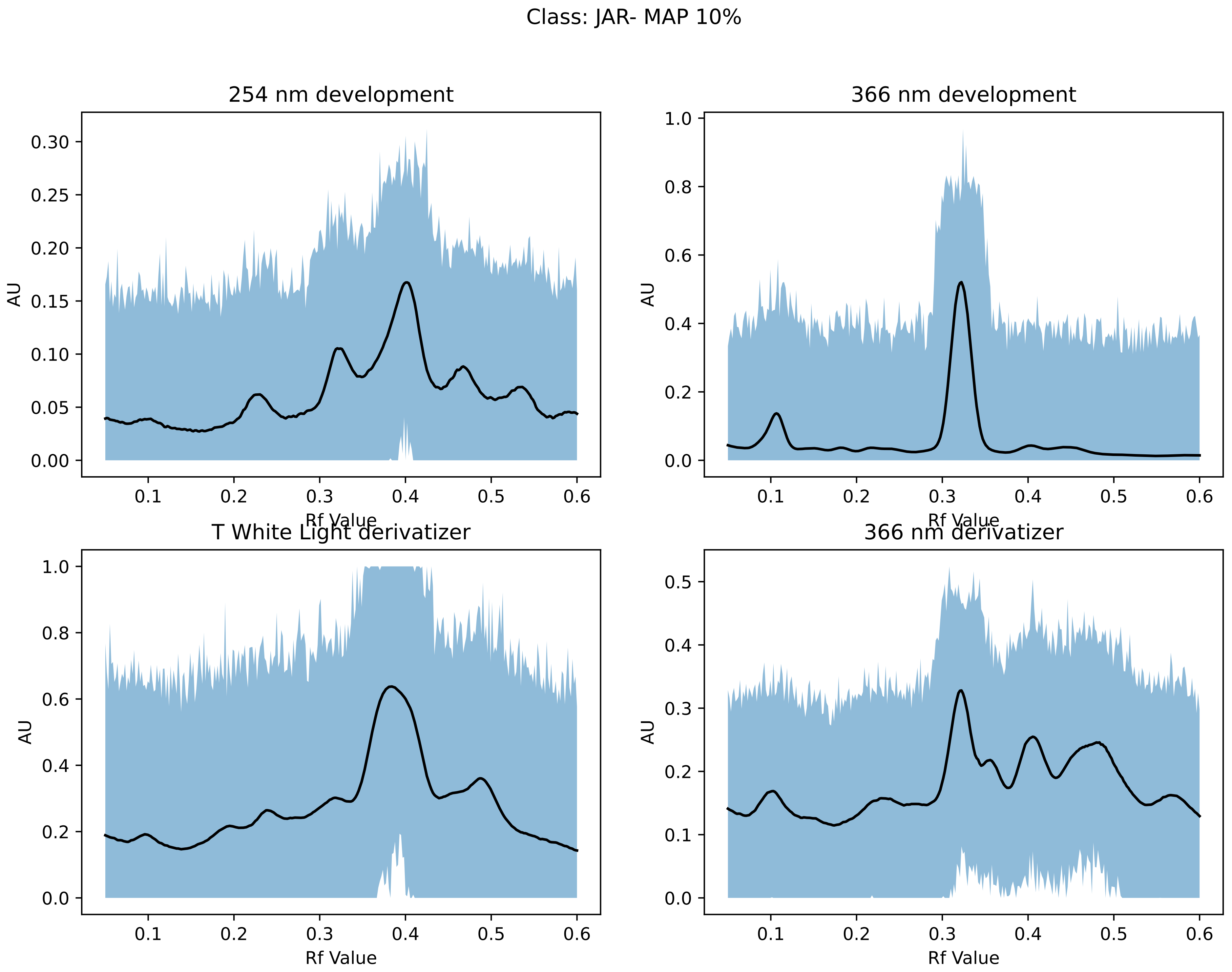
Class: JAR- MAP 10%
254 nm development
366 nm development
T White Light derivatizer
366 nm derivatizer
AU
Rf Value
0.00
0.05
0.10
0.15
0.20
0.25
0.30
0.0
0.2
0.4
0.6
0.8
1.0
0.1
0.2
0.3
0.4
0.5
0.6

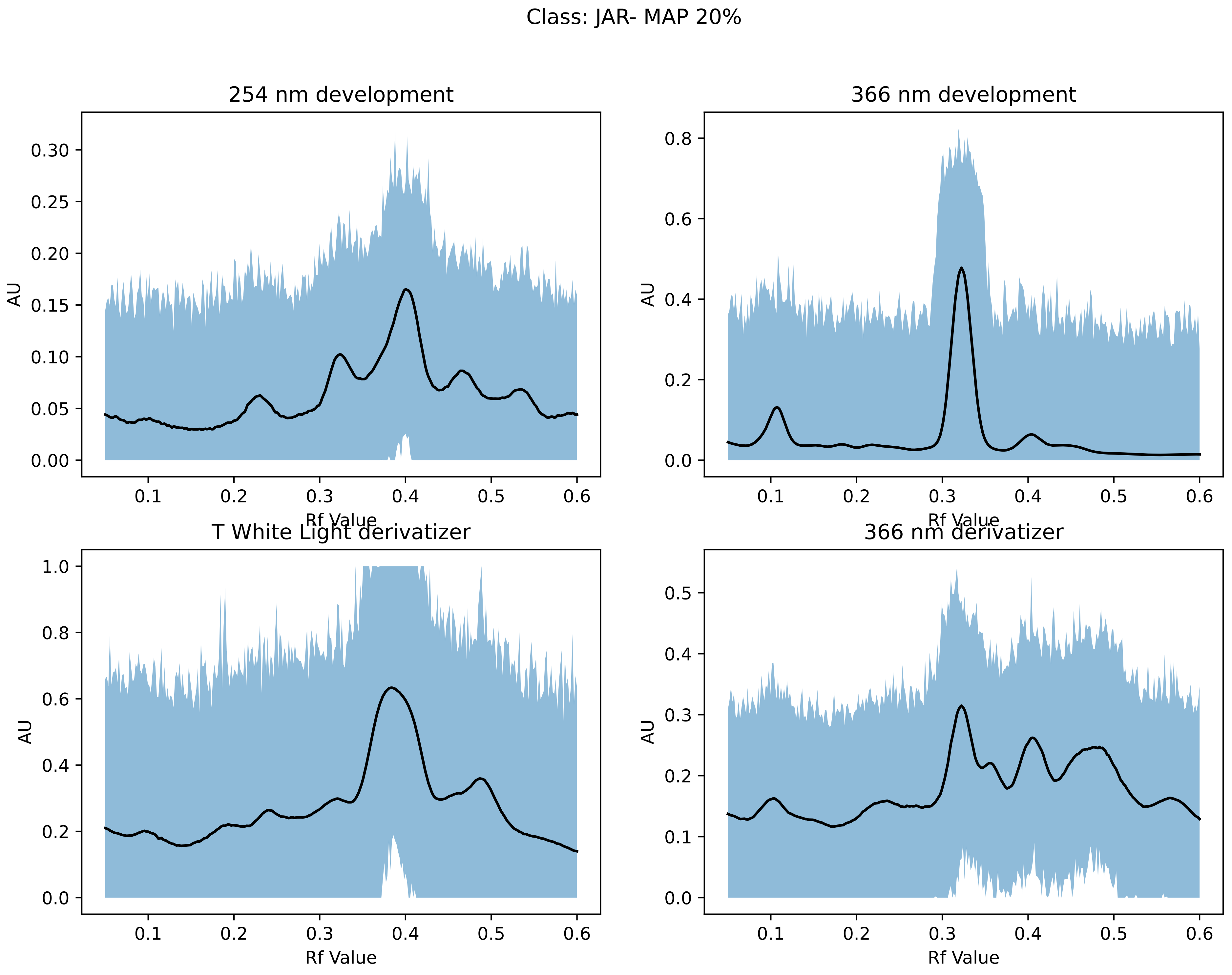
Class: JAR- MAP 20%
254 nm development
366 nm development
T White Light derivatizer
366 nm derivatizer
AU
Rf Value
0.00
0.05
0.10
0.15
0.20
0.25
0.30
0.0
0.2
0.4
0.6
0.8
1.0
0.1
0.3
0.5
0.6

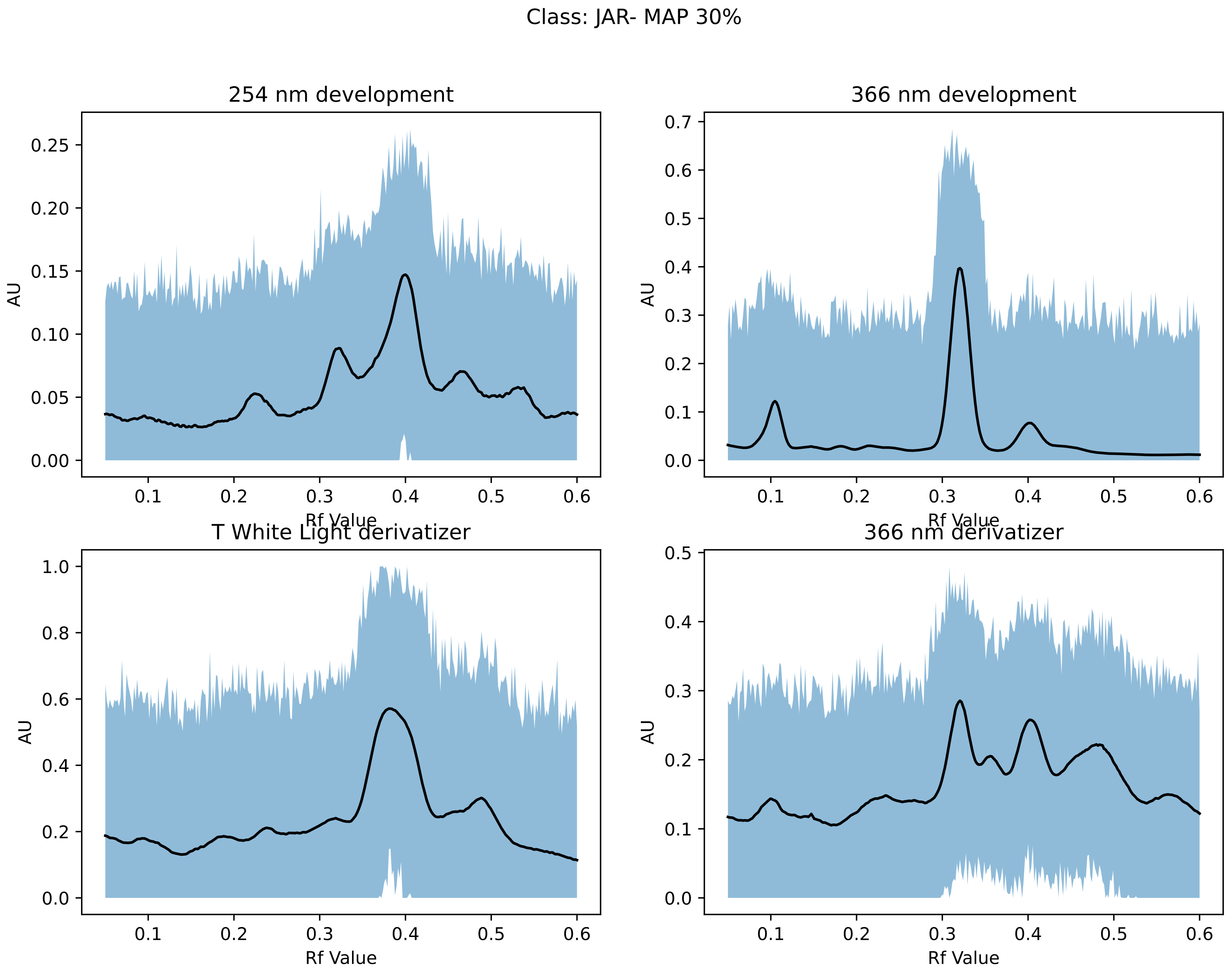
Class: JAR- MAP 30%
254 nm development
366 nm development
T White Light derivatizer
366 nm derivatizer
AU
Rf Value
0.00
0.05
0.10
0.15
0.20
0.25
0.0
0.1
0.2
0.3
0.4
0.5
0.6
0.7
0.8
1.0

Class: JAR- MAP 40%

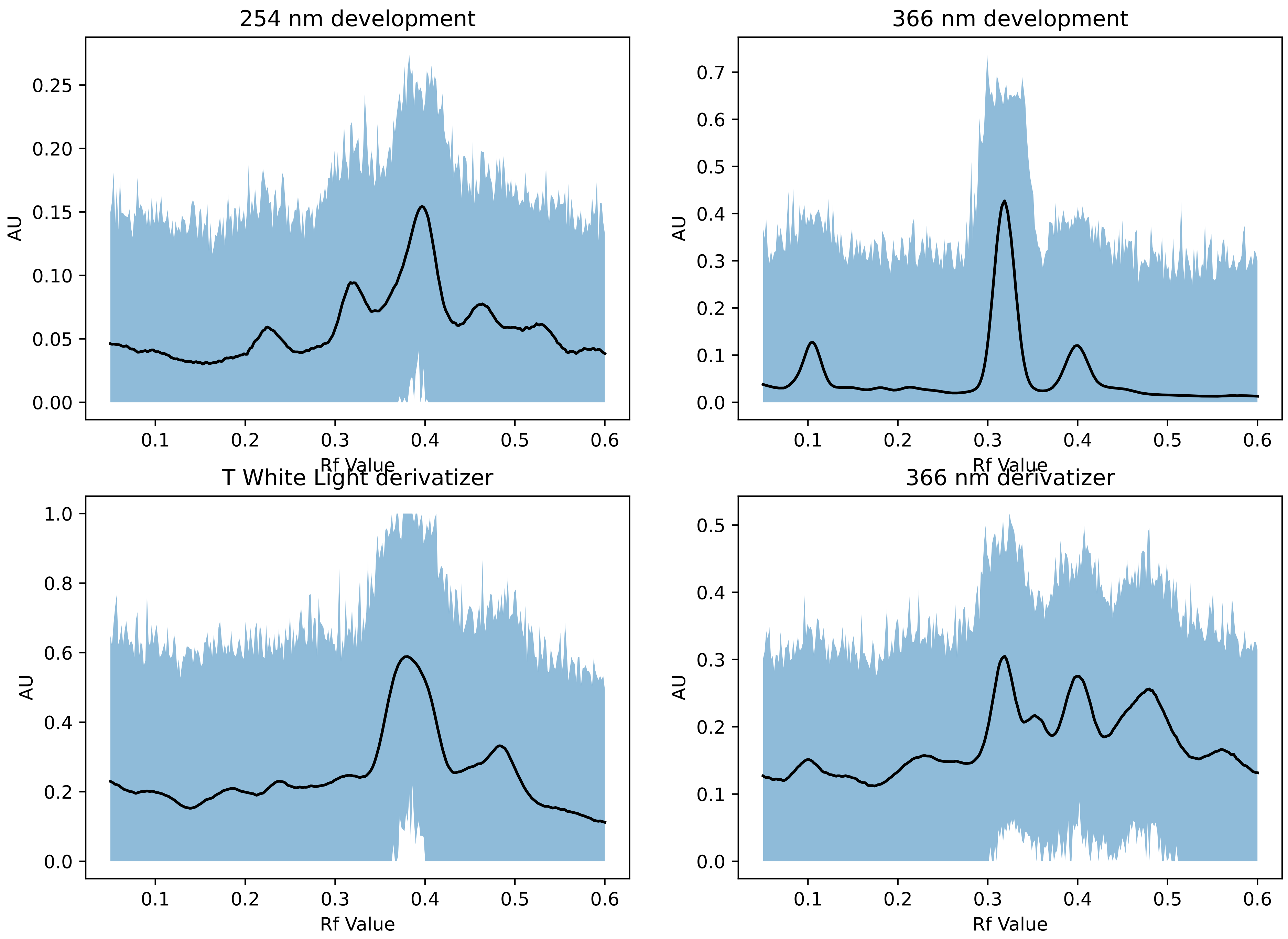

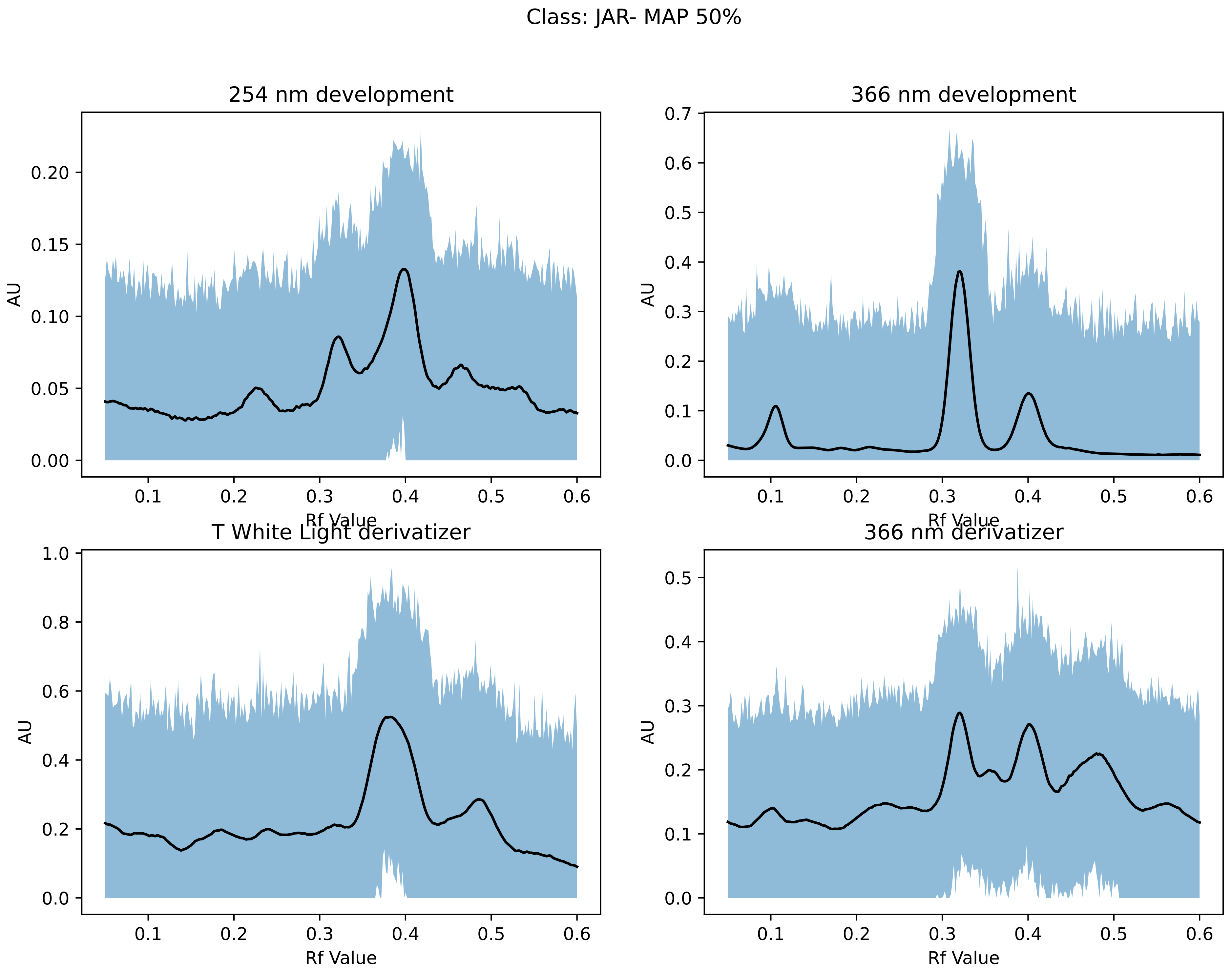
Class: JAR- MAP 50%
254 nm development
366 nm development
T White Light derivatizer
366 nm derivatizer
AU
Rf Value

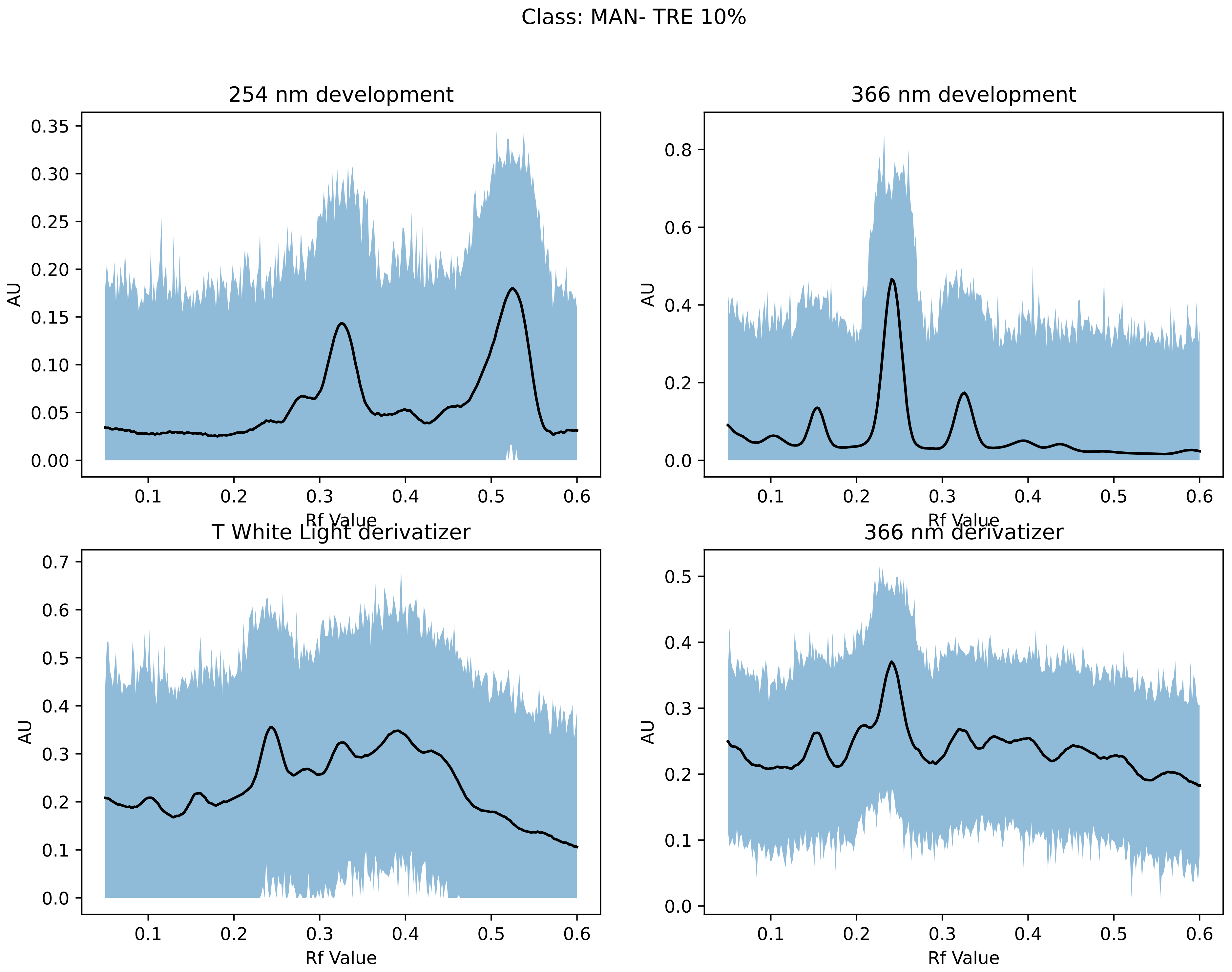

Class: MAN- TRE 10%
254 nm development
366 nm development
T White Light derivatizer
366 nm derivatizer
AU
Rf Value
0.00
0.05
0.10
0.15
0.20
0.25
0.30
0.35
0.0
0.1
0.2
0.3
0.4
0.5
0.6
0.7
0.8

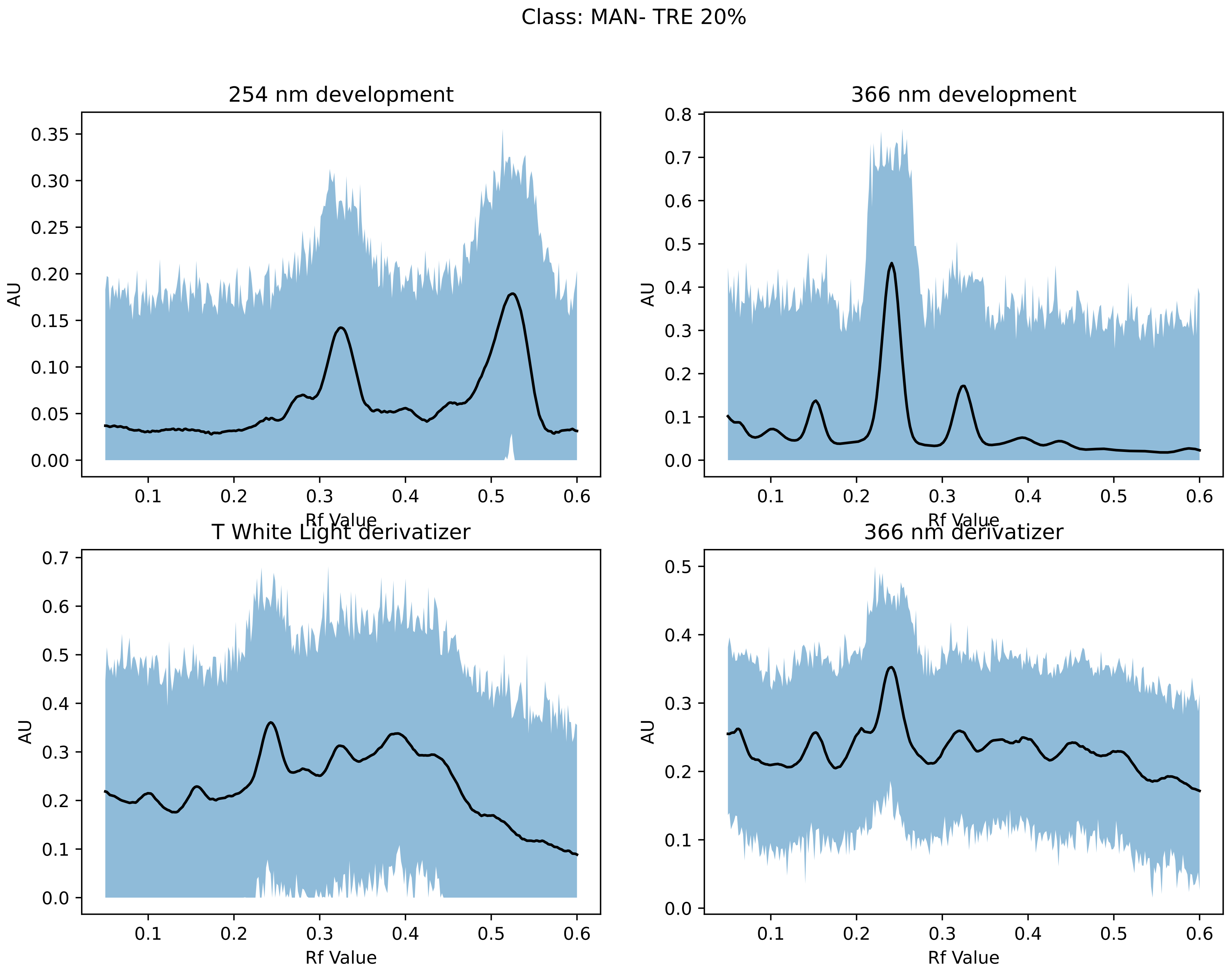

Class: MAN- TRE 20%
254 nm development
366 nm development
T White Light derivatizer
366 nm derivatizer
AU
Rf Value
0.00
0.05
0.10
0.15
0.20
0.25
0.30
0.35
0.0
0.1
0.2
0.3
0.4
0.5
0.6
0.7
0.8

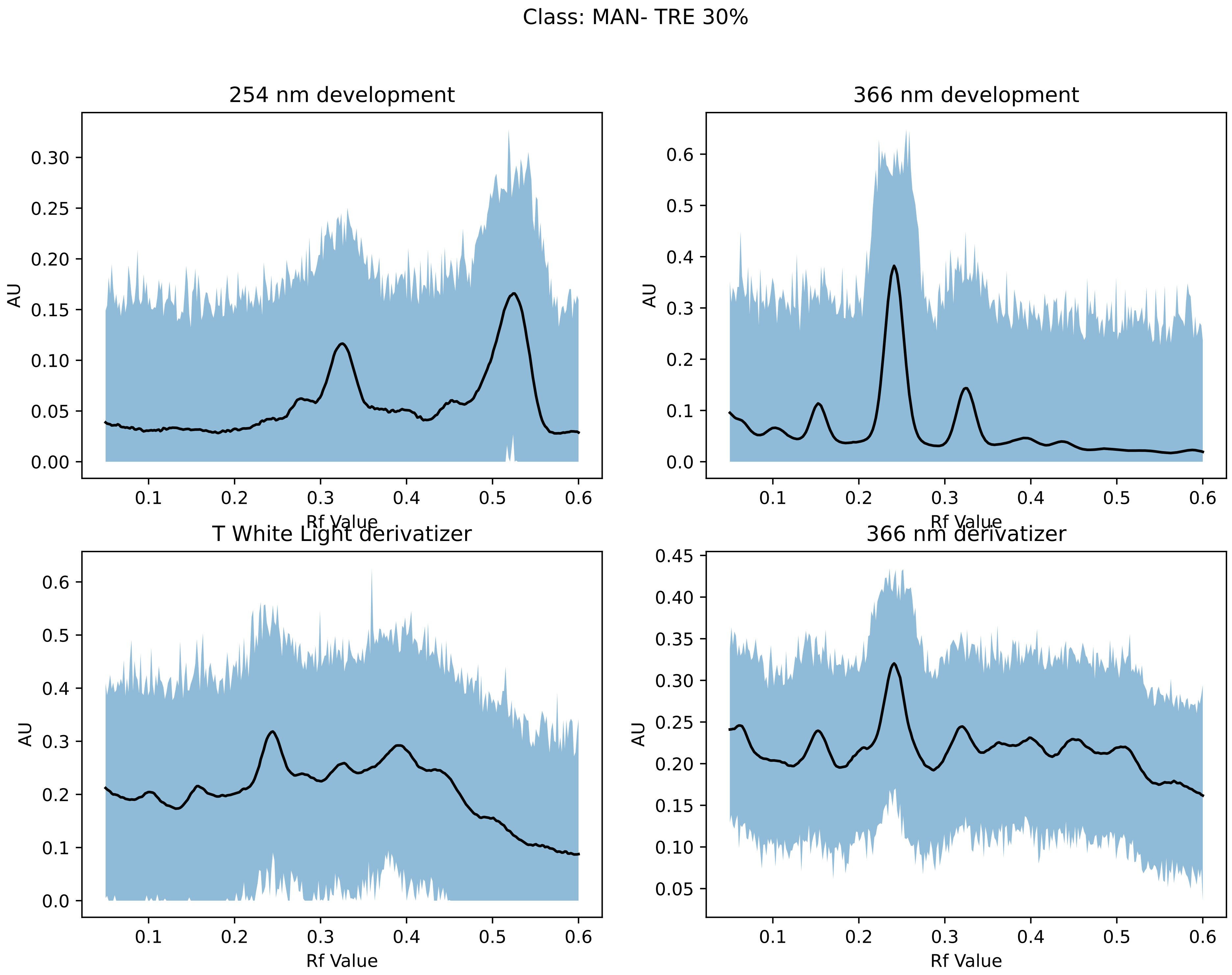
Class: MAN- TRE 30%
254 nm development
366 nm development
T White Light derivatizer
366 nm derivatizer
AU
Rf Value
0.00
0.05
0.10
0.15
0.20
0.25
0.30
0.0
0.1
0.2
0.3
0.4
0.5
0.6
0.35
0.40
0.45

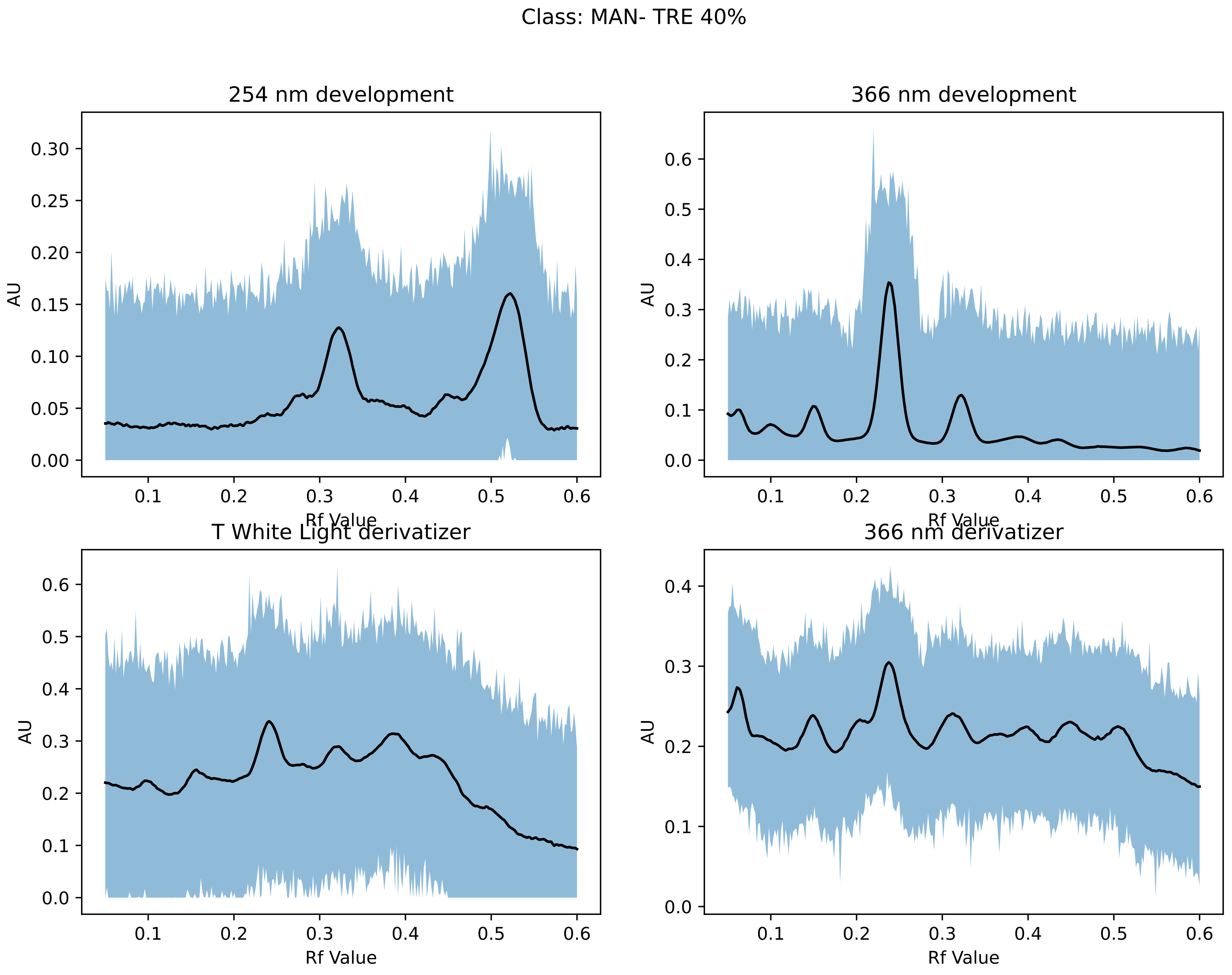

Class: MAN- TRE 40%
254 nm development
366 nm development
T White Light derivatizer
366 nm derivatizer
AU
Rf Value
0.30
0.25
0.20
0.15
0.10
0.05
0.00
0.6
0.5
0.4
0.3
0.2
0.1
0.0
0.1
0.2
0.3
0.4
0.5
0.6

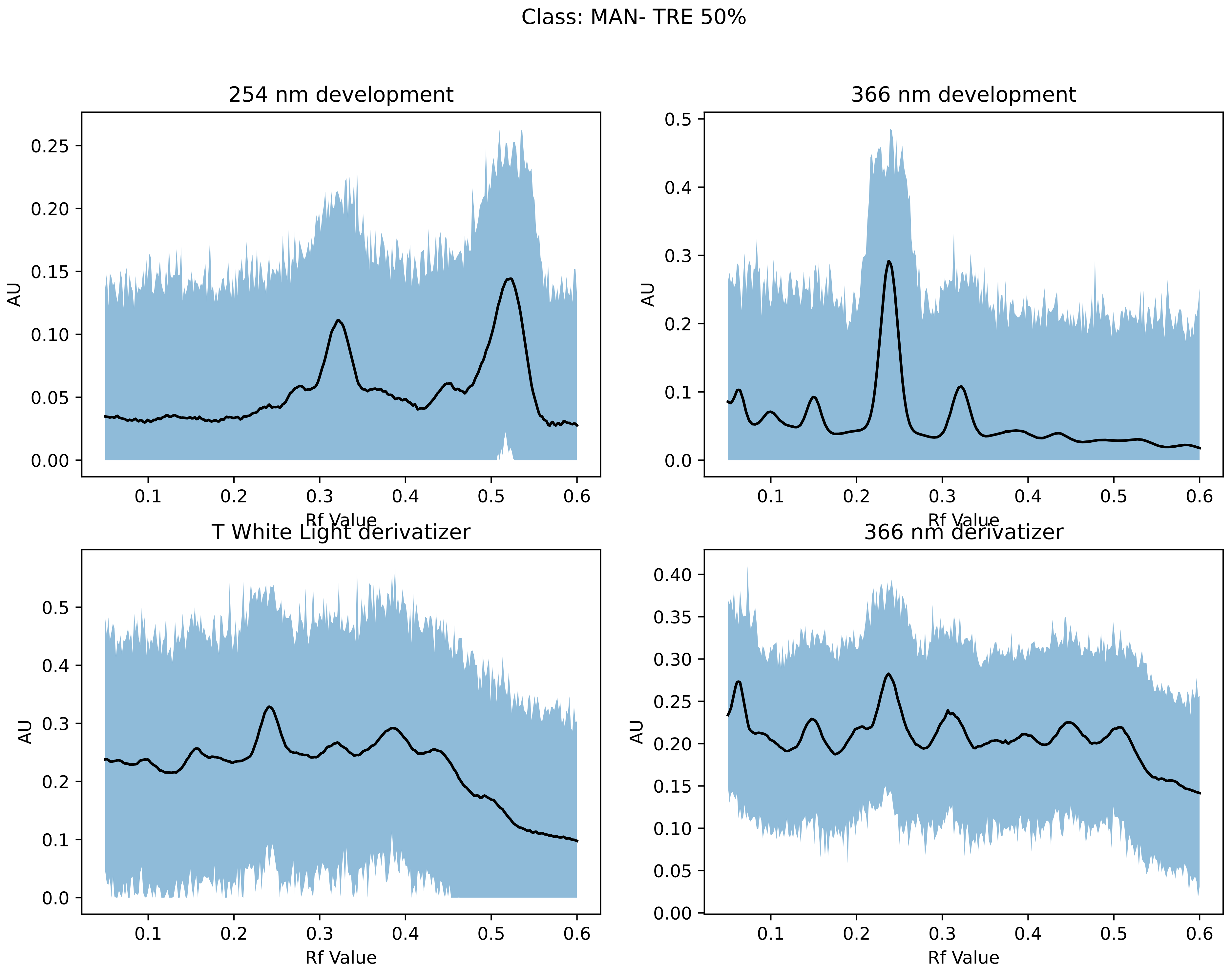

Class: MAN- TRE 50%
254 nm development
366 nm development
T White Light derivatizer
366 nm derivatizer
AU
Rf Value
0.00
0.05
0.10
0.15
0.20
0.25
0.0
0.1
0.2
0.3
0.4
0.5
0.6
0.30
0.35
0.40

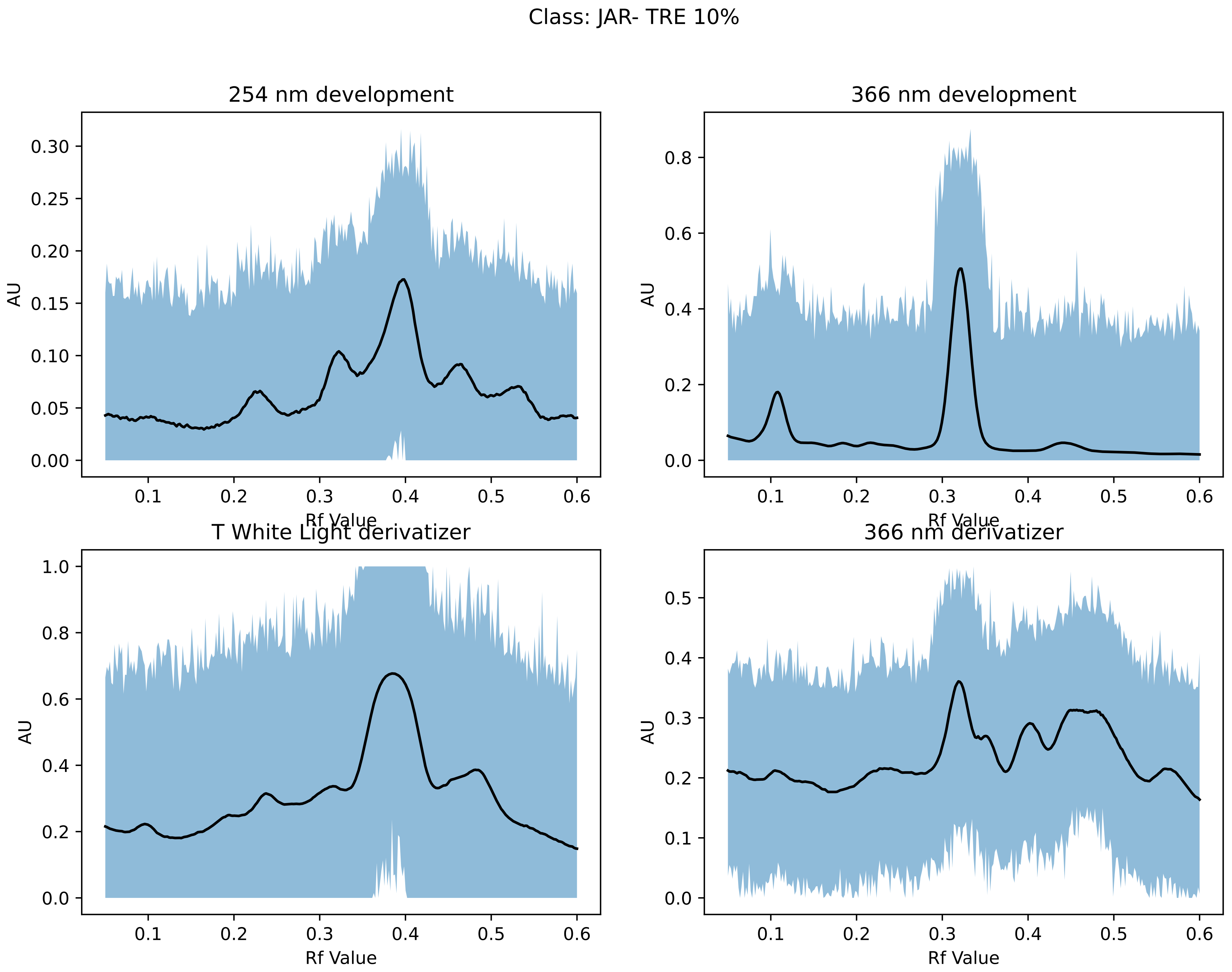

Class: JAR- TRE 10%
254 nm development
366 nm development
T White Light derivatizer
366 nm derivatizer
AU
Rf Value
0.00
0.05
0.10
0.15
0.20
0.25
0.30
0.0
0.2
0.4
0.6
0.8
1.0
0.1
0.3
0.5

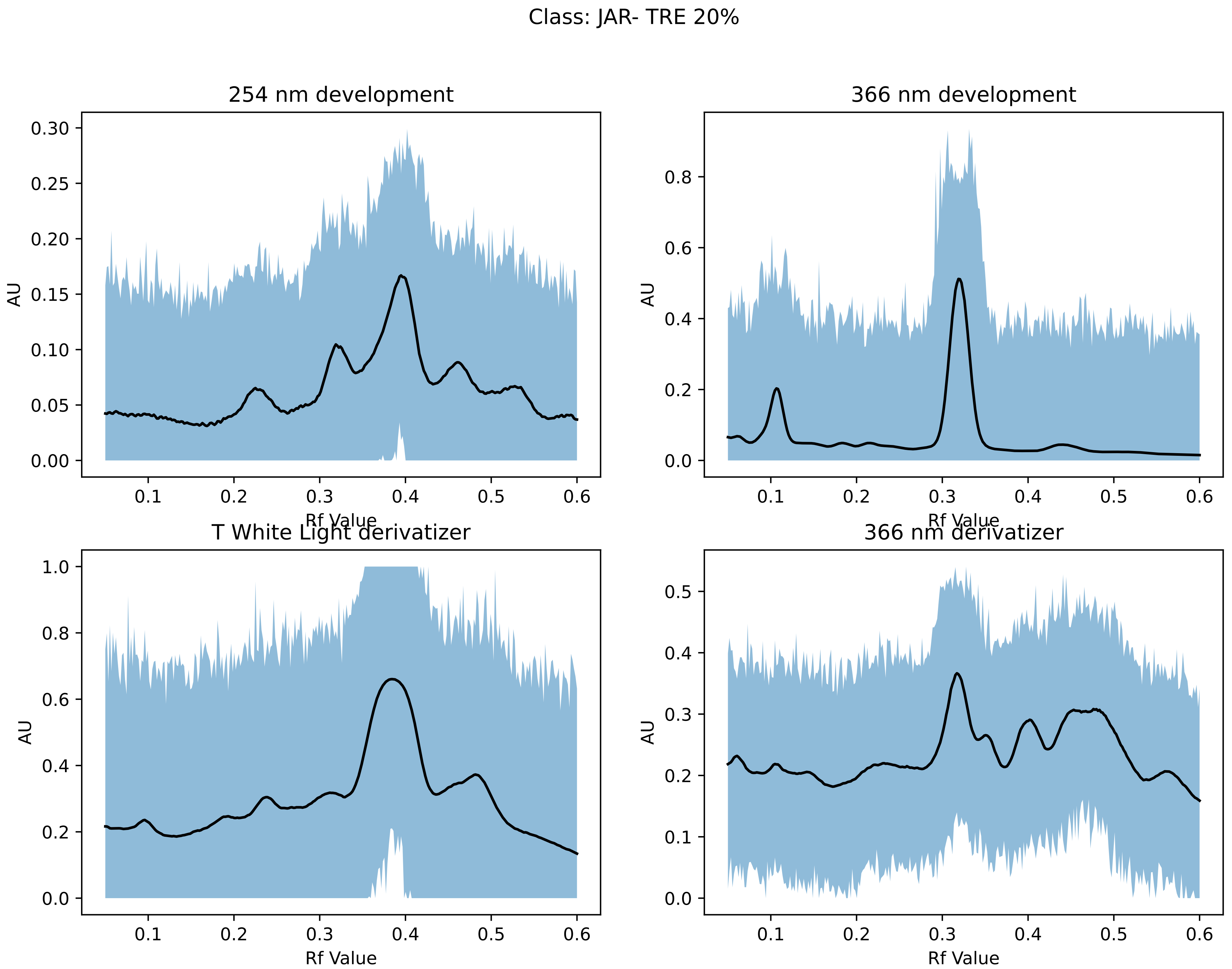

Class: JAR- TRE 20%
254 nm development
366 nm development
T White Light derivatizer
366 nm derivatizer
AU
Rf Value

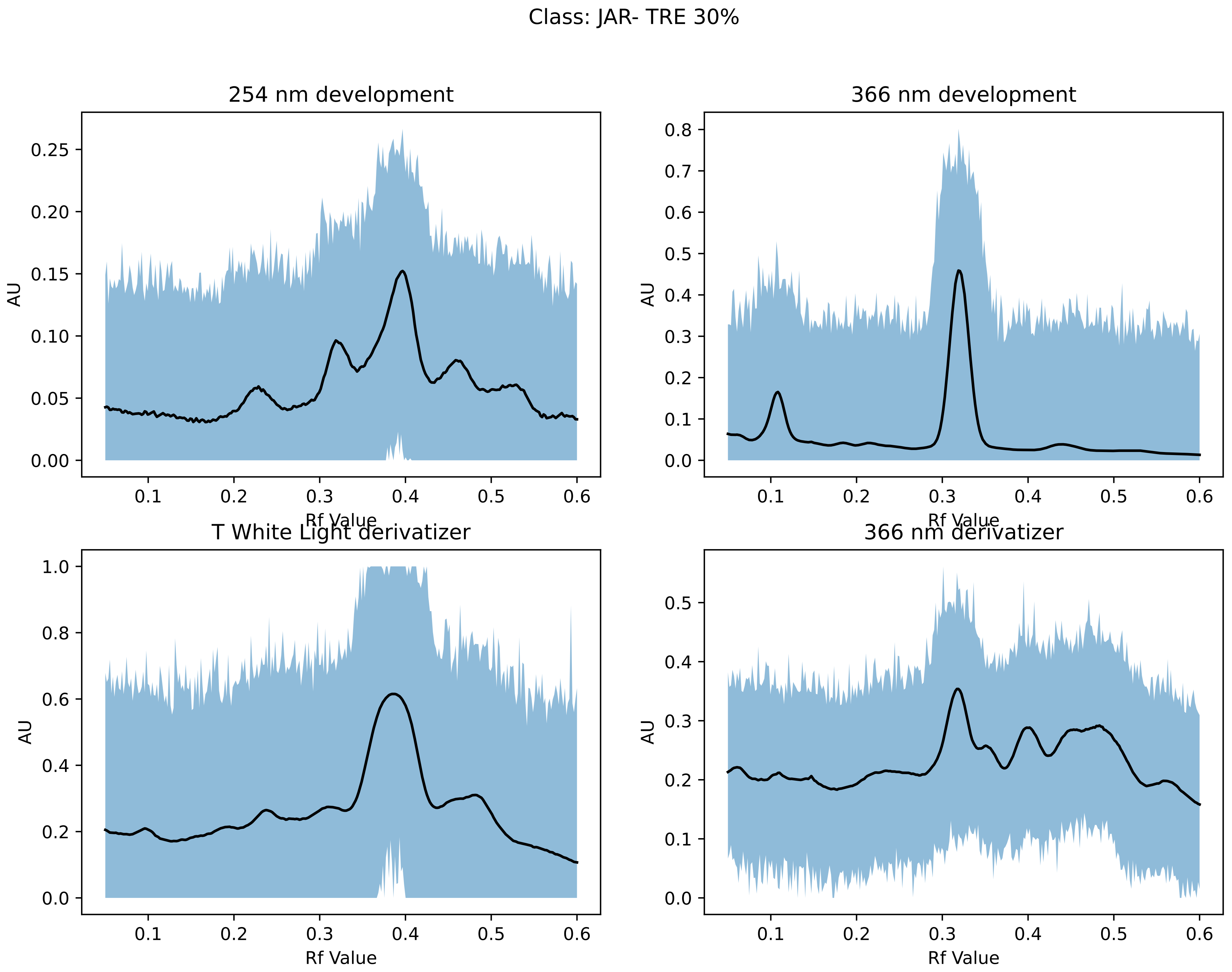

Class: JAR- TRE 30%
254 nm development
366 nm development
T White Light derivatizer
366 nm derivatizer
AU
Rf Value
0.00
0.05
0.10
0.15
0.20
0.25
0.0
0.1
0.2
0.3
0.4
0.5
0.6
0.7
0.8
1.0

Class: JAR- TRE 40%

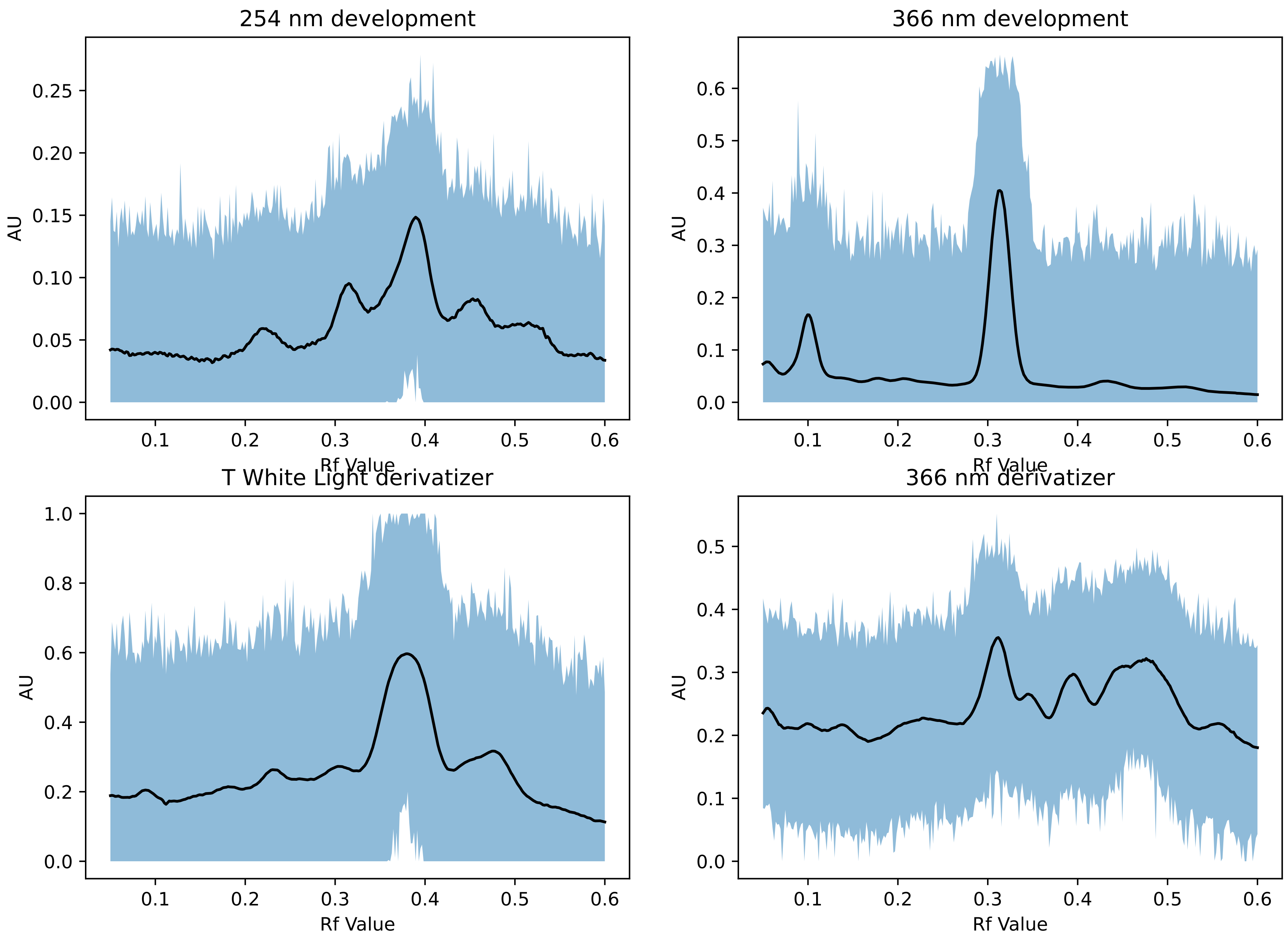

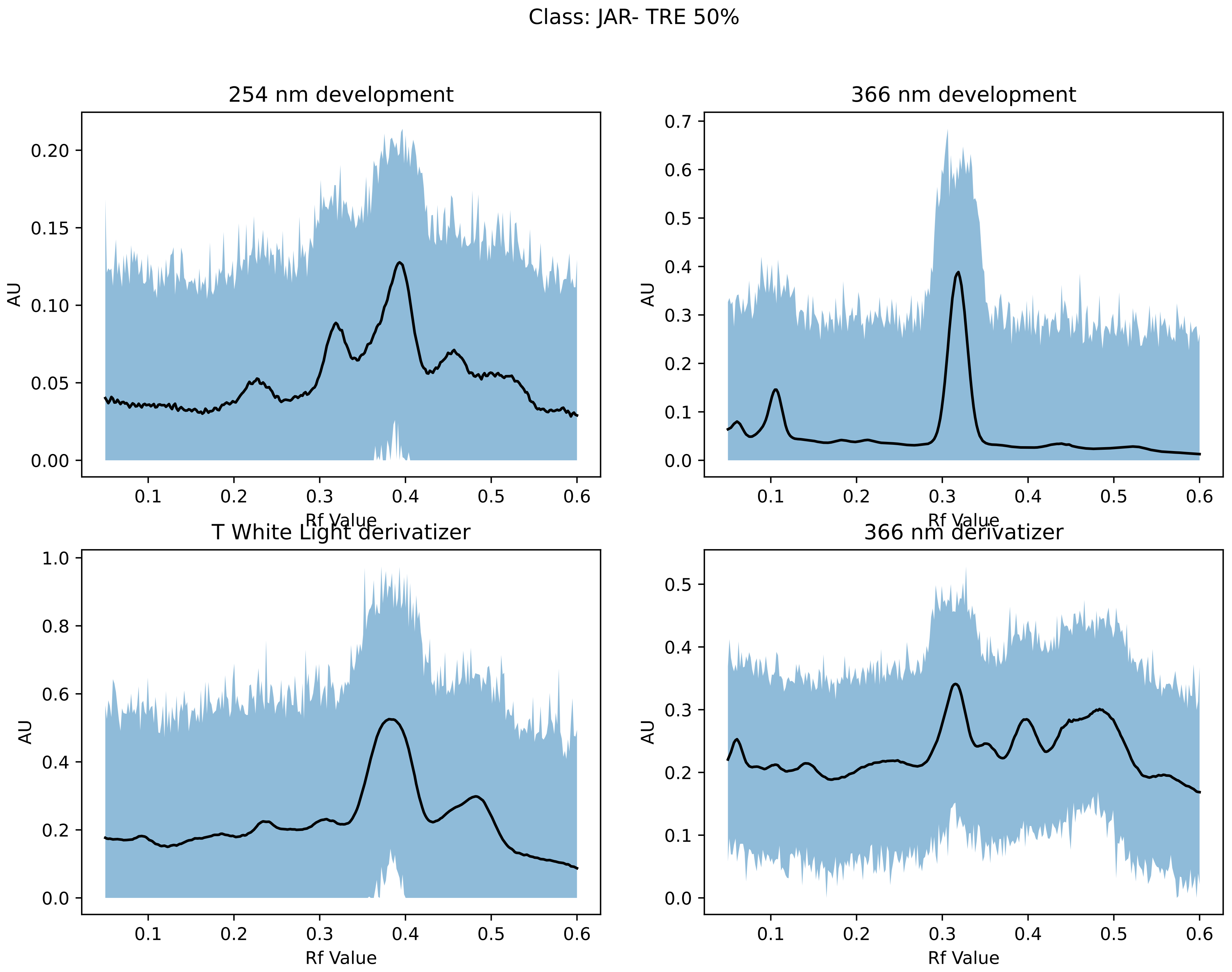
Class: JAR- TRE 50%
254 nm development
366 nm development
T White Light derivatizer
366 nm derivatizer
AU
Rf Value
0.00
0.05
0.10
0.15
0.20
0.0
0.1
0.2
0.3
0.4
0.5
0.6
0.7
0.8
1.0

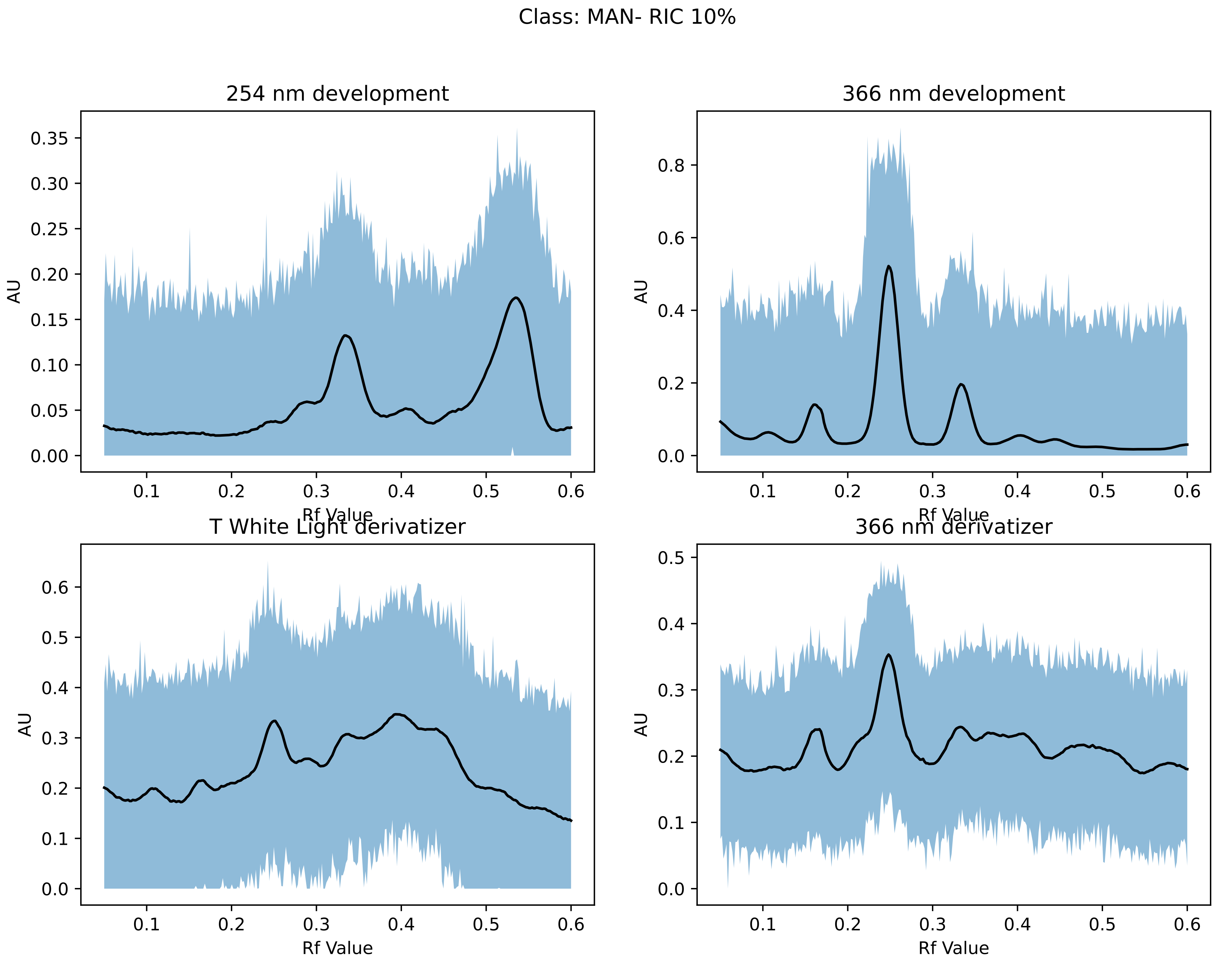
Class: MAN- RIC 10%
254 nm development
366 nm development
T White Light derivatizer
366 nm derivatizer
AU
Rf Value
0.00
0.05
0.10
0.15
0.20
0.25
0.30
0.35
0.0
0.1
0.2
0.3
0.4
0.5
0.6
0.8

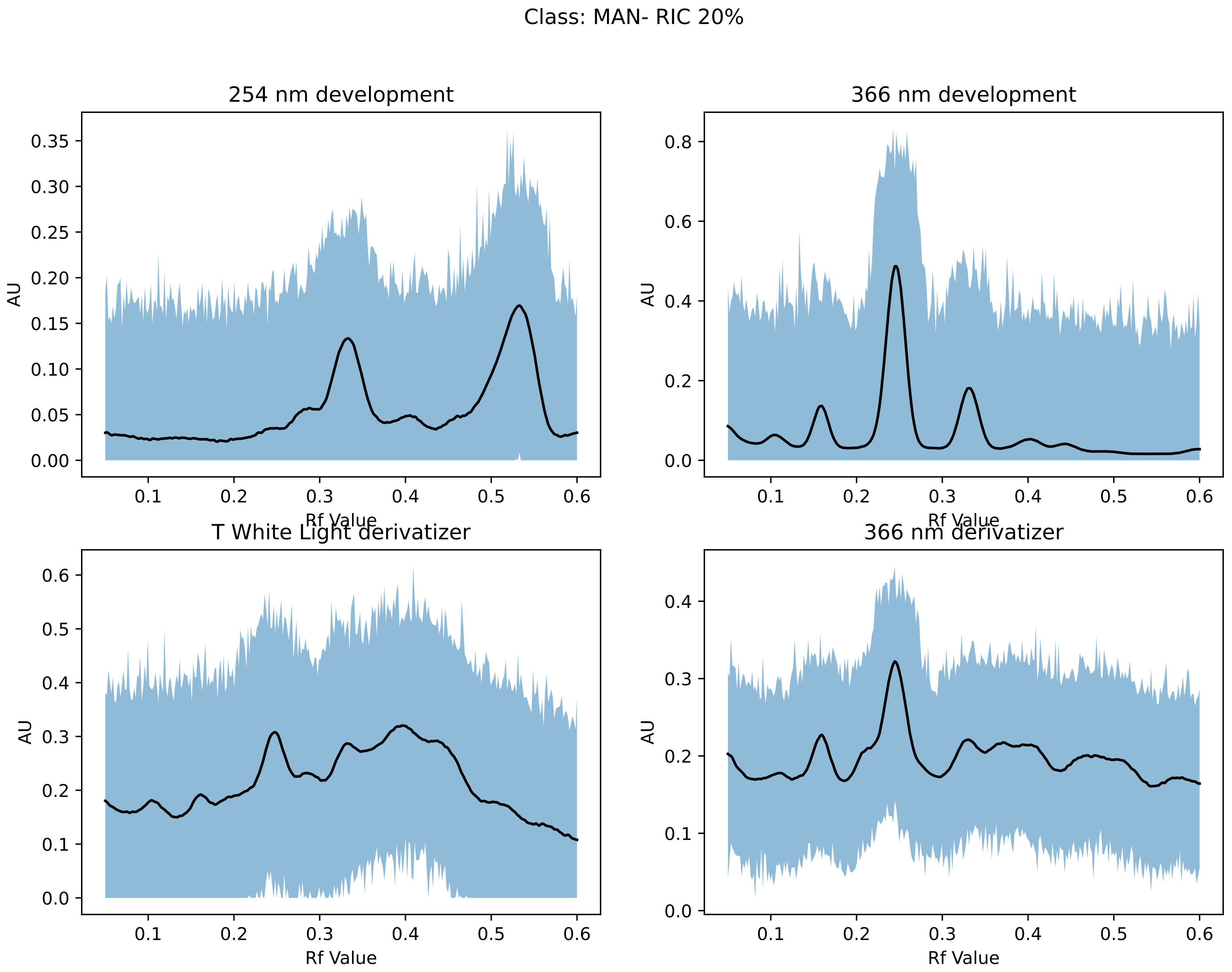

Class: MAN- RIC 20%
254 nm development
366 nm development
T White Light derivatizer
366 nm derivatizer
AU
Rf Value

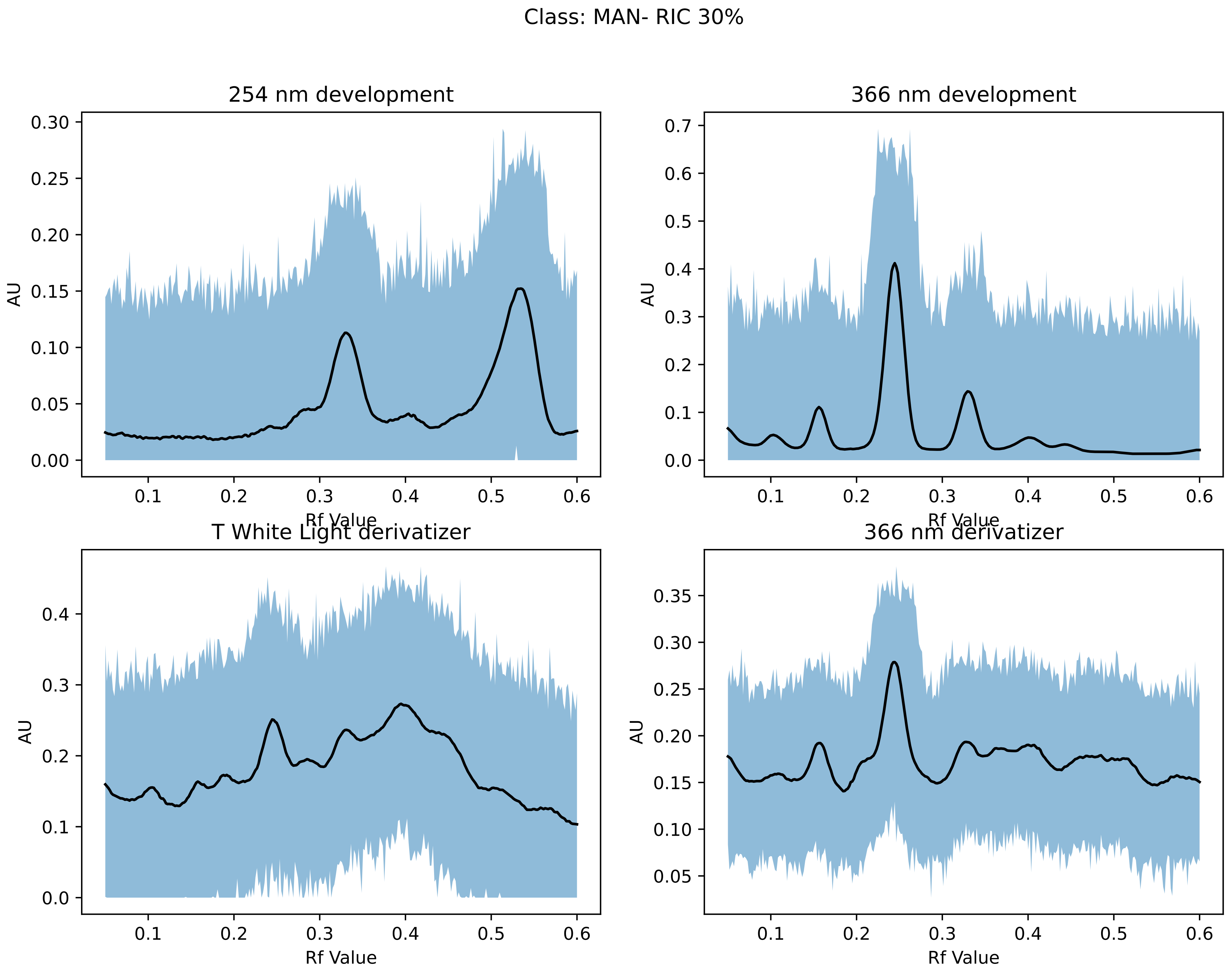
Class: MAN- RIC 30%
254 nm development
366 nm development
T White Light derivatizer
366 nm derivatizer
AU
Rf Value

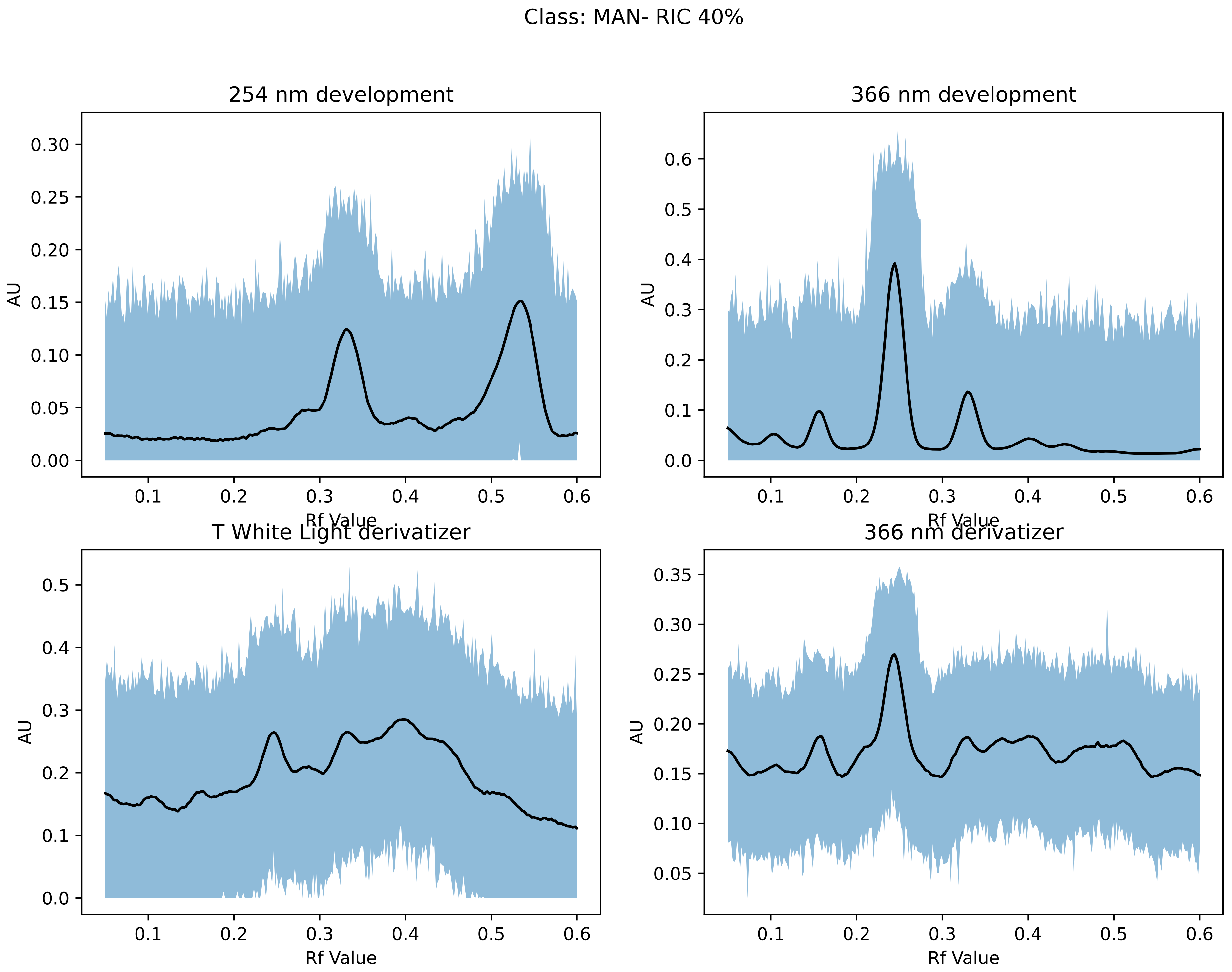
Class: MAN- RIC 40%
254 nm development
366 nm development
T White Light derivatizer
366 nm derivatizer
AU
Rf Value
0.00
0.05
0.10
0.15
0.20
0.25
0.30
0.0
0.1
0.2
0.3
0.4
0.5
0.6
0.35

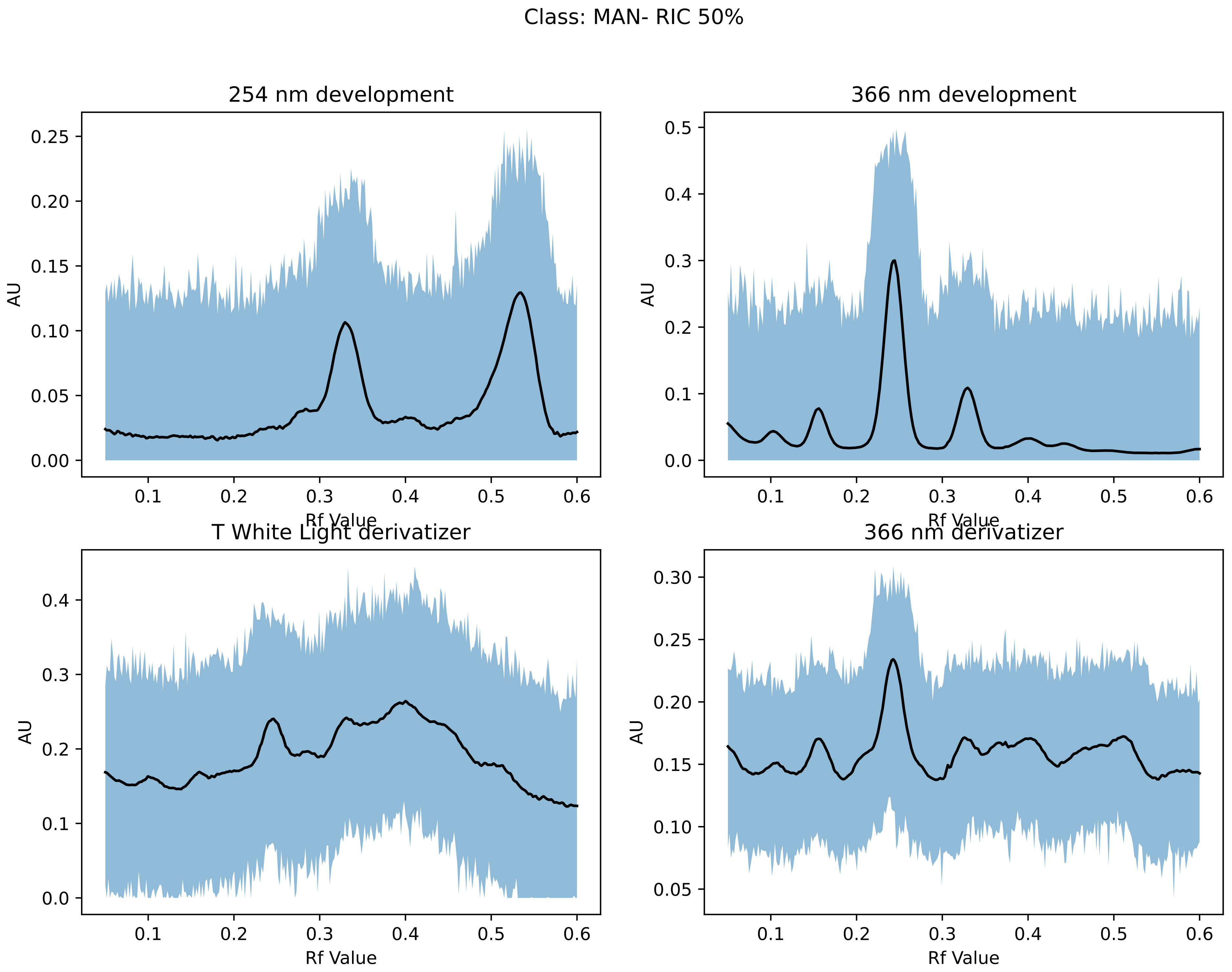

Class: MAN- RIC 50%
254 nm development
366 nm development
T White Light derivatizer
366 nm derivatizer
AU
Rf Value

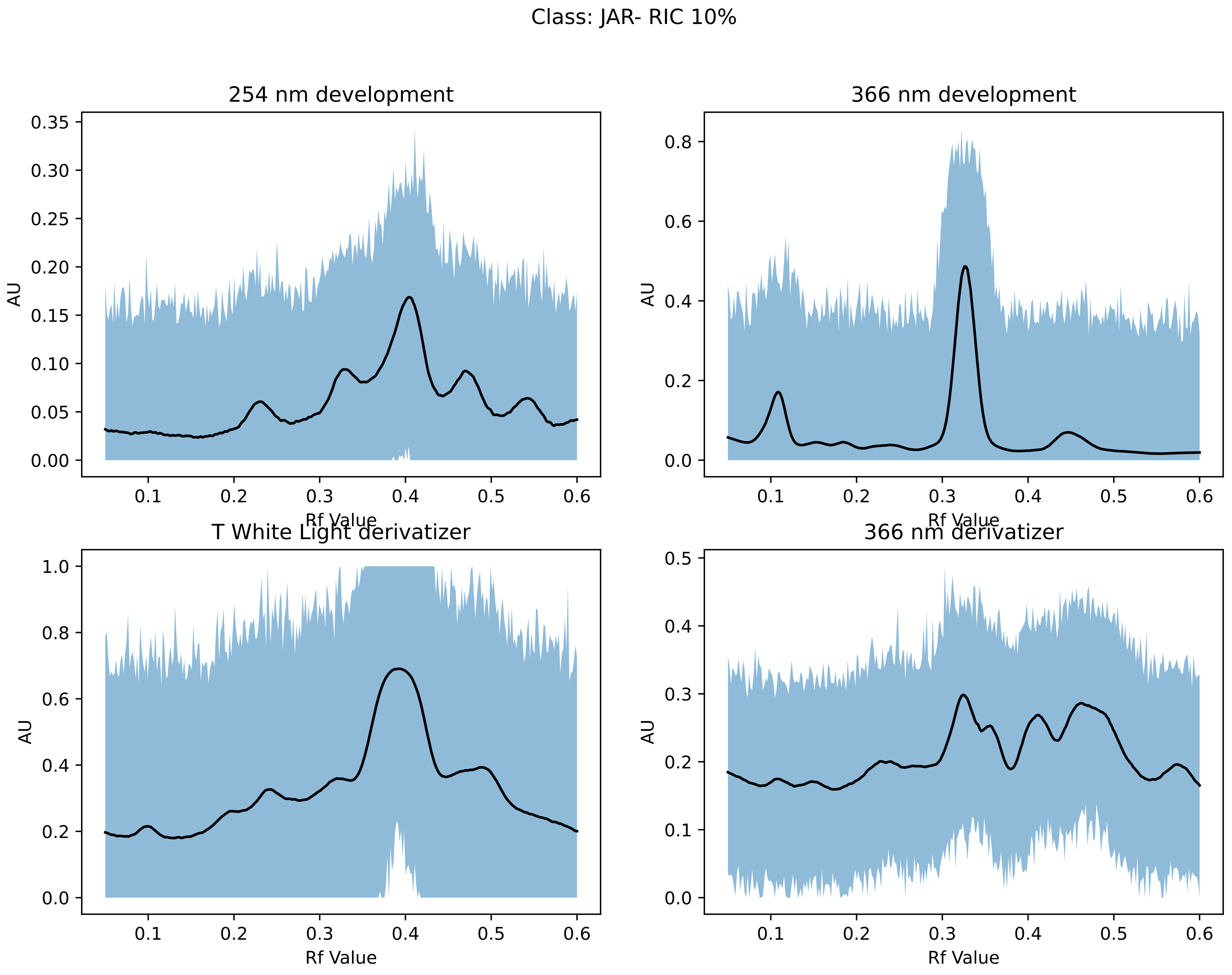

Class: JAR- RIC 10%
254 nm development
366 nm development
T White Light derivatizer
366 nm derivatizer
AU
Rf Value
0.35
0.30
0.25
0.20
0.15
0.10
0.05
0.00
0.8
0.6
0.4
0.2
0.0
1.0
0.5
0.3
0.1
0.1
0.2
0.3
0.4
0.5
0.6

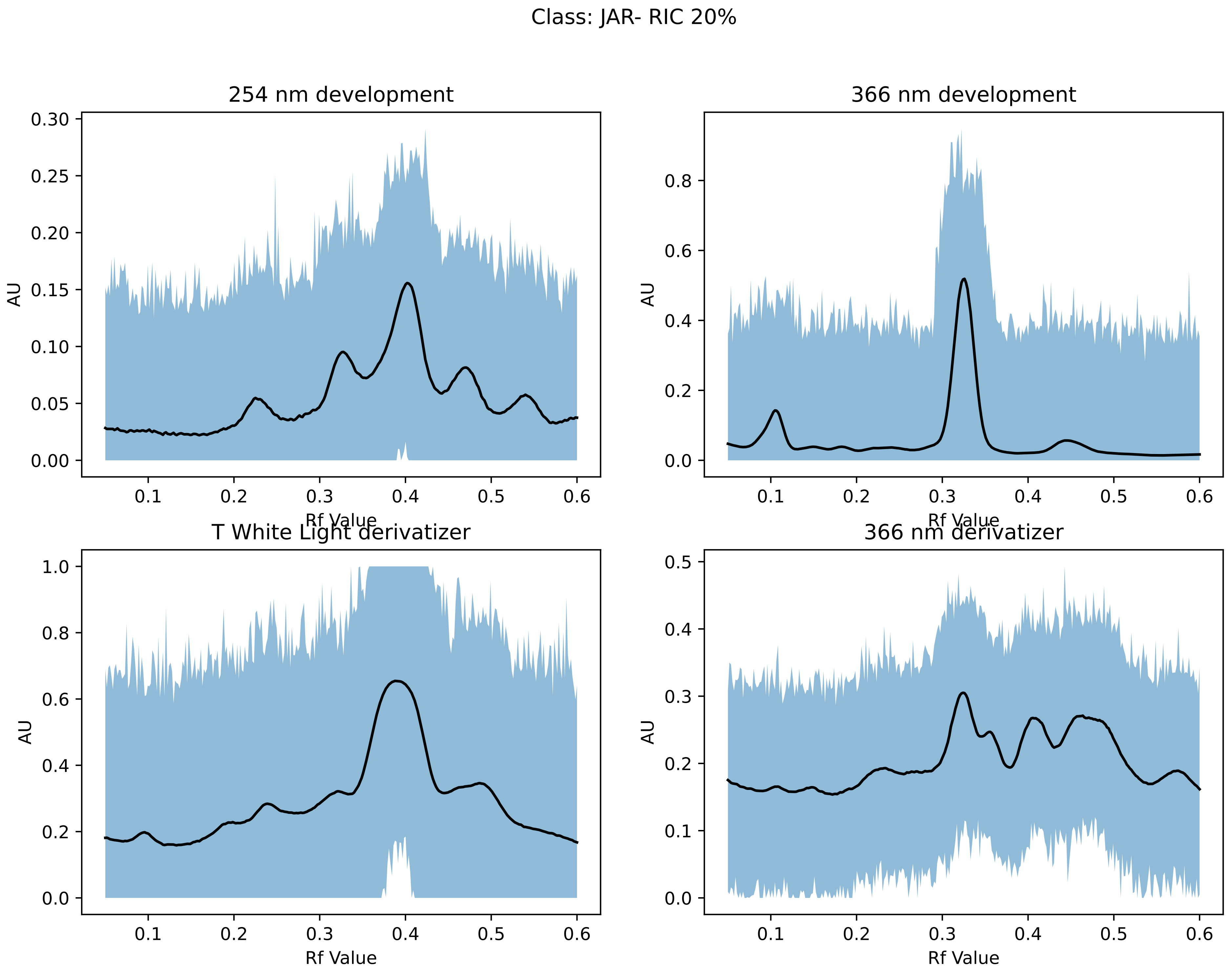
Class: JAR- RIC 20%
254 nm development
366 nm development
T White Light derivatizer
366 nm derivatizer
AU
Rf Value
0.00
0.05
0.10
0.15
0.20
0.25
0.30
0.0
0.2
0.4
0.6
0.8
1.0
0.1
0.3
0.5
0.6

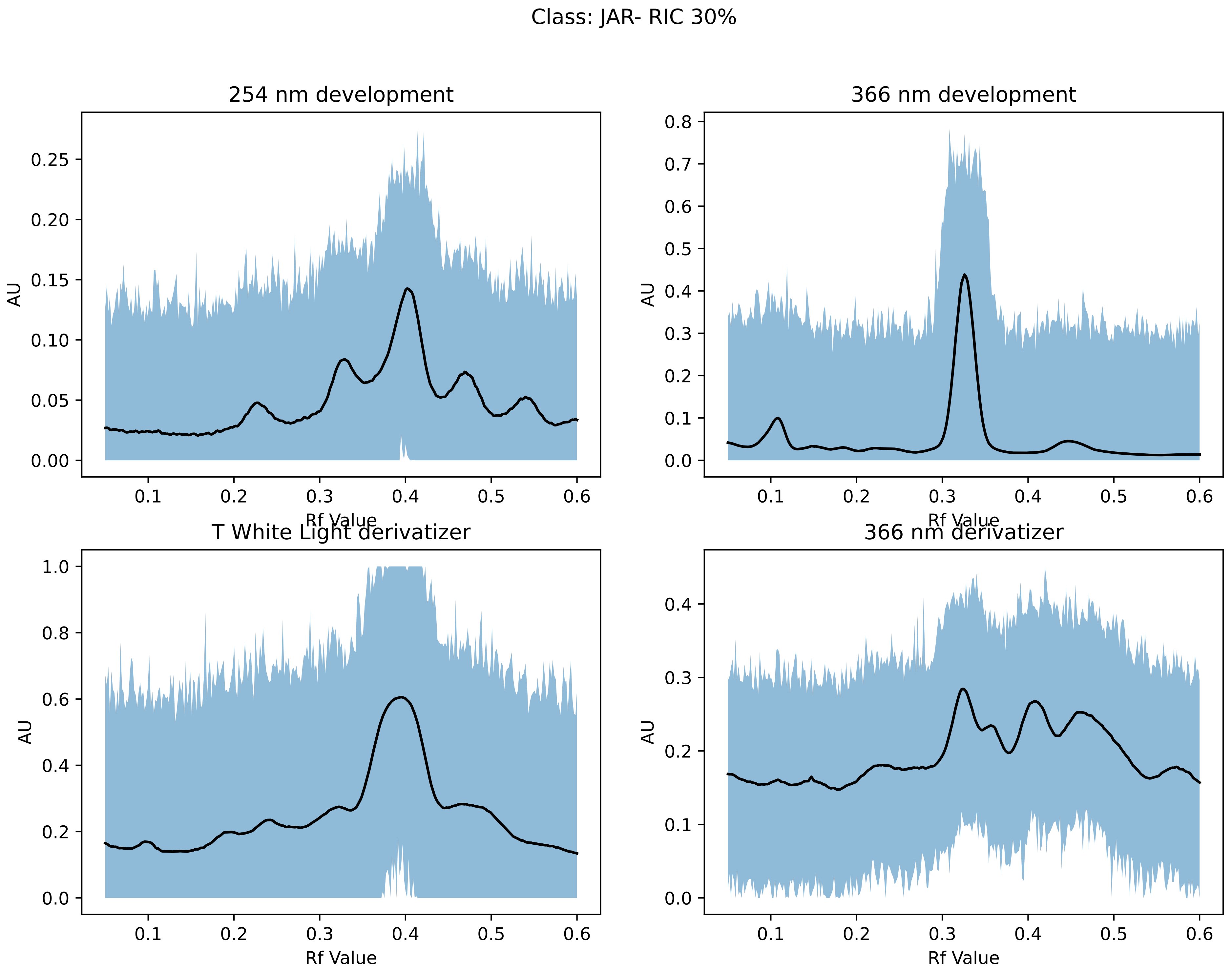
Class: JAR- RIC 30%
254 nm development
366 nm development
T White Light derivatizer
366 nm derivatizer
AU
Rf Value

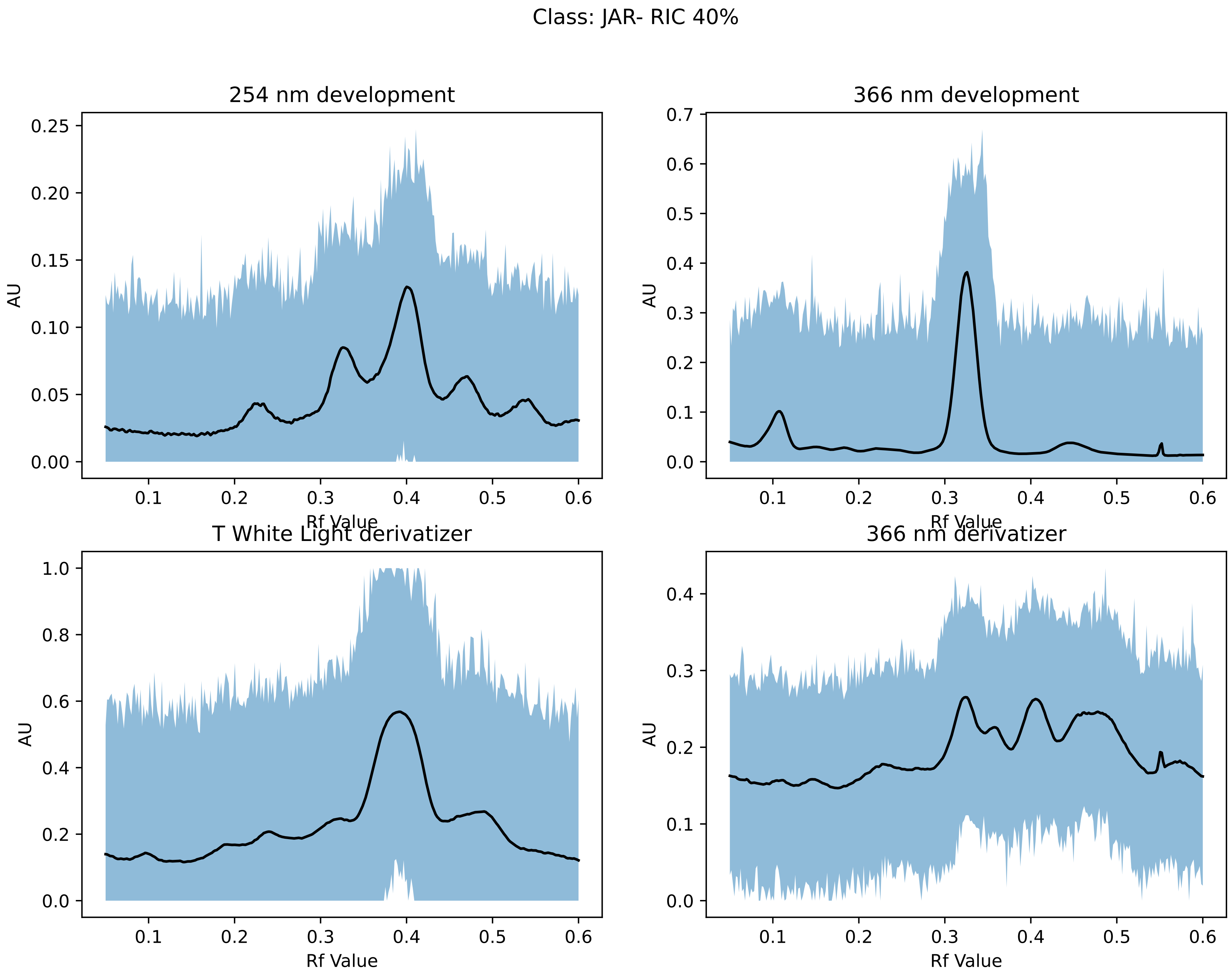

Class: JAR- RIC 40%
254 nm development
366 nm development
T White Light derivatizer
366 nm derivatizer
AU
Rf Value
0.00
0.05
0.10
0.15
0.20
0.25
0.0
0.1
0.2
0.3
0.4
0.5
0.6
0.7
0.8
1.0

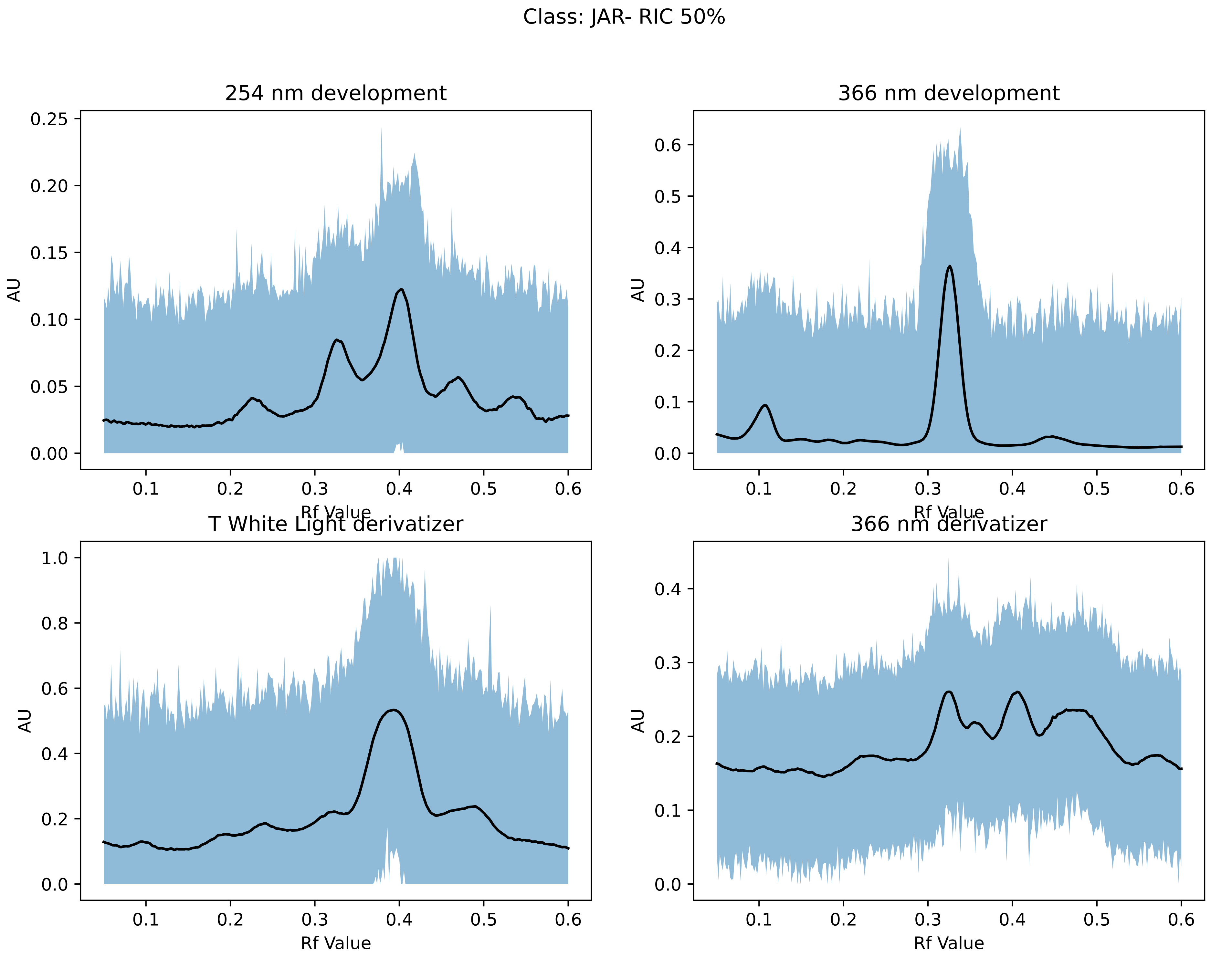

Class: JAR- RIC 50%
254 nm development
366 nm development
T White Light derivatizer
366 nm derivatizer
AU
Rf Value
0.00
0.05
0.10
0.15
0.20
0.25
0.0
0.1
0.2
0.3
0.4
0.5
0.6
0.8
1.0
