# Supplementary material for: Detection of syrup adulterants in manuka and jarrah honey using HPTLC-multivariate data analysis
Source: PeerJ. 2021 Sep 22;9:e12186. doi: 10.7717/peerj.12186 (PMC8464195; doi:10.7717/peerj.12186)
Supplement: Supplemental Information 2 [file peerj-09-12186-s002.pdf]

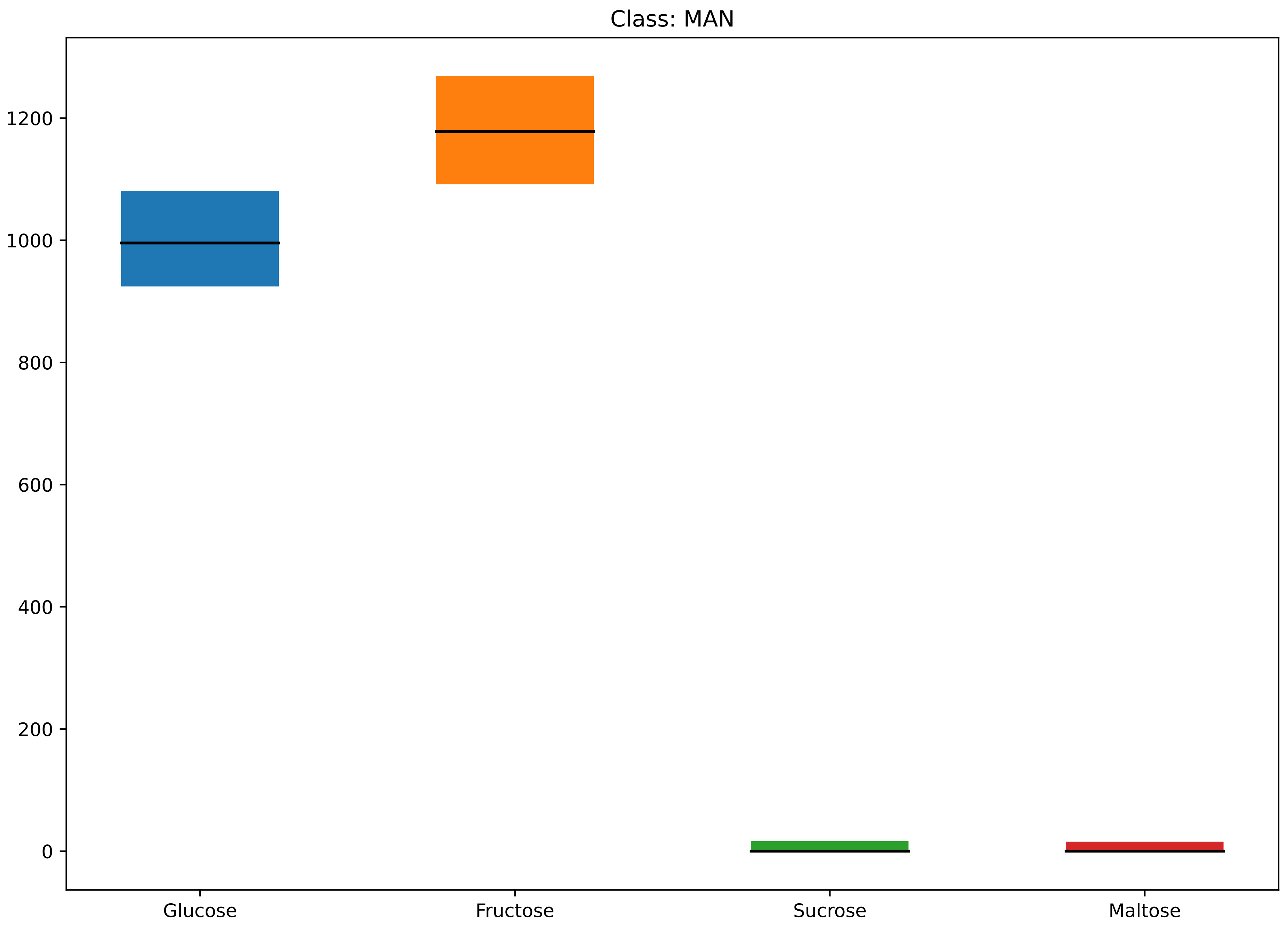

Class: MAN
1200
1000
800
600
400
200
0
Glucose
Fructose
Sucrose
Maltose

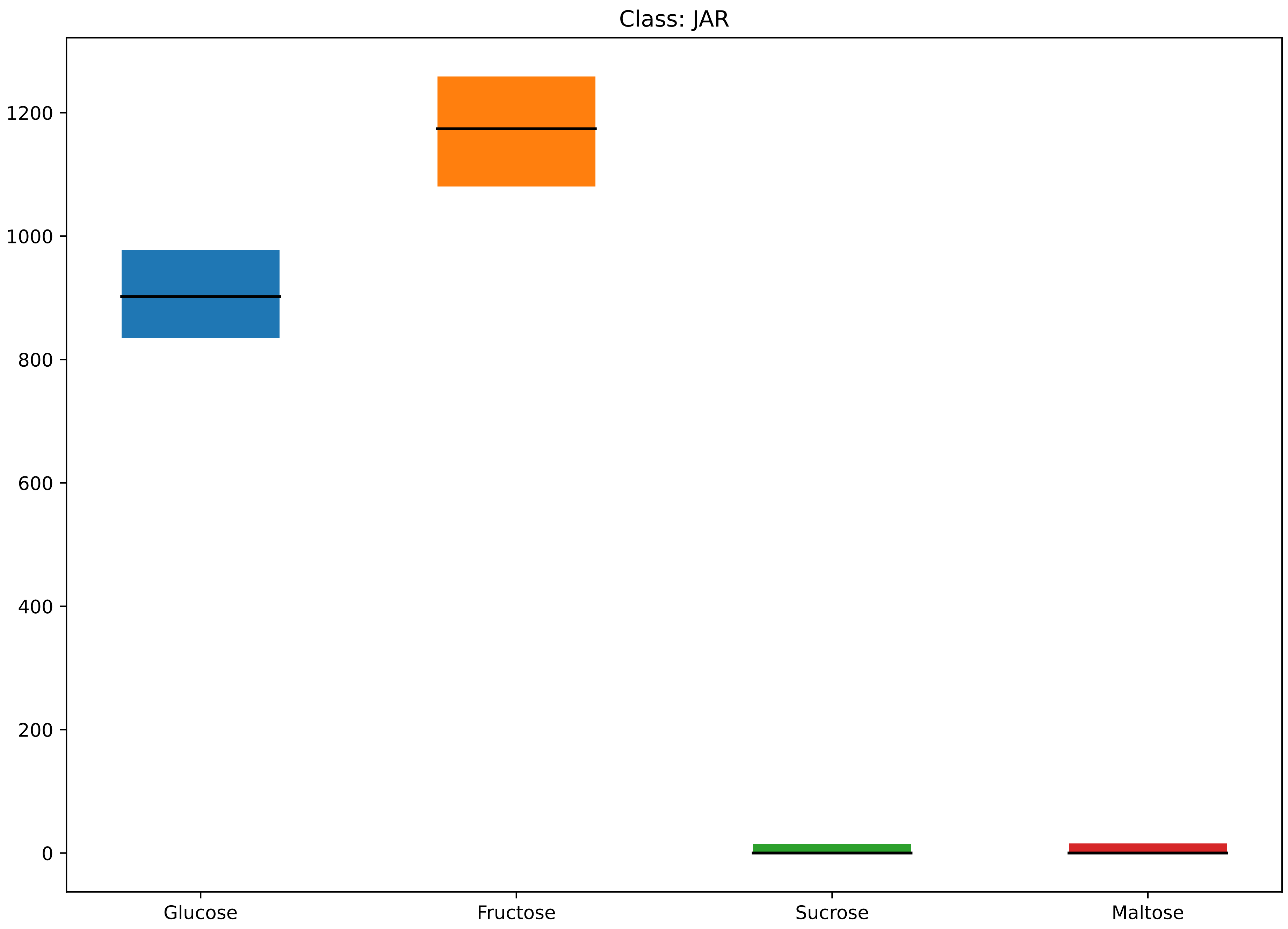

Class: JAR
1200
1000
800
600
400
200
0
Glucose
Fructose
Sucrose
Maltose

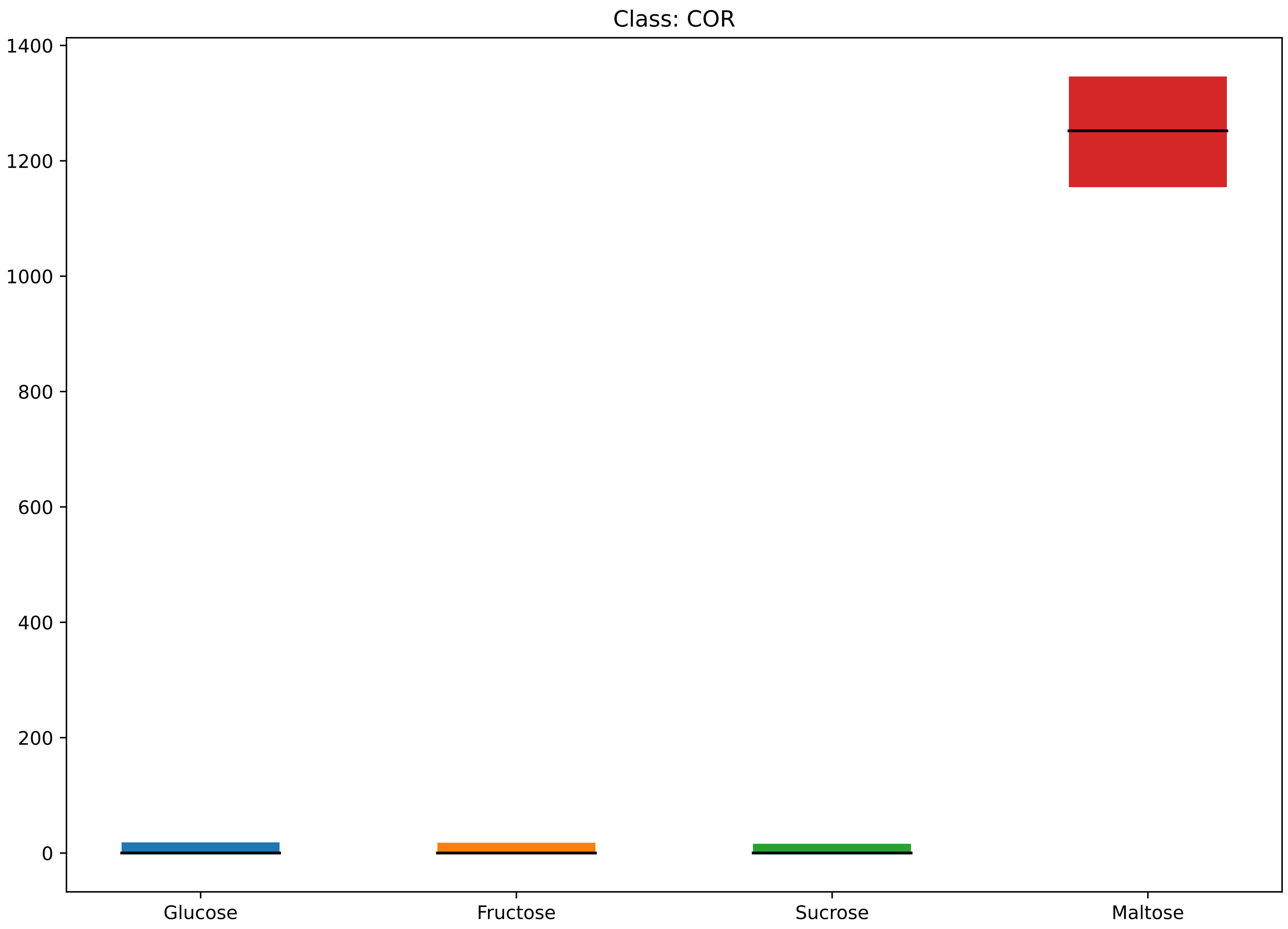
Class: COR
1400
1200
1000
800
600
400
200
0
Glucose
Fructose
Sucrose
Maltose

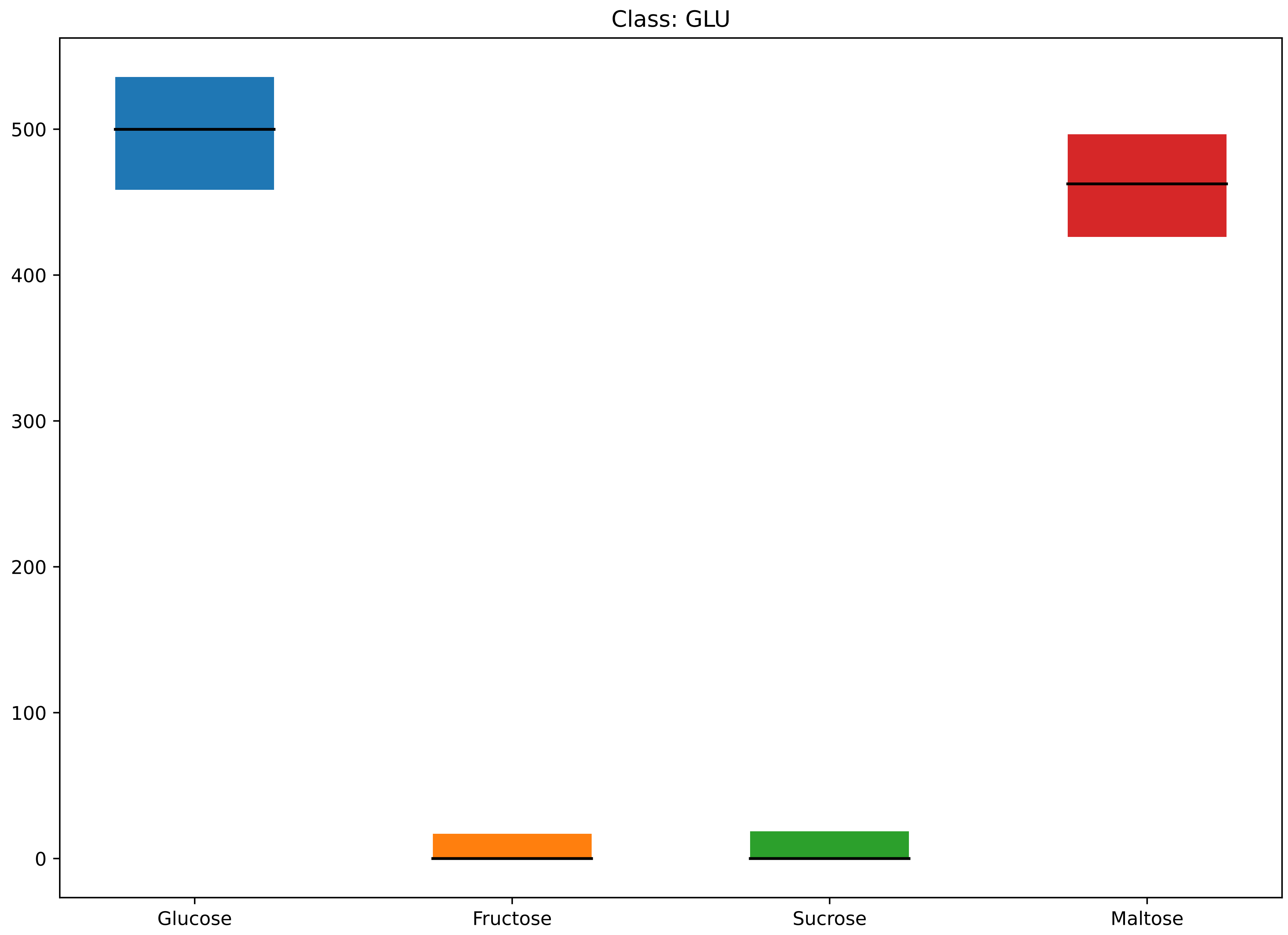
Class: GLU
500
400
300
200
100
0
Glucose
Fructose
Sucrose
Maltose

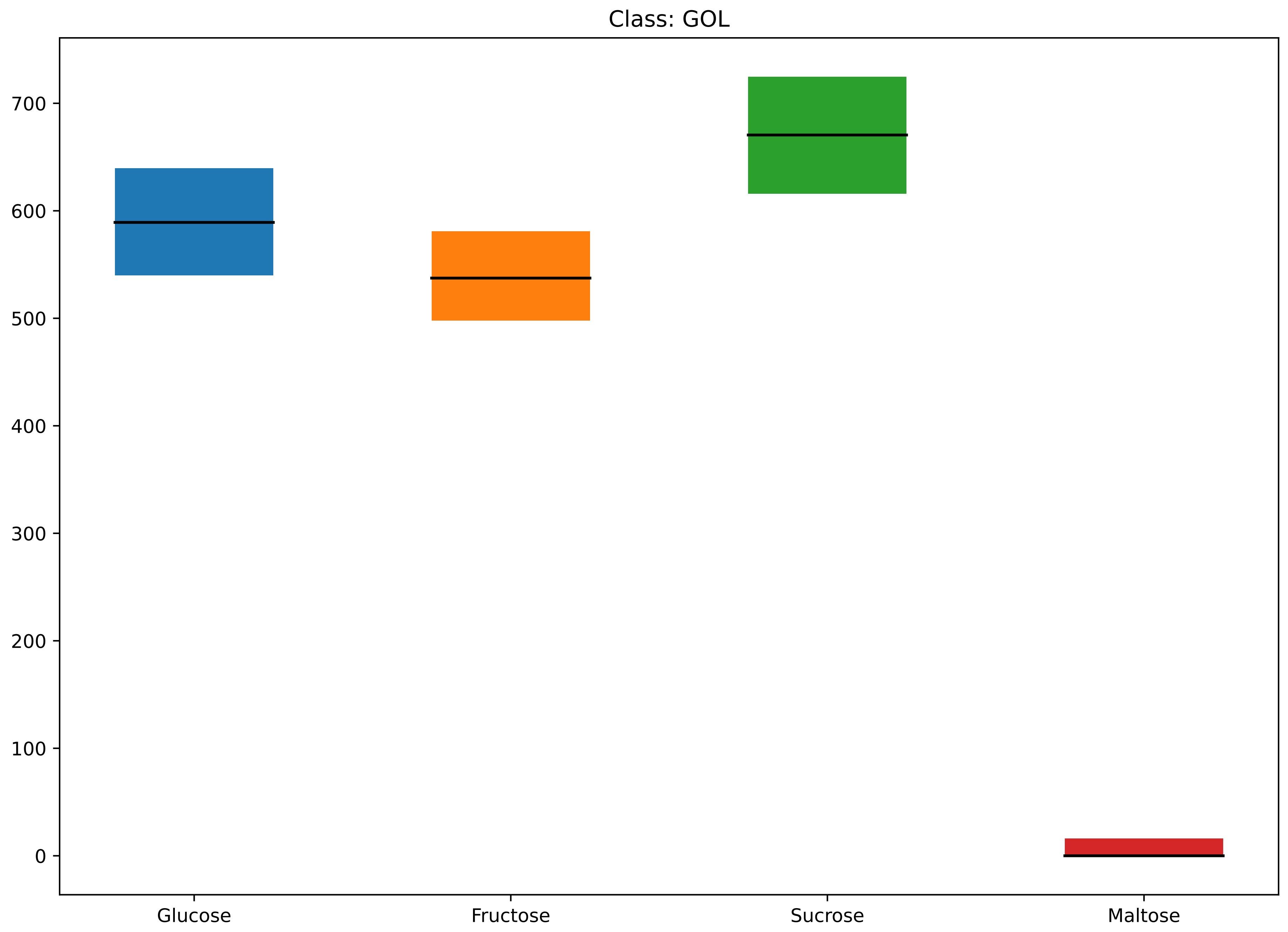

Class: GOL
700
600
500
400
300
200
100
0
Glucose
Fructose
Sucrose
Maltose

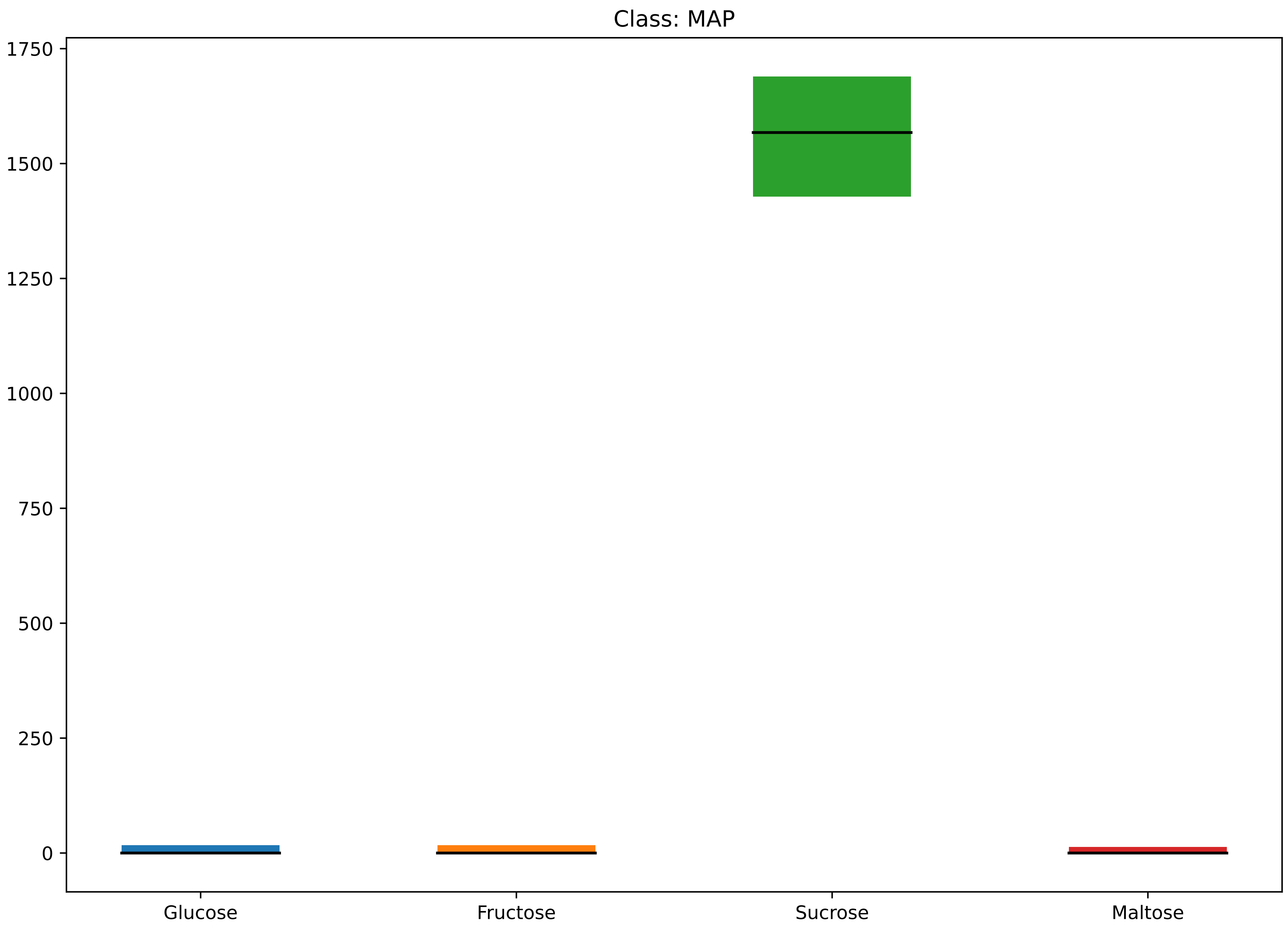

Class: MAP
1750
1500
1250
1000
750
500
250
0
Glucose
Fructose
Sucrose
Maltose

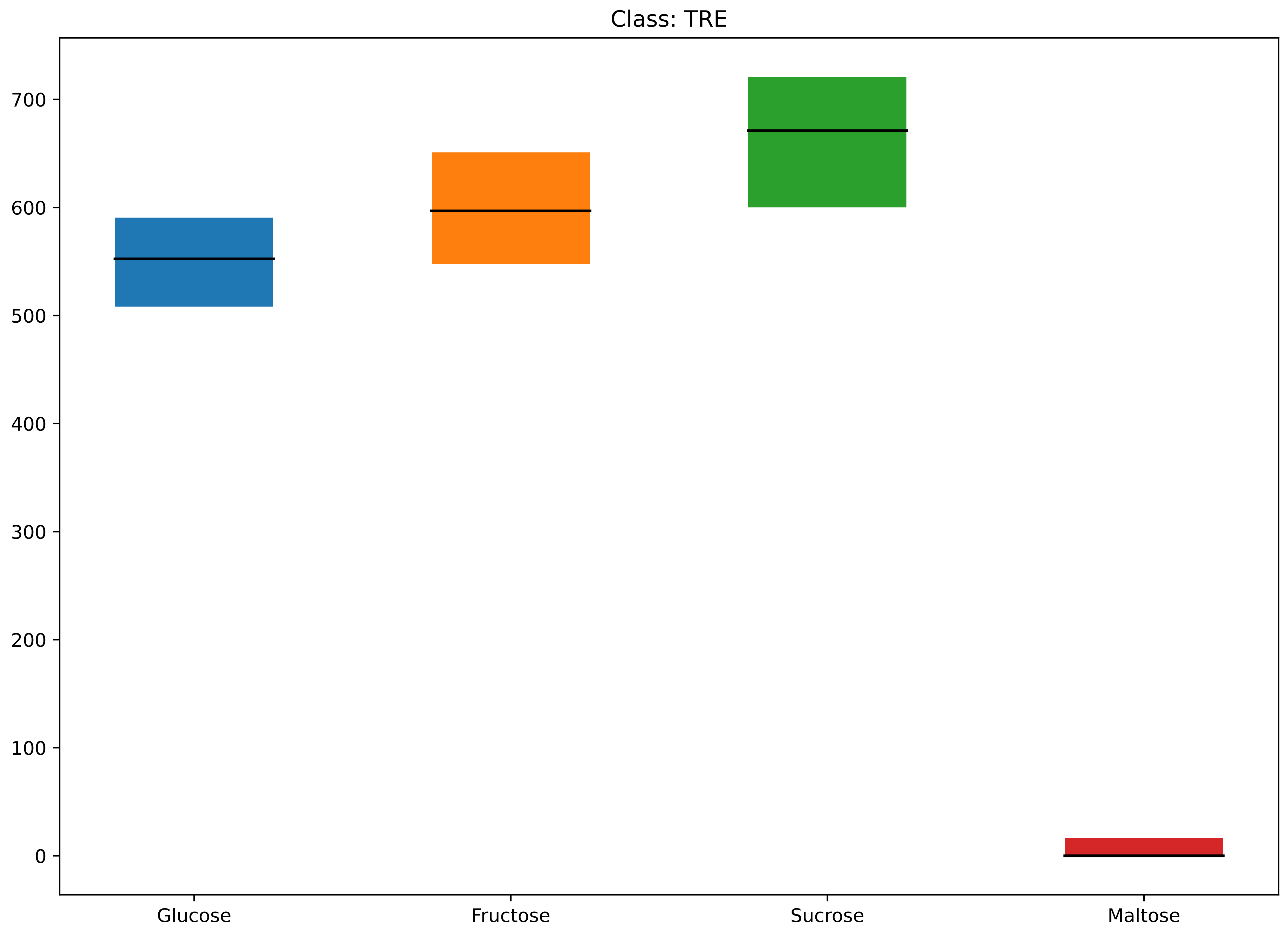

Class: TRE
700
600
500
400
300
200
100
0
Glucose
Fructose
Sucrose
Maltose

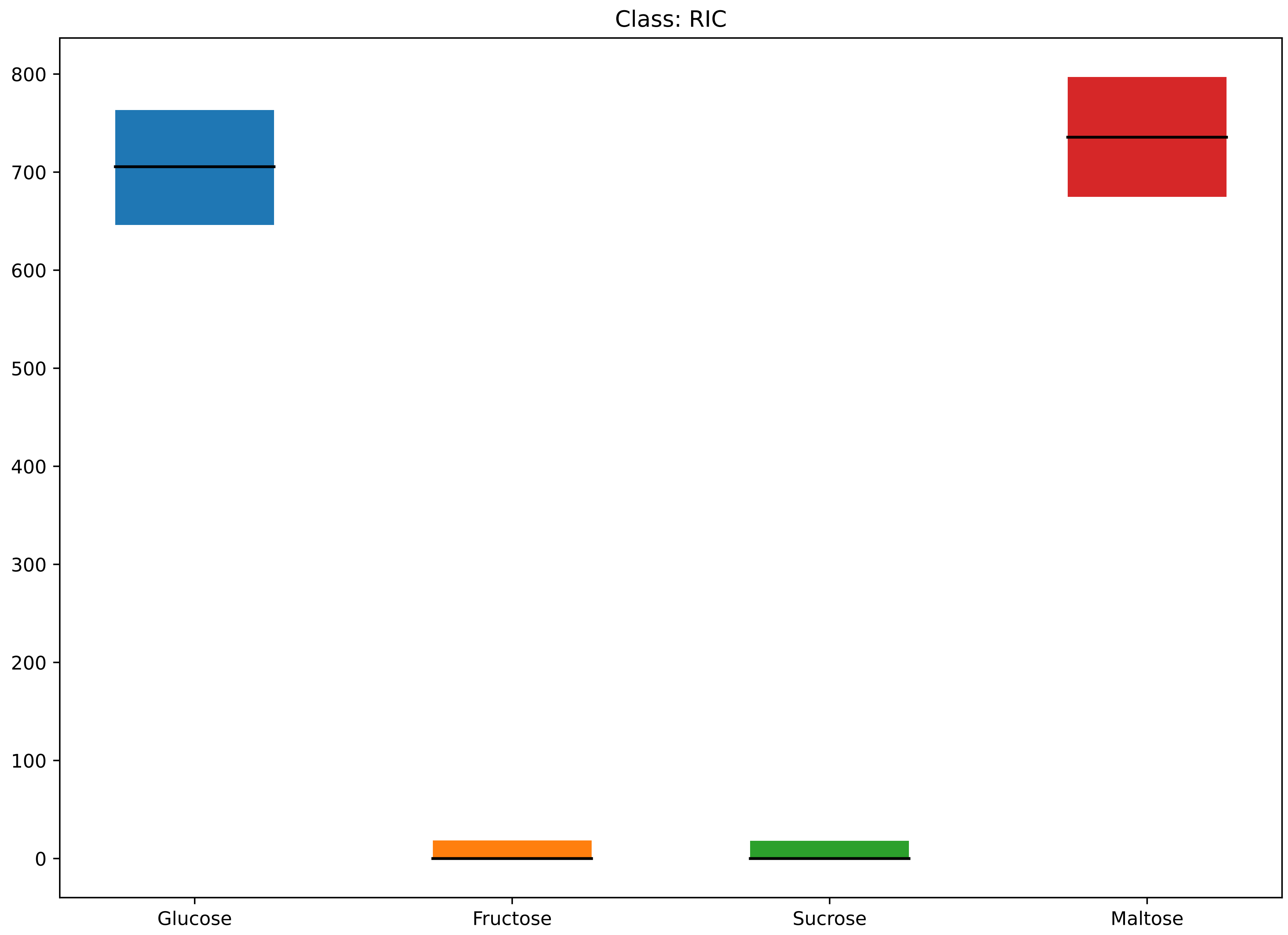
Class: RIC
800
700
600
500
400
300
200
100
0
Glucose
Fructose
Sucrose
Maltose

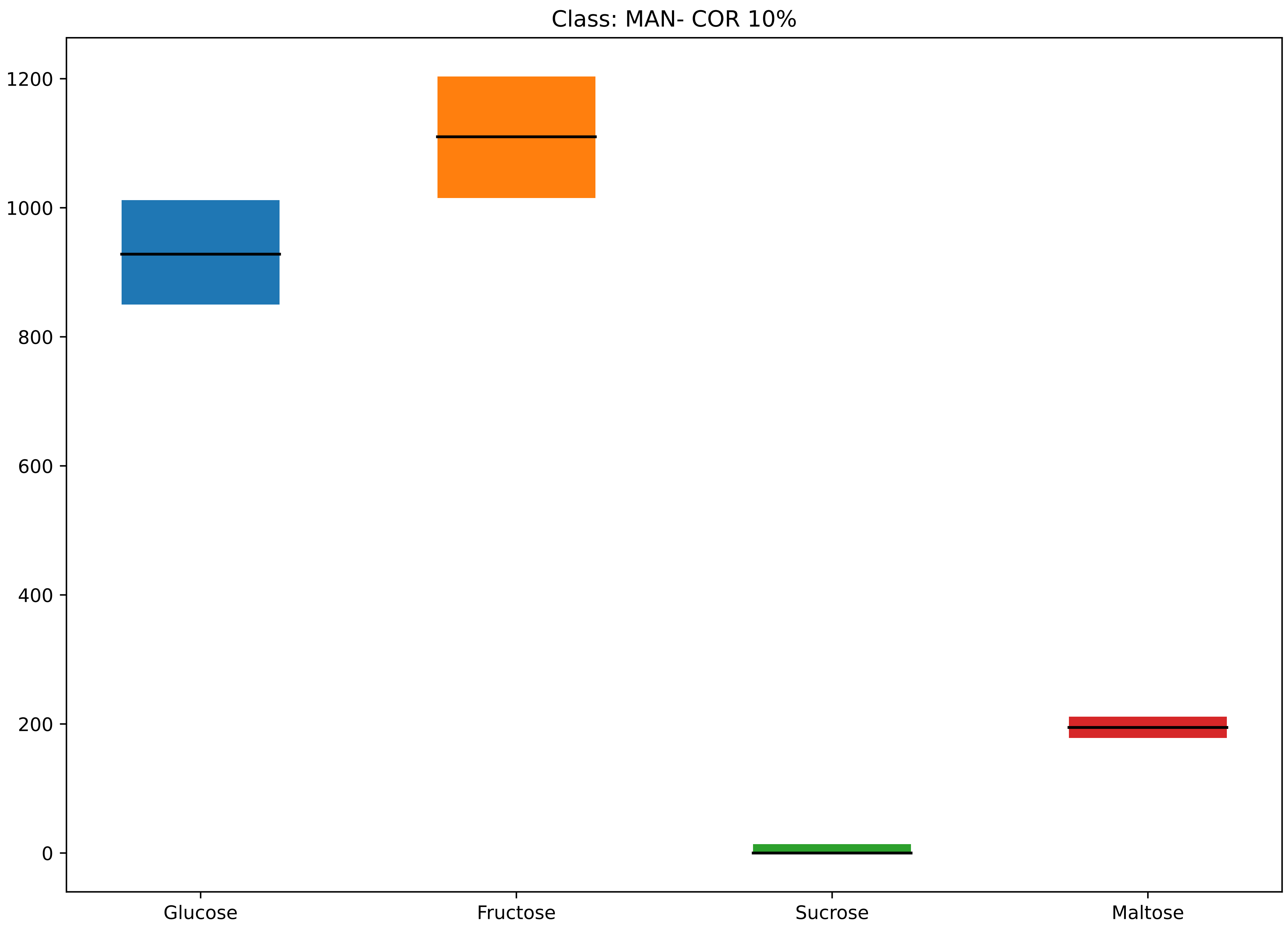

Class: MAN- COR 10%
1200
1000
800
600
400
200
0
Glucose
Fructose
Sucrose
Maltose

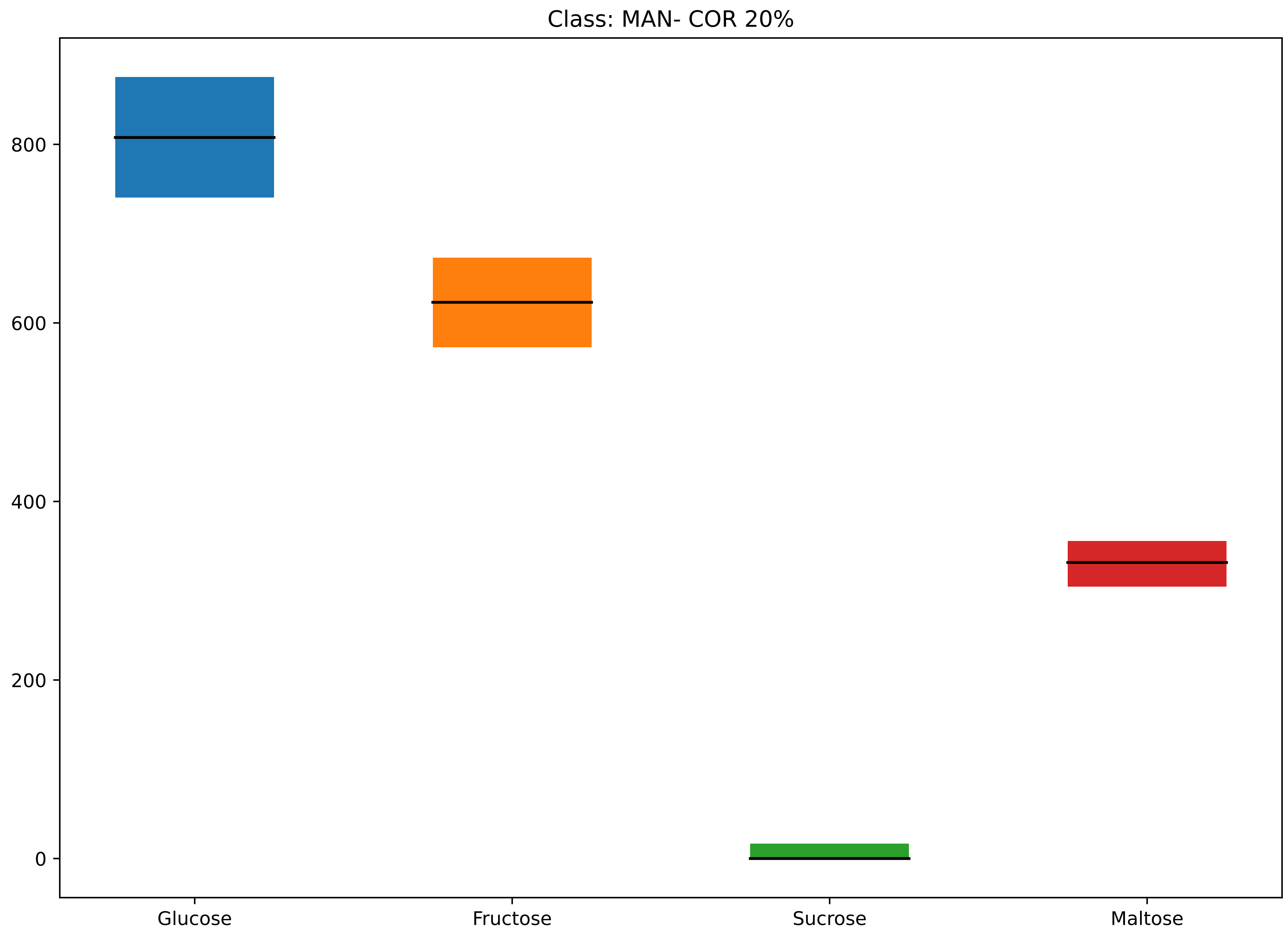
Class: MAN- COR 20%
800
600
400
200
0
Glucose
Fructose
Sucrose
Maltose

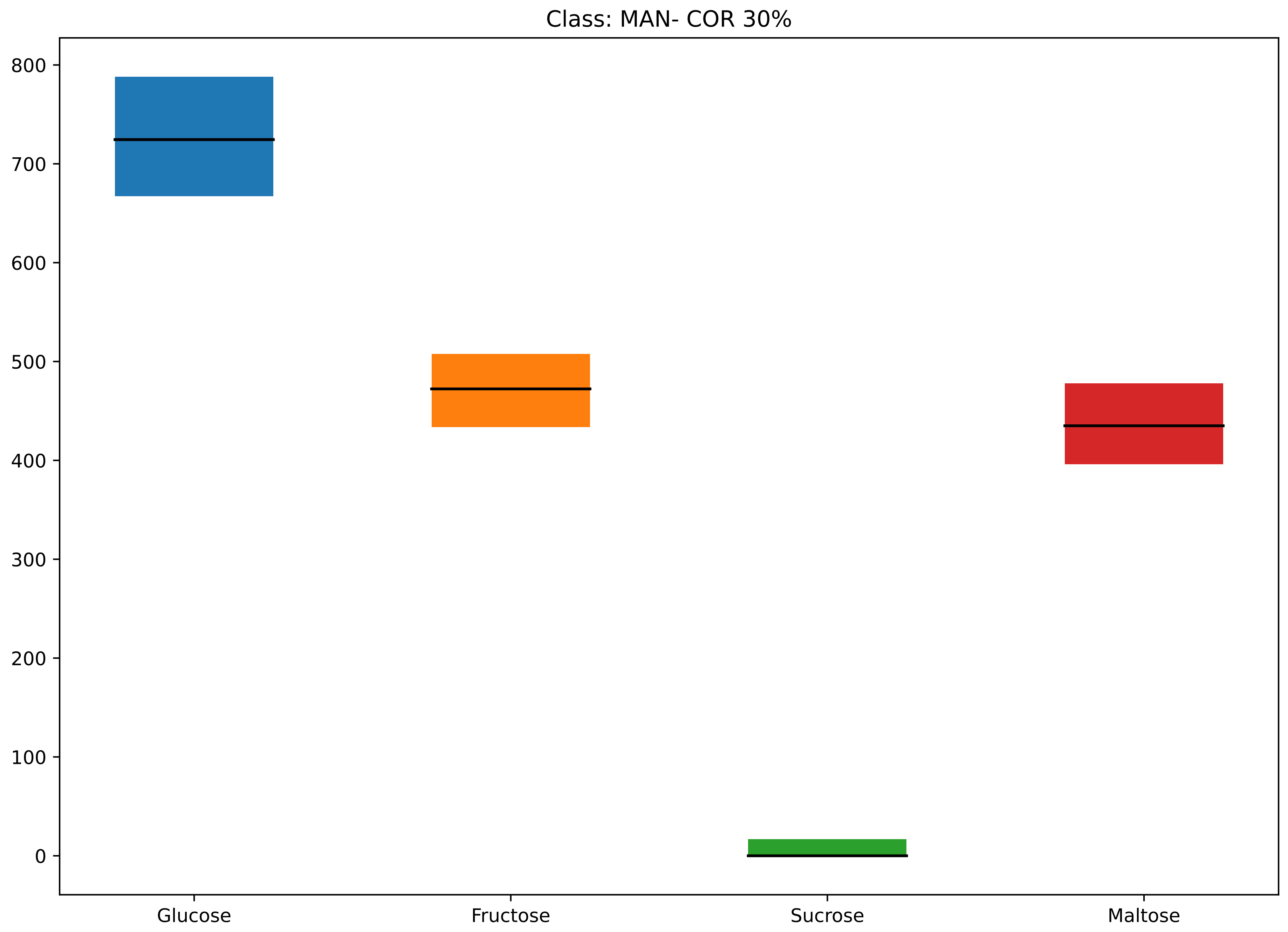

Class: MAN- COR 30%
800
700
600
500
400
300
200
100
0
Glucose
Fructose
Sucrose
Maltose

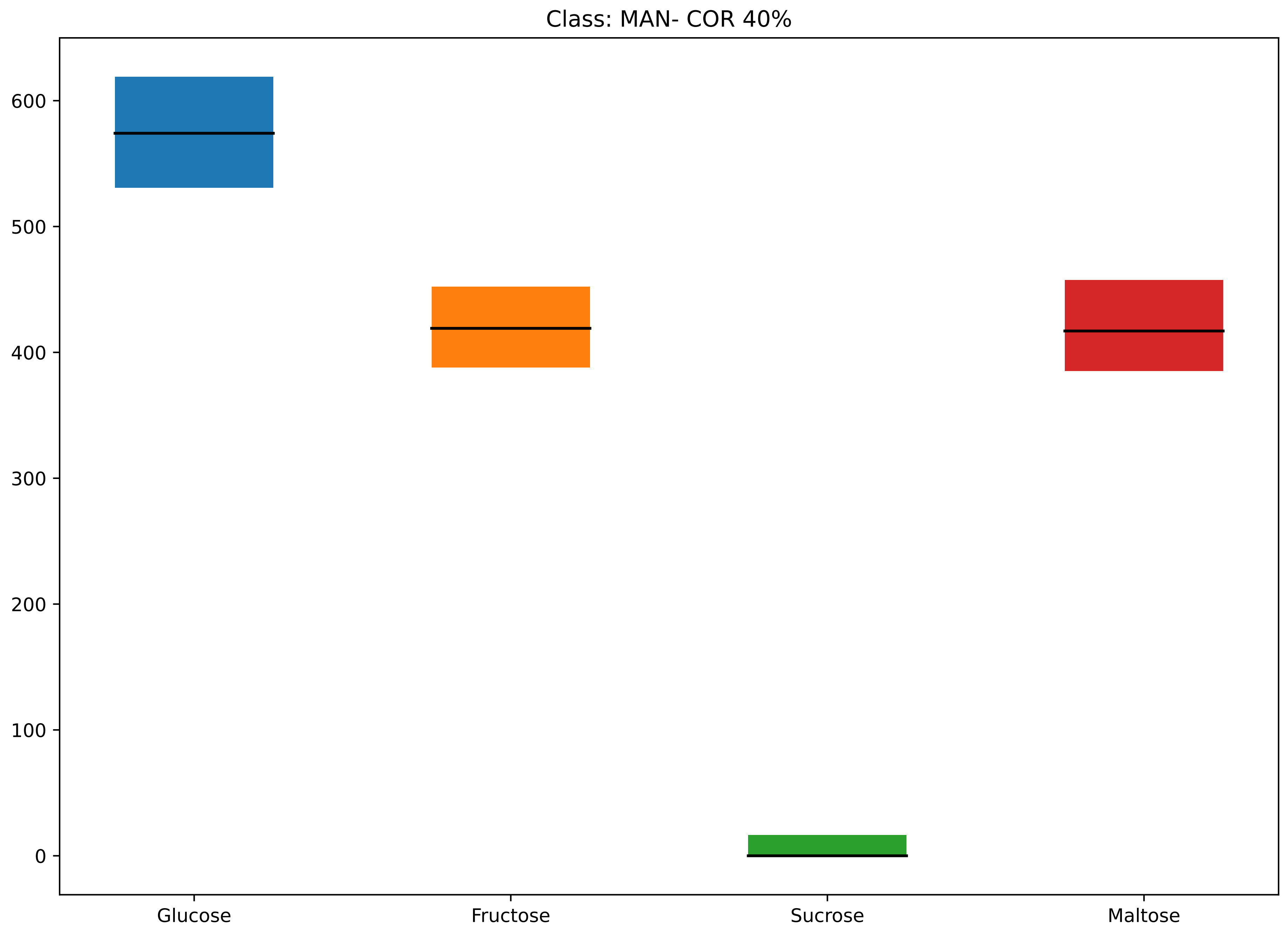
Class: MAN- COR 40%
600
500
400
300
200
100
0
Glucose
Fructose
Sucrose
Maltose

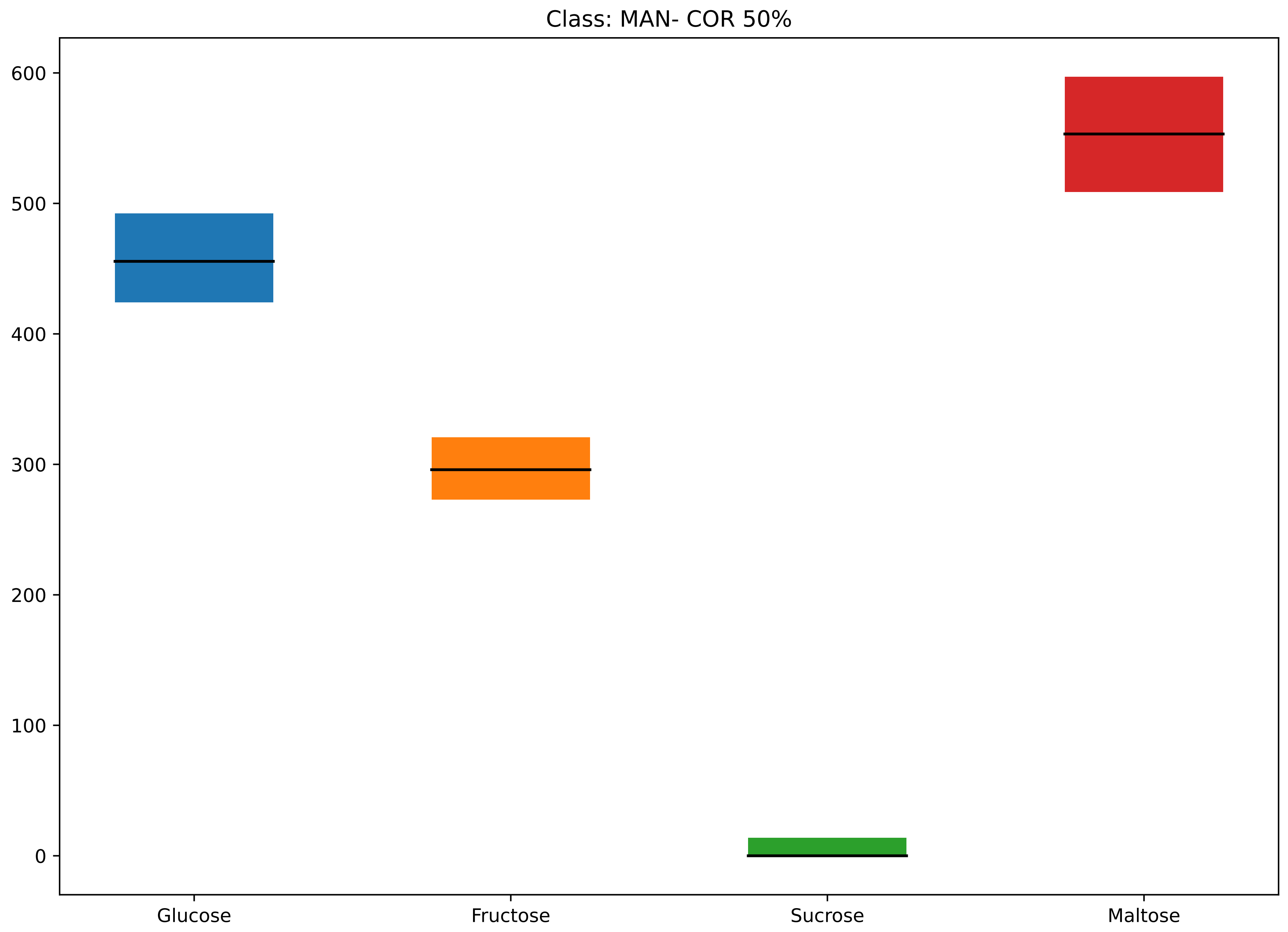

Class: MAN- COR 50%
600
500
400
300
200
100
0
Glucose
Fructose
Sucrose
Maltose

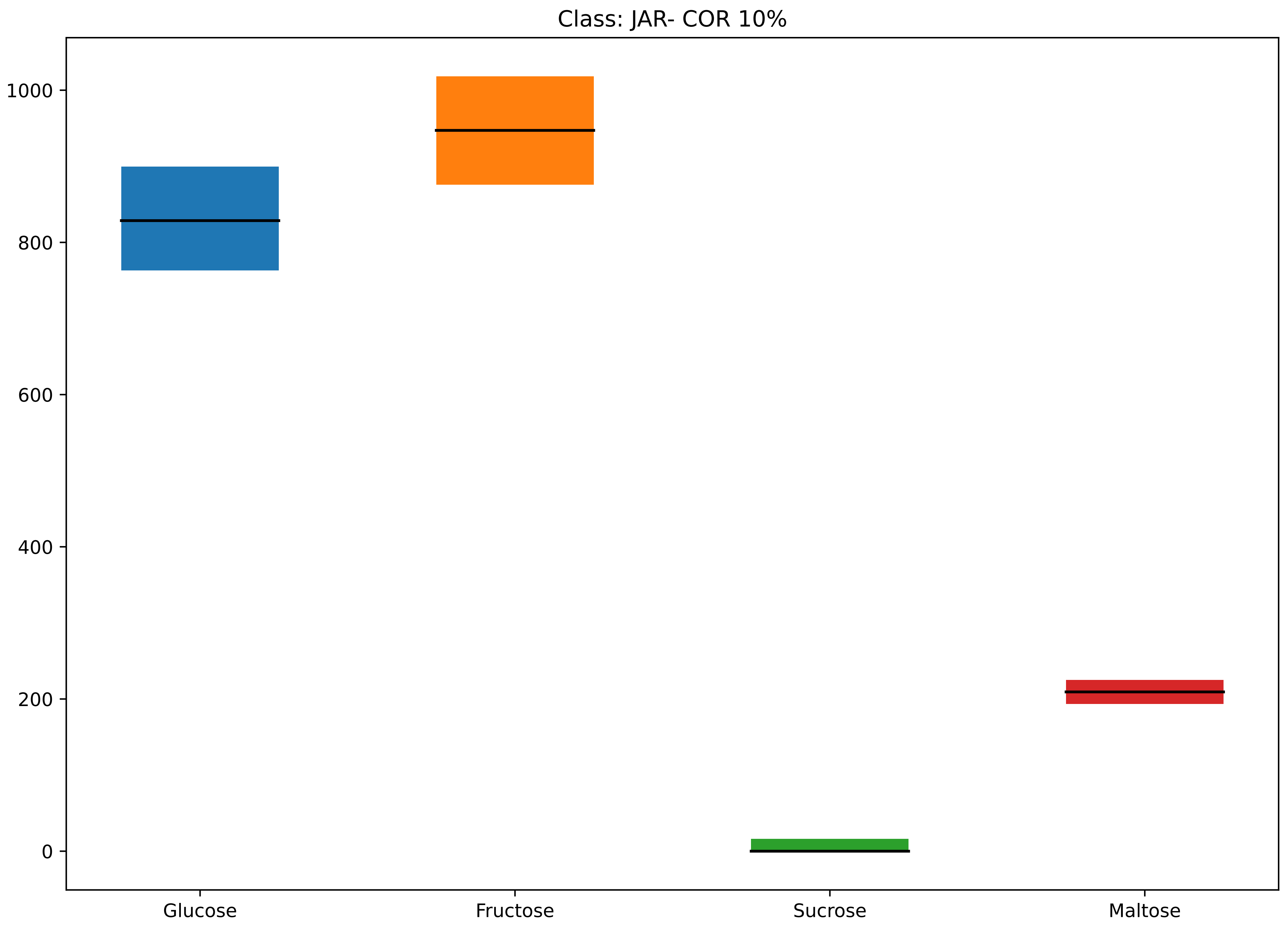

Class: JAR- COR 10%
1000
800
600
400
200
0
Glucose
Fructose
Sucrose
Maltose

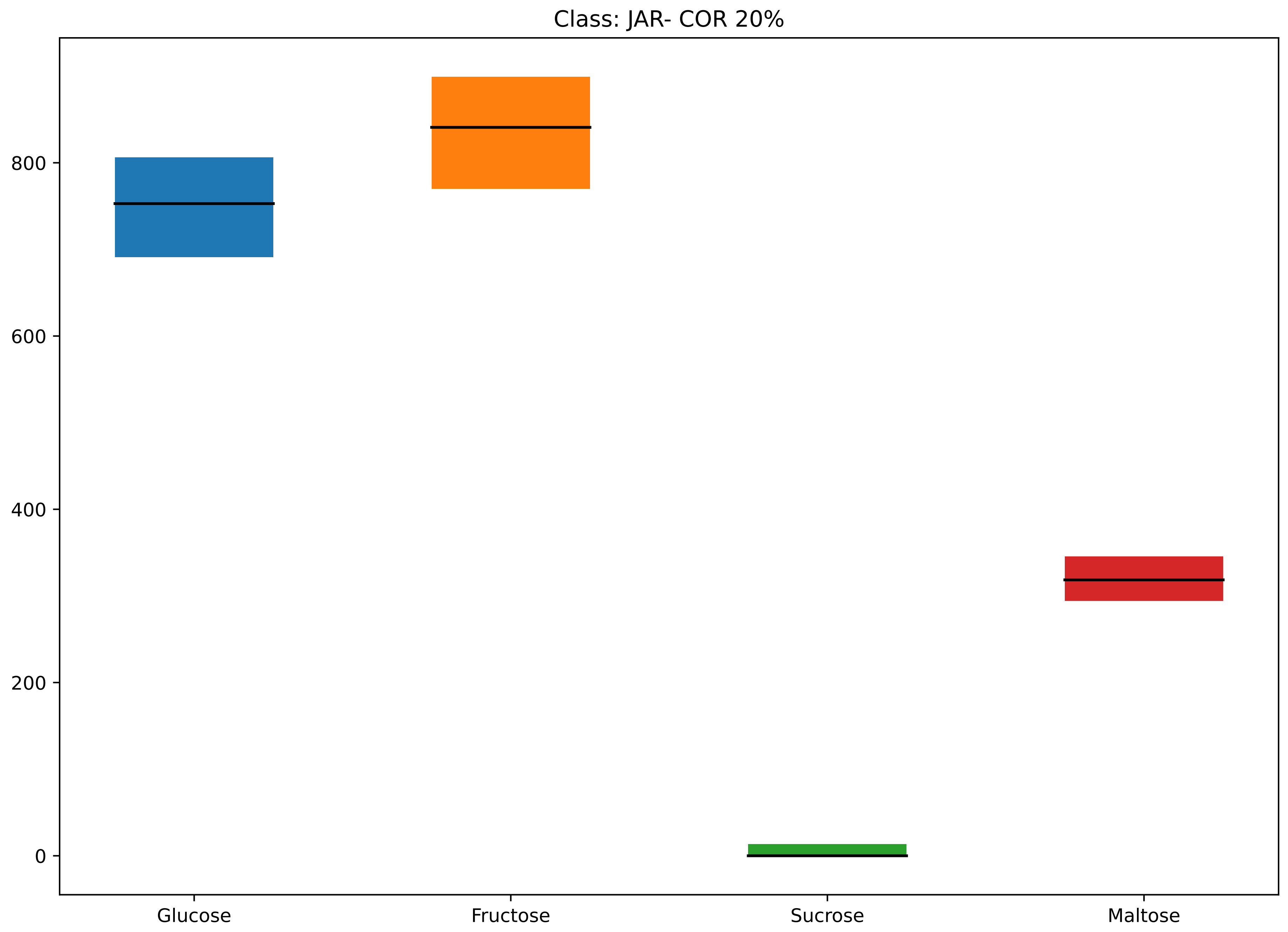

Class: JAR- COR 20%
800
600
400
200
0
Glucose
Fructose
Sucrose
Maltose

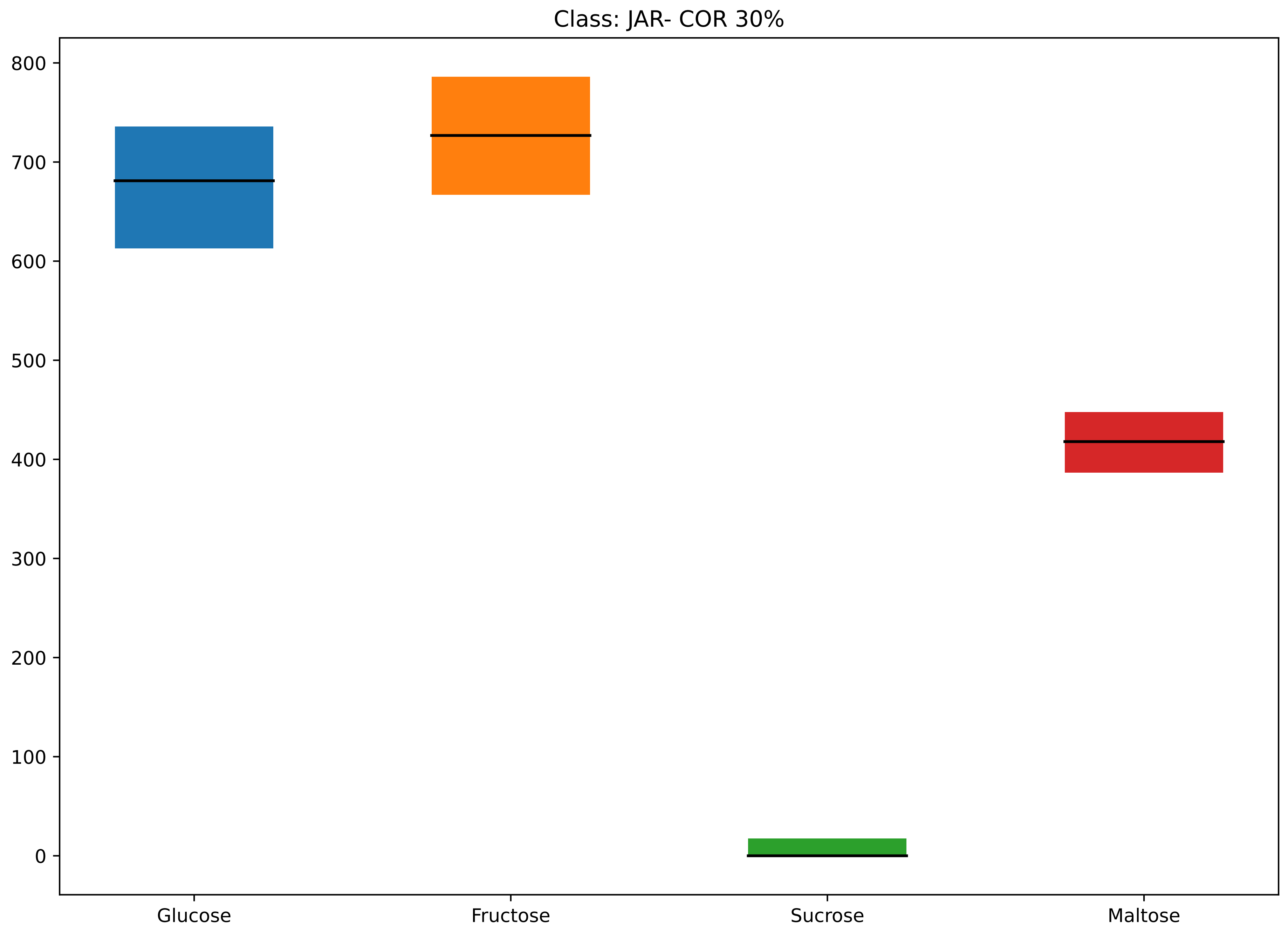

Class: JAR- COR 30%
800
700
600
500
400
300
200
100
0
Glucose
Fructose
Sucrose
Maltose

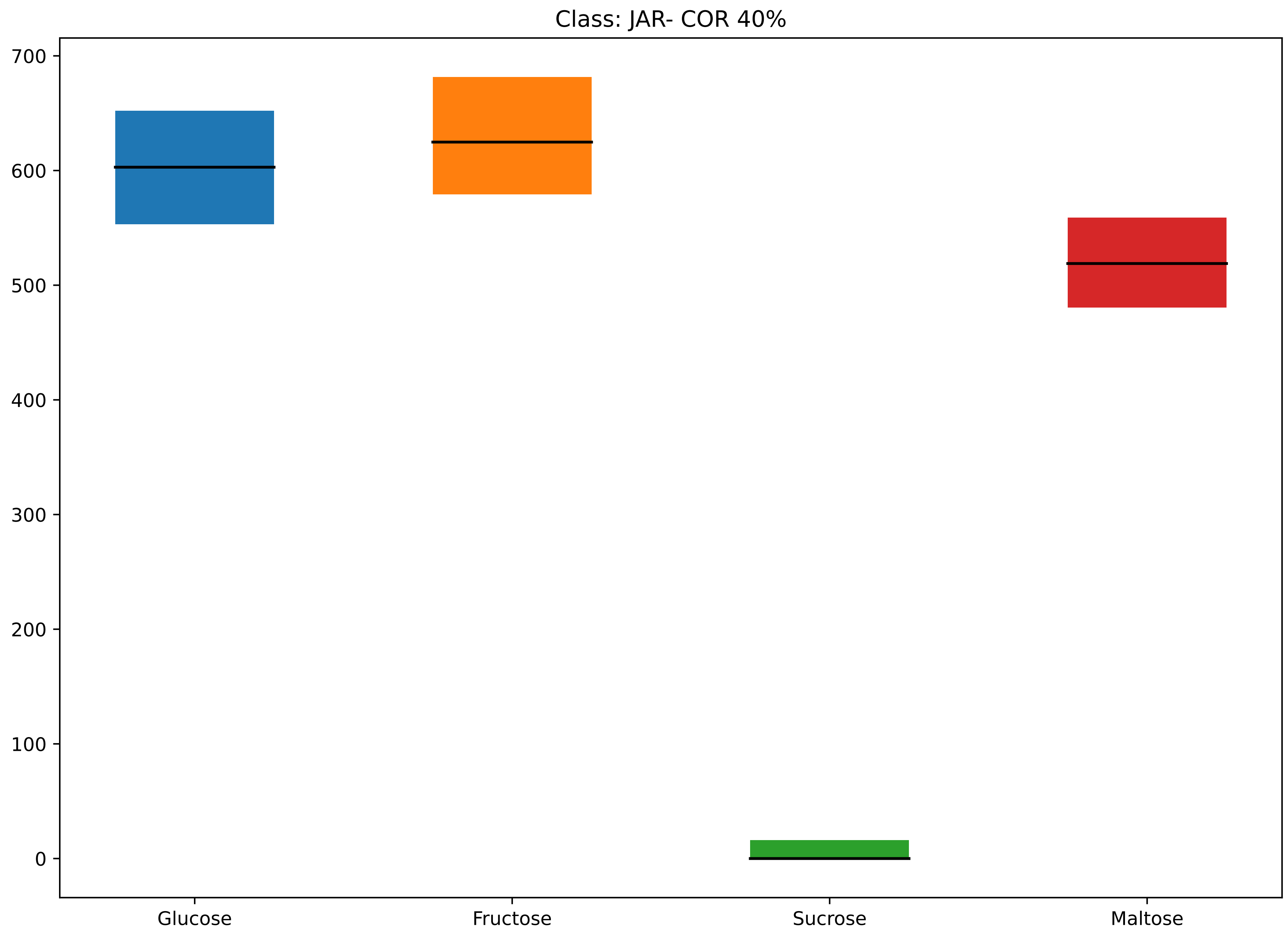
Class: JAR- COR 40%
700
600
500
400
300
200
100
0
Glucose
Fructose
Sucrose
Maltose

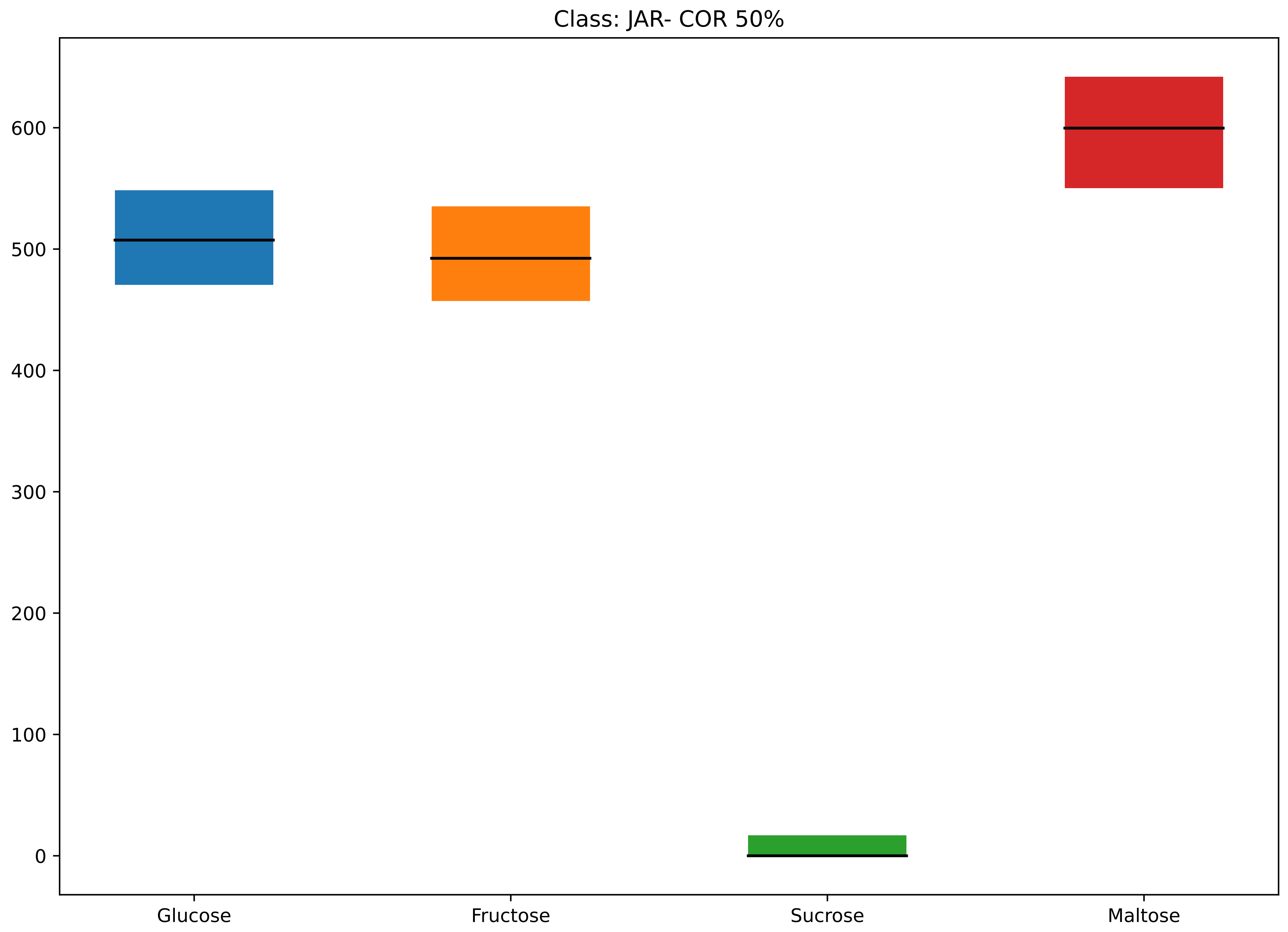

Class: JAR- COR 50%
600
500
400
300
200
100
0
Glucose
Fructose
Sucrose
Maltose

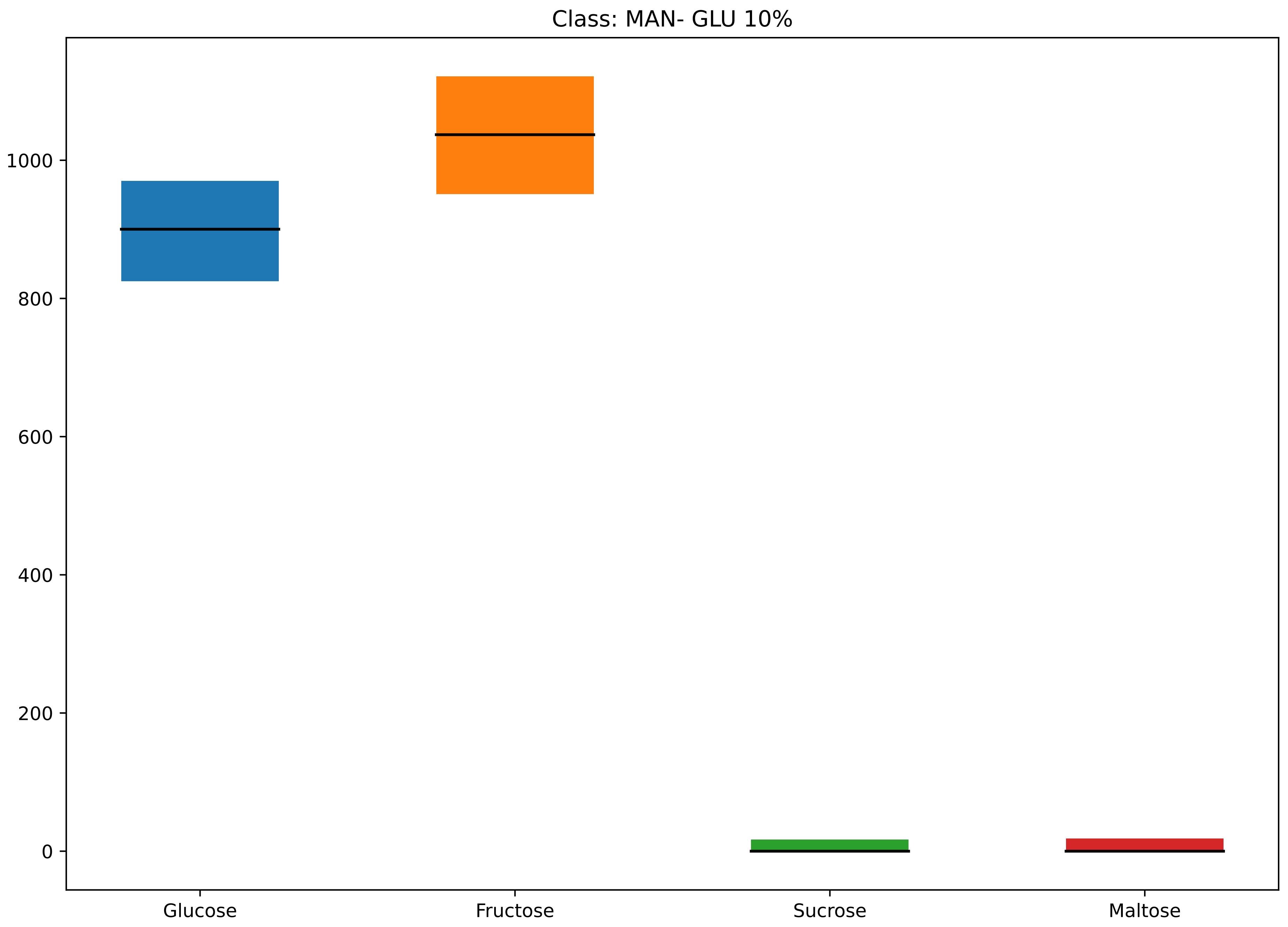

Class: MAN- GLU 10%
1000
800
600
400
200
0
Glucose
Fructose
Sucrose
Maltose

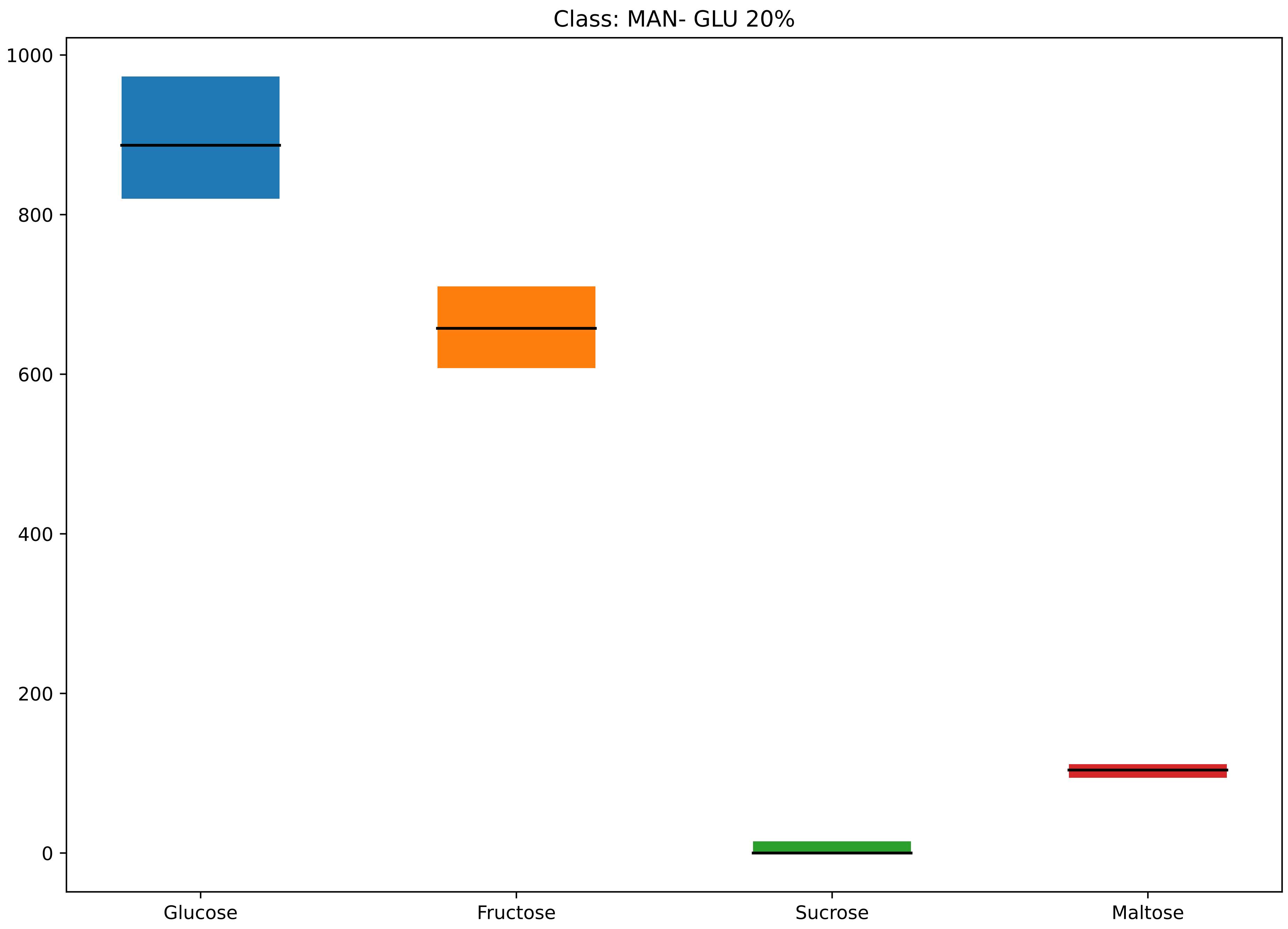

Class: MAN- GLU 20%
1000
800
600
400
200
0
Glucose
Fructose
Sucrose
Maltose

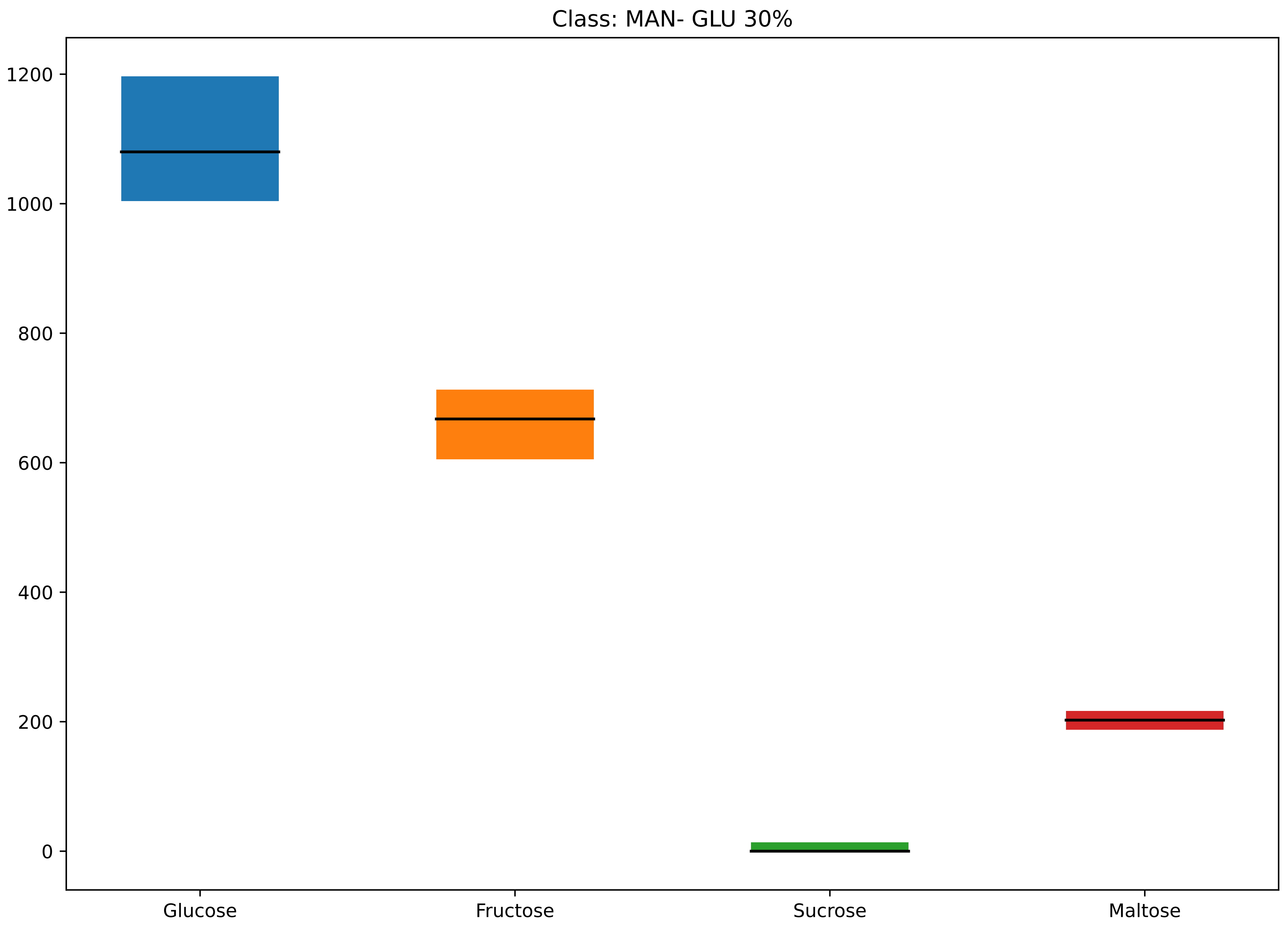
Class: MAN- GLU 30%
1200
1000
800
600
400
200
0
Glucose
Fructose
Sucrose
Maltose

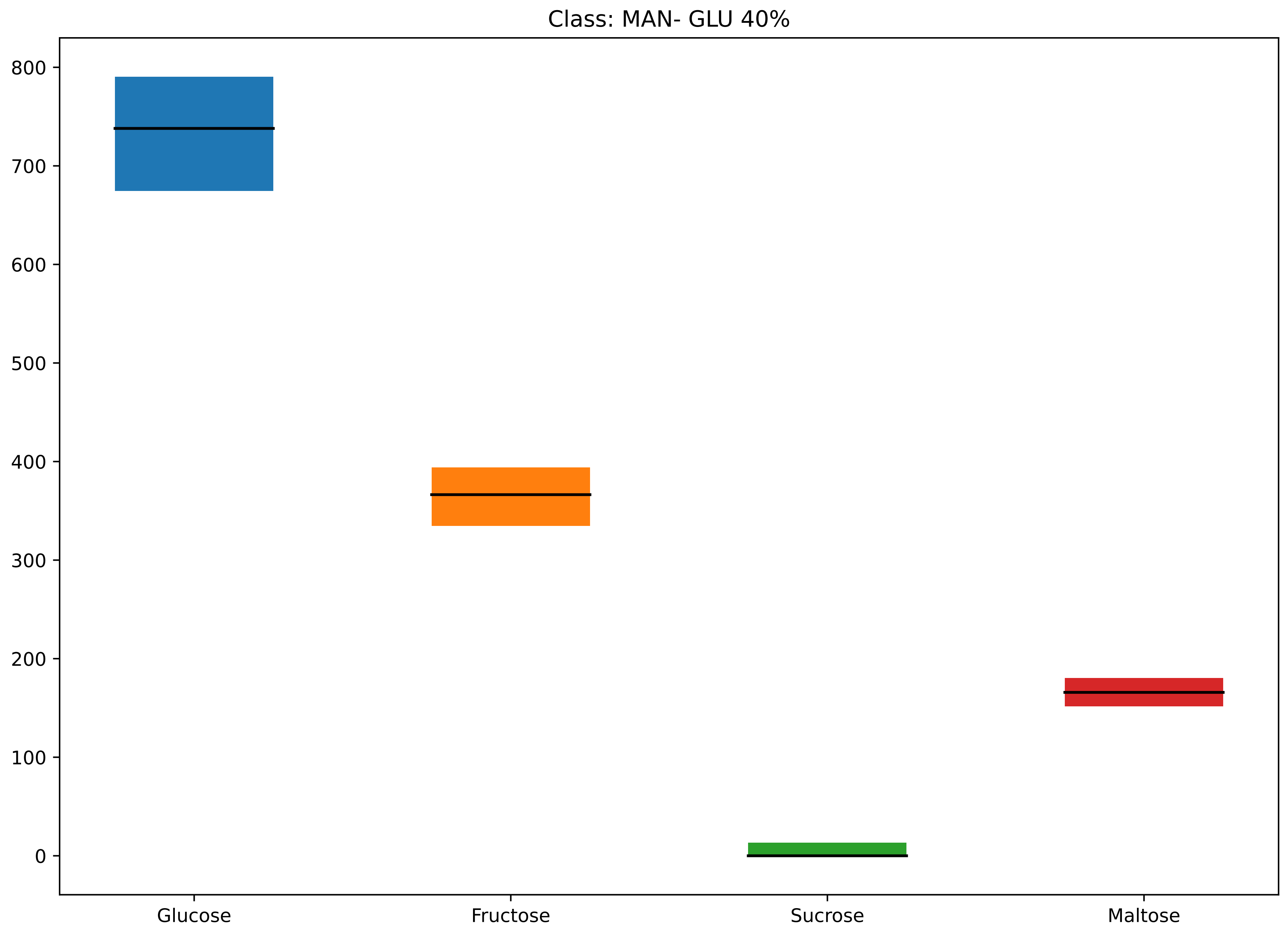

Class: MAN- GLU 40%
800
700
600
500
400
300
200
100
0
Glucose
Fructose
Sucrose
Maltose

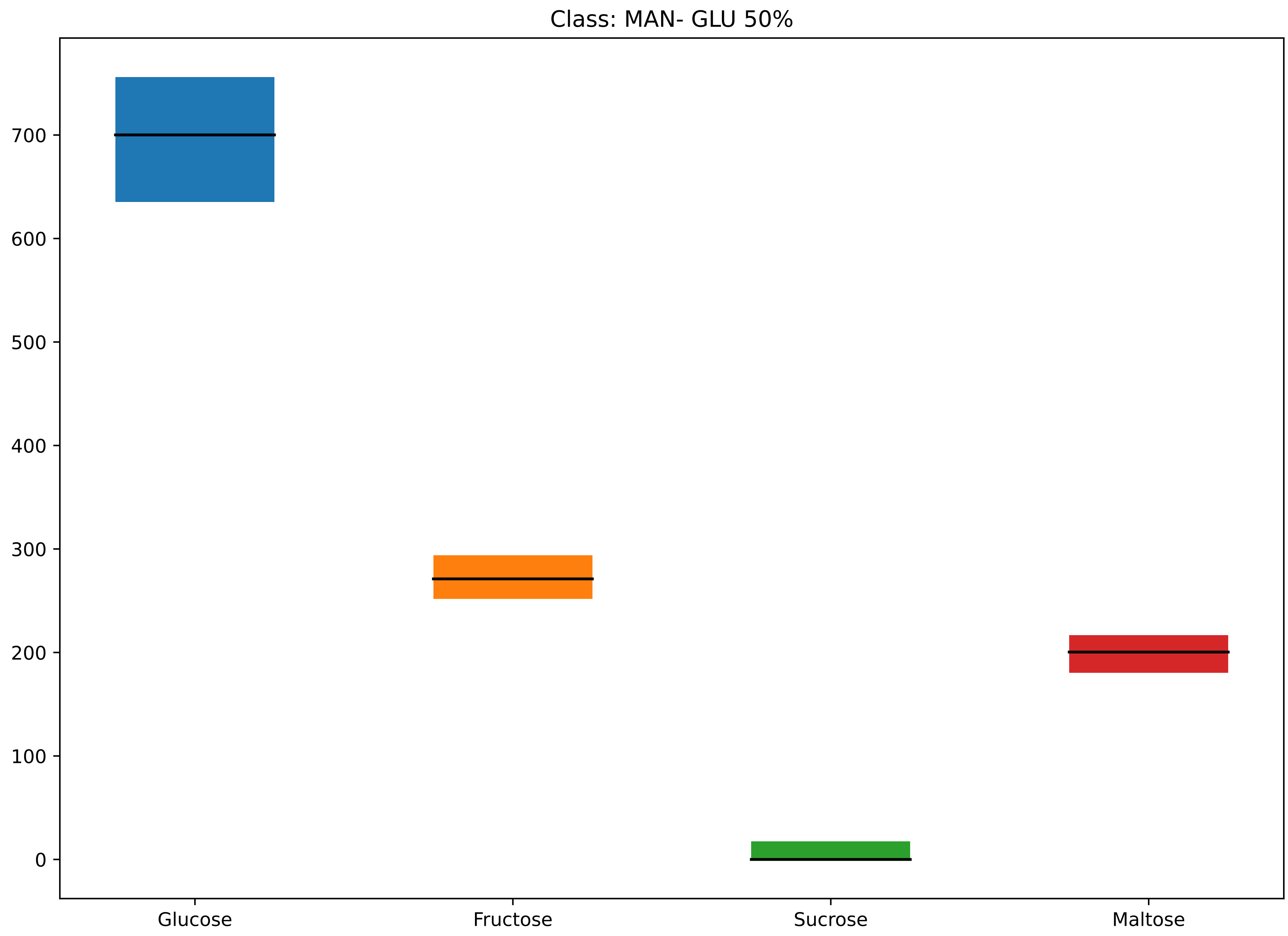

Class: MAN- GLU 50%
700
600
500
400
300
200
100
0
Glucose
Fructose
Sucrose
Maltose

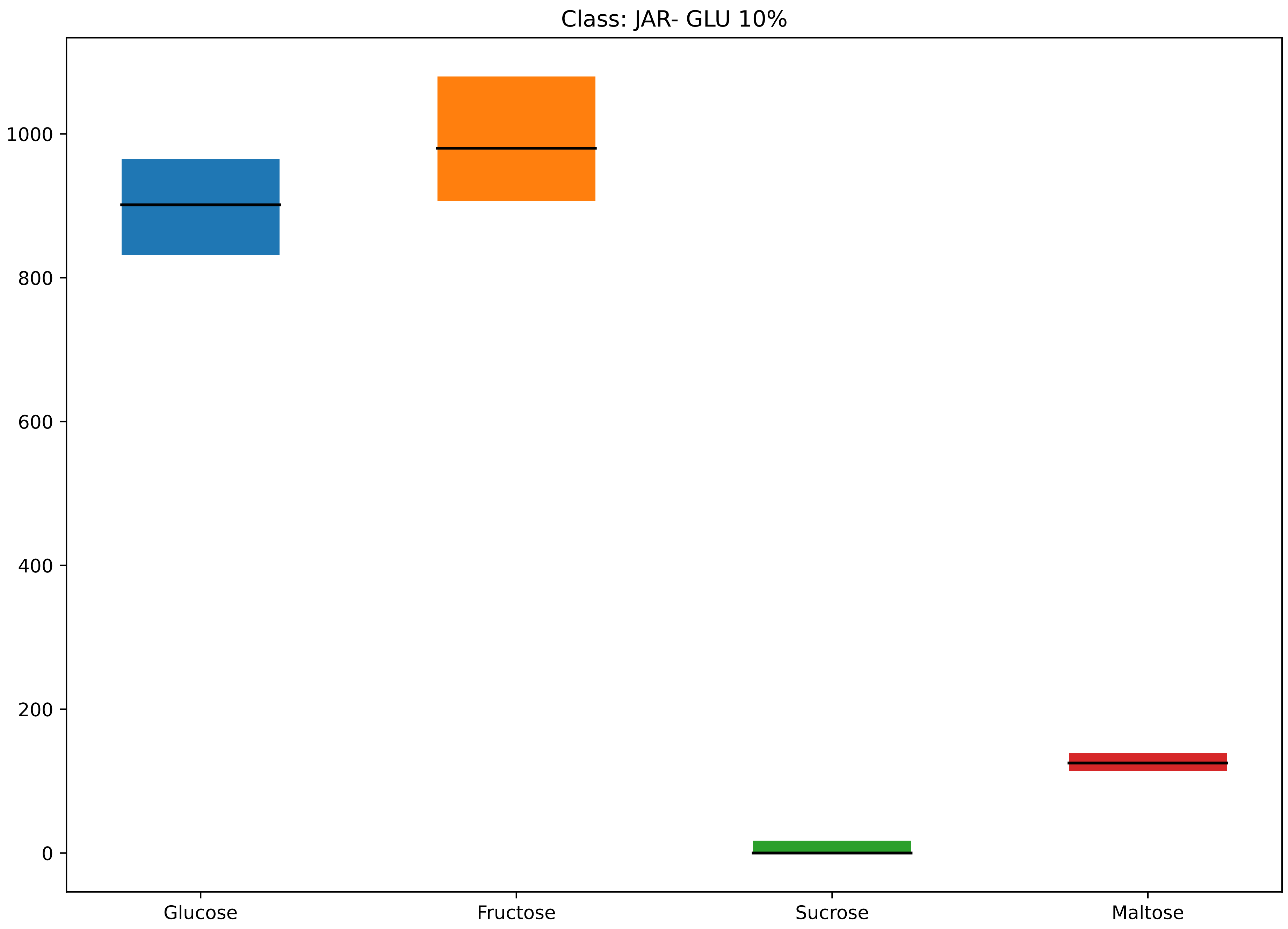
Class: JAR- GLU 10%
1000
800
600
400
200
0
Glucose
Fructose
Sucrose
Maltose

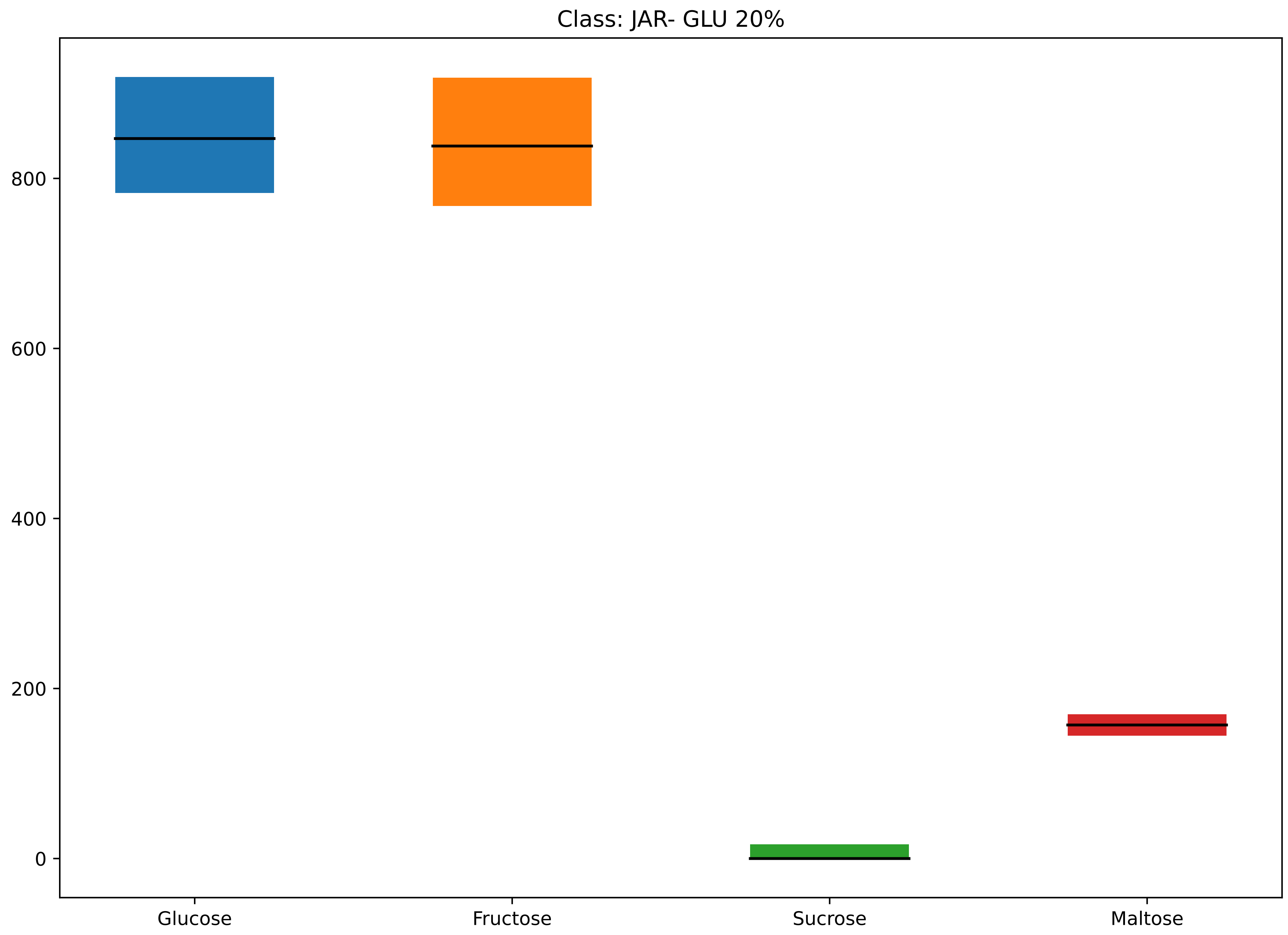

Class: JAR- GLU 20%
800
600
400
200
0
Glucose
Fructose
Sucrose
Maltose

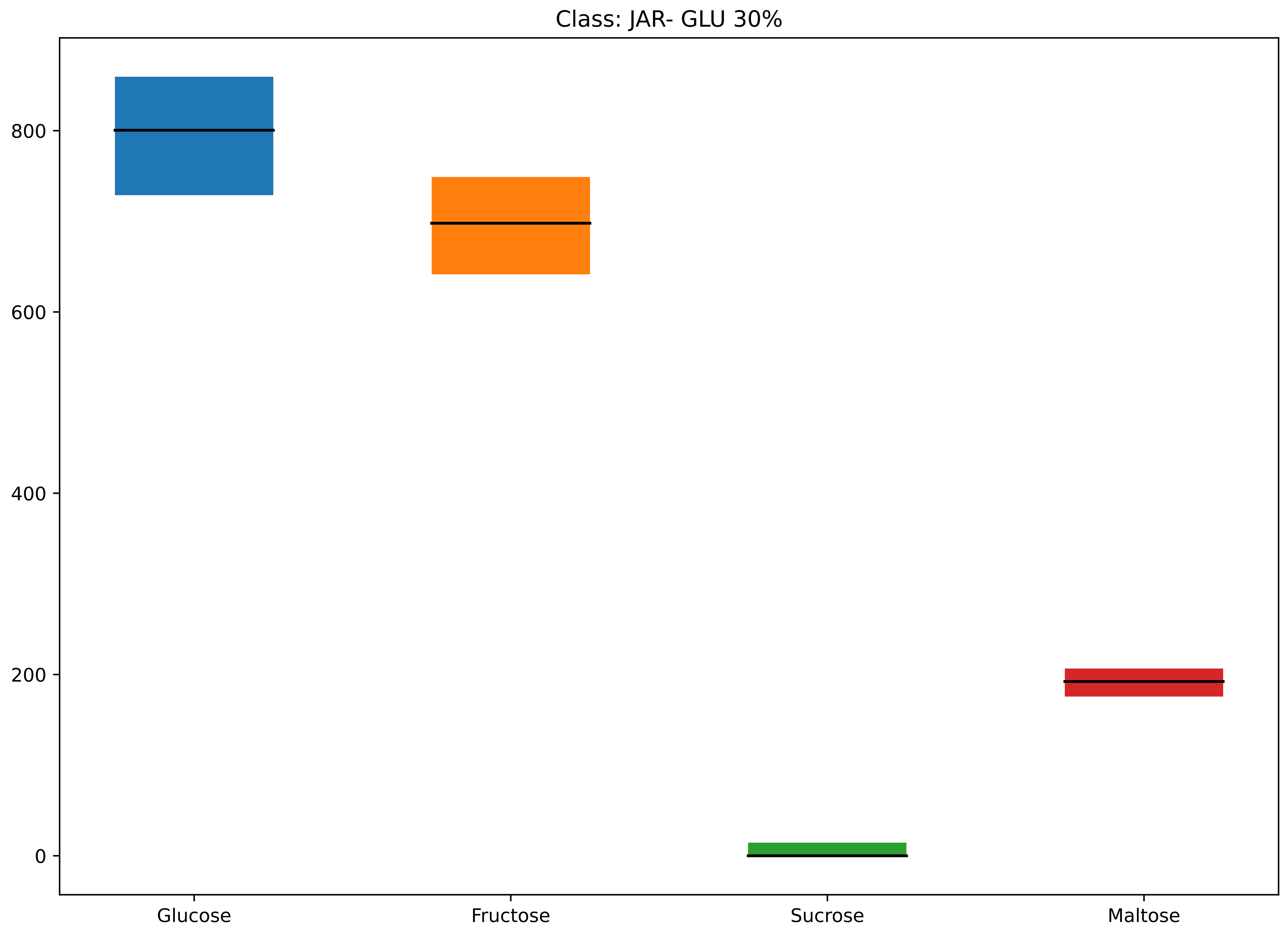
Class: JAR- GLU 30%
800
600
400
200
0
Glucose
Fructose
Sucrose
Maltose

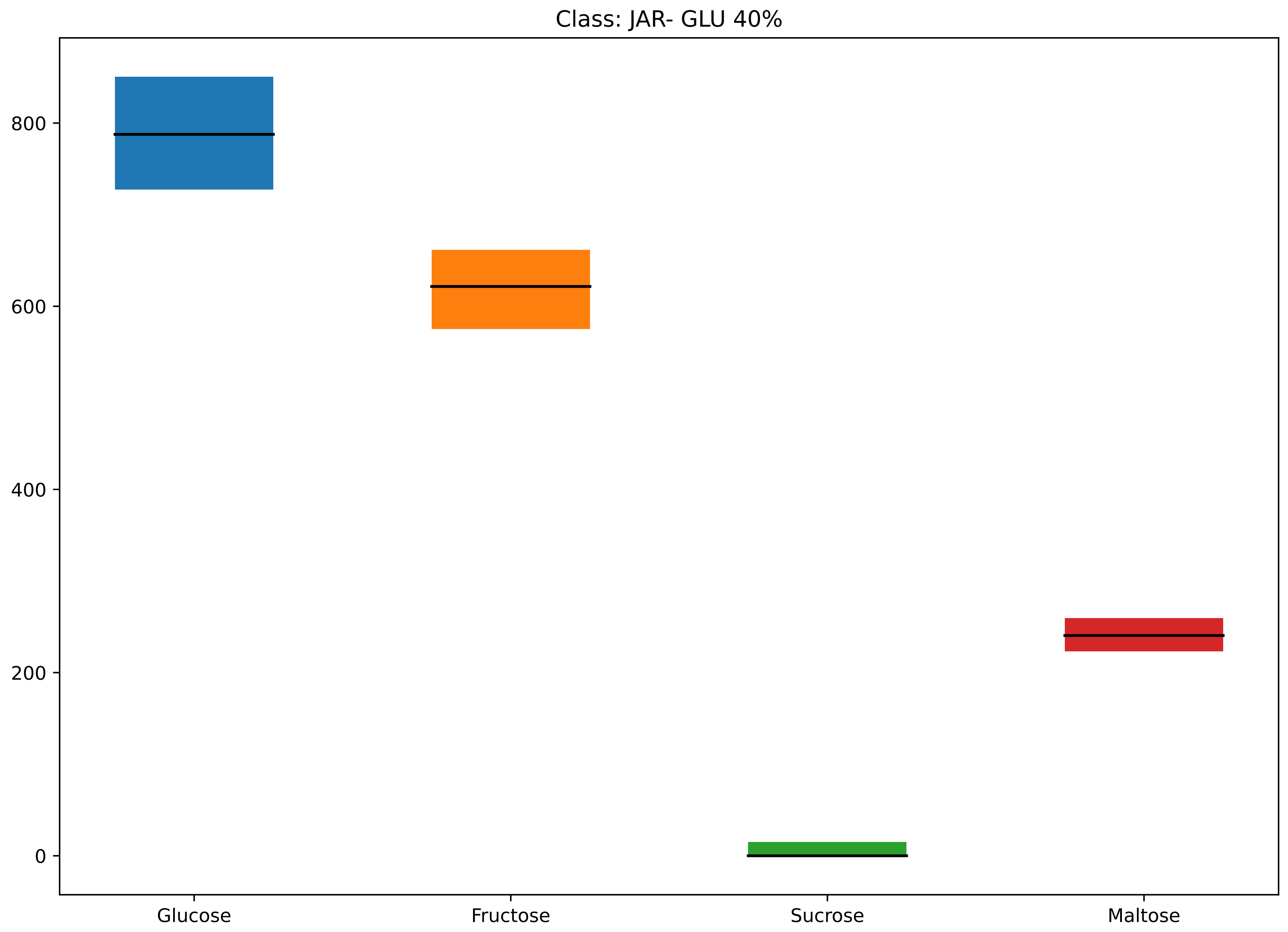

Class: JAR- GLU 40%
800
600
400
200
0
Glucose
Fructose
Sucrose
Maltose

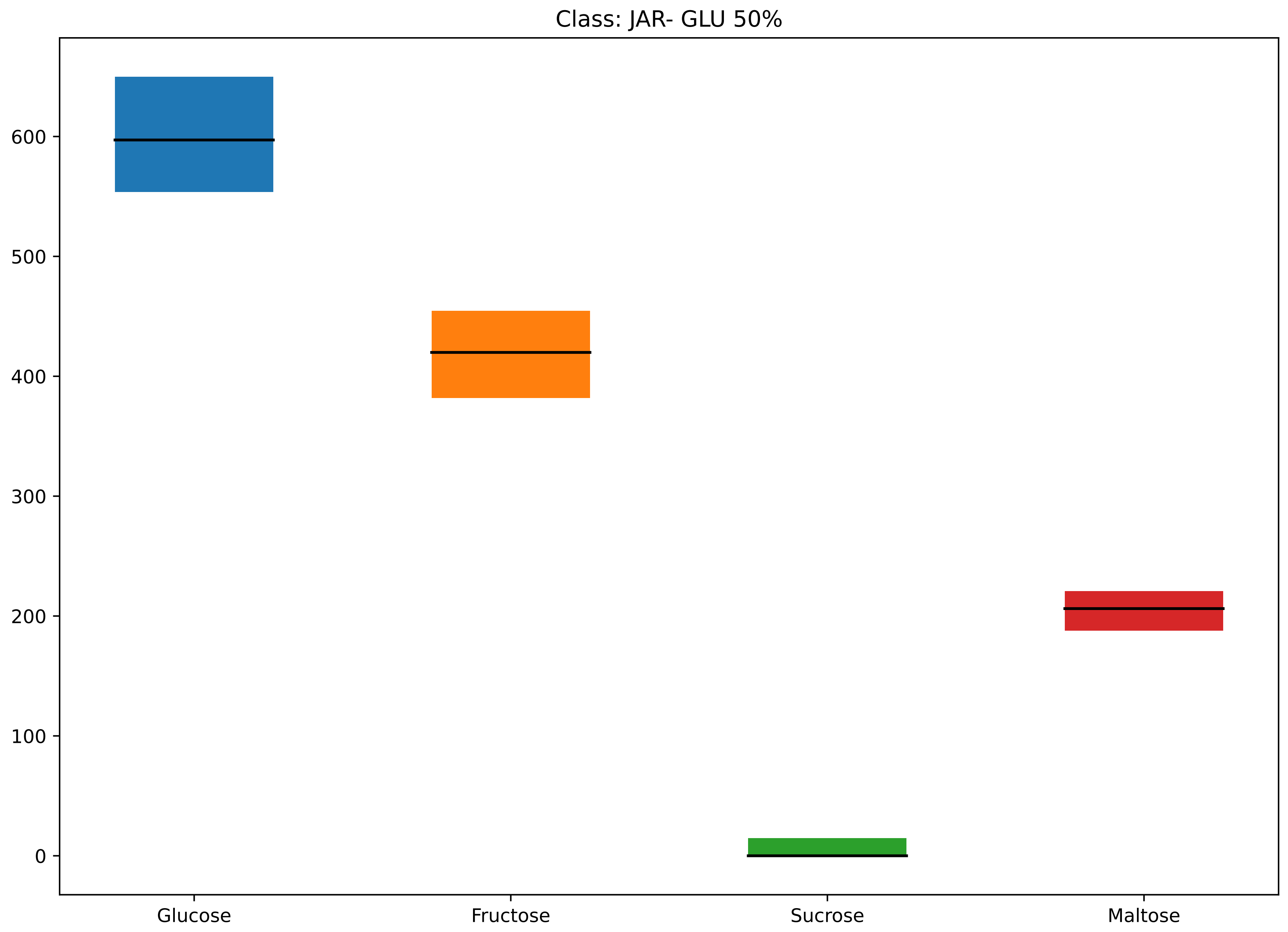
Class: JAR- GLU 50%
600
500
400
300
200
100
0
Glucose
Fructose
Sucrose
Maltose

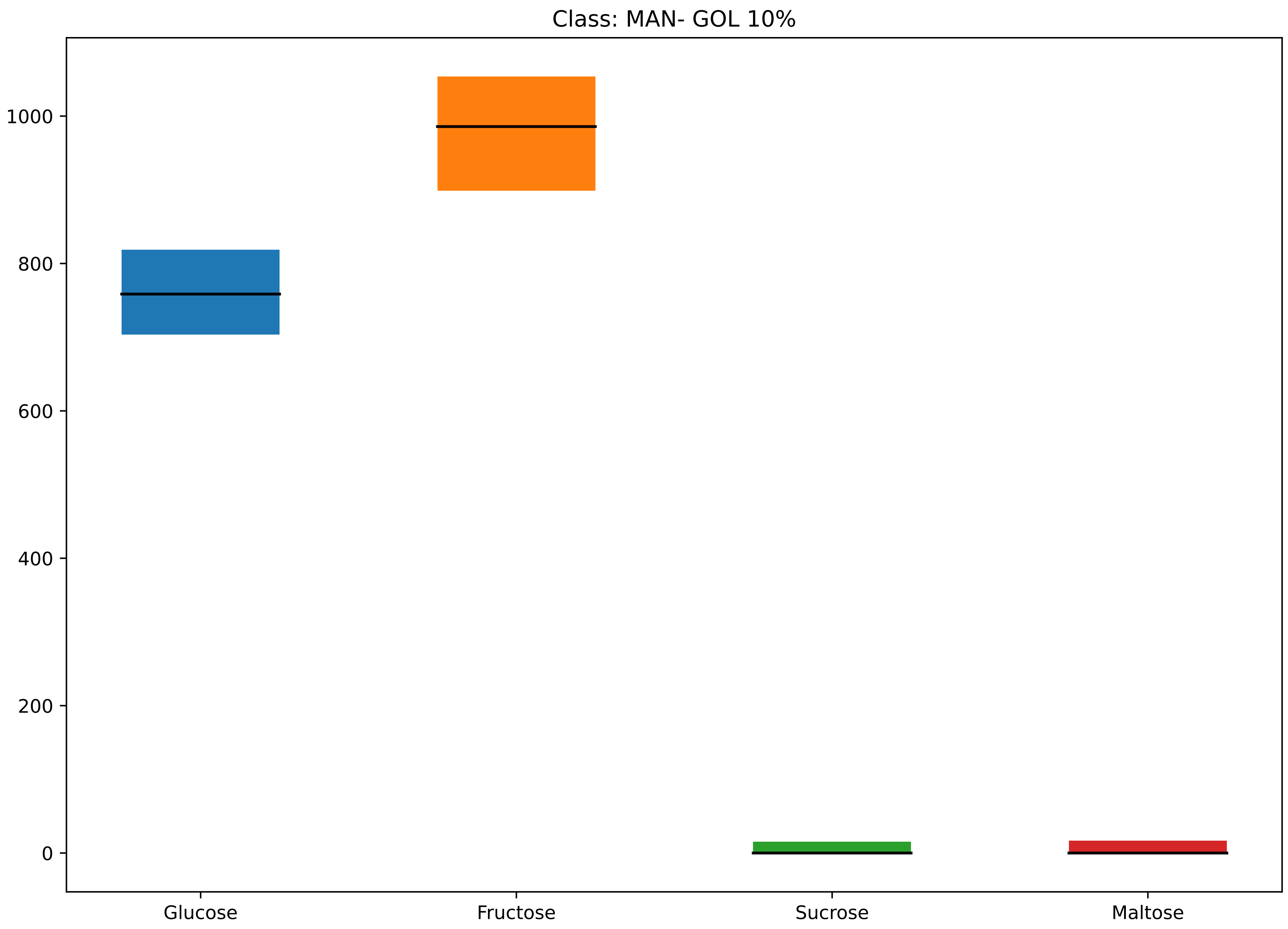
Class: MAN- GOL 10%
1000
800
600
400
200
0
Glucose
Fructose
Sucrose
Maltose

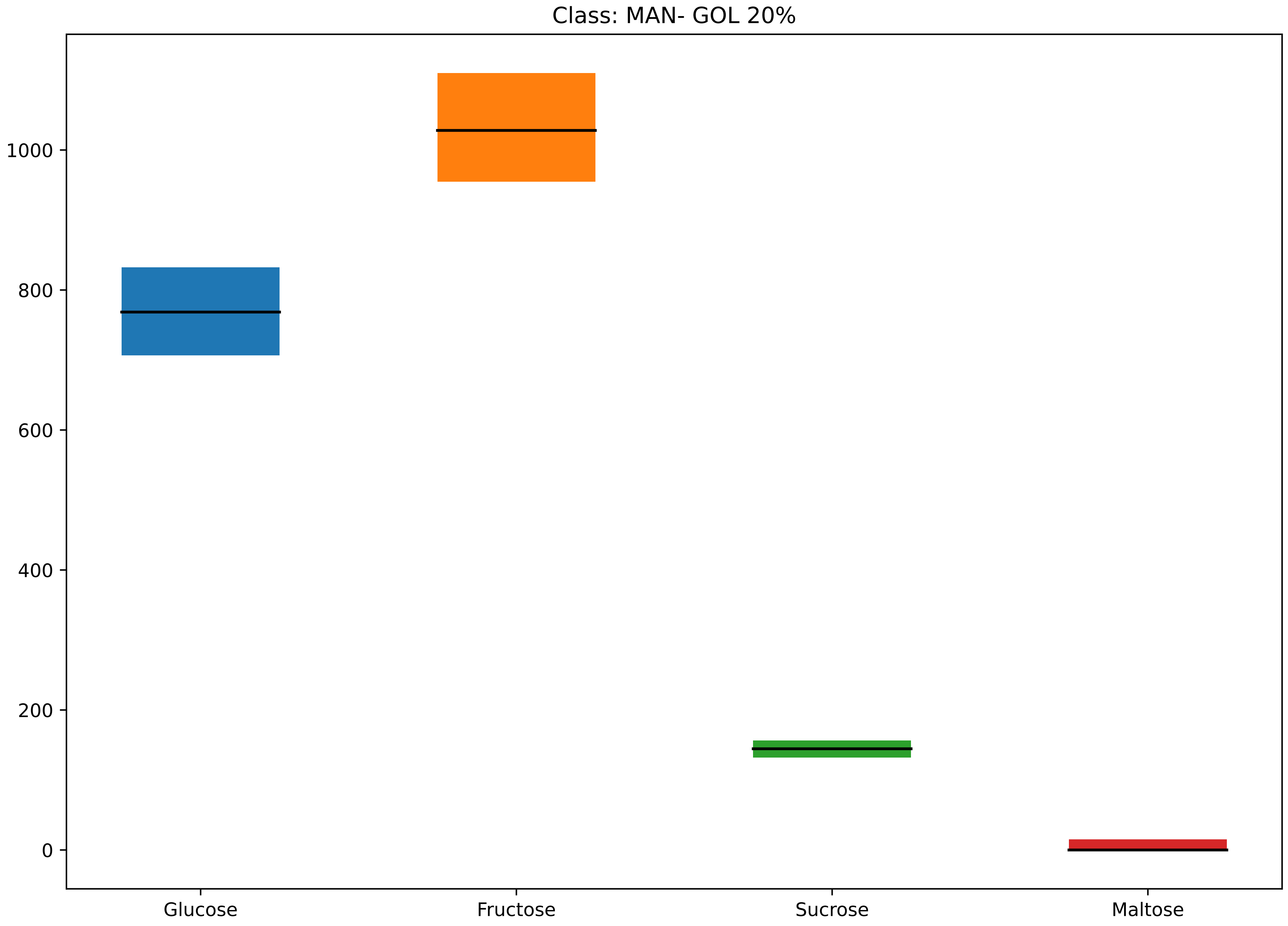

Class: MAN- GOL 20%
0
200
400
600
800
1000
Glucose
Fructose
Sucrose
Maltose

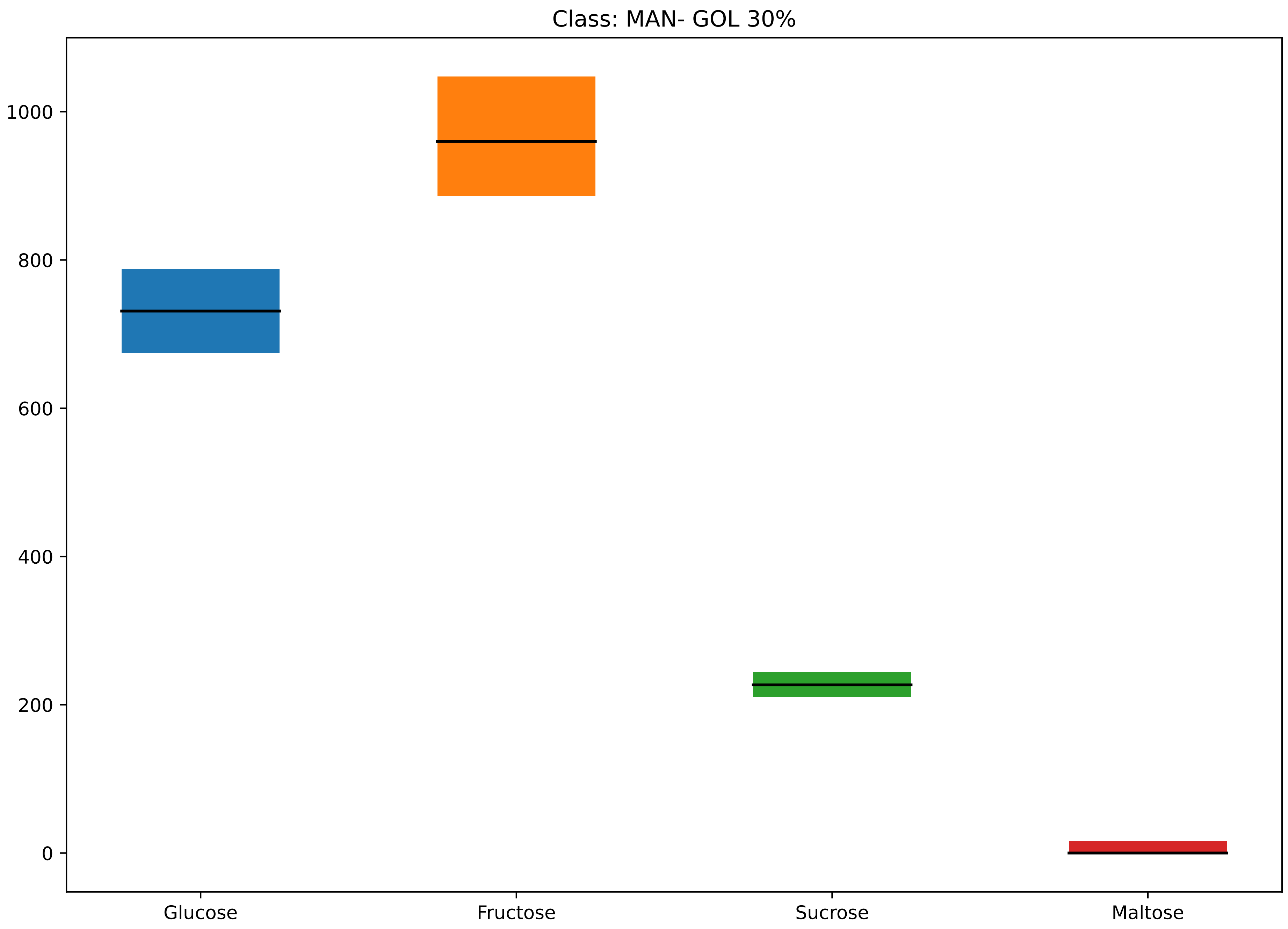

Class: MAN- GOL 30%
1000
800
600
400
200
0
Glucose
Fructose
Sucrose
Maltose

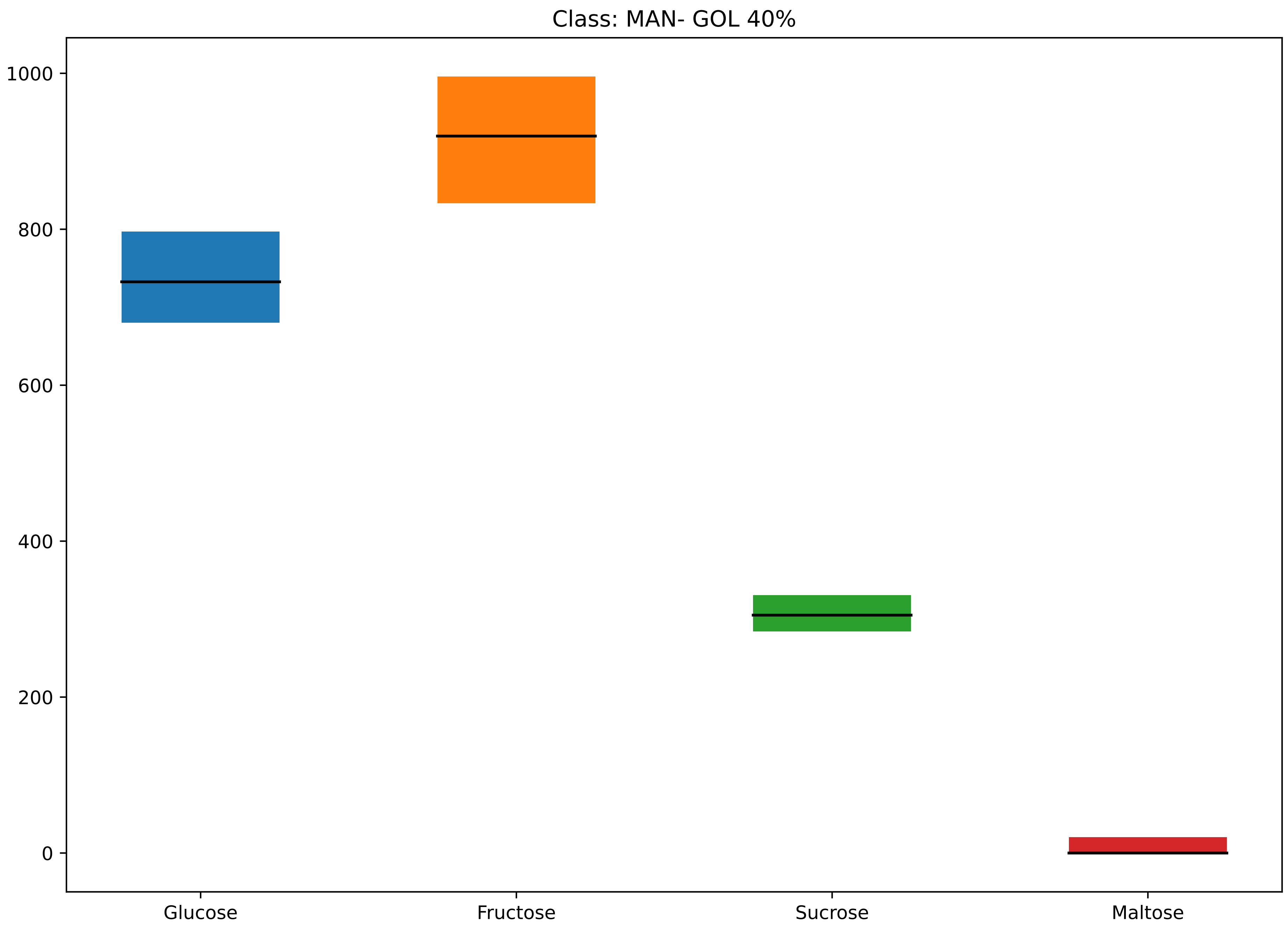
Class: MAN- GOL 40%
1000
800
600
400
200
0
Glucose
Fructose
Sucrose
Maltose

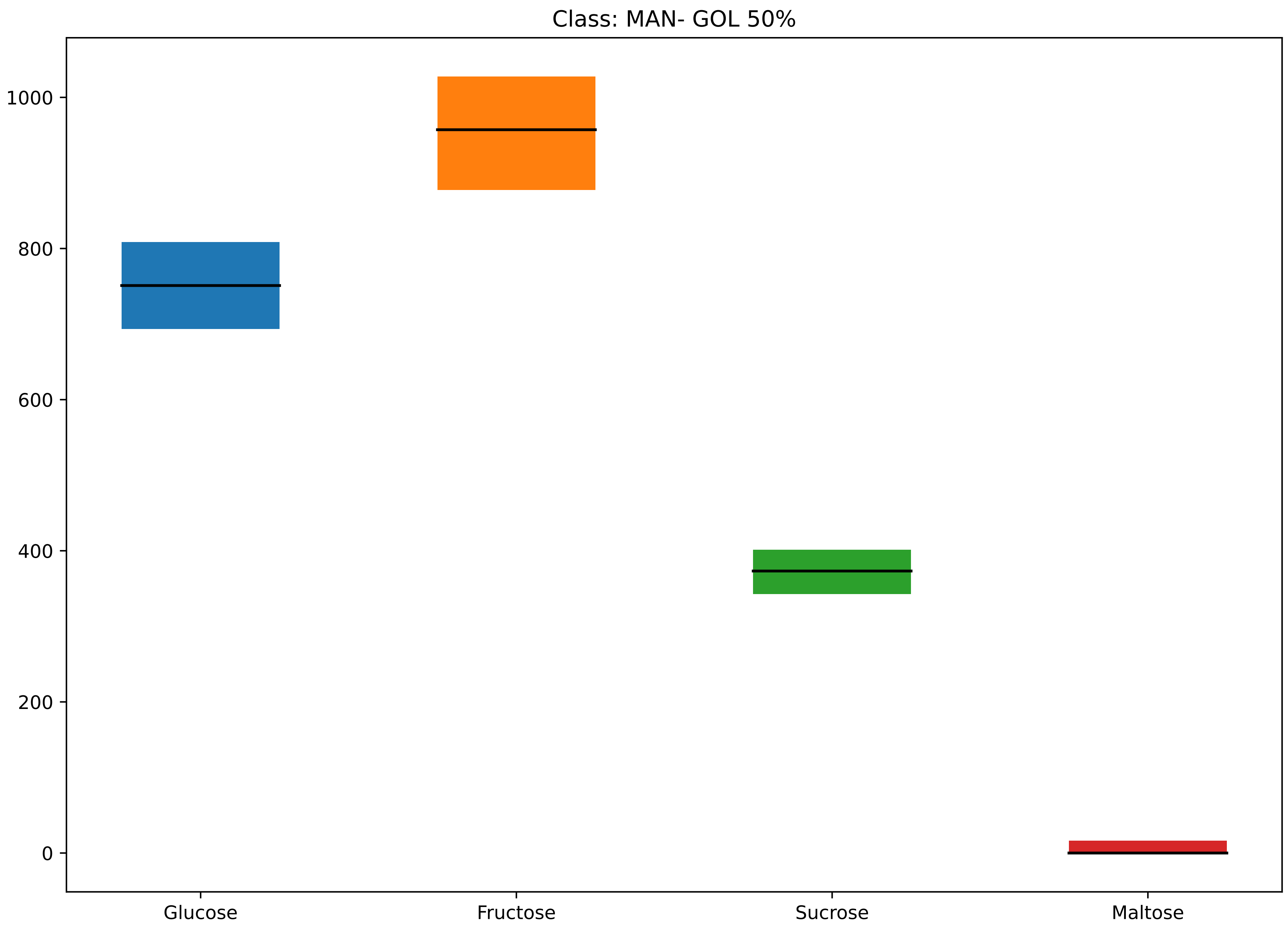
Class: MAN- GOL 50%
1000
800
600
400
200
0
Glucose
Fructose
Sucrose
Maltose

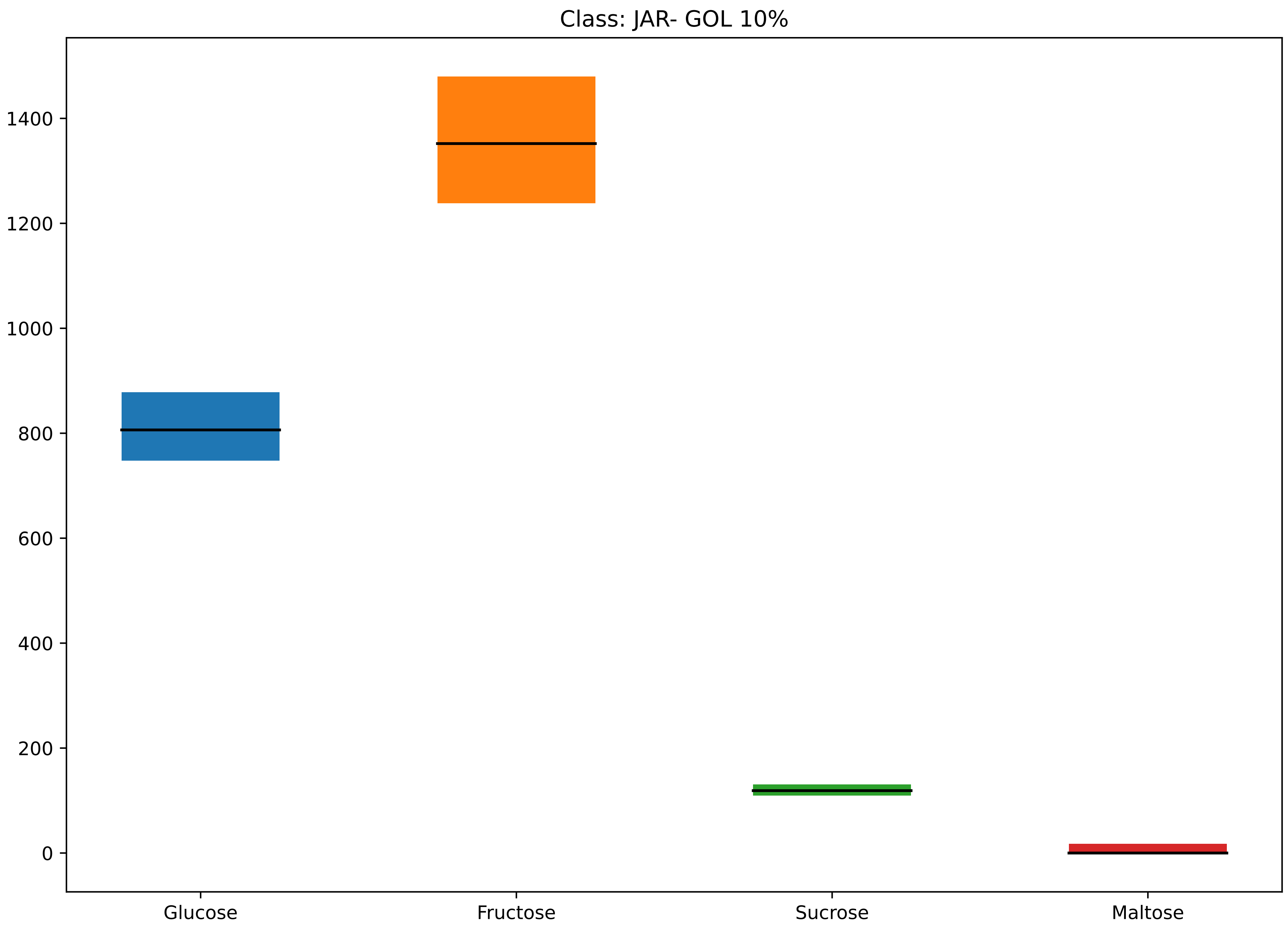

Class: JAR- GOL 10%
1400
1200
1000
800
600
400
200
0
Glucose
Fructose
Sucrose
Maltose

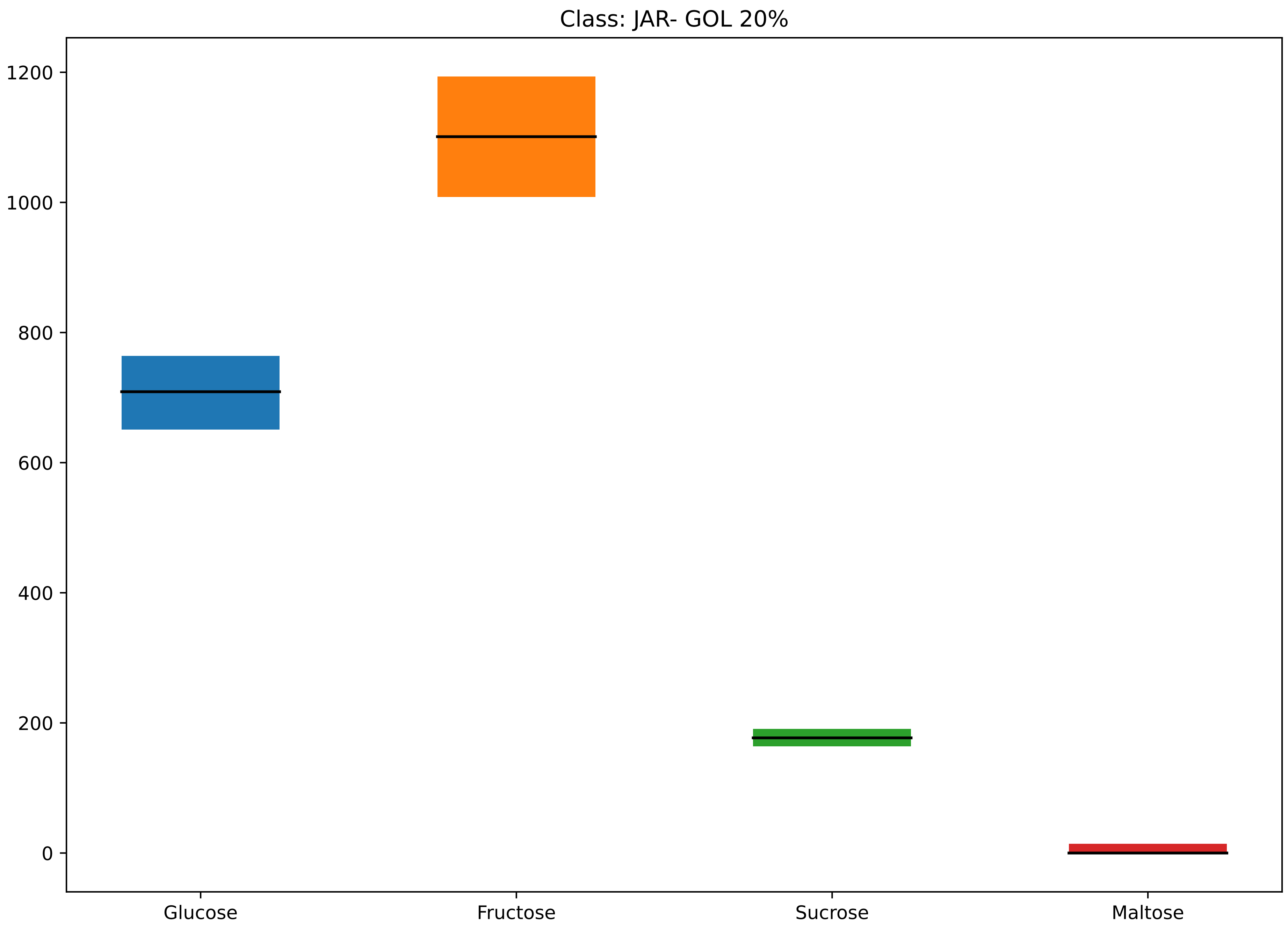
Class: JAR- GOL 20%
1200
1000
800
600
400
200
0
Glucose
Fructose
Sucrose
Maltose

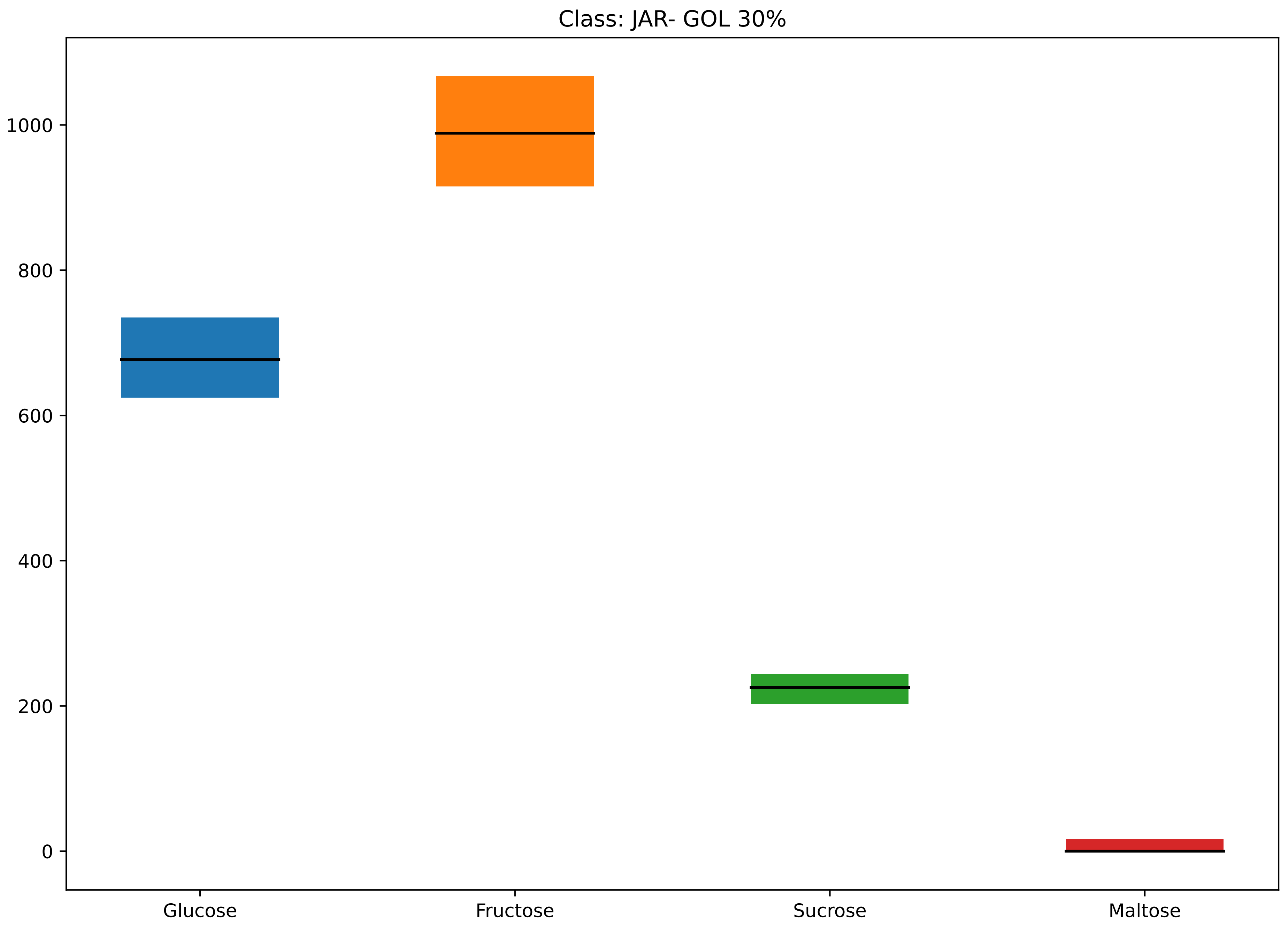
Class: JAR- GOL 30%
1000
800
600
400
200
0
Glucose
Fructose
Sucrose
Maltose

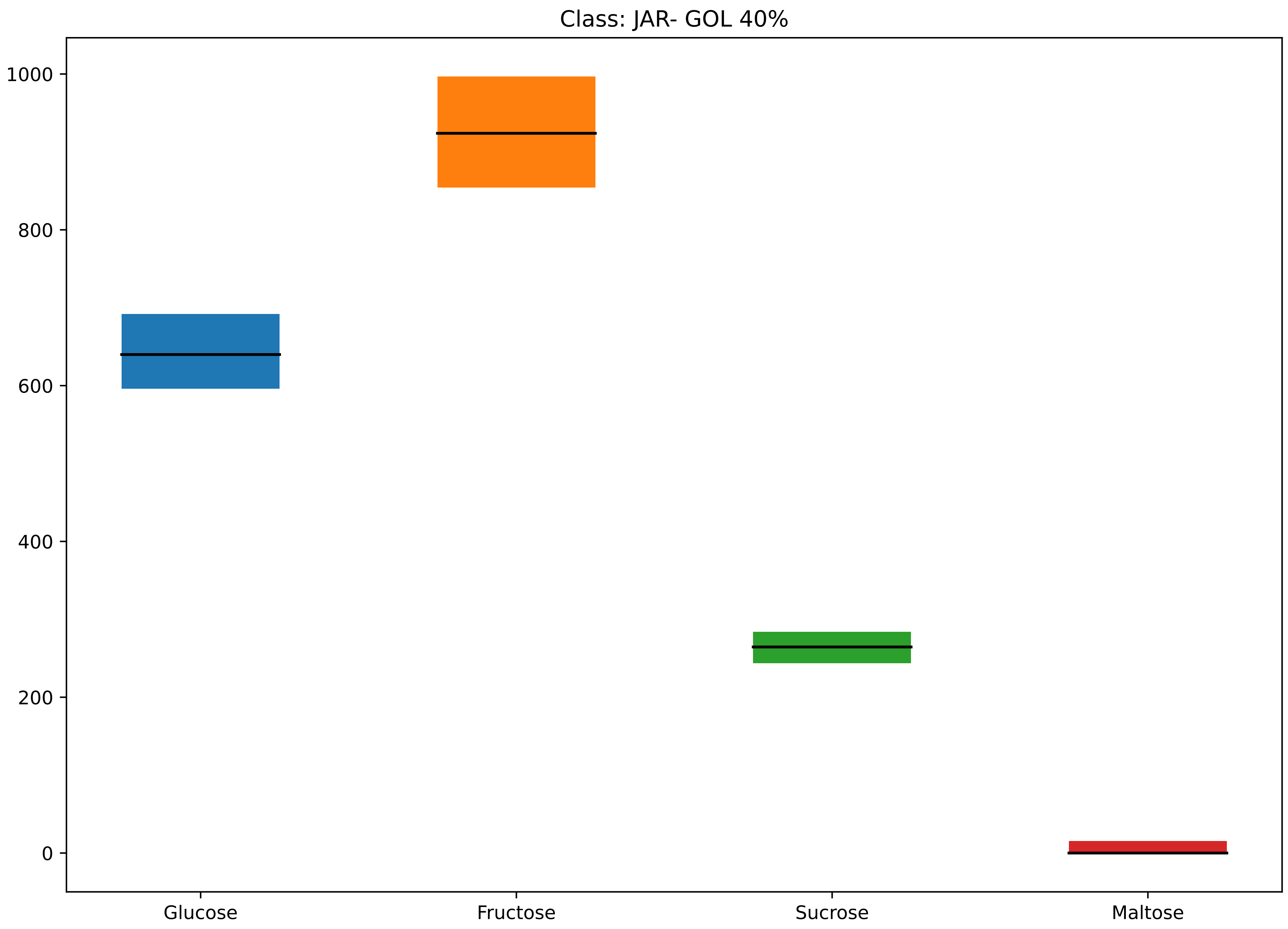

Class: JAR- GOL 40%
1000
800
600
400
200
0
Glucose
Fructose
Sucrose
Maltose

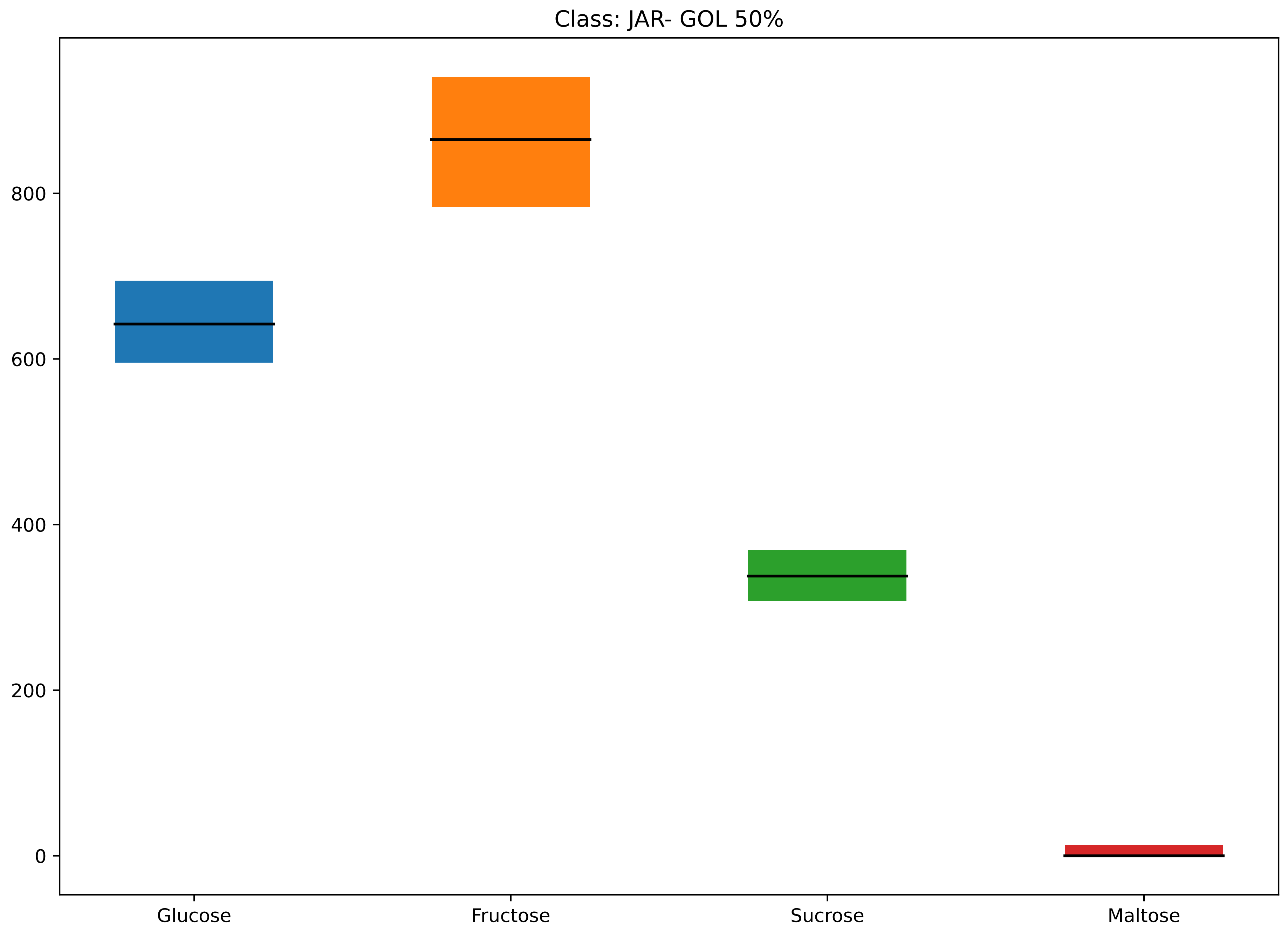
Class: JAR- GOL 50%
800
600
400
200
0
Glucose
Fructose
Sucrose
Maltose

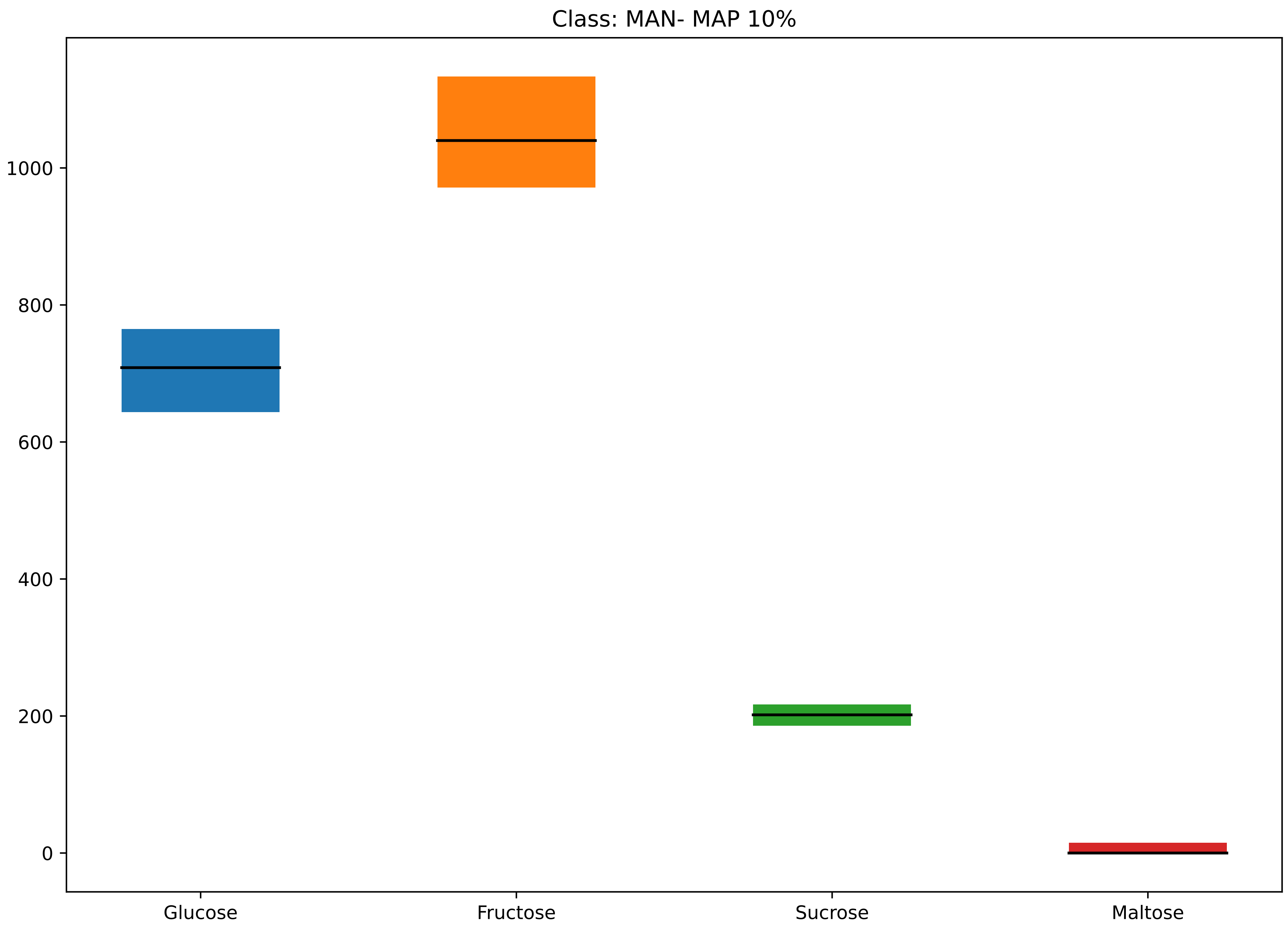
Class: MAN- MAP 10%
1000
800
600
400
200
0
Glucose
Fructose
Sucrose
Maltose

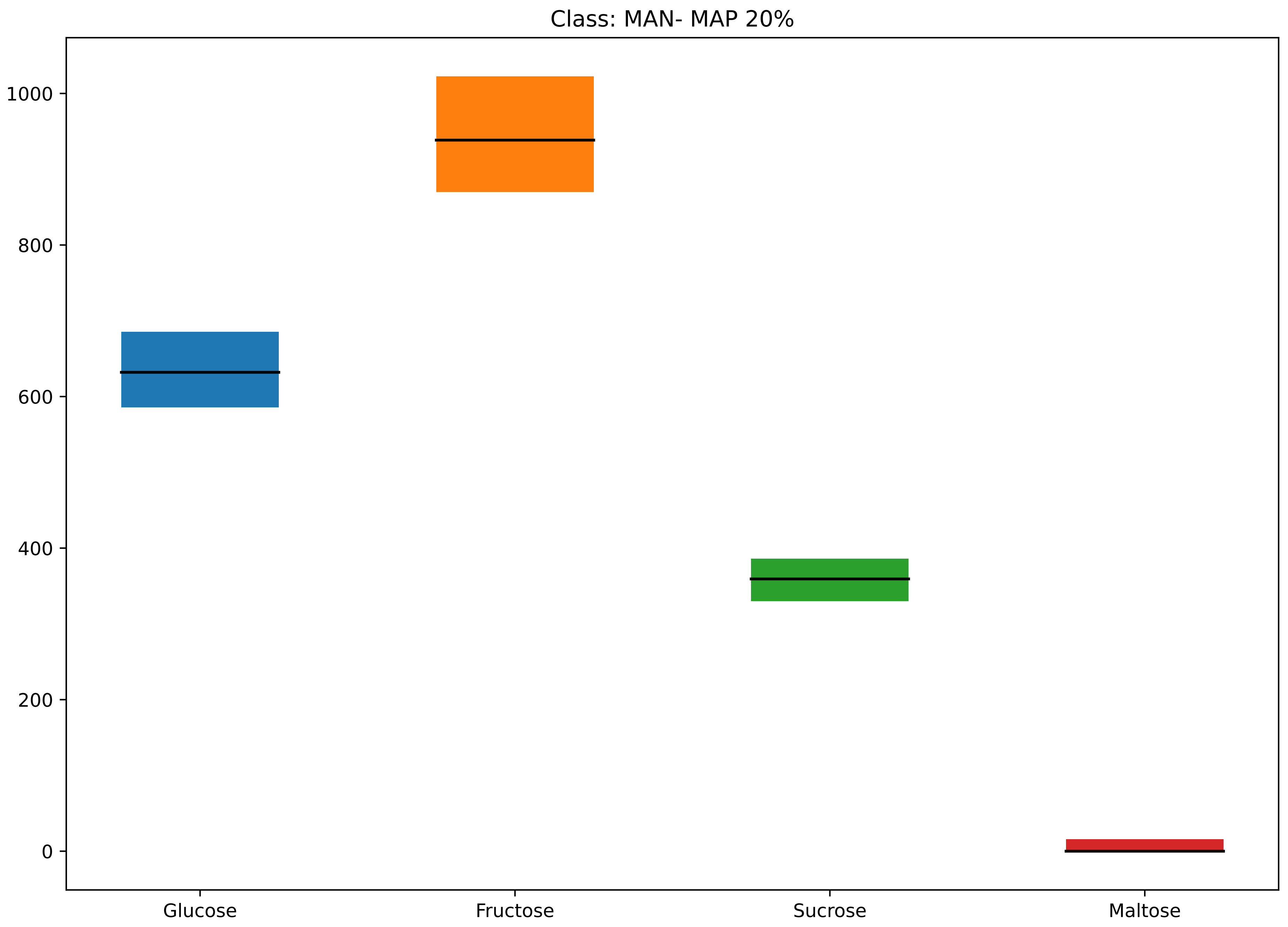

Class: MAN- MAP 20%
1000
800
600
400
200
0
Glucose
Fructose
Sucrose
Maltose

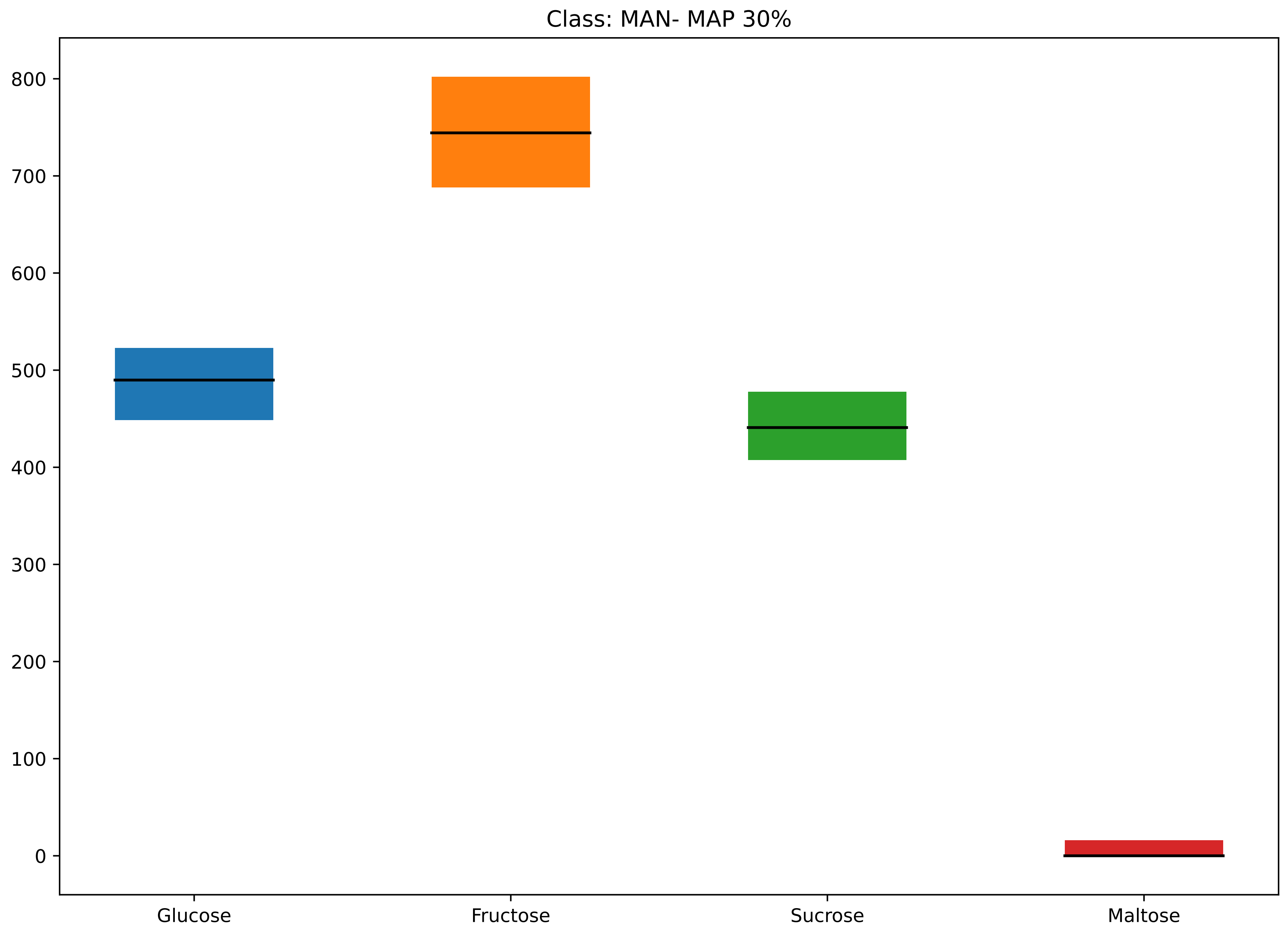

Class: MAN- MAP 30%
800
700
600
500
400
300
200
100
0
Glucose
Fructose
Sucrose
Maltose

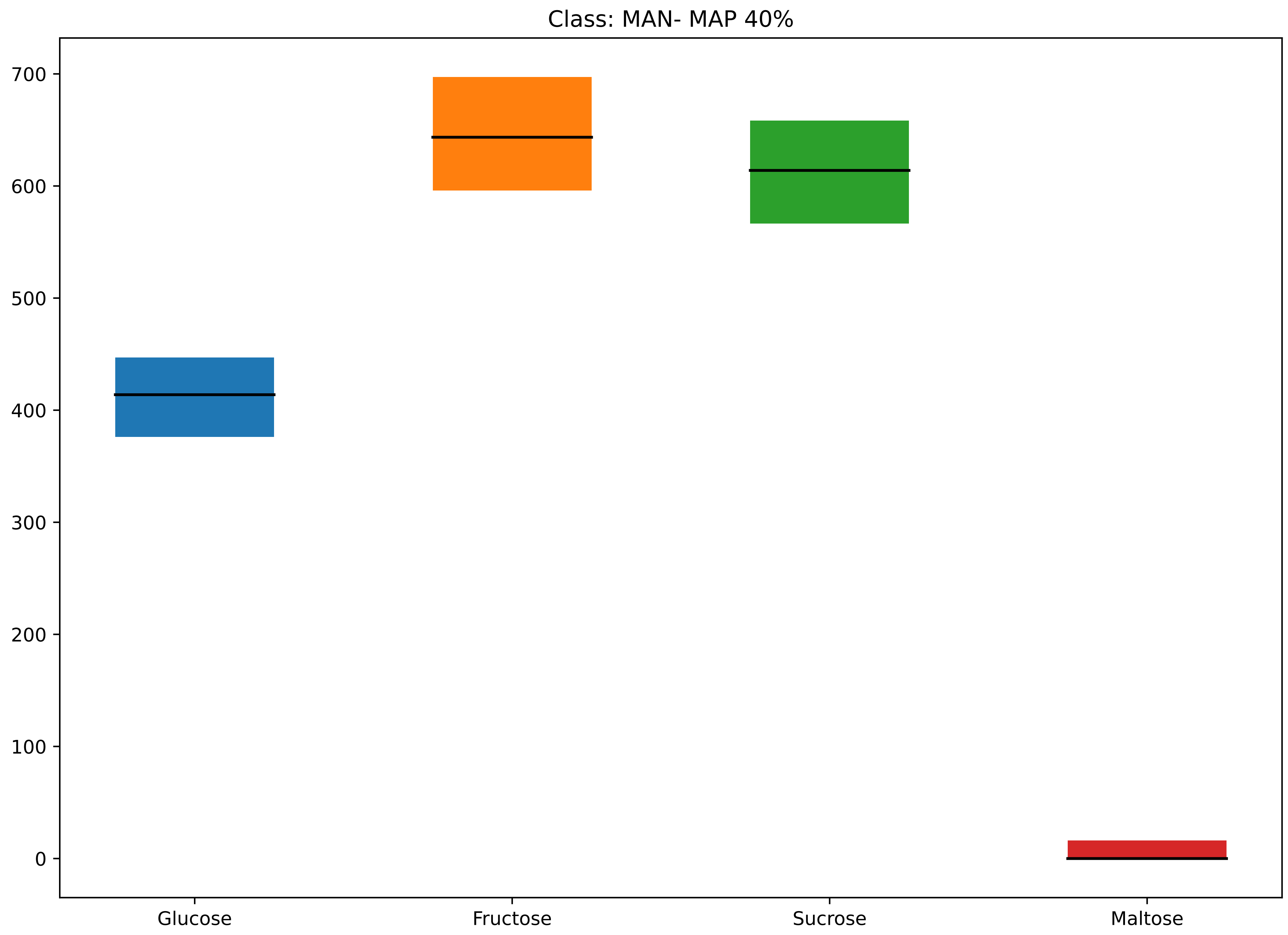
Class: MAN- MAP 40%
700
600
500
400
300
200
100
0
Glucose
Fructose
Sucrose
Maltose

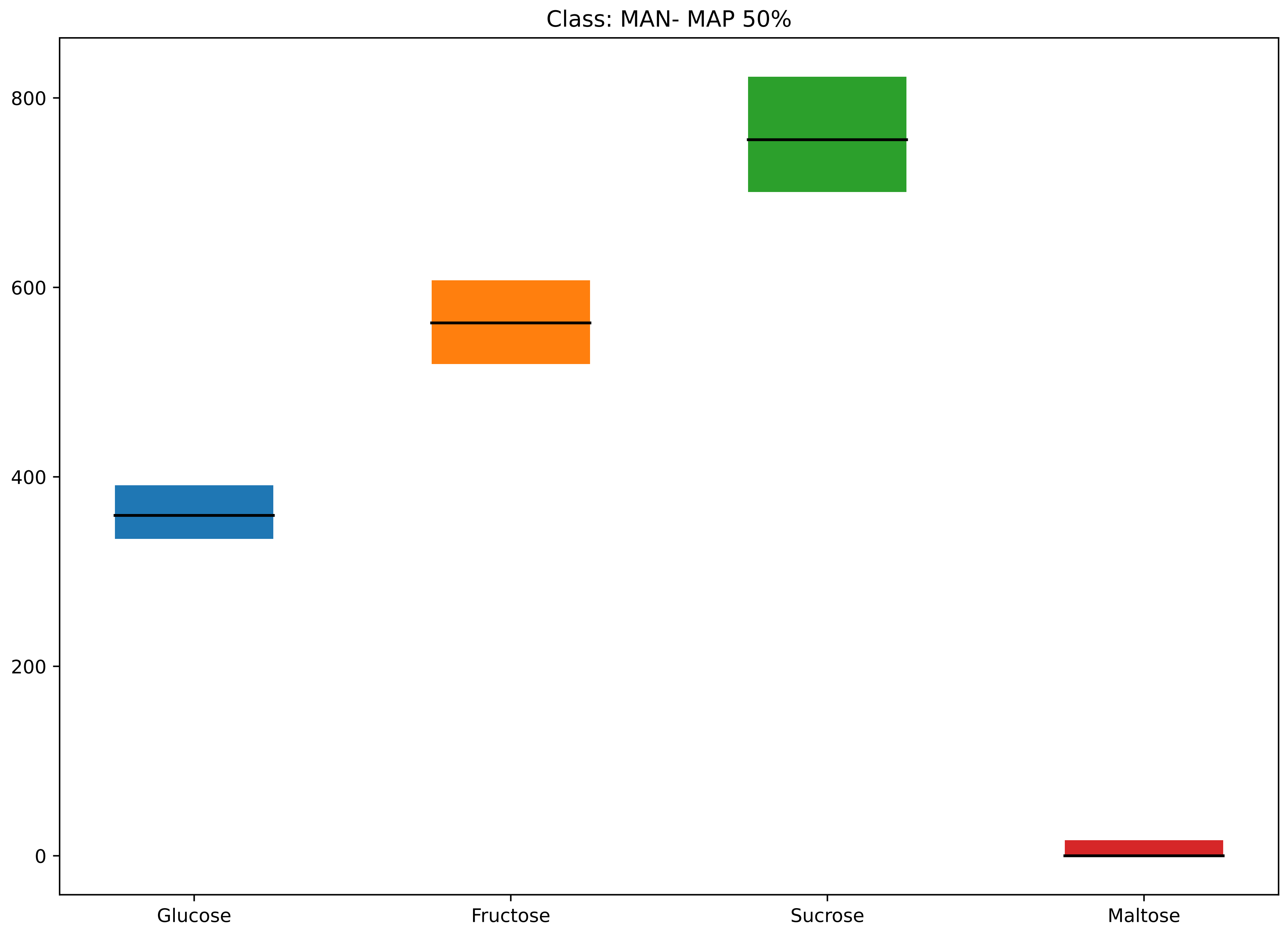
Class: MAN- MAP 50%
800
600
400
200
0
Glucose
Fructose
Sucrose
Maltose

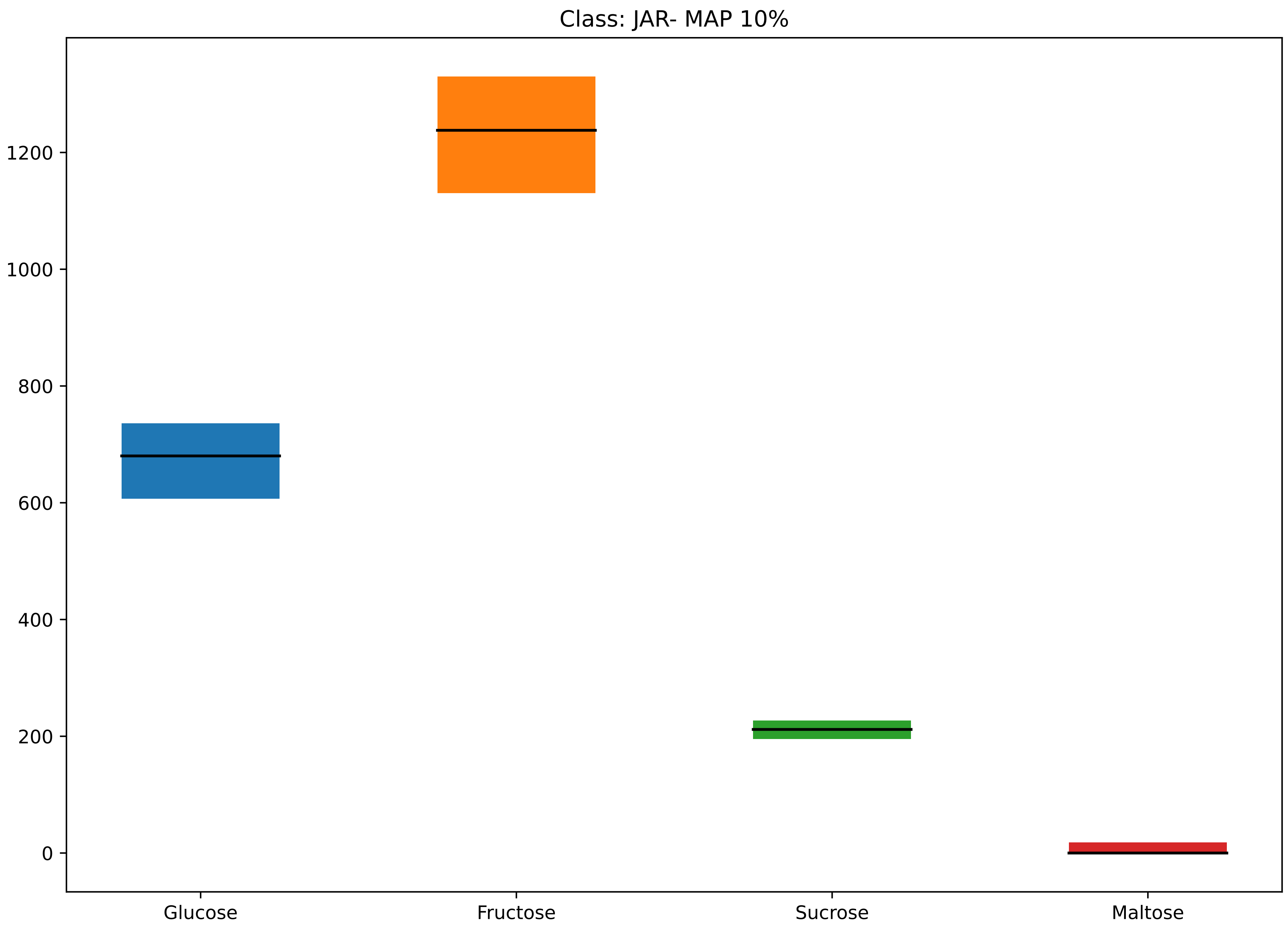

Class: JAR- MAP 10%
1200
1000
800
600
400
200
0
Glucose
Fructose
Sucrose
Maltose

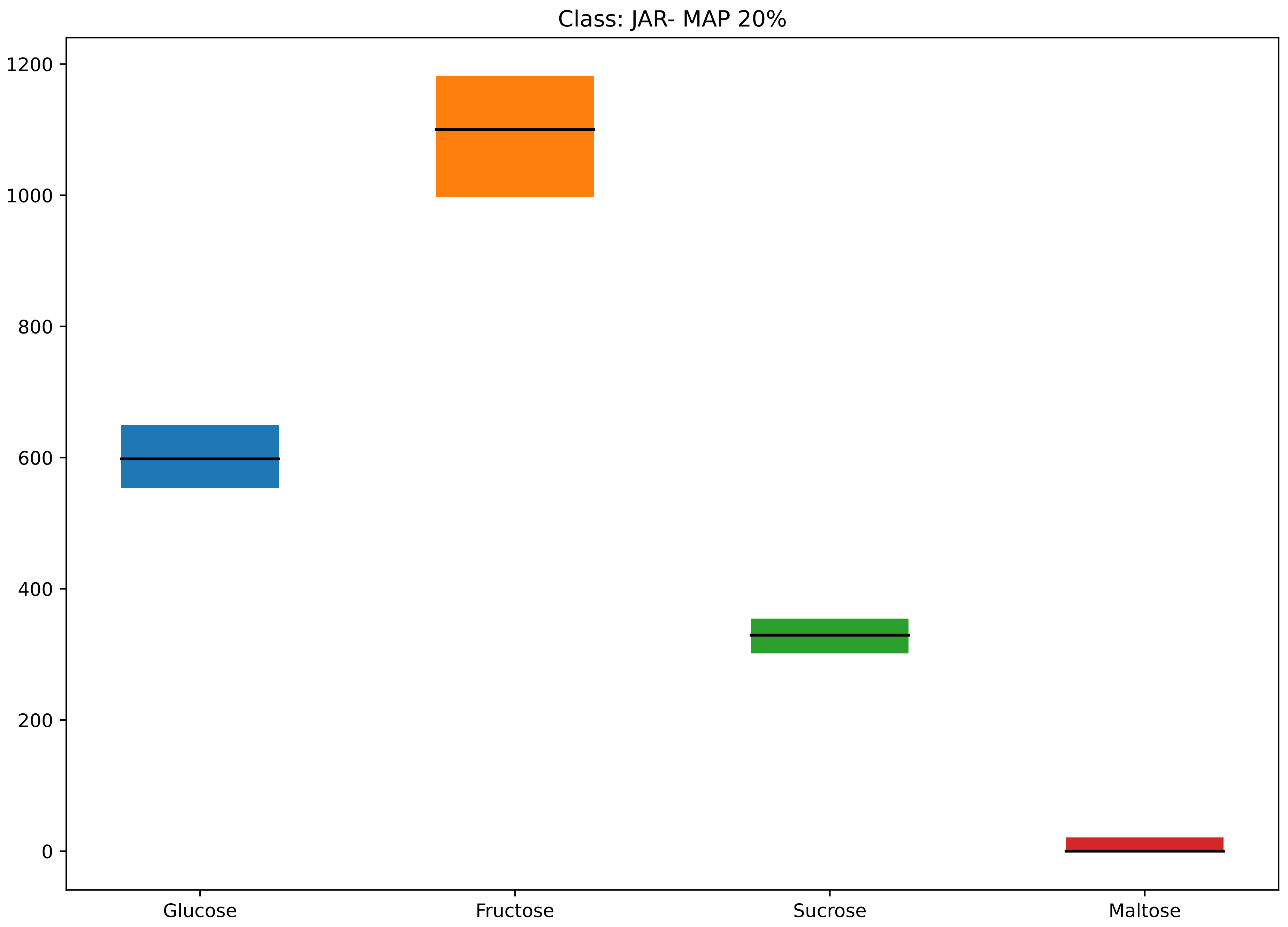

Class: JAR- MAP 20%
1200
1000
800
600
400
200
0
Glucose
Fructose
Sucrose
Maltose

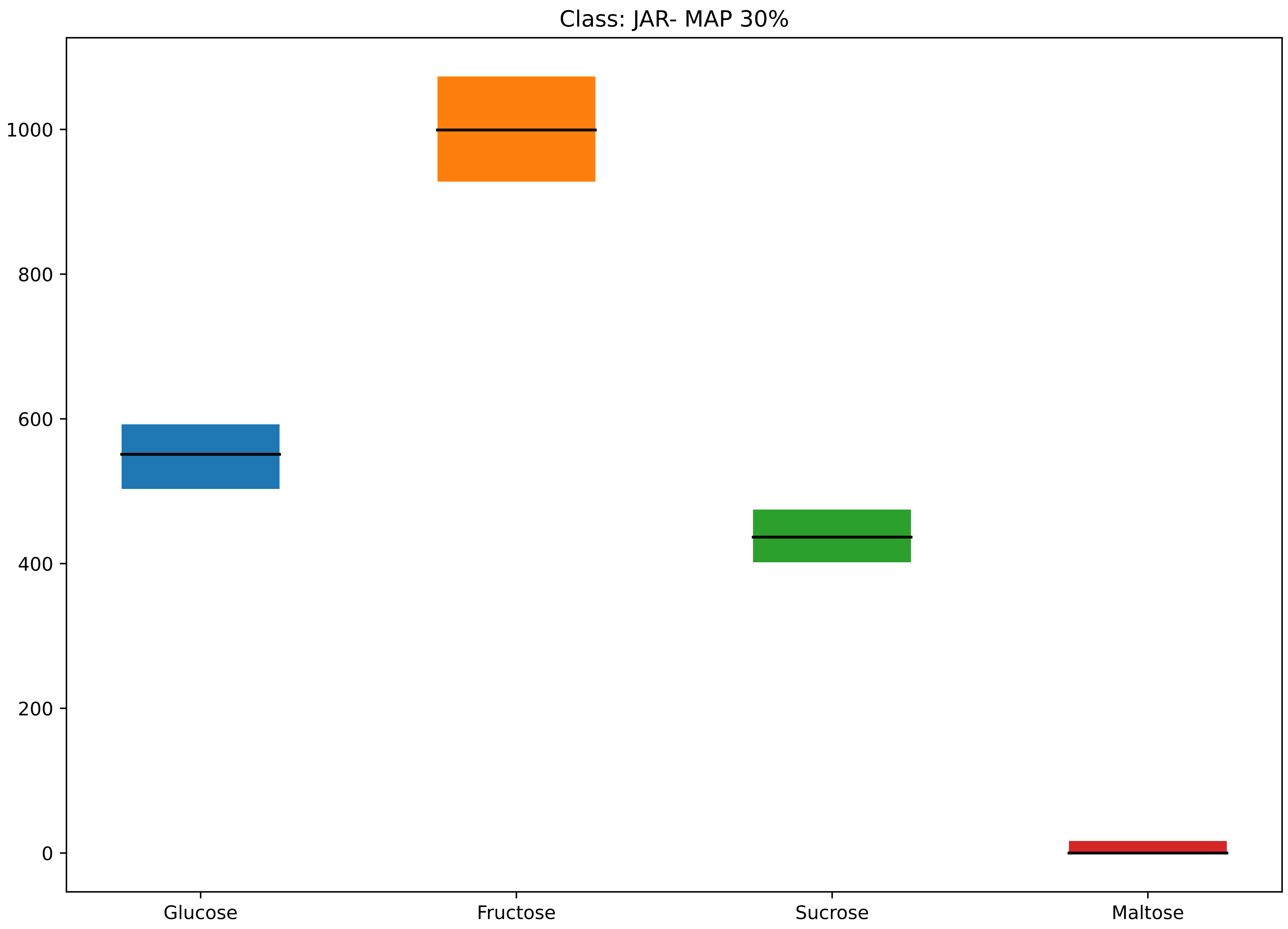

Class: JAR- MAP 30%
1000
800
600
400
200
0
Glucose
Fructose
Sucrose
Maltose

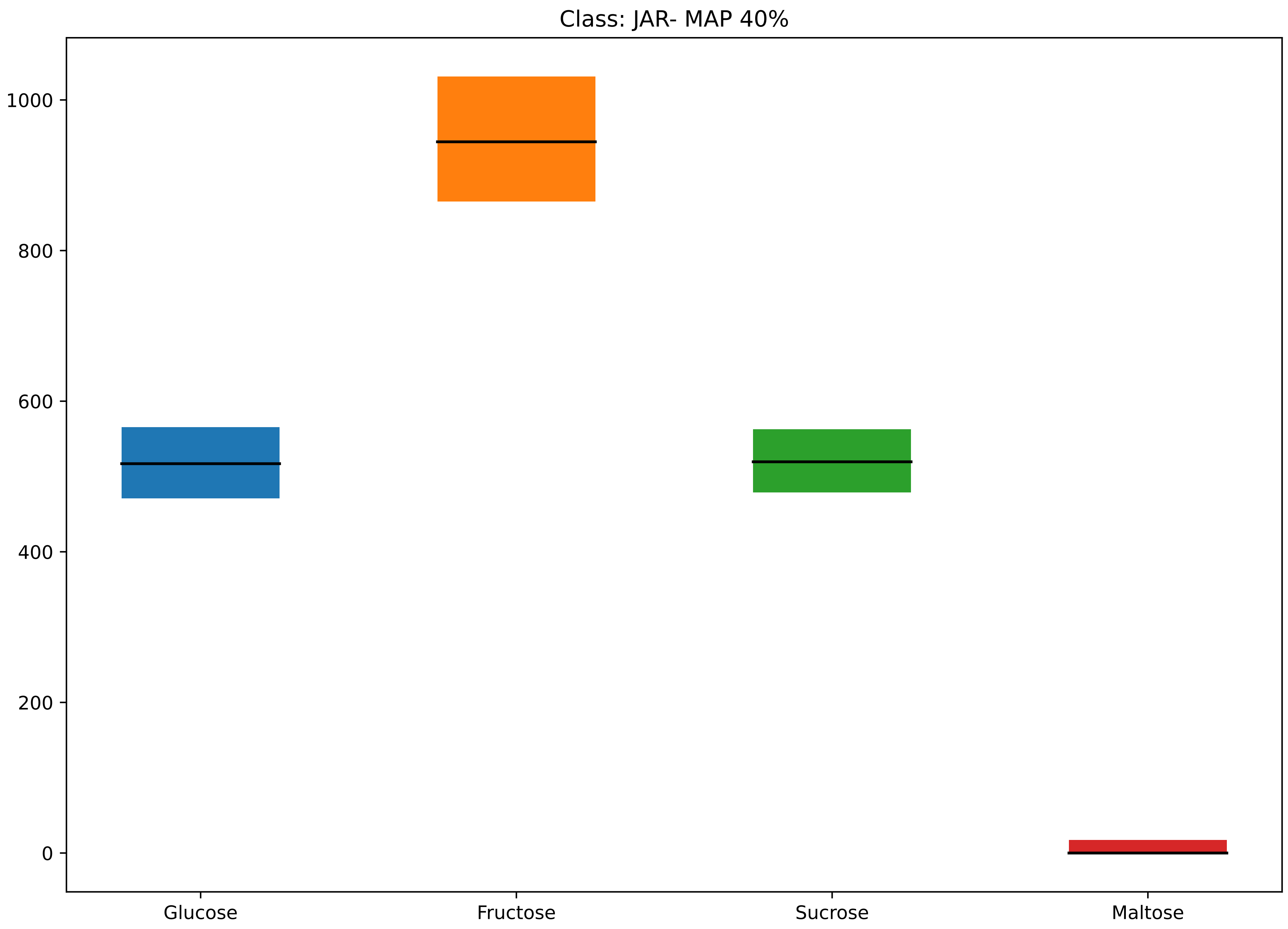

Class: JAR- MAP 40%
1000
800
600
400
200
0
Glucose
Fructose
Sucrose
Maltose

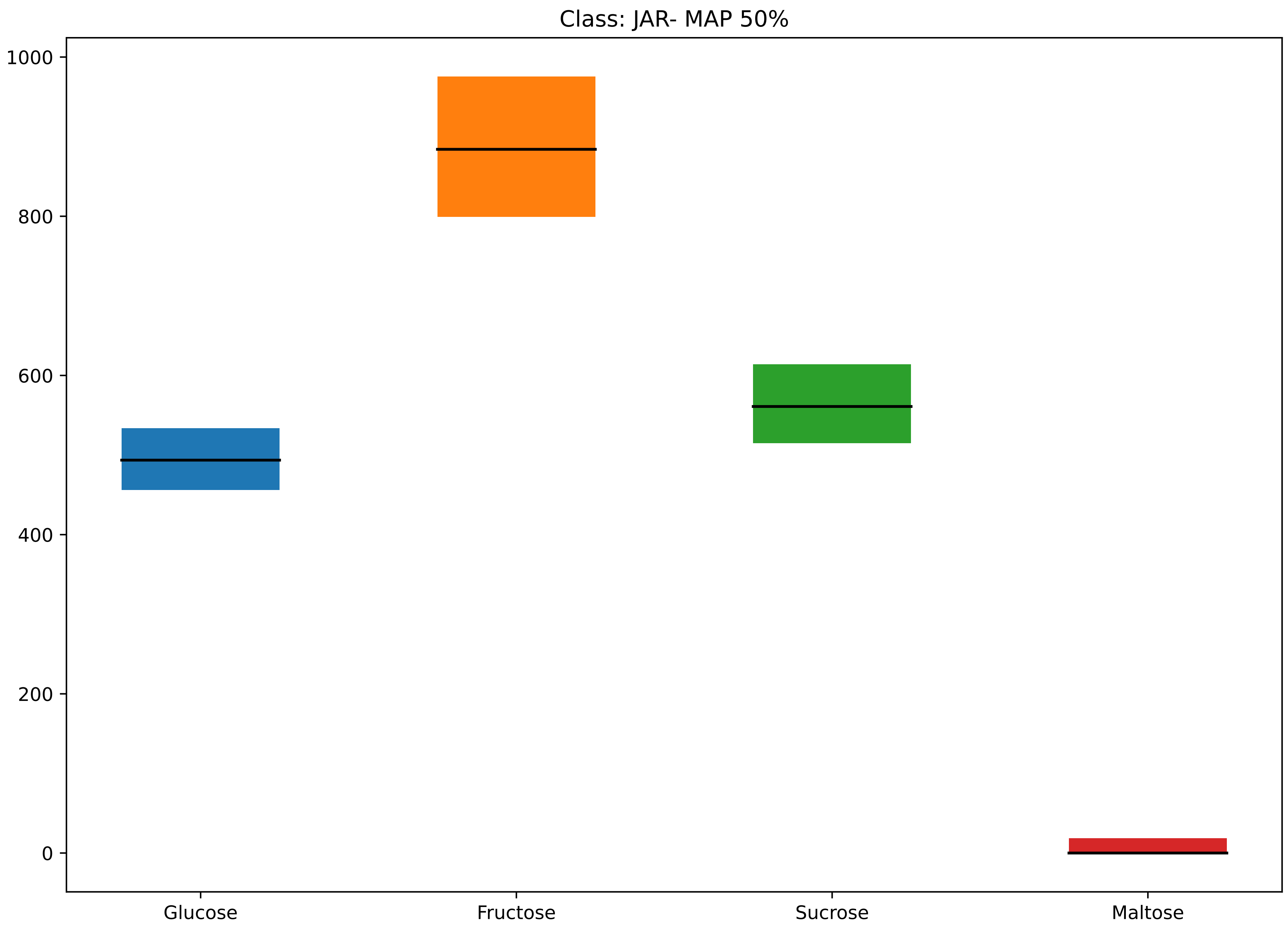

Class: JAR- MAP 50%
1000
800
600
400
200
0
Glucose
Fructose
Sucrose
Maltose

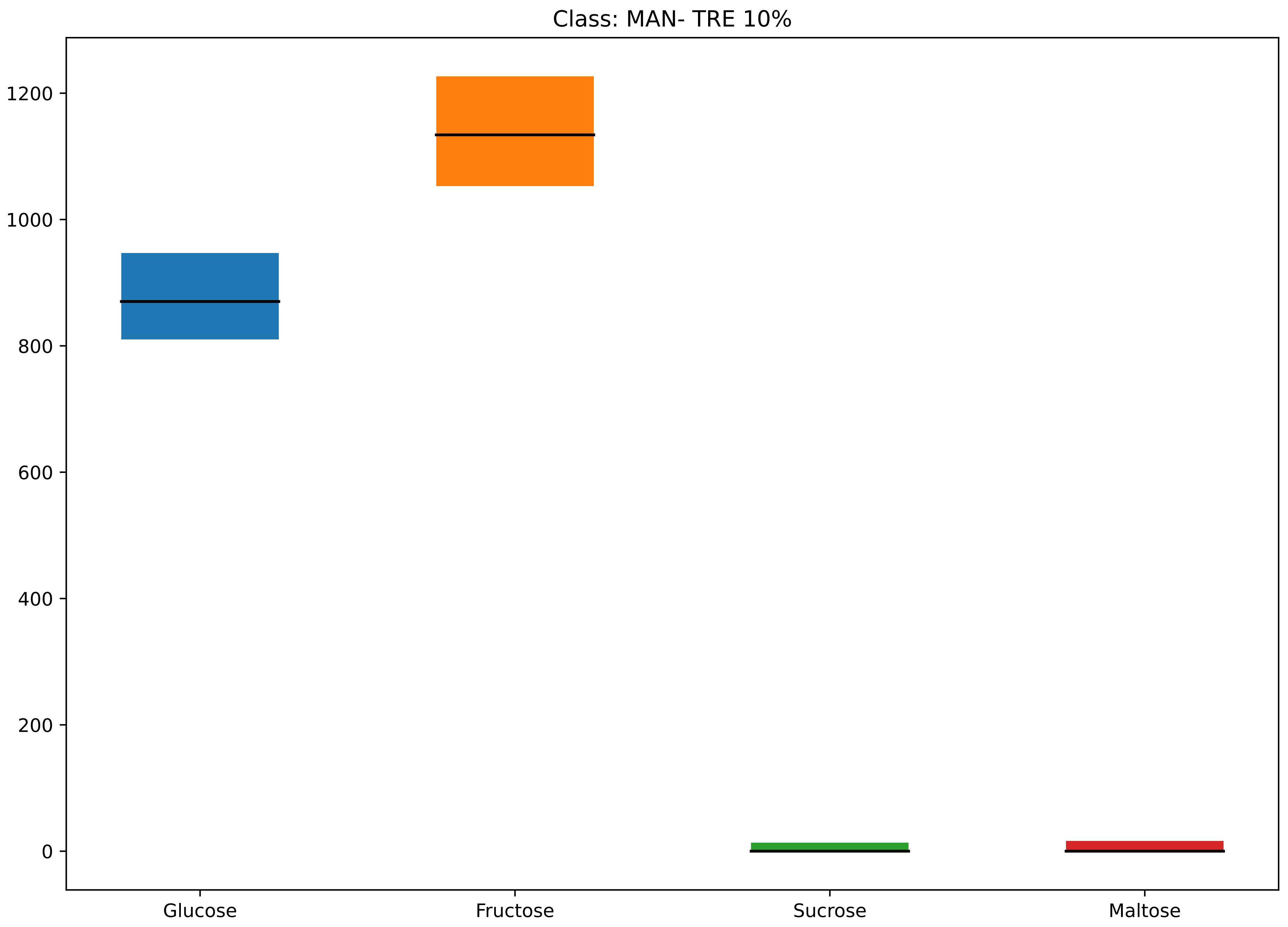

Class: MAN- TRE 10%
1200
1000
800
600
400
200
0
Glucose
Fructose
Sucrose
Maltose

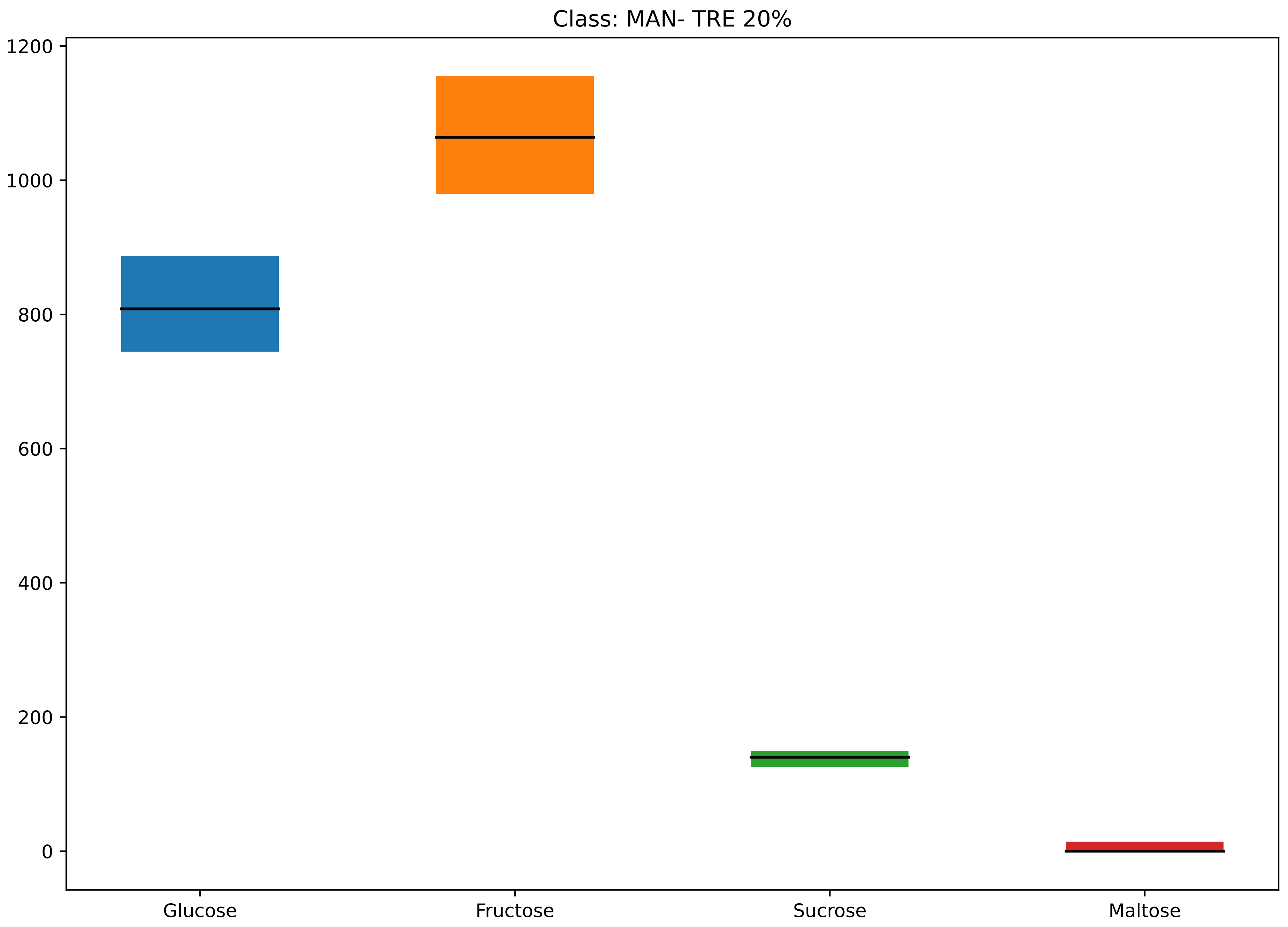

Class: MAN- TRE 20%
1200
1000
800
600
400
200
0
Glucose
Fructose
Sucrose
Maltose

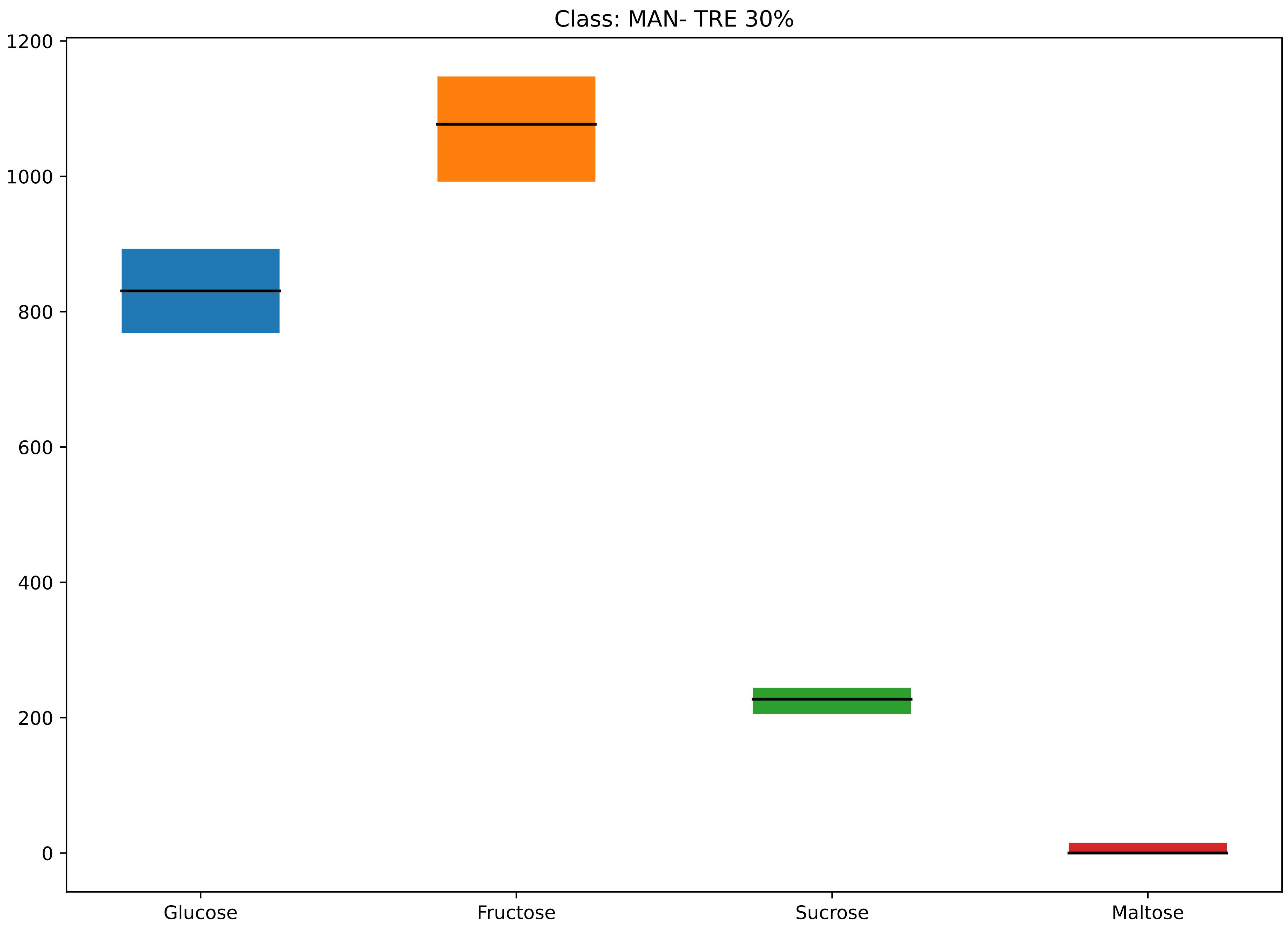

Class: MAN- TRE 30%
1200
1000
800
600
400
200
0
Glucose
Fructose
Sucrose
Maltose

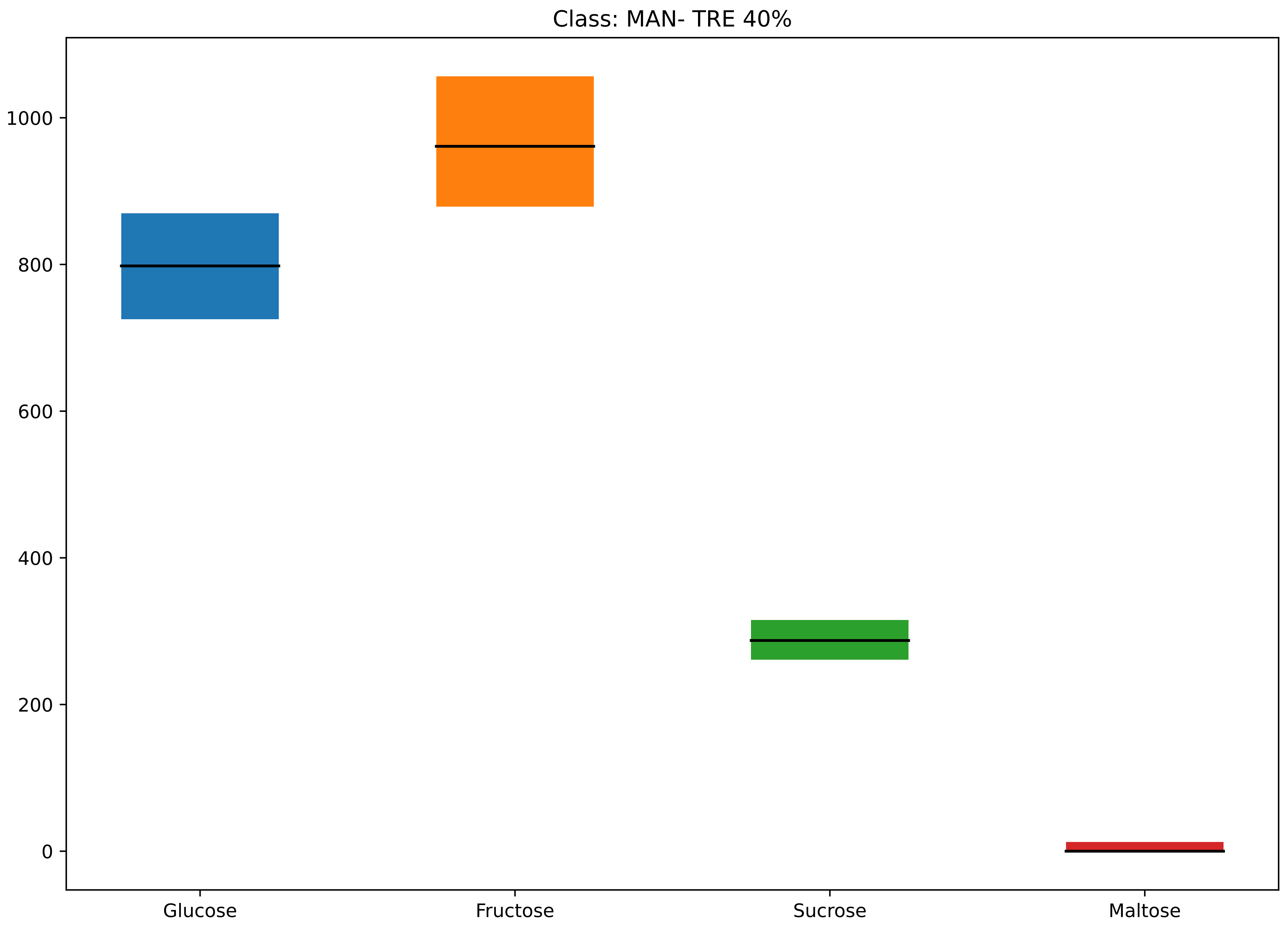

Class: MAN- TRE 40%
1000
800
600
400
200
0
Glucose
Fructose
Sucrose
Maltose

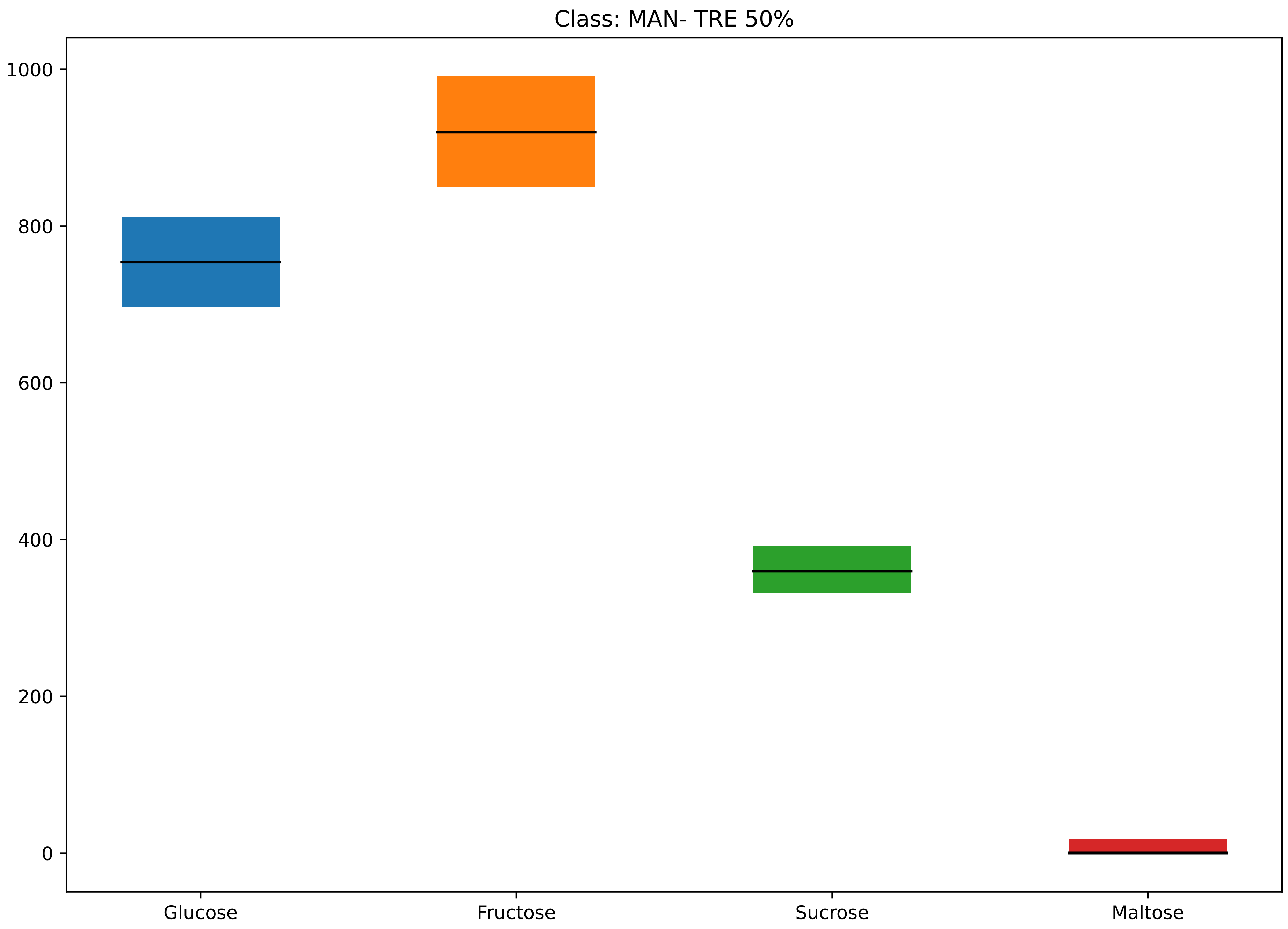
Class: MAN- TRE 50%
1000
800
600
400
200
0
Glucose
Fructose
Sucrose
Maltose

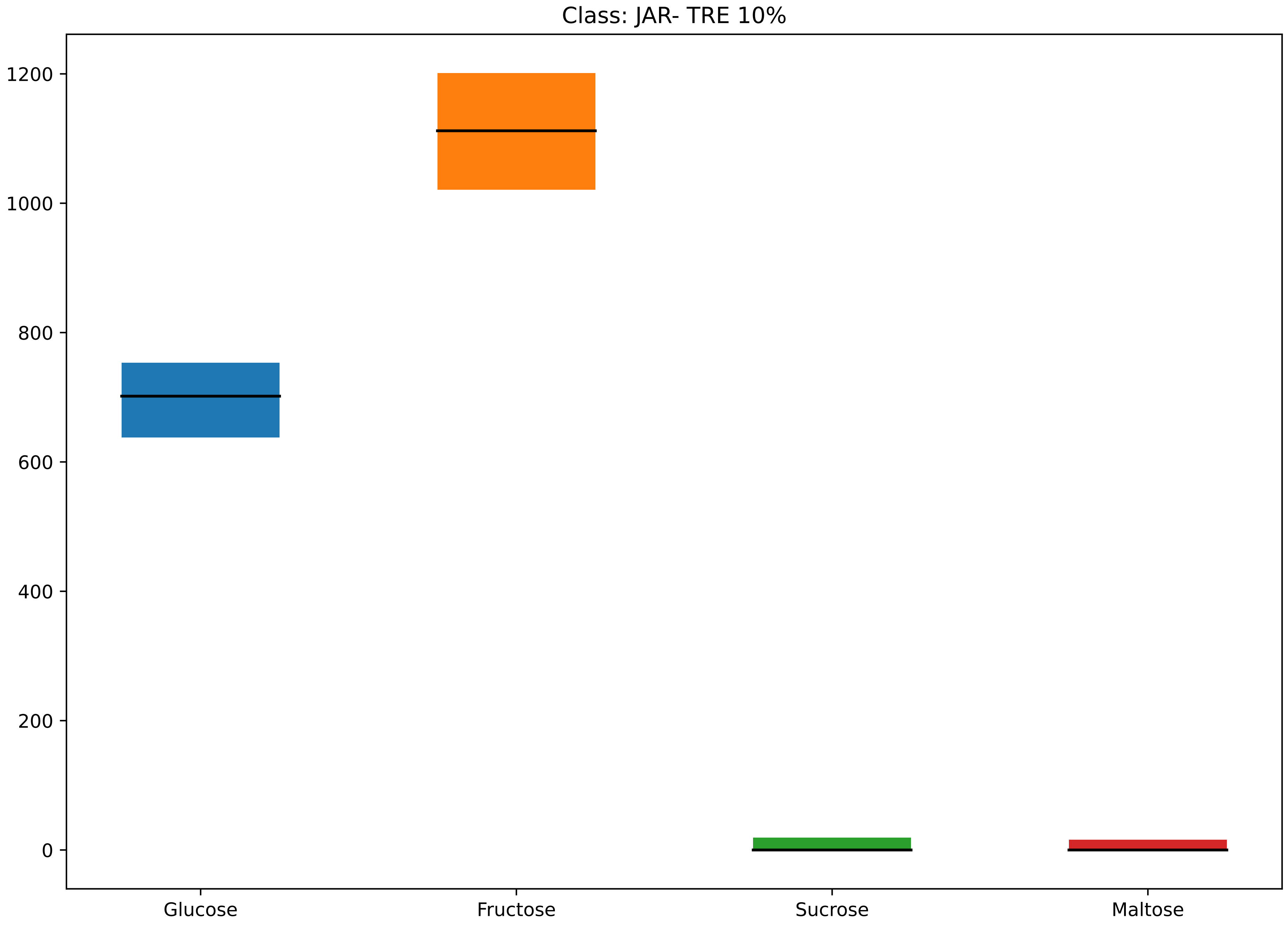
Class: JAR- TRE 10%
1200
1000
800
600
400
200
0
Glucose
Fructose
Sucrose
Maltose

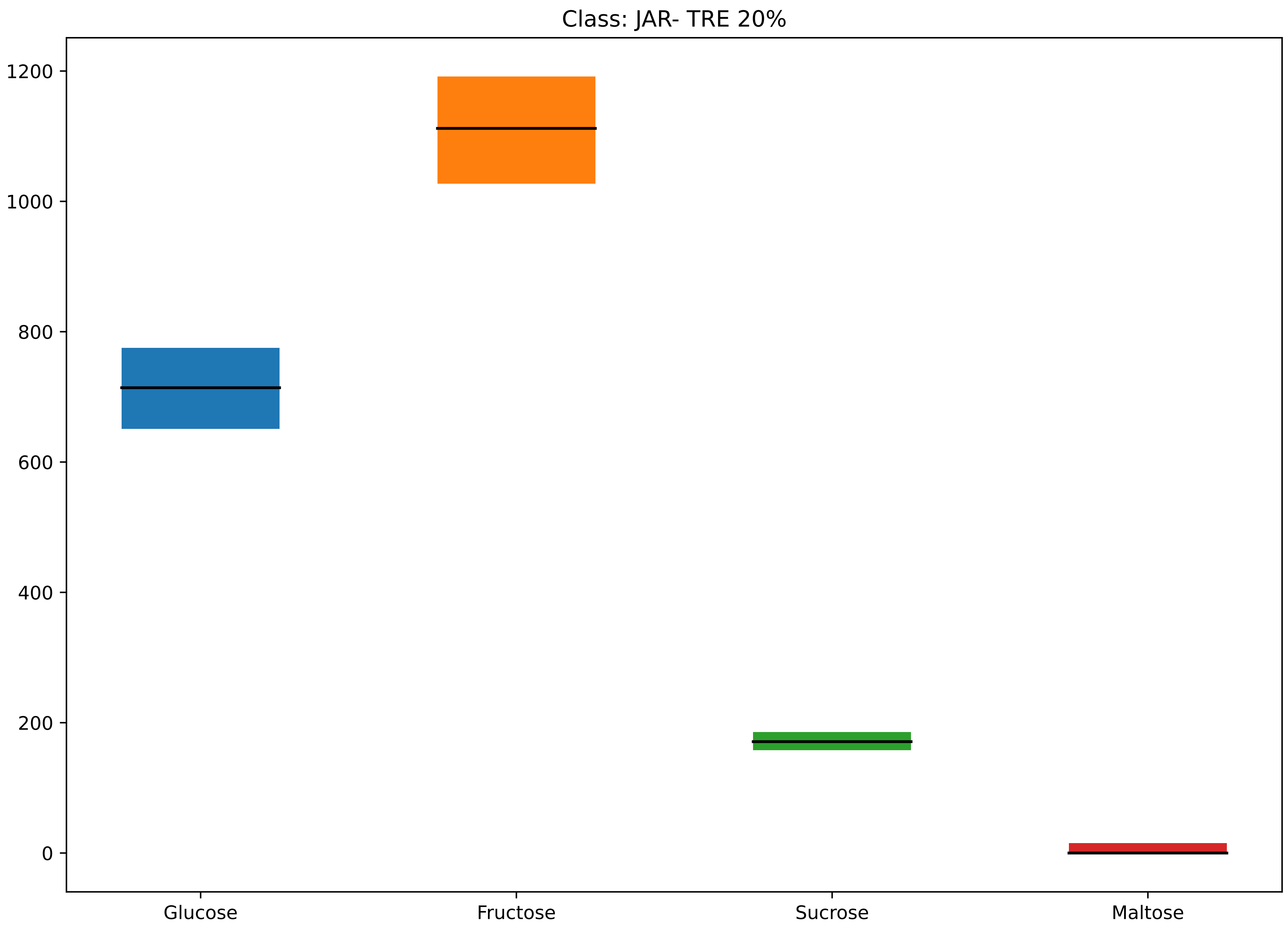
Class: JAR- TRE 20%
1200
1000
800
600
400
200
0
Glucose
Fructose
Sucrose
Maltose

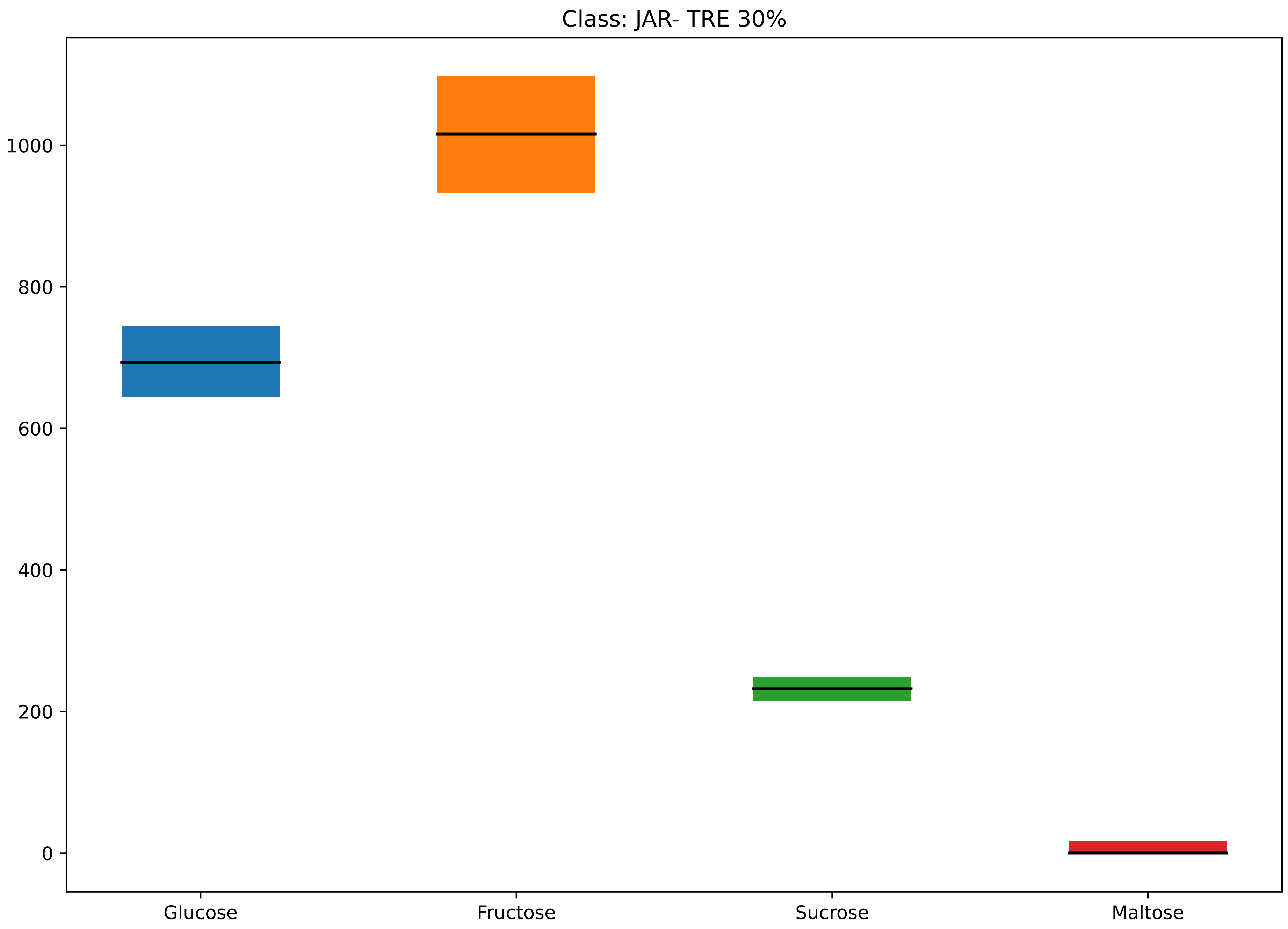
Class: JAR- TRE 30%
1000
800
600
400
200
0
Glucose
Fructose
Sucrose
Maltose

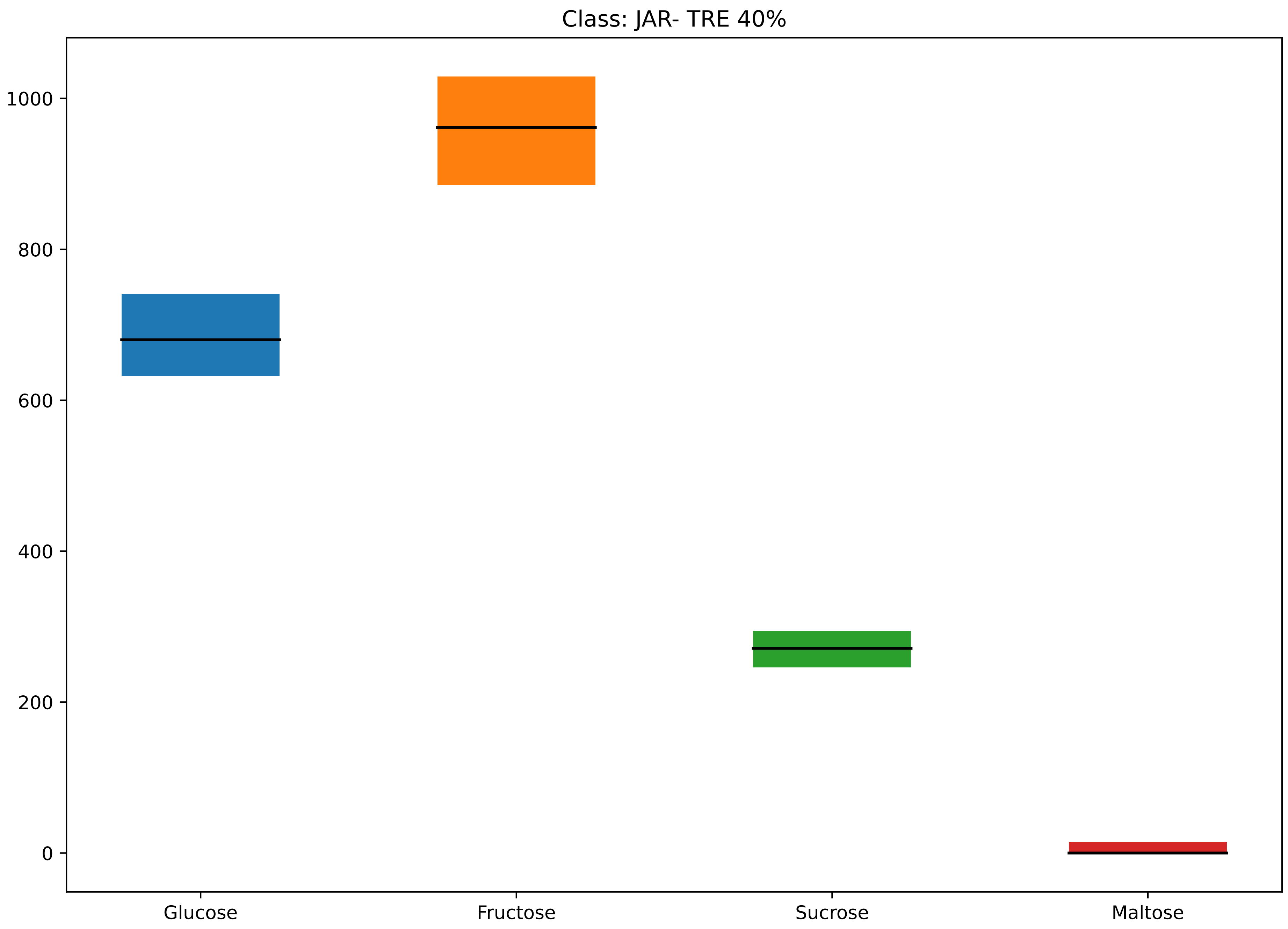

Class: JAR- TRE 40%
1000
800
600
400
200
0
Glucose
Fructose
Sucrose
Maltose

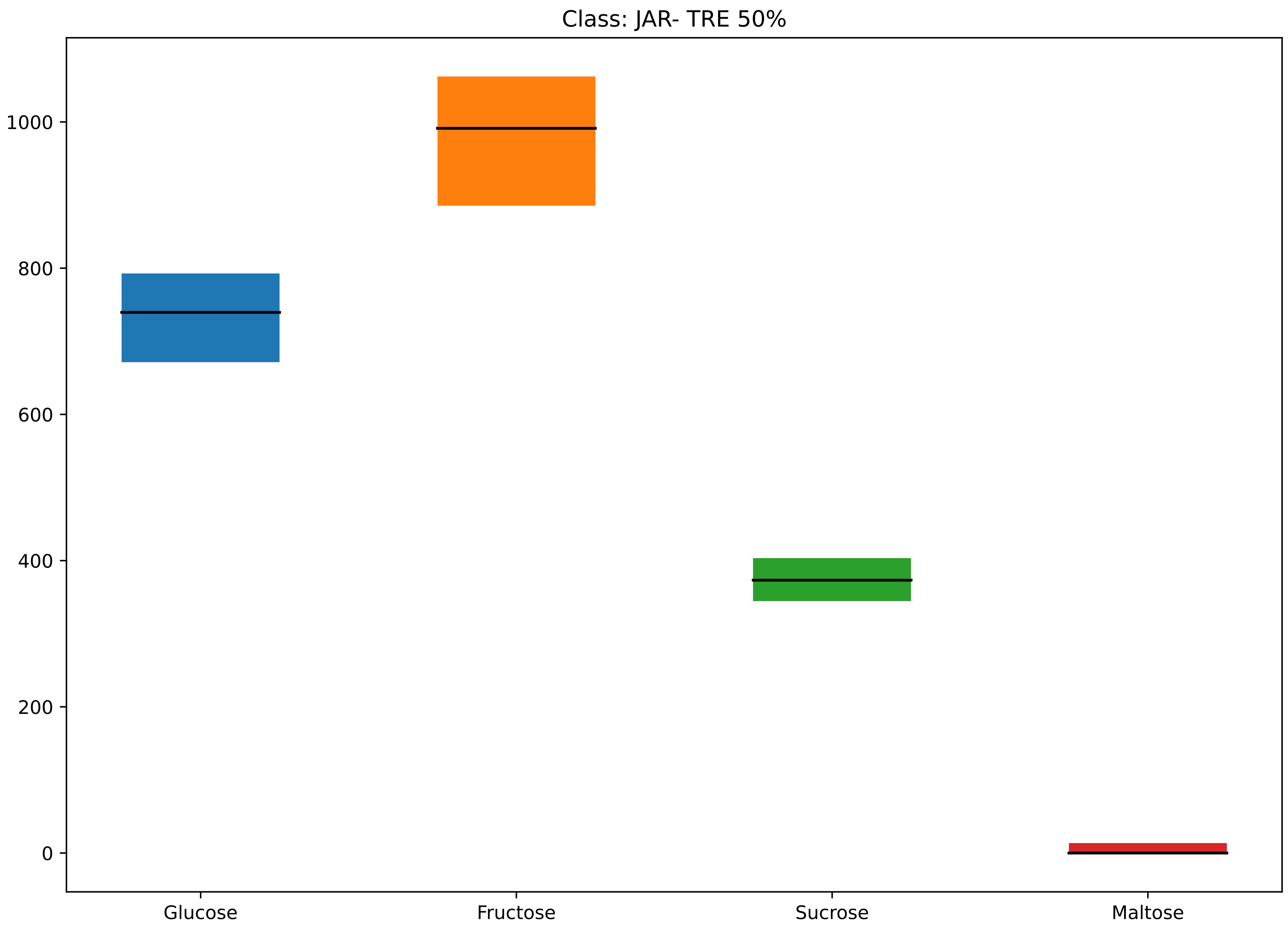
Class: JAR- TRE 50%
1000
800
600
400
200
0
Glucose
Fructose
Sucrose
Maltose

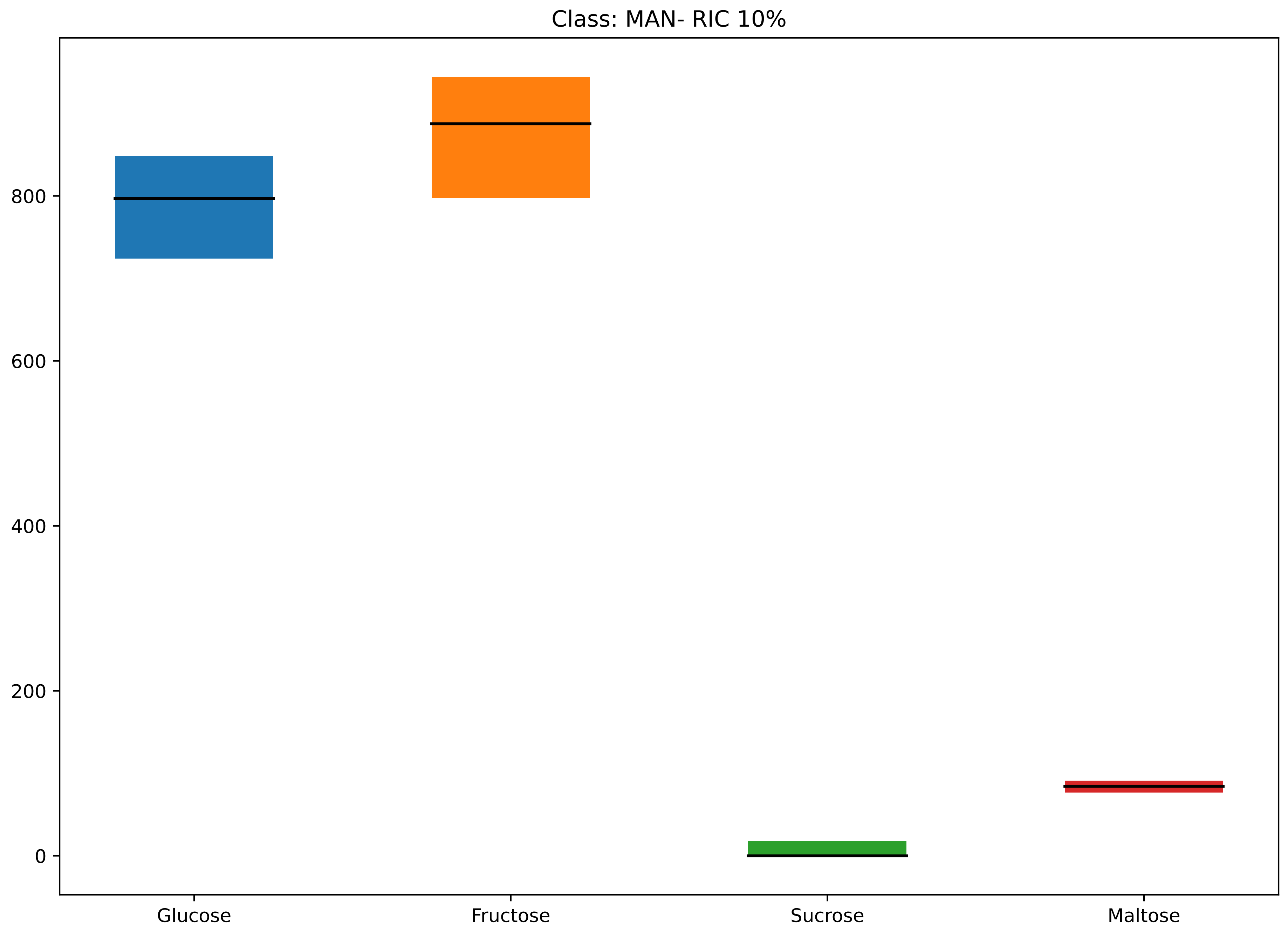
Class: MAN- RIC 10%
800
600
400
200
0
Glucose
Fructose
Sucrose
Maltose

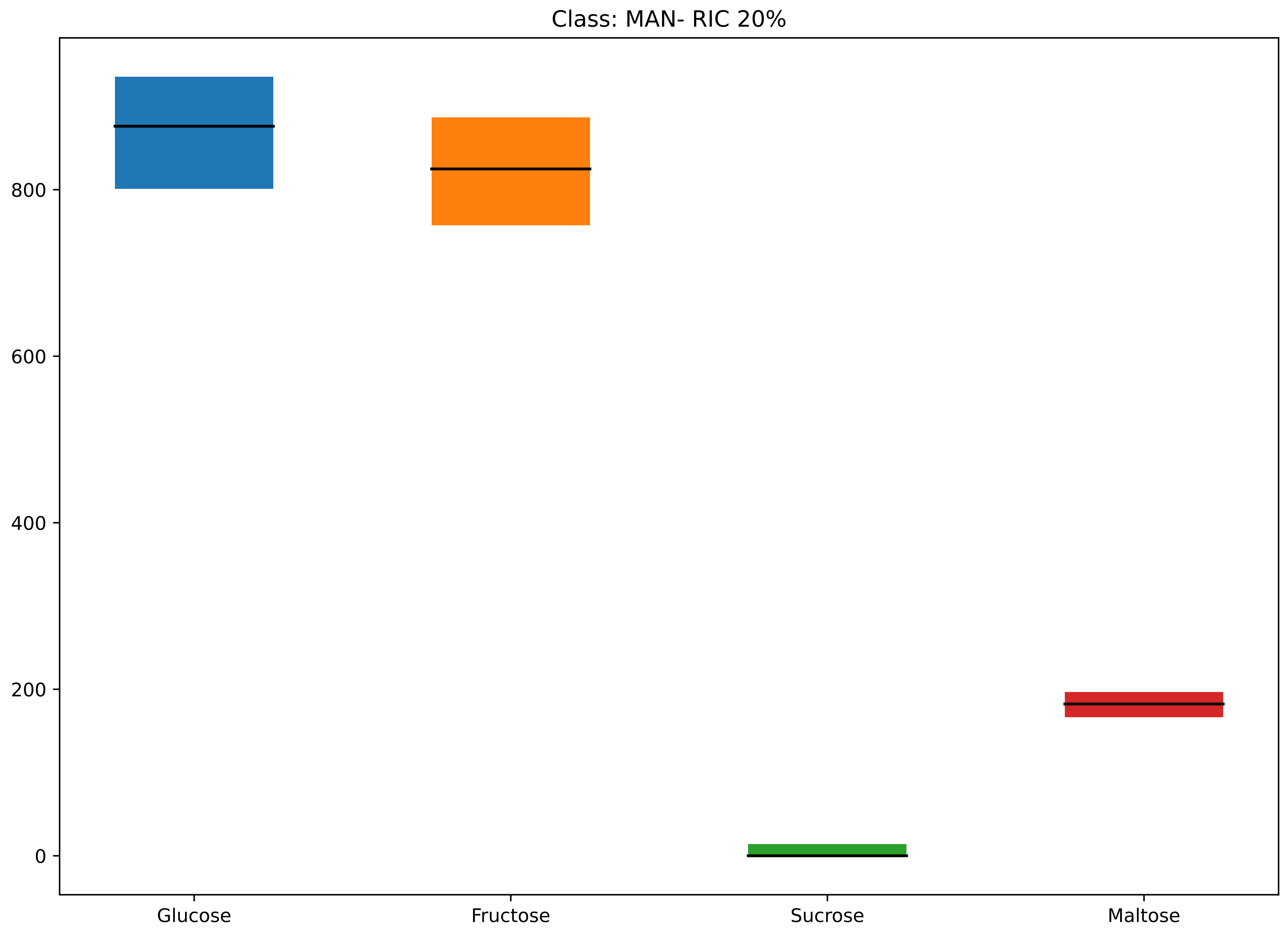
Class: MAN- RIC 20%
800
600
400
200
0
Glucose
Fructose
Sucrose
Maltose

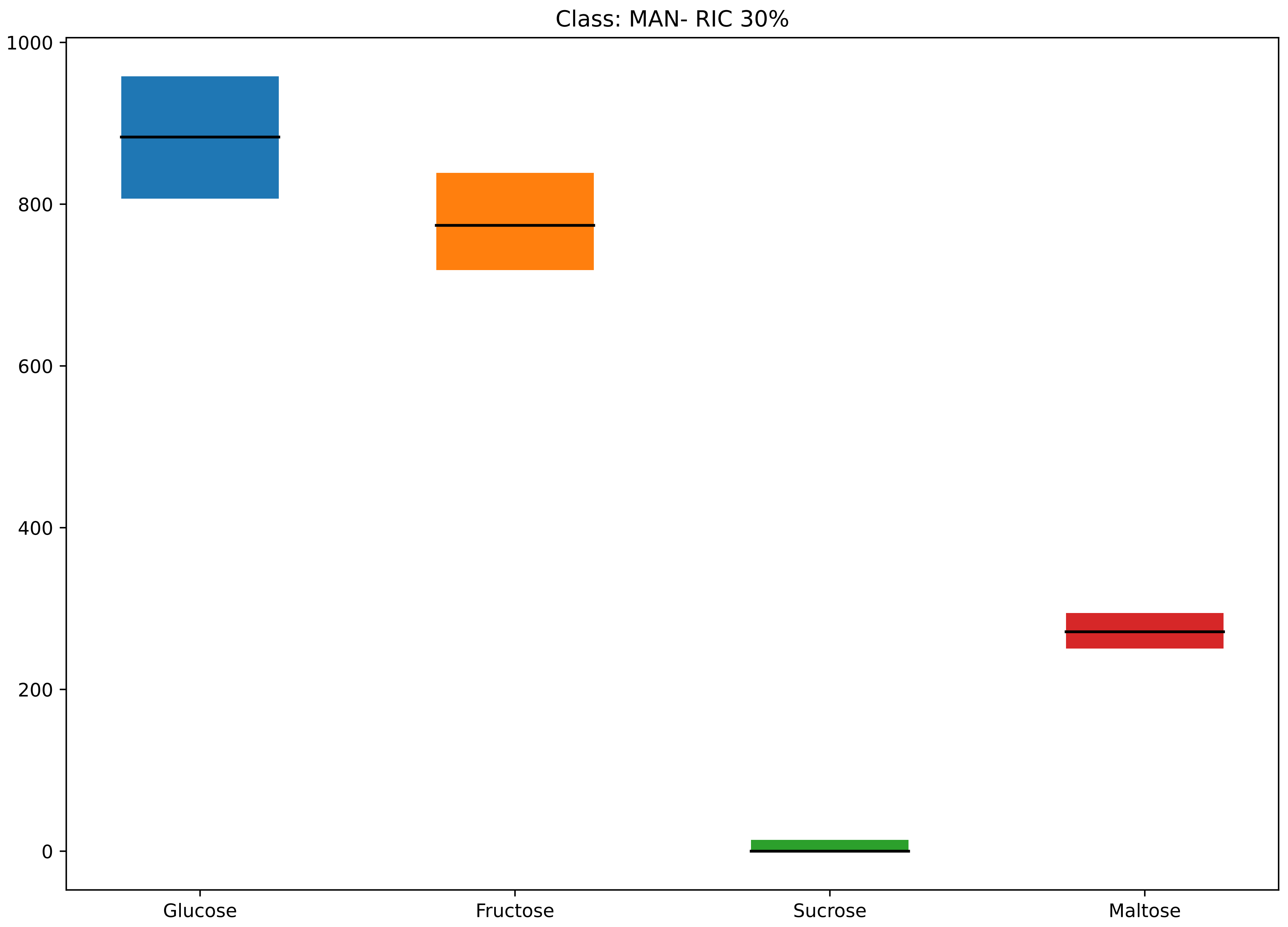

Class: MAN- RIC 30%
1000
800
600
400
200
0
Glucose
Fructose
Sucrose
Maltose

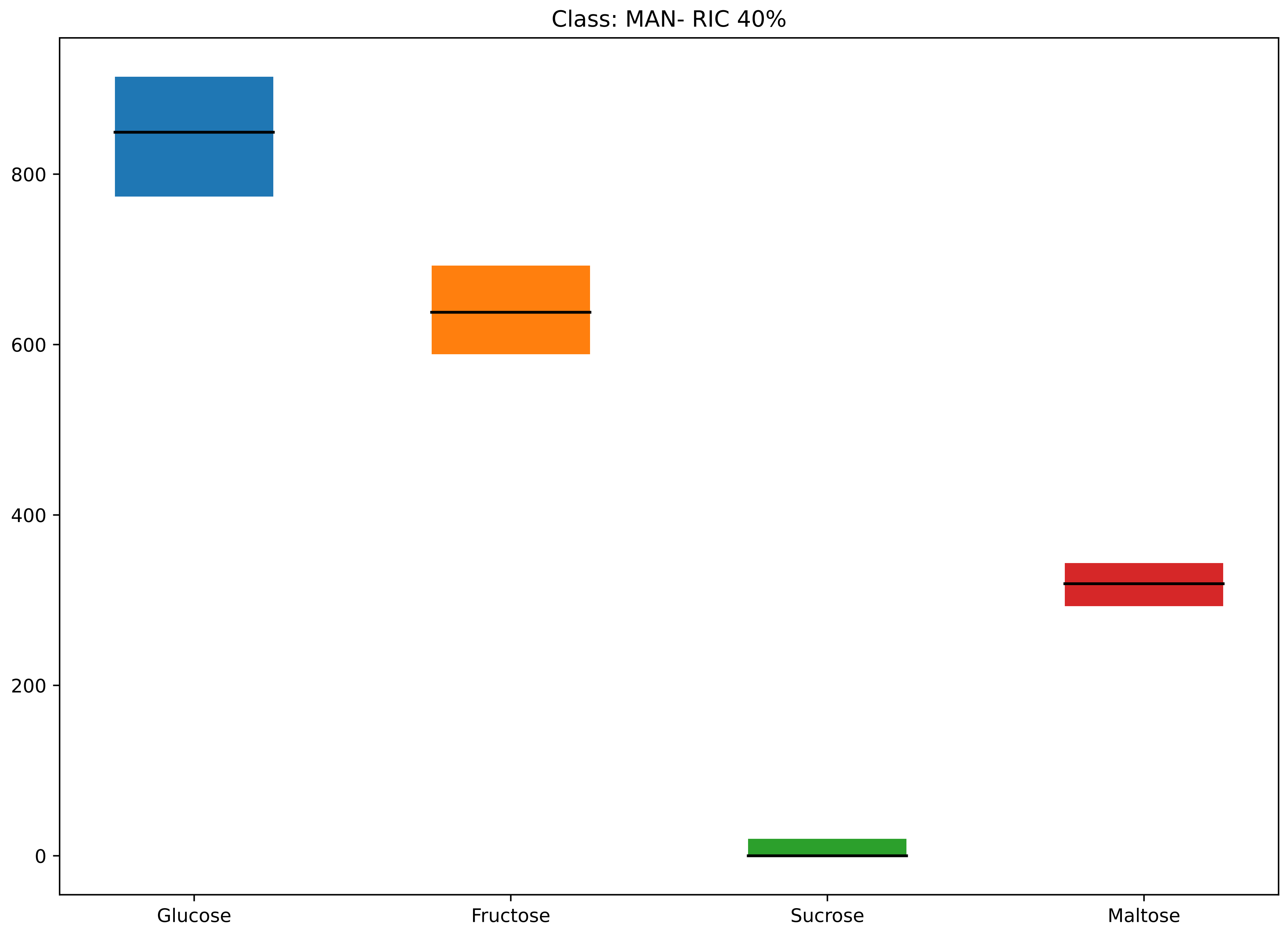

Class: MAN- RIC 40%
800
600
400
200
0
Glucose
Fructose
Sucrose
Maltose

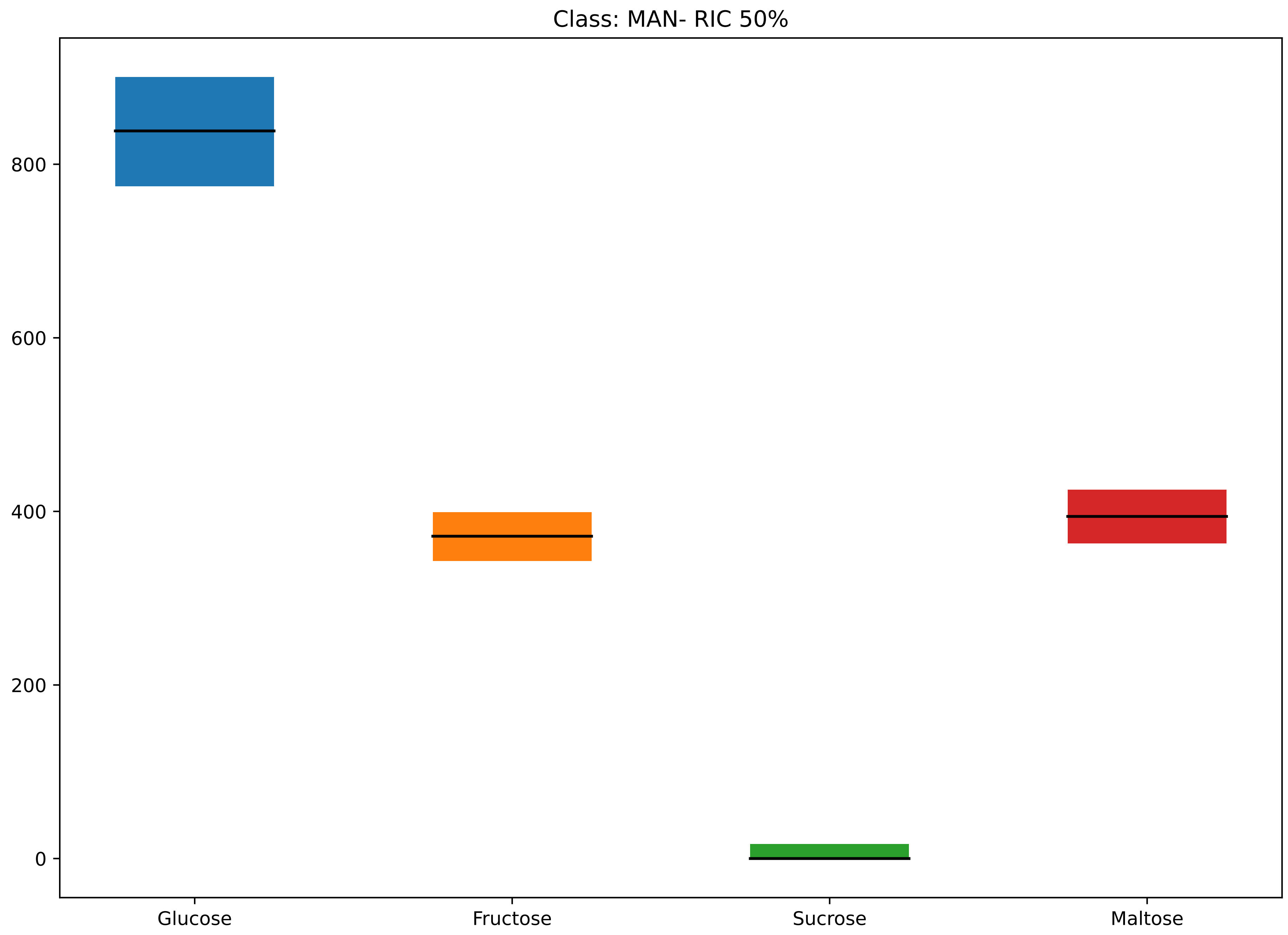
Class: MAN- RIC 50%
800
600
400
200
0
Glucose
Fructose
Sucrose
Maltose

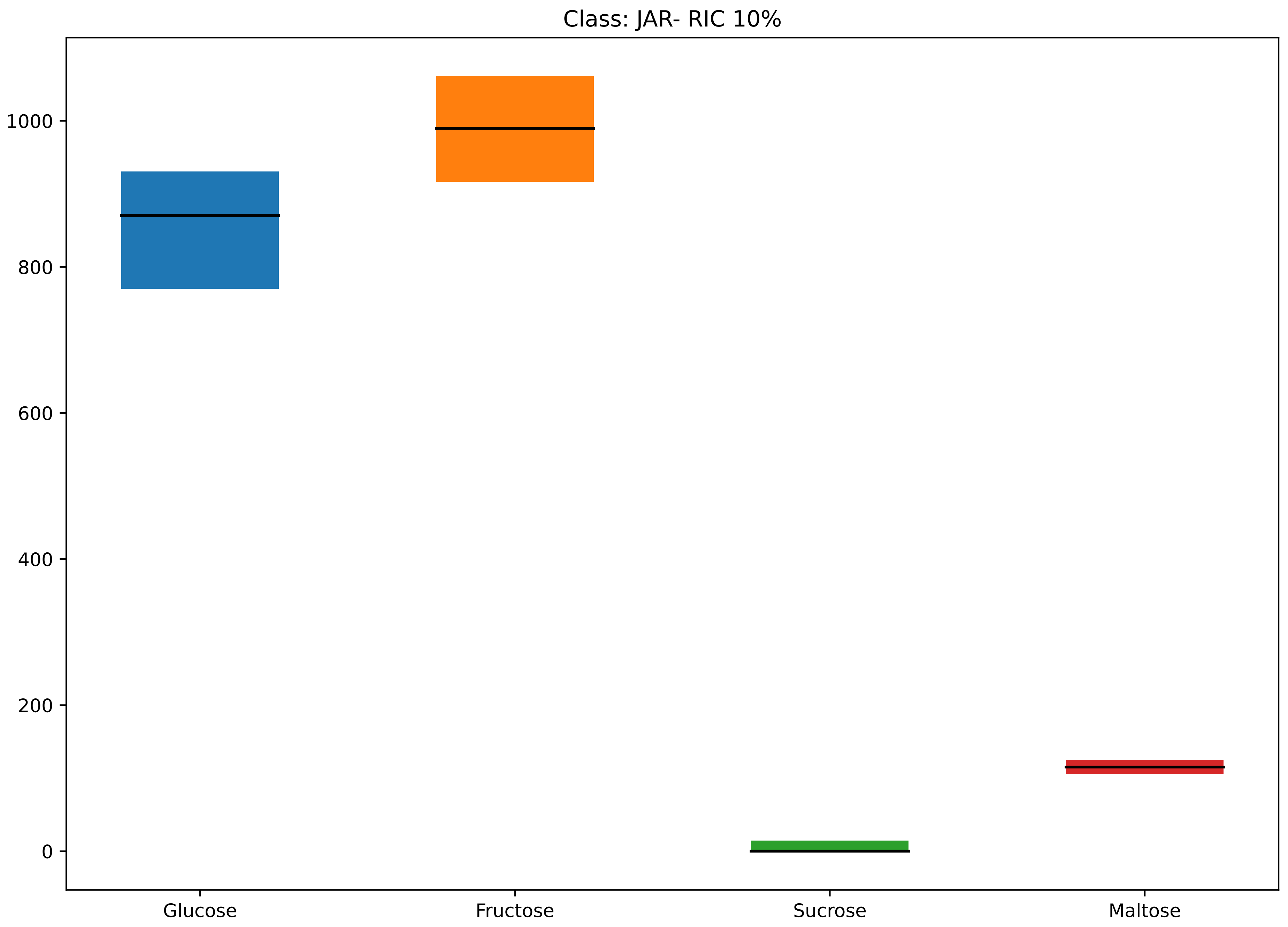

Class: JAR- RIC 10%
1000
800
600
400
200
0
Glucose
Fructose
Sucrose
Maltose

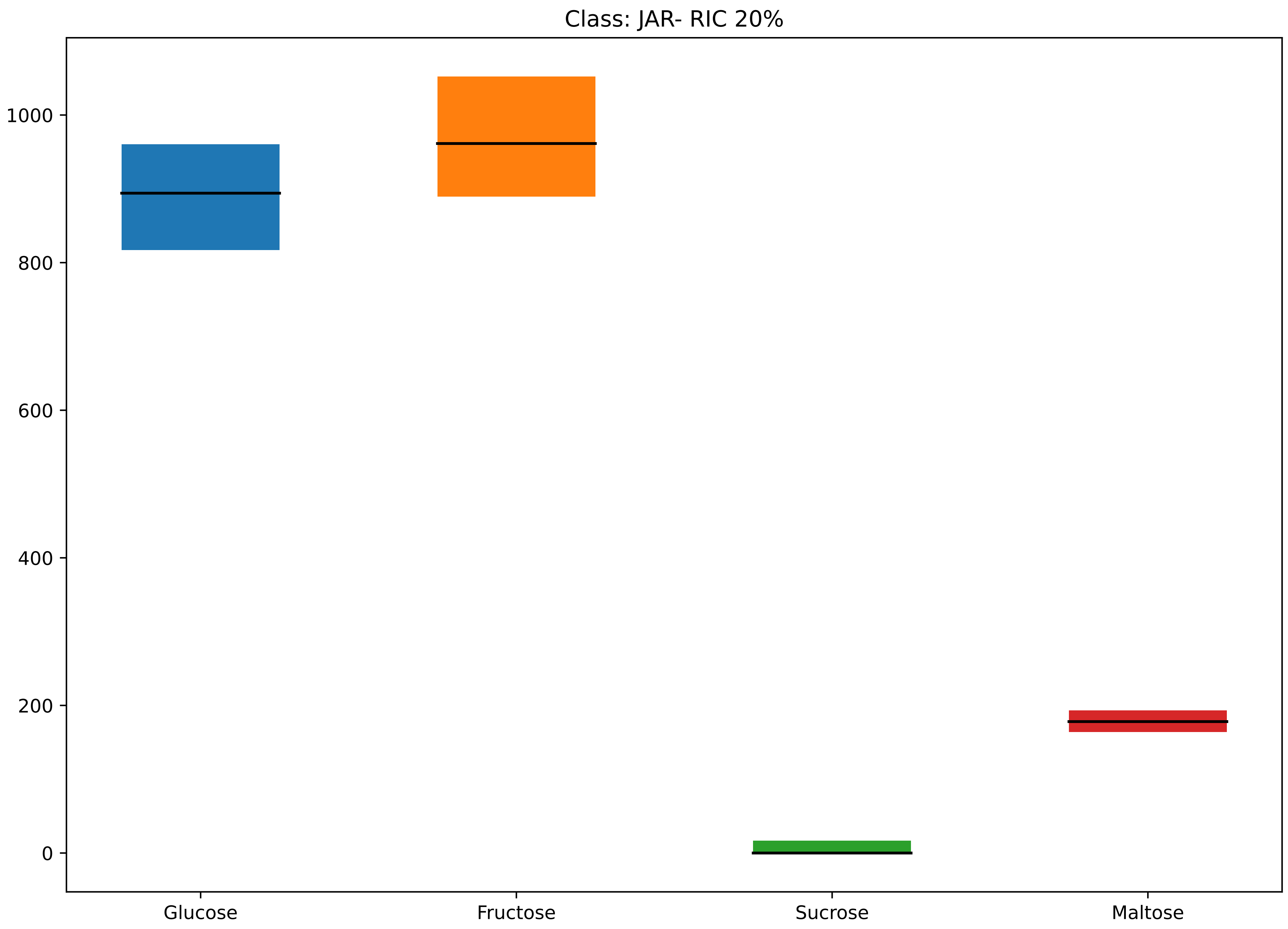

Class: JAR- RIC 20%
1000
800
600
400
200
0
Glucose
Fructose
Sucrose
Maltose

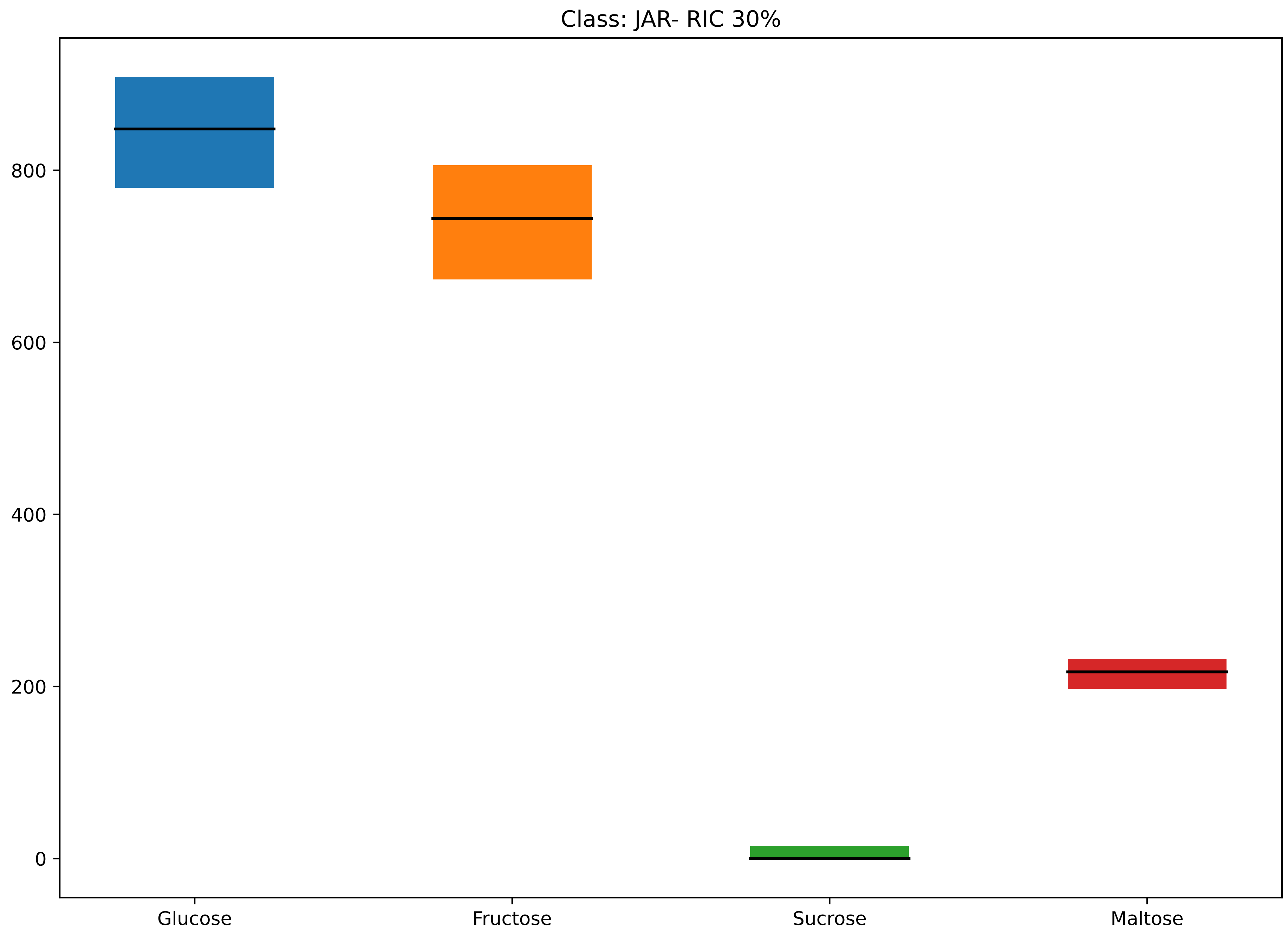
Class: JAR- RIC 30%
800
600
400
200
0
Glucose
Fructose
Sucrose
Maltose

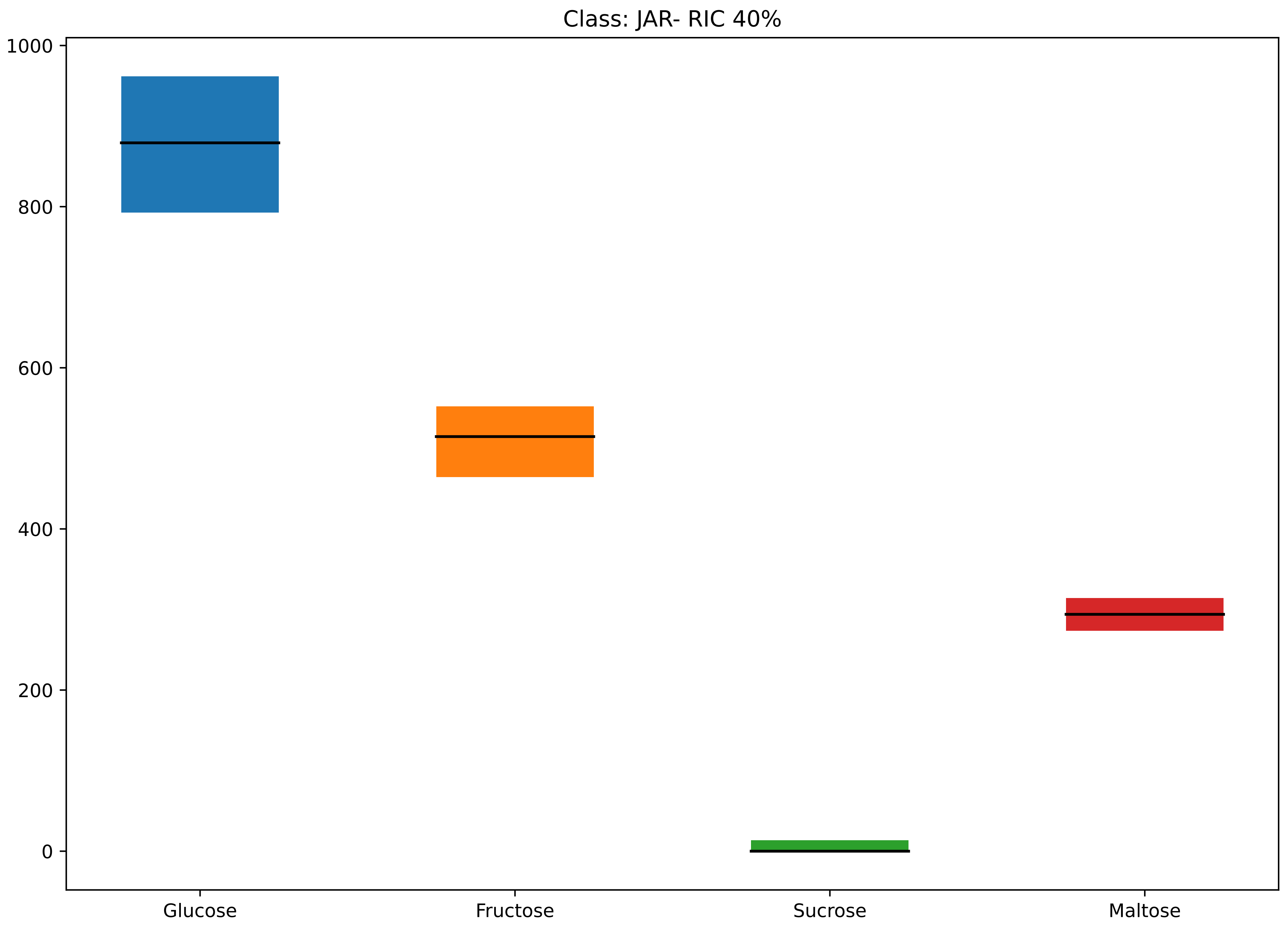

Class: JAR- RIC 40%
1000
800
600
400
200
0
Glucose
Fructose
Sucrose
Maltose

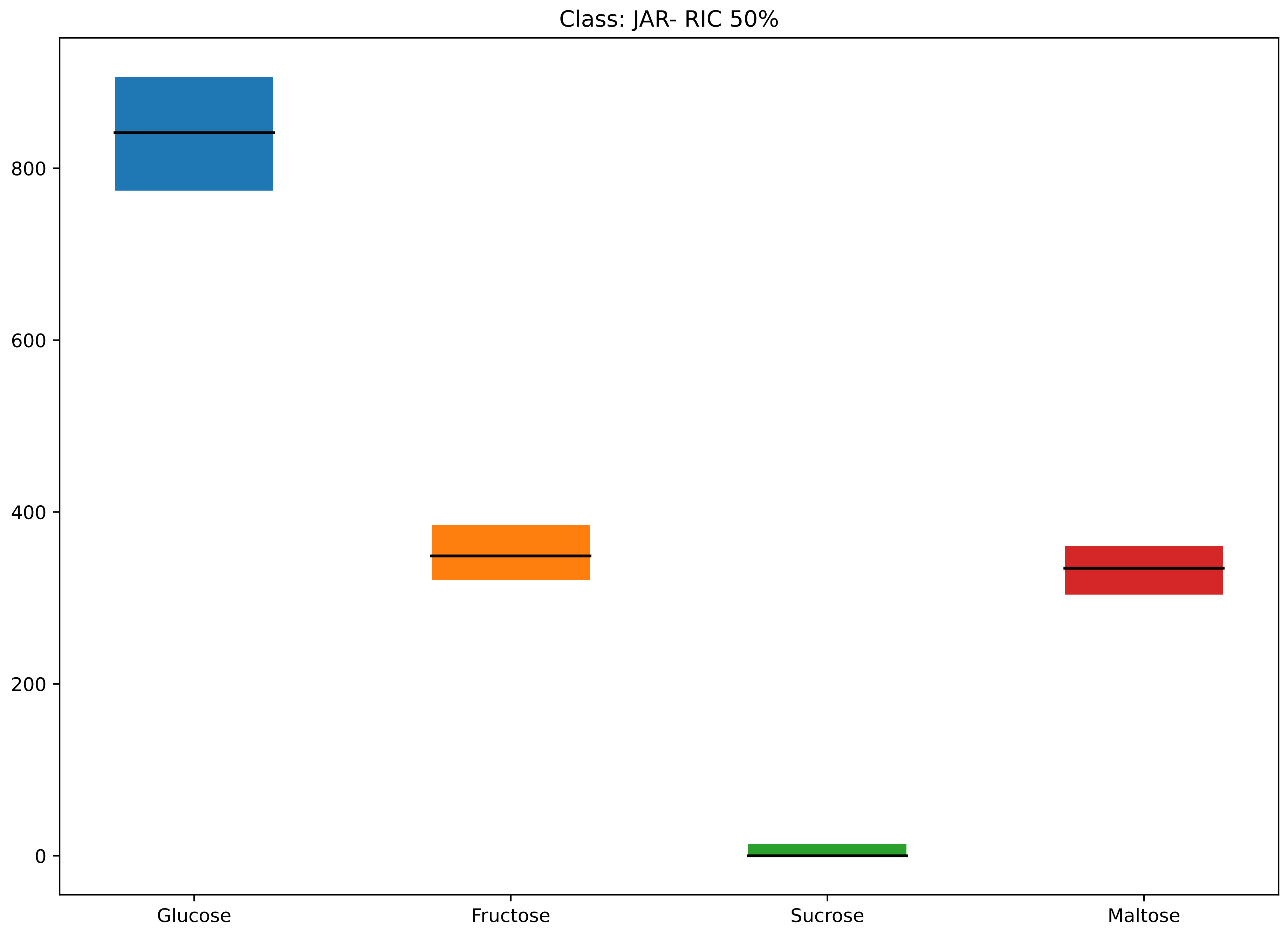

Class: JAR- RIC 50%
800
600
400
200
0
Glucose
Fructose
Sucrose
Maltose
