# Supplementary material for: Detection of syrup adulterants in manuka and jarrah honey using HPTLC-multivariate data analysis
Source: PeerJ. 2021 Sep 22;9:e12186. doi: 10.7717/peerj.12186 (PMC8464195; doi:10.7717/peerj.12186)
Supplement: Supplemental Information 3 [file peerj-09-12186-s003.pdf]

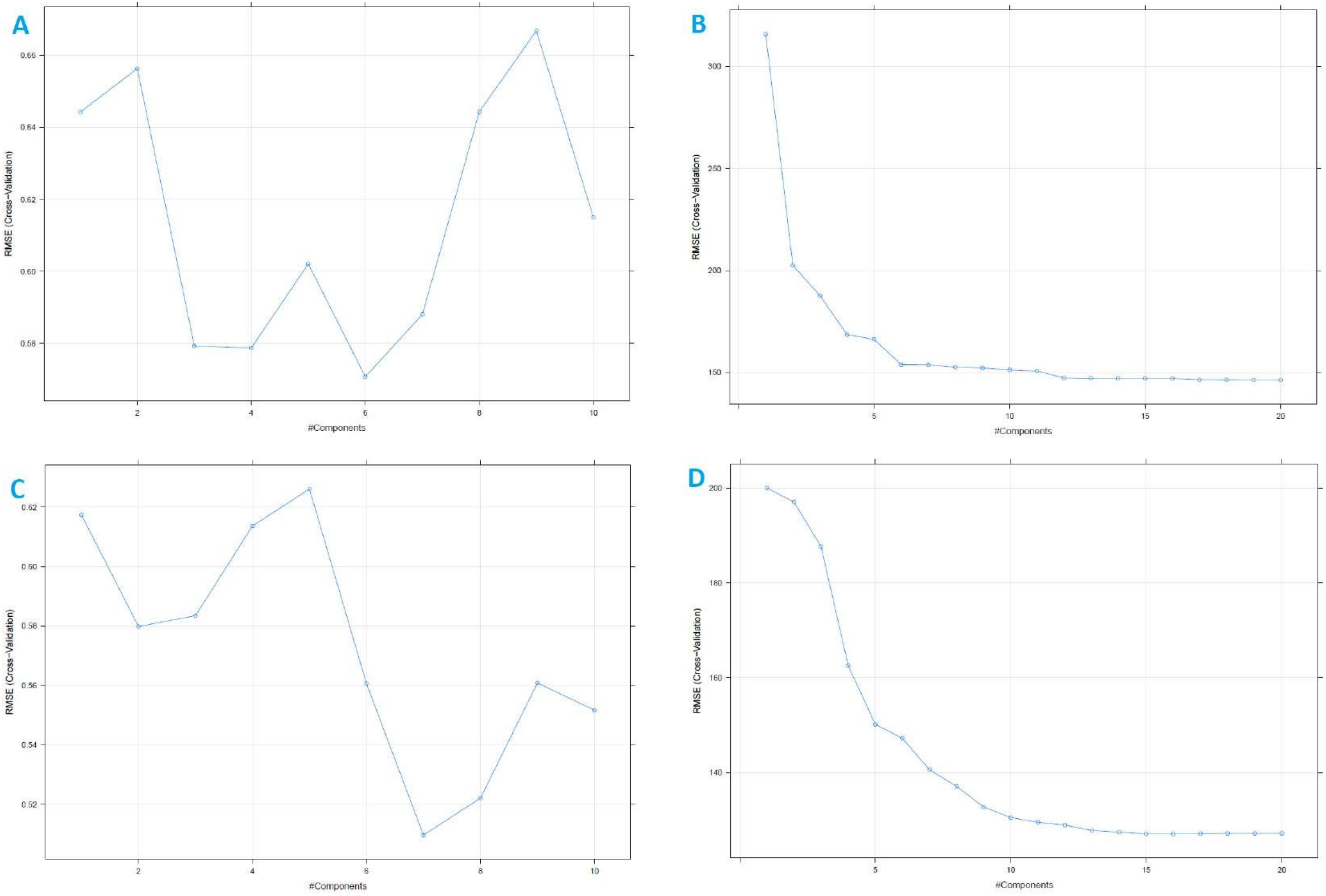

Fructose: PCR - Standardized data (a), PCR - Augmented data (b), PLS - Standardized data (c), and PLS - Augmented data (d)

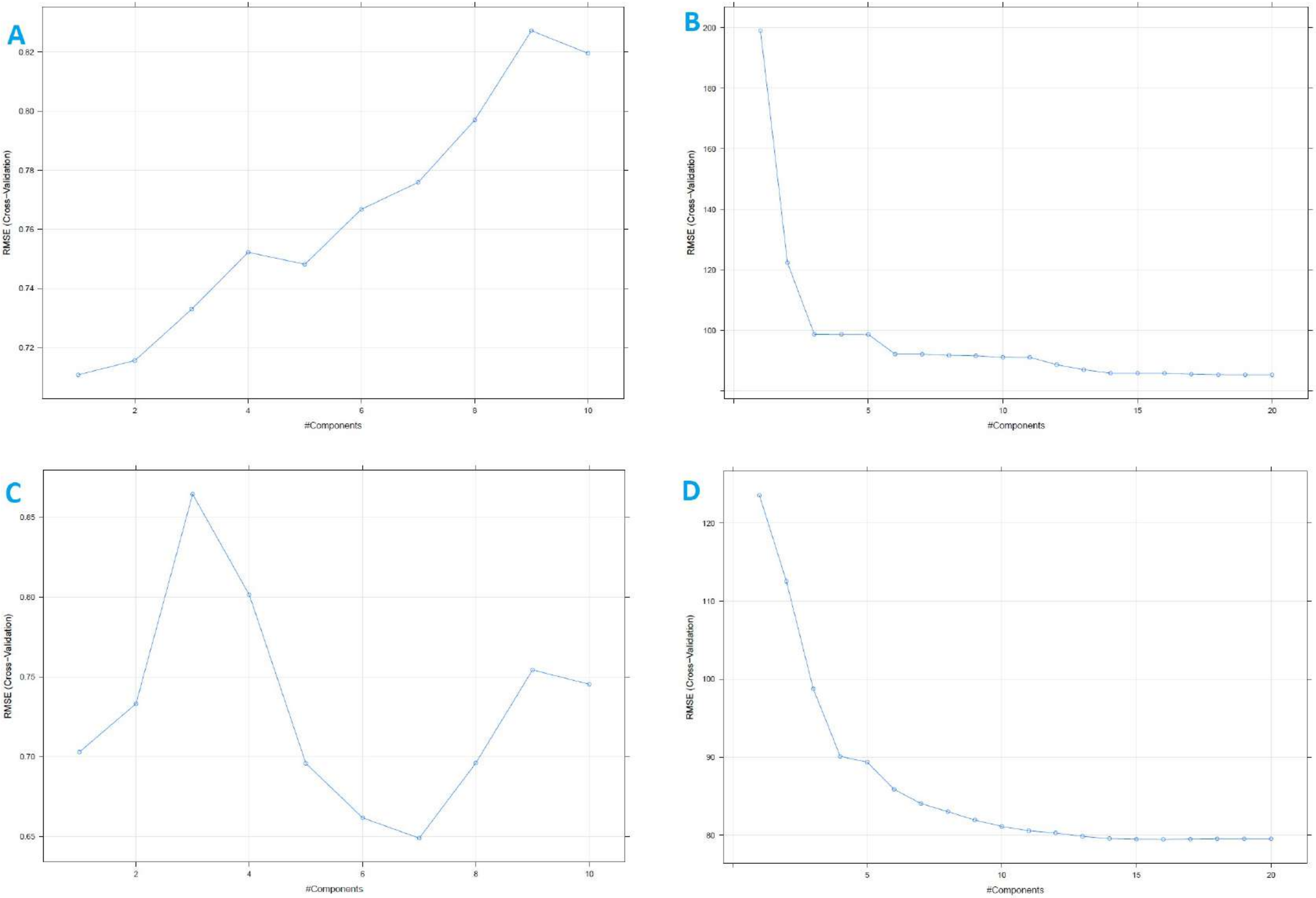

Maltose: PCR - Standardized data (a), PCR - Augmented data (b), PLS - Standardized data (c), and PLS - Augmented data (d)

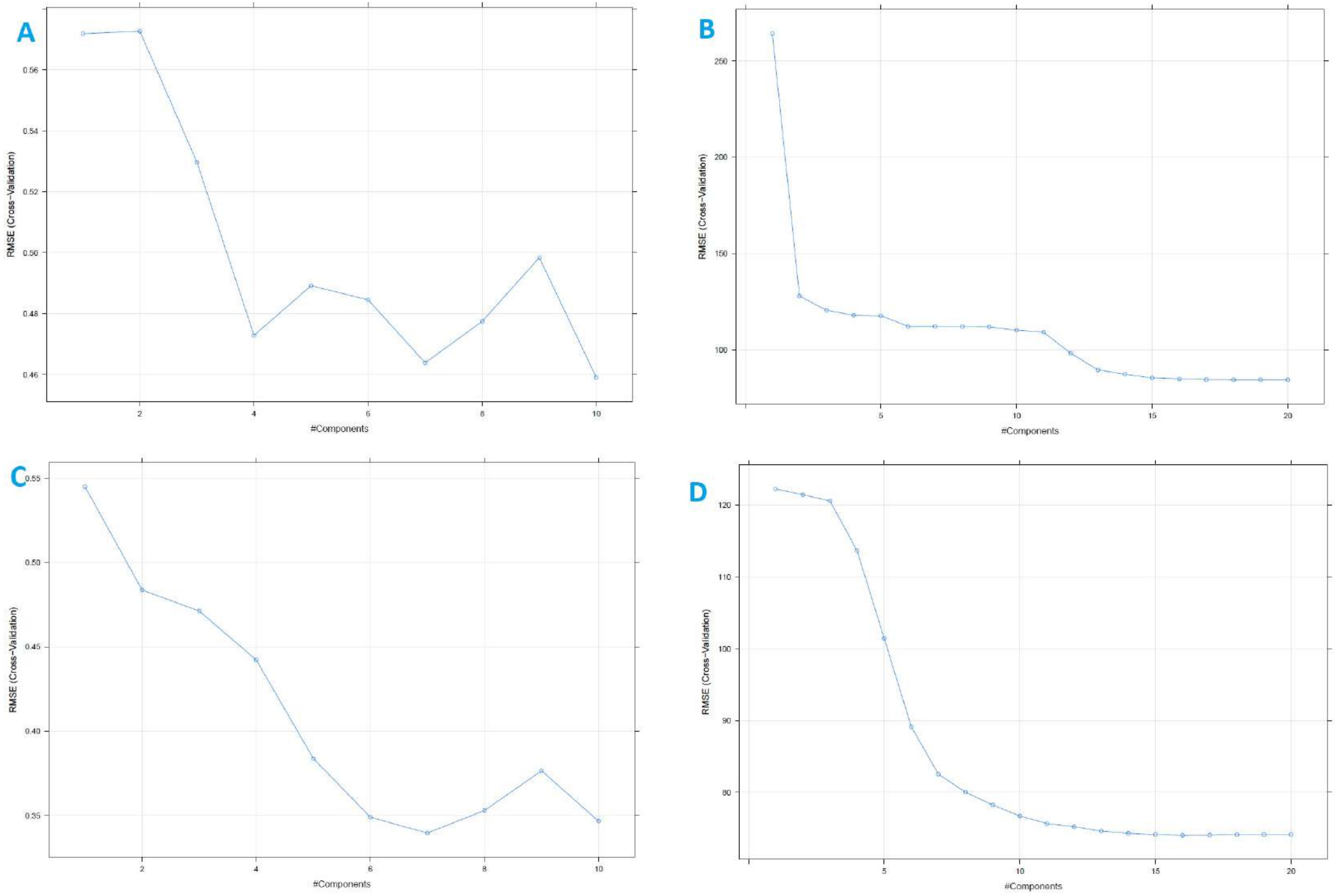

Sucrose: PCR - Standardized data (a), PCR - Augmented data (b), PLS - Standardized data (c), PLS - Augmented data (d)
